# Meta-Analyses of the Effects of Habitual Running on Indices of Health in Physically Inactive Adults

**DOI:** 10.1007/s40279-015-0359-y

**Published:** 2015-07-16

**Authors:** Luiz Carlos Hespanhol Junior, Julian David Pillay, Willem van Mechelen, Evert Verhagen

**Affiliations:** Department of Public and Occupational Health and the EMGO+ Institute for Health and Care Research, VU University Medical Center, Van der Boechorststraat 7, 1081 BT Amsterdam, The Netherlands; Department of Basic Medical Sciences, Durban University of Technology, PO Box 1334, Durban, 4001 South Africa

## Abstract

**Background:**

In order to implement running to promote physical activity, it is essential to quantify the extent to which running improves health.

**Objective:**

The aim was to summarise the literature on the effects of endurance running on biomedical indices of health in physically inactive adults.

**Data Sources:**

Electronic searches were conducted in October 2014 on PubMed, Embase, CINAHL, SPORTDiscus, PEDro, the Cochrane Library and LILACS, with no limits of date and language of publication.

**Study Selection:**

Randomised controlled trials (with a minimum of 8 weeks of running training) that included physically inactive but healthy adults (18–65 years) were selected. The studies needed to compare intervention (i.e. endurance running) and control (i.e. no intervention) groups.

**Study Appraisal and Synthesis Methods:**

Two authors evaluated study eligibility, extracted data, and assessed risk of bias; a third author resolved any uncertainties. Random-effects meta-analyses were performed to summarise the estimates for length of training and sex. A dose-response analysis was performed with random-effects meta-regression in order to investigate the relationship between running characteristics and effect sizes.

**Results:**

After screening 22,380 records, 49 articles were included, of which 35 were used to combine data on ten biomedical indices of health. On average the running programs were composed of 3.7 ± 0.9 sessions/week, 2.3 ± 1.0 h/week, 14.4 ± 5.4 km/week, at 60–90 % of the maximum heart rate, and lasted 21.5 ± 16.8 weeks. After 1 year of training, running was effective in reducing body mass by 3.3 kg [95 % confidence interval (CI) 4.1–2.5], body fat by 2.7 % (95 % CI 5.1–0.2), resting heart rate by 6.7 min^−1^ (95 % CI 10.3–3.0) and triglycerides by 16.9 mg dl^−1^ (95 % CI 28.1–5.6). Also, running significantly increased maximal oxygen uptake (*V*O_2max_) by 7.1 ml min^−1^ kg^−1^ (95 % CI 5.0–9.1) and high-density lipoprotein (HDL) cholesterol by 3.3 mg dl^−1^ (95 % CI 1.2–5.4). No significant effect was found for lean body mass, body mass index, total cholesterol and low-density lipoprotein cholesterol after 1 year of training. In the dose-response analysis, larger effect sizes were found for longer length of training.

**Limitations:**

It was only possible to combine the data of ten out the 161 outcome measures identified. Lack of information on training characteristics precluded a multivariate model in the dose-response analysis.

**Conclusions:**

Endurance running was effective in providing substantial beneficial effects on body mass, body fat, resting heart rate, *V*O_2max_, triglycerides and HDL cholesterol in physically inactive adults. The longer the length of training, the larger the achieved health benefits. Clinicians and health authorities can use this information to advise individuals to run, and to support policies towards investing in running programs.

**Electronic supplementary material:**

The online version of this article (doi:10.1007/s40279-015-0359-y) contains supplementary material, which is available to authorized users.

## Key Points

**Table:** 

Endurance running was found to be beneficial for health in physically inactive adults with regards to body mass, body fat, resting heart rate, maximal oxygen uptake, triglycerides and high-density lipoprotein cholesterol.
The effects of running on biomedical indices of health are beneficially correlated to running exposure.
Clinicians and health authorities can use this information to advise individuals to run, and also to support policies towards investing in running programs.

## Introduction

Physical inactivity is a leading risk factor for mortality, accounting for millions of deaths per year [1]. Consequently, physical inactivity is a global public health concern [2] as a contributor to the worldwide epidemic of non-communicable diseases [3]. Increasing physical activity levels throughout the population is a major challenge for the 21st century [2, 4]. Societal trends, nonetheless, show a steady decline in physical activity levels [4]. Commitment to change this scenario is therefore critical [2], and efforts are constantly made towards promoting a physically active lifestyle, the health benefits of which are well documented [5–8].

Regular running is a popular mode of physical activity [9], undertaken by many individuals seeking a healthier lifestyle [10]. It is easy to perform, it has a social component, and it is relatively inexpensive, time efficient and easily accessible [10, 11]. The high popularity and accessibility of running is seen as a strong contributor towards promoting and enhancing a physically active lifestyle within the population [11]. In order to ensure effective running programs that promote physical activity, and consequently to reduce the risk of lifestyle-related diseases, it is essential to quantify the extent to which running improves health. Such information is valuable in identifying target populations for specific physical activity programs and, more importantly, towards increasing the effectiveness thereof [4]. The aim of this study was, therefore, to summarise the evidence on the effects of endurance running on biomedical indices of health in physically inactive adults.

## Methods

### Eligibility Criteria

Studies were considered for inclusion if they were randomised controlled trials published in peer-reviewed journals; included physically inactive but healthy adults (18–65 years) at baseline; studied an endurance type of running intervention; compared the effects of endurance running to a group not engaged in any physical activity intervention; provided a follow-up of 8 or more weeks; and included at least one biomedical health indicator (indices of health) as an outcome measure. Physically inactive participants were considered if the studies clearly stated that the participants were physically inactive or sedentary, or if they did not reach the physical activity recommendations at baseline [12].

Studies were not included if they aimed to evaluate the effects of running exclusively on performance or neuromuscular outcomes, as these were not considered to be general indices of health; involved a very specific sample that could be significantly different from the general population at baseline (e.g. obese) and, therefore, may respond differently to the running training, providing a biased estimate; provided an intervention composed of running in combination with something else (e.g. diet), as these interventions do not reflect the independent effect of running; and included less than 8 weeks of training, in order to ensure a reasonable time for physiological responses.

### Information Sources and Search Strategy

Systematic electronic searches were conducted in October 2014 on PubMed, Embase, CINAHL, SPORTDiscus, PEDro, the Cochrane Library and LILACS. The searches were structured following the Cochrane Collaboration recommendations [13] and were not limited by date or language of publication. The detailed search strategy for each database can be found in Electronic Supplementary Material Appendix S1. Reference lists of included articles were also accessed to search for additional studies that might be eligible for inclusion.

### Study Selection and Data Collection Process

The selection process involved screening of titles and reading abstracts of the retrieved search results. The full texts of potentially relevant articles were subsequently obtained and analysed to check eligibility. The data were collected using a standardised data extraction form, which can be found in Electronic Supplementary Material Appendix S2. Two authors (LCHJ, JDP) evaluated the eligibility and extracted the relevant data of each article independently. In cases of uncertainty, a third author (EV) provided a consensus.

### Risk of Bias Assessment

The risk of bias of all included studies was assessed by the Cochrane Collaboration’s tool for assessing risk of bias of randomised trials [14]. This tool comprises seven items assessing selection bias (random sequence generation and allocation concealment), performance bias (blinding of participants and personnel), detection bias (blinding of outcome assessment), attrition bias (incomplete outcome data), reporting bias (selective reporting) and other sources of bias. In addition, it was assessed if the analysis was conducted on the basis of the intention-to-treat principle. The judgment was achieved in order to inform low risk of bias (i.e. criterion satisfied and clearly described in the article), high risk of bias (i.e. criterion not satisfied) and unclear risk of bias (i.e. insufficient information to permit judgment). Two authors (LCHJ, JDP) assessed each item independently, and in cases of uncertainties, a consensus was obtained through discussion and/or arbitration by a third author (EV).

### Data Analysis

In order to summarise the effects of running on biomedical indices of health, the mean change from baseline and its standard deviation (SD) were used. In cases where the mean change from baseline was not available, but the study provided the mean at baseline and the mean after the follow-up, the mean change was calculated. In cases where the SD was not available, but the study provided another uncertainty measure as standard error or confidence intervals (CIs), the SD was estimated according to the Cochrane Collaboration recommendations [13]. Studies that did not provide sufficient data (e.g. number of participants, mean values or uncertainty measures for each group) were not included in the meta-analysis for that particular outcome. Duplicated results (articles related to the same study, but published as per different purposes) were considered in the meta-analysis only once for each outcome measure. The criteria used in deciding which duplicated result would be considered for the meta-analysis were based on (1) the primary aim of the study; (2) the date of publication, with preference for the earliest date; and (3) the number of participants, with preference for the largest sample available.

Random-effects meta-analyses were used to summarise the results of each outcome measure. The summary measure was the combined mean difference weighted by the inverse of the variance within and between (tau-squared) studies, and its 95 % CI. Heterogeneity was assessed by the *I*^2^ estimate. Subgroup analyses were performed in order to explain the effect variations by length of training and sex that were hypothesised before the analyses. Therefore, only the outcome measures that had at least ten comparisons between running and control groups were included in the meta-analysis, otherwise the subgroups analysis would not be possible. Outcome measures that did not meet this criterion were summarised descriptively.

A dose-response analysis was performed in order to investigate the relationship between running characteristics and effect sizes. Univariate linear meta-regressions with random effects were performed using the mean difference between running and control groups as the dependent variable (effect size), and the running characteristics (length of training, frequency, duration, distance, intensity and speed) as numeric linear predictors. Larger studies had more influence in the meta-regressions than smaller studies, and residual heterogeneity among effect sizes not modelled by the running characteristics was also considered in the analyses (random-effects) [13]. The summary measure was the linear regression coefficient (*β*) and its 95 % CI.

Meta-analyses and meta-regressions were conducted in Stata/SE 12.0 (StataCorp, College Station, Texas, USA), using the commands metan and metareg, respectively. Statistically significant results were considered for the estimates with the 95 % CI not including zero [15].

## Results

### Selection of the Studies

**Fig. 1:**
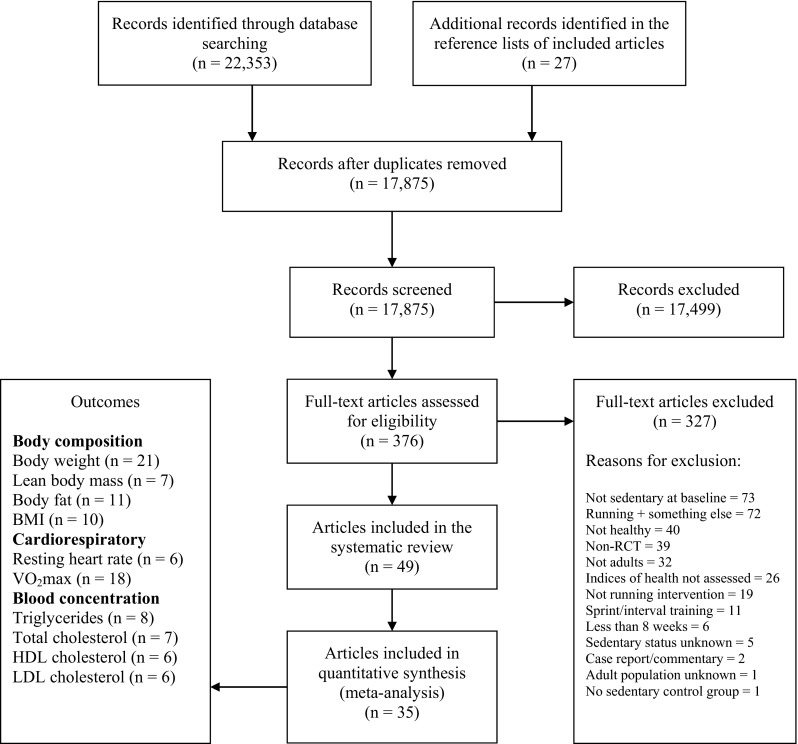
Flow of the studies during the selection process. The databases searched were: PubMed, Embase, Cumulative Index to Nursing and Allied Health Literature (CINAHL), SPORTDiscus, Physiotherapy Evidence Database (PEDro), the Cochrane Library and Latin American and Caribbean Center on Health Sciences Information (LILACS). *BMI* body mass index, *HDL* high-density lipoprotein, *LDL* low-density lipoprotein, *RCT* randomised controlled trial, *VO*_*2max*_ maximal oxygen uptake

A total of 22,380 records were retrieved, 22,353 from the electronic search strategy and 27 from references of included articles. Of the 17,875 unique records retrieved (duplicates removed), 49 articles were considered eligible. Only 35 articles, however, presented sufficient and original data and were, therefore, included in the meta-analysis. Figure 1 presents the flow diagram of the selection process.

### Description of the Included Articles

The 49 articles included in this systematic review were published between 1980 and 2014. The sample size ranged from 14 to 120, with a mean of 41.3 ± 25.5 (mean ± SD) participants. The total sample was composed of 2024 participants (79 % males, *n* = 1592; 21 % females, *n* = 432), aged 33.8 ± 10.2 years. In the control groups, the sample size ranged from 6 to 60 (17.2 ± 11.9), and the total was 844 participants (78 % males, *n* = 658; 22 % females, *n* = 186), aged 34.1 ± 9.9 years.

In the running groups, the sample size ranged from 7 to 60 (24.1 ± 14.6), and the total was 1180 participants (79 % males, *n* = 934; 21 % females, *n* = 246), aged 34.2 ± 9.1 years. On average the running programs were composed of 3.7 ± 0.9 sessions/week, 2.3 ± 1.0 h/week, 14.4 ± 5.4 km/week, and ranged from 60 to 90 % of the maximum heart rate (77.6 ± 6.3 %), and the length of training was 21.5 ± 16.8 weeks. Detailed description of the data extracted from the included articles can be found in Electronic Supplementary Material Appendix S2.

### Risk of Bias Assessment

Table 1 presents the risk of bias assessment of all included articles. In general, underreporting of information was identified as a main concern. Consequently, it was difficult to judge certain criteria because of missing information. Most articles did not clearly describe the method of randomisation (90 %, *n* = 44), whether the allocation of the participants to study groups was concealed (90 %, *n* = 44), information on the study protocol (82 %, *n* = 40) and whether or not the analysis was conducted on the basis of the intention-to-treat principle (98 %, *n* = 48). A low risk of bias was achieved for most of the studies regarding incomplete outcome data (73 %, *n* = 36) and other sources of bias (100 %, *n* = 49). Blinding of participants provided the highest risk of bias, which can be expected because of the nature of the intervention.

**Table 1:** Risk of bias assessment of included studies

References	Year	Cochrane Collaboration tool [14]							Intention-to-treat analysis
		Randomisation	Concealed allocation	Blinding of participants and personnel	Blinding of outcome assessment	Incomplete outcome data	Selective reporting	Other bias	
Moghadasi and Mohammadi Domieh [62]	2014	?	?	−	−	+	?	+	?
Celik et al. [60]	2013	+	+	−	Blood samples: + Physical/physiological: −	+	?	+	?
Gregory et al. [63]	2013	?	?	−	−	−	?	+	?
Asad et al. [64]	2012	?	?	−	−	+	?	+	?
Hosseini et al. [65]	2012	?	?	−	−	+	?	+	?
Lo et al. [66]	2011	?	?	−	−	+	?	+	?
Andersen et al. [67]	2010	?	?	−	Echocardiography: + *V*O_2max_/tissue Doppler: −	+	?	+	?
Hendrickson et al. [30]	2010	?	?	−	−	?	−	+	?
Krustrup et al. [68]	2010	−	−	−	Echocardiography: + DXA, BP, RHR, FG: −	−	?	+	?
Nindl et al. [69]	2010	?	?	−	−	+	−	+	?
Ozdemir et al. [61]	2010	?	?	−	−	+	?	+	?
Sedlock et al. [25]	2010	?	?	−	−	+	?	+	?
Lee et al. [70]	2009	?	?	−	−	+	?	+	?
Lester et al. [71]	2009	+	?	−	−	+	?	+	?
Brixius et al. [72]	2008	?	?	−	−	+	?	+	?
Meyer et al. [17]	2007	?	?	−	−	+	?	+	?
Ring-Dimitriou et al. [31]	2007	?	?	−	Blood samples: + BC, aerobic fitness: −	−	?	+	?
Beneke and Hutler [36]	2005	?	?	−	−	+	?	+	?
Hautala et al. [73]	2004	?	?	−	−	+	?	+	?
Poehlman et al. [74]	2000	?	?	−	−	−	?	+	?
Bourque et al. [75]	1997	?	?	−	−	−	−	+	?
Hubinger and Mackinnon [76]	1996	?	?	−	−	−	?	+	?
Suter et al. [77]	1994	?	?	−	−	+	?	+	?
Garber et al. [78]	1992	?	?	−	−	−	?	+	?
Suter and Marti [26]	1992	?	?	−	−	+	?	+	?
Williams et al. [79]	1992	?	?	−	−	+	?	+	?
Oja et al. [33]	1991	?	?	−	−	+	−	+	?
Marti et al. [28]	1990	?	?	−	−	+	?	+	+
Suter et al. [29]	1990	?	?	−	−	+	?	+	?
Williams et al. [80]	1990	?	+	−	−	+	?	+	?
Williams et al. [35]	1990	?	?	−	−	+	−	+	?
Moses et al. [18]	1989	?	?	−	−	−	−	+	?
Williams et al. [27]	1989	?	?	−	−	+	?	+	?
Wood et al. [34]	1988	?	+	−	−	+	?	+	?
Juneau et al. [81]	1987	?	?	−	−	+	−	+	?
Allen et al. [32]	1986	?	?	−	−	+	?	+	?
Gossard et al. [16]	1986	?	?	−	−	+	?	+	?
Hagan et al. [82]	1986	?	?	−	−	+	−	+	?
Mueller et al. [19]	1986	?	?	−	−	?	?	+	?
Savage et al. [20]	1986	?	?	−	−	+	?	+	?
Thomas et al. [83]	1985	?	?	−	−	−	?	+	?
Iltis et al. [21]	1984	?	?	−	−	+	?	+	?
Mathur and Toriola [23]	1984	?	?	−	−	+	?	+	?
Thomas et al. [22]	1984	−	?	−	−	−	?	+	?
Toriola [24]	1984	?	?	−	−	+	?	+	?
Williams et al. [84]	1983	?	?	−	−	−	?	+	?
Wood et al. [85]	1983	?	+	−	−	+	?	+	?
Williams et al. [86]	1982	?	?	−	−	+	?	+	?
Wilmore et al. [87]	1980	+	?	−	−	+	−	+	?

Source of bias: selection bias (randomisation and concealed allocation), performance bias (blinding of participants and personnel), detection bias (blinding of outcome assessment), attrition bias (incomplete outcome data: >20 %), reporting bias (selective reporting) and other source of bias

*BC* body composition, *BP* blood pressure, *DXA* dual energy X-ray absorptiometry, *FG* fasting glucose, *RHR* resting heart rate, *VO*_*2max*_ maximal oxygen uptake, *+* low risk of bias (plausible bias unlikely to seriously alter the results), *−* high risk of bias (plausible bias that seriously weakens confidence in the results), ? unclear risk of bias (plausible bias that raises some doubt about the results)

### Effects of Running on Biomedical Indices of Health: Meta-Analysis

A total of 161 biomedical indices of health were collectively investigated in the 49 articles included in the systematic review (Electronic Supplementary Material Appendix S3). Outcome measures were classified into three groups: body composition, cardiorespiratory measures and blood serum concentrations. Some of the studies compared more than one running group (i.e. high/moderate-intensity and low-intensity training [16–20]; 4- and 2-mile training [21, 22]; or 4.8-, 3.2- and 1.6-km training [23, 24]) to a control group, allowing multiple comparisons.

Meta-analyses were possible for ten outcome measures, and the main findings are summarised in Table 2. Forest plots, with detailed information of the number of studies and the results of individual studies included in each meta-analysis can be found in Electronic Supplementary Material Appendix S4.

**Table 2:** Meta-analyses on the effects of running on biomedical indices of health

Outcome measure	Subgroup analysis by length of training			Subgroup analysis by sex	
	12 weeks WMD (95 % CI)	26 weeks WMD (95 % CI)	52 or 69 weeks WMD (95 % CI)	Males WMD (95 % CI)	Females WMD (95 % CI)
Body composition					
Body mass (kg)	−0.9 (−2.6 to 0.8)	−0.9 (−3.6 to 1.9)	−3.3 (−4.1 to −2.5)*	−3.1 (−3.8 to −2.3)*	−0.6 (−2.5 to 1.4)
Heterogeneity	*I* ^2^ = 0 %	*I* ^2^ = 0 %	*I* ^2^ = 0 %	*I* ^2^ = 0 %	*I* ^2^ = 0 %
Lean body mass (kg)	−0.6 (−2.0 to 0.8)	−0.1 (−0.6 to 0.3)	−0.1 (−2.2 to 2.1)	−0.2 (−1.2 to 0.9)	−0.3 (−1.5 to 0.8)
Heterogeneity	*I* ^2^ = 0 %	*I* ^2^ = 0 %	*I* ^2^ = 85 %	*I* ^2^ = 71 %	*I* ^2^ = 0 %
Body fat (%)	−1.3 (−2.0 to −0.6)*	−1.9 (−2.8 to −1.0)*	−2.7 (−5.1 to −0.2)*	−1.8 (−2.3 to −1.3)*	0.4 (−1.7 to 2.6)
Heterogeneity	*I* ^2^ = 0 %	*I* ^2^ = 0 %	*I* ^2^ = 24 %	*I* ^2^ = 0 %	*I* ^2^ = 0 %
BMI (kg m^−2^)	−0.6 (−2.2 to 1.0)	−0.2 (−0.6 to 0.2)	−0.2 (−1.4 to 1.0)	−0.3 (−0.7 to 0.2)	0.0 (−1.1 to 1.1)
Heterogeneity	*I* ^2^ = 0 %	*I* ^2^ = 0 %	*I* ^2^ = 0 %	*I* ^2^ = 0 %	*I* ^2^ = 0 %
Cardiorespiratory					
Resting heart rate (min^−1^)	−3.4 (−5.6 to −1.2)*	−3.5 (−6.7 to −0.3)*	−6.7 (−10.3 to −3.0)*	−4.6 (−6.5 to −2.7)*	−2.6 (−5.7 to 0.5)
Heterogeneity	*I* ^2^ = 0 %	*I* ^2^ = 0 %	*I* ^2^ = 0 %	*I* ^2^ = 0 %	*I* ^2^ = 0 %
*V*O_2max_ (ml min^−1^ kg^−1^)	3.8 (3.1 to 4.6)*	4.1 (3.0 to 5.1)*	7.1 (5.0 to 9.1)*	4.6 (3.9 to 5.3)*	3.0 (1.7 to 4.3)*
Heterogeneity	*I* ^2^ = 0 %	*I* ^2^ = 0 %	*I* ^2^ = 27 %	*I* ^2^ = 4 %	*I* ^2^ = 0 %
Blood serum concentration					
Triglycerides (mg dl^−1^)	−12.9 (−24.6 to −1.2)*	−6.0 (−26.1 to 14.1)	−16.9 (−28.1 to −5.6)*	−13.8 (−21.8 to −5.8)*	−15.2 (−38.6 to 8.2)
Heterogeneity	*I* ^2^ = 0 %	*I* ^2^ = 0 %	*I* ^2^ = 0 %	*I* ^2^ = 0 %	*I* ^2^ = 0 %
Total cholesterol (mg dl^−1^)	−5.3 (−10.0 to −0.5)*	11.7 (1.4 to 22.0)*	−2.2 (−7.8 to 3.3)	−2.5 (−6.0 to 1.1)	0.4 (−14.2 to 15.0)
Heterogeneity	*I* ^2^ = 0 %	*I* ^2^ = 0 %	*I* ^2^ = 0 %	*I* ^2^ = 0 %	*I* ^2^ = 8 %
HDL cholesterol (mg dl^−1^)	−0.2 (−3.7 to 3.4)	2.0 (−1.0 to 4.9)	3.3 (1.2 to 5.4)*	2.6 (1.0 to 4.2)*	2.8 (−3.0 to 8.6)
Heterogeneity	*I* ^2^ = 0 %	*I* ^2^ = 2 %	*I* ^2^ = 41 %	*I* ^2^ = 30 %	*I* ^2^ = 9 %
LDL cholesterol (mg dl^−1^)	−0.7 (−11.7 to 10.4)	9.8 (−2.3 to 21.9)	−2.6 (−7.6 to 2.4)	−0.4 (−4.9 to 4.2)	0.3 (−13.6 to 14.2)
Heterogeneity	*I* ^2^ = 0 %	*I* ^2^ = 0 %	*I* ^2^ = 0 %	*I* ^2^ = 0 %	*I* ^2^ = 0 %

All estimates came from the random-effects meta-analysis comparing the intervention groups (endurance running) with the control groups (physically inactive)

*BMI* body mass index, *CI* confidence interval, *HDL* high-density lipoprotein, *LDL* low-density lipoprotein, *VO*_*2max*_ maximal oxygen uptake, *WMD* weighted mean difference

* Statistically significant estimate (95% CI around the WMD not including zero)

#### Body Composition Outcomes

Meta-analyses were possible for four body composition outcome measures: body mass, lean body mass, percentage body fat and body mass index (BMI). A statistically significant reduction was found for body mass and percentage body fat in favour of the running group after 1 year of training. A greater reduction was found in males for both of these outcome measures. Differences in lean body mass and BMI were not statistically significant.

#### Cardiorespiratory Outcomes

Meta-analyses were possible for two cardiorespiratory outcome measures: resting heart rate and maximal oxygen uptake (*V*O_2max_ in ml min^−1^ kg^−1^). A statistically significant reduction in resting heart rate was found after 12, 26 and 52 weeks, and also in males. Statistically significant increases in *V*O_2max_ were found for all subgroup categories, with a larger effect size after 1 year of training and in males. The longer the length of training, the larger the effect of running on resting heart rate and *V*O_2max_.

#### Blood Serum Concentration Outcomes

Meta-analyses were possible for four blood serum concentration outcome measures: triglycerides, total cholesterol, high-density lipoprotein (HDL) cholesterol and low-density lipoprotein (LDL) cholesterol. Statistically significant reductions were found for triglycerides and statistically significant increases were found for HDL cholesterol in favour of the running group after 1 year of training and in males. Conflicting results were found for total cholesterol after 12 and 26 weeks of training. Differences in LDL cholesterol were not statistically significant.

#### Heterogeneity

Table 2 presents results on heterogeneity. Most of the meta-analyses revealed low inconsistencies. Moderate heterogeneity was found in the meta-analyses for body fat (*I*^2^ = 24 %), *V*O_2max_ (*I*^2^ = 27 %) and HDL cholesterol (*I*^2^ = 41 %) after 1 year of training, and for HDL cholesterol in males (*I*^2^ = 30 %).

### Dose-Response Analysis: Meta-Regression

A total of 34 articles (97 %) reported data on running frequency (sessions/week), 31 (89 %) on duration (h/week) and intensity (percentage of maximum heart rate), and nine (26 %) on distance (km/week). Sufficient data were provided in order to perform a dose-response analysis for length of training, running frequency, duration, distance and intensity. In addition, it was possible to calculate and analyse the average running speed by dividing the weekly distance by the weekly duration (7.9 ± 3.3 km/h) in nine articles (26 %).

Table 3 describes the meta-regression results. Longer length of training was statistically significantly associated with a reduction in body mass. Furthermore, longer length of training was statistically significantly associated with an increase in *V*O_2max_. Both associations indicated larger health benefits for longer running programs. However, an increase in weekly duration (h/week) was statistically significantly associated with a decrease in

**Table 3:** Dose-response analysis on the effects of running on biomedical indices of health

Outcome measure	Length of training (weeks) *β* (95 % CI)	Frequency (sessions/week) *β* (95 % CI)	Duration (h/week) *β* (95 % CI)	Distance (km/week) *β* (95 % CI)	Intensity (%HR_max_) *β* (95 % CI)	Speed (km/h) *β* (95 % CI)
Body composition						
Body mass (kg)	−0.06 (−0.12 to −0.01)*	−0.19 (−1.63 to 1.24)	0.55 (−0.90 to 2.00)	−0.20 (−0.62 to 0.23)	0.25 (−0.02 to 0.52)	–
Lean body mass (kg)	0.00 (−0.05 to 0.06)	−0.46 (−1.45 to 0.53)	−0.33 (−1.09 to 0.44)	–	–	–
Body fat (%)	−0.05 (−0.09 to 0.00)	0.07 (−0.78 to 0.92)	0.22 (−0.35 to 0.79)	–	0.24 (−0.03 to 0.52)	–
BMI (kg m^−2^)	0.00 (−0.06 to 0.07)	−0.04 (−0.79 to 0.71)	−0.21 (−1.11 to 0.68)	–	–	–
Cardiorespiratory						
Resting heart rate (min^−1^)	−0.07 (−0.18 to 0.04)	1.60 (−0.45 to 3.66)	1.50 (−0.49 to 3.48)	–	–	–
*V*O_2max_ (ml min^−1^ kg^−1^)	0.07 (0.03 to 0.11)*	−0.10 (−0.88 to 0.68)	−0.71 (−1.32 to −0.11)*	–	−0.07 (−0.30 to 0.15)	–
Blood serum concentration						
Triglycerides (mg dl^−1^)	−0.08 (−0.57 to 0.41)	−2.48 (−14.61 to 9.65)	−1.87 (−9.52 to 5.78)	−0.85 (−3.08 to 1.37)	–	1.19 (−2.08 to 4.47)
Total cholesterol (mg dl^−1^)	0.06 (−0.18 to 0.29)	4.40 (−1.13 to 9.93)	3.52 (−0.06 to 7.09)	0.27 (−0.69 to 1.22)	–	−0.59 (−1.96 to 0.78)
HDL cholesterol (mg dl^−1^)	0.07 (−0.03 to 0.18)	−2.10 (−6.29 to 2.09)	1.71 (−2.12 to 5.53)	–	–	–
LDL cholesterol (mg dl^−1^)	−0.19 (−0.51 to 0.12)	4.67 (−5.32 to 14.65)	–	–	–	–

All estimates came from the random-effects meta-regression using the mean difference between the intervention groups (endurance running) and the control groups (physically inactive) as the dependent variable, and the running characteristics as numeric linear predictors. The average of the weekly running speed (km/h) was calculated dividing the weekly running distance (km/week) by the weekly duration (h/week)

*β* linear regression coefficient, *%HR*_*max*_ percentage of maximum heart rate, *BMI* body mass index, *CI* confidence interval, *HDL* high-density lipoprotein, *LDL* low-density lipoprotein, *VO*_*2max*_ maximal oxygen uptake, “–” not applicable

* Statistically significant estimate (95 % CI around *β* not including zero)

*V*O_2max_.

### Biomedical Indices of Health Not Included in the Meta-Analysis

It was not possible to perform meta-analyses for 151 indices of health (Electronic Supplementary Material Appendix S3). Within the body composition category, there were only three outcome measures evaluated by more than two studies: body fat-free mass, sum of skin-folds and waist/hip ratio. Five studies evaluated body fat-free mass, with only one of these studies showing a statistically significant increase in the running group compared with the control group [25]. Seven studies evaluated the sum of skin-folds, two of which showed a statistically significant decrease in favour of the running group [20, 26]. One of these studies additionally found an increase in sum of skin-folds in the low-intensity running group compared with the control group [20]. Eight studies evaluated waist/hip ratio, one of which indicated a statistically significant decrease in favour of the running group [27], whilst two of the studies reported a statistically significant increase [28, 29].

Within the cardiorespiratory category, there were nine outcome measures evaluated by more than two studies: a change in submaximal heart rate at a fixed exercise intensity, left ventricular diameter in the end of the systole, left ventricular diameter at the end of the diastole, left ventricular posterior wall thickness at the end of the diastole, systolic blood pressure, diastolic blood pressure, peak oxygen uptake (*V*O_2peak_), maximum pulmonary ventilation (VE_max_) and respiratory exchange ratio. Of the five studies that evaluated *V*O_2peak_, two studies found a statistically significant increase in favour of the running group [30, 31]. Of the three studies that evaluated VE_max_, two reported a statistically significant increase in favour of the running group [32, 33]. No statistically significant results were found for the other seven cardiorespiratory outcome measures.

Within the blood serum concentration category, there were 16 outcome measures evaluated by more than two studies: fasting glucose, fasting insulin, total cholesterol/HDL ratio, HDL/total cholesterol ratio, lecithin:cholesterol acyltransferase (LCTA), HDL2 subfraction, HDL3 subfraction, small LDL, large LDL, LDL peak flotation rate, very low-density lipoprotein (VLDL), intermediate-density lipoprotein, apolipoprotein A-I, apolipoprotein A-II, apolipoprotein B and lactate. A statistically significant decrease, in favour of the running group, was found in one of the four studies that evaluated glucose [23] and total cholesterol/HDL ratio [34]. Of the seven studies that evaluated HDL2 and HDL3 subfractions, two studies found a statistically significant increase in favour of the running group [34, 35]. Of the six studies that evaluated VLDL, two studies found a statistically significant decrease in favour of the running group [28, 35]. Ten and nine studies evaluated apolipoprotein A-I and B, respectively. Only one study, however, found a statistically significant increase in favour of the running group for apolipoprotein A-I and a statistically significant decrease for apolipoprotein B [31]. Of the four studies that evaluated lactate, only one study found a statistically significant decrease in favour of the running group [36]. No statistically significant results were found for the other eight blood serum concentration outcome measures.

## Discussion

This study was a comprehensive systematic review aiming to summarise the evidence about the effects of endurance running on biomedical indices of health. Running provided a beneficial effect on body mass, body fat, resting heart rate, *V*O_2max_, triglycerides and HDL cholesterol. In general, larger effects were observed with longer length of training and in males. With regards to the running dose, the results also suggested that the effect of running on body mass and *V*O_2max_ was larger with longer length of training.

### Risk of Bias

Underreporting was the main factor identified in the risk of bias assessment of the included randomised controlled trials. One explanation could be that most of the studies were published before the Consolidated Standards of Reporting Trials (CONSORT) statement [37]. Studies published after CONSORT, however, presented similar underreporting issues. Owing to the nature of the intervention, the blinding of participants and personnel presented a high risk of bias in all studies. The blinding of participants and personnel is challenging or almost impossible in physical activity interventions [37]. However, blinding of the outcome assessment is often achievable [37]. It was, therefore, surprising to note the high risk of bias judgment in most articles regarding blinding of outcome assessment. Researchers should be aware about reporting all relevant methodological information in randomised controlled trials, and also about designing studies with the lowest risk of bias as possible.

### Possible Mechanisms of the Effects of Endurance Running

The reduction found in body mass can be explained by the reduction in percentage body fat with no significant changes in lean body mass. Prolonged endurance exercise training is known to increase lipids metabolism during exercise [38]. This is probably the most reasonable mechanism explaining the reduction in body fat (and consequently in body mass), and also explains the effect of running on triglycerides (an important fat substrate [39]) and on HDL cholesterol [40]. The reduction in resting heart rate could be explained by adaptations of exercise such as increases in blood volume [41] and reductions in sympathetic and/or increases in parasympathetic autonomic control at rest [42]. The increase in relative *V*O_2max_ (ml min^−1^ kg^−1^) could be partially explained by the reduction in body mass, and partially by physiological adaptions of exercise. Increases in stroke volume and cardiac output (as a result of the increased blood volume caused by the exercise training [41]) can increase the oxygen delivery [43].

Physical activity has been considered as a drug [44] because of the similarities in health benefits achieved by both [45]. An essential aspect of physical activity, therefore, relates to dosage [46]. The effects of running on body mass and *V*O_2max_ were larger for longer length of training (1 additional week of running training reduces the body mass by 0.06 kg and increases the *V*O_2max_ by 0.07 ml min^−1^ kg^−1^), and this trend was consistent with all other outcomes. There is an evident explanation for these results: the longer one exercises, the larger the benefits one achieves. However, larger effect sizes were achieved with shorter weekly duration for *V*O_2max_, and although this result was counterintuitive with our previous hypothesis, studies have shown that the duration of exercise per session is not a suitable characteristic to be manipulated in order to enhance cardiorespiratory outcomes [47, 48]. On the other hand, training intensity seems to play an important role [47–49], but the results of this systematic review were inconclusive in this regard.

### Implications for Practice

Endurance running was found to be beneficial for health with regard to biomedical indices of health related to cardiovascular disease, and also presented beneficial dose-response relationships. Clinicians can use the outcomes of the current systematic review to advise running in order to improve health in physically inactive adults. Outcomes of this review can also be used by (public) health authorities to support policies towards investing in running programs, therefore, combating physical inactivity, which is a leading risk factor for mortality [1]. This rationale is of particular significance for public health, as running is well known to be easily accessible and relatively inexpensive to implement [10, 11].

After evidencing that an intervention is effective, the next step is to evaluate if the intervention is implementable in the real world (i.e. in the non-controlled environment) [50]. One important aspect of implementation is to investigate whether or not individuals continue to adhere to the intervention after the study ends. Unfortunately, no studies included in this systematic review investigated this issue. However, Ooms et al. [11] showed that 4.5 months after the end of a 6-week start-to-run program, 69 % of the participants in the start-to-run group were still running and they were spending 152 more min/week (95 % CI 80–223) in vigorous-intensity physical activities, and 107 more min/week (95 % CI 69–145) in sports activities compared with the control group. Therefore, available evidence suggests that promoting running in order to decrease physical inactivity is effective, beneficial for health, implementable and sustainable in the short term [11, 51, 52]. However, more studies investigating implementation issues of running programs are needed, especially in the long term.

The results of this systematic review were based on running programs designed for physically inactive adults. Therefore, the running volume and/or intensity were progressively increased and sometimes walking was allowed or an integral part of the running programs (detailed descriptions of the running programs can be found in Electronic Supplementary Material Appendix S2). This characteristic of the running programs could result in an underestimation of the effects of actual running on the biomedical indices of health [52]. Yet, the inclusion of walking reflects the reality of such programs in which participants walk every now and then [11]. Consequently, this increases the external validity of the results throughout the adult inactive population that decides to start running.

### Benefits Versus Risks of Running

Despite the health benefits, running is not free from adverse effects. Although death during running is extremely rare (incidence of 0.39 per 100,000 runners) [53], running has a substantial risk of injuries [54]. The incidence of running injuries in novice runners is about 30 injuries per 1000 h of running exposure [55–57], and these injuries can affect up to 30 % of novice runners in 1 year [58]. However, the health benefits achieved by running in physically inactive individuals outweigh the risks, since running significantly reduces much more severe outcomes, such as death (30 and 45 % lower risk of all-cause and cardiovascular mortality, respectively) [52] and disability [51], which may be partially explained by the results of this systematic review.

### Limitations

This systematic review was conducted in order to compare the running effects with no intervention. The advantages of this approach include the investigation of the crude effect of running and the comparability across studies. The main disadvantage is, however, that this study did not compare running with other types of physical activity, and it may be that other types of physical activity could reach similar health effects. According to Wen et al. [59], a 5-min run is as beneficial as a 15-min walk for the reduction of all-cause mortality; however, to get the same benefits as a 25-min run, one should walk four times longer. Studies have shown that running, cycling and swimming training at the same volume and intensity result in similar effects on *V*O_2max_ in physically inactive individuals [60, 61].

The limitations of this systematic review also include the following: of the 161 biomedical indices of health identified, it was only possible to combine the data for ten indices of health (6 %), which may have yielded an underestimation of the health benefits of endurance running; in some cases, the subgroup analyses were carried out with few studies, which may have limited the ability to draw strong conclusions about some subgroups; and the lack of a proper description of the training characteristics in some of the included papers precluded a multivariate meta-regression analysis investigating the influence of a combination of the training characteristics on the effect sizes.

### Future Recommendations

Important areas for future research were identified on the basis of the current gaps noted. Few studies have investigated important biomedical indices of health, such as blood pressure, insulin and hormones, warranting the need for future studies that explore the effects of running on these indices. Studies included in this systematic review usually included males only; therefore, the effects on females should be further investigated. Most of the studies were conducted with a short follow-up; hence, more long-term studies should be conducted to investigate if the effects of running increase consistently over time or if there is a plateau in this relationship. Additionally, there is a need for studies investigating implementation issues regarding the continuation of running practice after organised running programs end (e.g. after the study has ended).

## Conclusions

Current evidence supports that endurance running is effective in providing beneficial effects on body mass, body fat, resting heart rate, *V*O_2max_, triglycerides and HDL cholesterol in physically inactive adults. In general, the longer the length of training, the larger the achieved health benefits. Further research is necessary to investigate the effectiveness of running on biomedical indices of health for which there was insufficient evidence in this systematic review to enable conclusions to be drawn.

## Electronic supplementary material

Supplementary material 1 (DOCX 6849 kb)
