# Supplementary material for: Meta-Analyses of the Effects of Habitual Running on Indices of Health in Physically Inactive Adults
Source: Sports Med. 2015 Jul 16;45(10):1455–68. doi: 10.1007/s40279-015-0359-y (PMC4579257; doi:10.1007/s40279-015-0359-y)
Supplement: Supplementary file 1 — Supplementary material 1 (DOCX 6849 kb) [file 40279_2015_359_MOESM1_ESM.docx]

**META-ANALYSES OF THE EFFECTS OF HABITUAL RUNNING ON INDICES OF HEALTH IN PHYSICALLY INACTIVE ADULTS**

Journal: Sports Medicine

Luiz Carlos Hespanhol Junior^1^, Julian David Pillay^2^, Willem van Mechelen^1^, Evert Verhagen^1^

1- Department of Public & Occupational Health and the EMGO+ Institute for Health and Care Research, VU University Medical Center, Van der Boechorststraat 7, 1081 BT Amsterdam, The Netherlands

2- Department of Basic Medical Sciences, Durban University of Technology, PO Box 1334, Durban, 4001, South Africa

**Corresponding author:** Evert Verhagen ([e.verhagen@vumc.nl](mailto:e.verhagen@vumc.nl))

**Electronic Supplementary Material**

Table of Contents

Electronic Supplementary Material Appendix S1. Search strategy 6

Description of PICOS: 7

Search Strategy for PubMed: 7

 Part A: Search for studies about running 7

 Part B: Search for studies about biomedical indices of health 7

 Part C: Generic search for randomised controlled trials and longitudinal studies 8

 Full search in PubMed (5734 records) 8

Search Strategy for EMBASE 9

 Part A: Search for studies about running 9

 Part B: Search for studies about biomedical indices of health 9

 Part C: Generic search for randomised controlled trials and longitudinal studies 10

 Full search in EMBASE (2650 records) 11

Search Strategy for Center on Health Sciences Information (CINAHL) [via EBSCO] 11

 Part A: Search for studies about running 11

 Part B: Search for studies about biomedical indices of health 11

 Part C: Generic search for randomised controlled trials and longitudinal studies 13

 Full search in CINAHL (7414 records) 13

Search Strategy for SPORTDiscus [via EBSCO] 13

 Part A: Search for studies about running 13

 Part B: Search for studies about biomedical indices of health 14

 Part C: Generic search for randomised controlled trials and longitudinal studies 15

 Full search in SPORTDiscus (5335 records) 16

Search Strategy for Physiotherapy Evidence Database (PEDro) 16

 Full search in PEDro 16

Search Strategy for The Cochrane Library 16

 Part A: Search for studies about running 16

 Part B: Search for studies about biomedical indices of health 16

 Full search in The Cochrane Library (1122 records) 17

Search Strategy for Latin American and Caribbean Center on Health Sciences Information (LILACS) 18

 Full LILACS search (52 records) 18

Electronic Supplementary Material Appendix S2. Study characteristics 19

Moghadasi M, Mohammadi Domieh A. Effects of Resistance versus Endurance Training on Plasma Lipocalin-2 in Young Men. Asian Journal of Sports Medicine. 2014;4(6):108-14. 22

Celik O, Salci Y, Ak E, Kalaci A, Korkusuz F. Serum cartilage oligomeric matrix protein accumulation decreases significantly after 12 weeks of running but not swimming and cycling training - a randomised controlled trial. The Knee. 2013;20(1):19-25. 24

Gregory SM, Spiering BA, Alemany JA, Tuckow AP, Rarick KR, Staab JS, et al. Exercise-induced insulin-like growth factor I system concentrations after training in women. Medicine and science in sports and exercise. 2013;45(3):420-8. 26

Asad M, Ravasi AA, Faramarzi M, Pournemati P. The effects of three training methods endurance, resistance and concurrent on adiponectin resting levels in overweighed untrained men. Bratislavske lekarske listy. 2012;113(11):664-8. 28

Hosseini M, Piri M, Agha-Alinejad H, Haj-Sadeghi S. The effect of endurance, resistance and concurrent training on the heart structure of female students. Biology of Sport. 2012;29(1):17-21. 30

Lo MS, Lin LL, Yao WJ, Ma MC. Training and detraining effects of the resistance vs. endurance program on body composition, body size, and physical performance in young men. Journal of strength and conditioning research / National Strength & Conditioning Association. 2011;25(8):2246-54. 32

Andersen LJ, Hansen PR, Sogaard P, Madsen JK, Bech J, Krustrup P. Improvement of systolic and diastolic heart function after physical training in sedentary women. Scandinavian journal of medicine & science in sports. 2010;20 Suppl 1:50-7. 35

Hendrickson NR, Sharp MA, Alemany JA, Walker LA, Harman EA, Spiering BA, et al. Combined resistance and endurance training improves physical capacity and performance on tactical occupational tasks. European journal of applied physiology. 2010;109(6):1197-208. 38

Krustrup P, Hansen PR, Andersen LJ, Jakobsen MD, Sundstrup E, Randers MB, et al. Long-term musculoskeletal and cardiac health effects of recreational football and running for premenopausal women. Scandinavian journal of medicine & science in sports. 2010;20 Suppl 1:58-71. 40

Nindl BC, Alemany JA, Tuckow AP, Rarick KR, Staab JS, Kraemer WJ, et al. Circulating bioactive and immunoreactive IGF-I remain stable in women, despite physical fitness improvements after 8 weeks of resistance, aerobic, and combined exercise training. Journal of applied physiology. 2010;109(1):112-20. 44

Ozdemir RA, Celik O, Asci FH. Exercise interventions and their effects on physical self-perceptions of male university students. International journal of psychology : Journal international de psychologie. 2010;45(3):174-81. 47

Sedlock DA, Lee MG, Flynn MG, Park KS, Kamimori GH. Excess postexercise oxygen consumption after aerobic exercise training. International journal of sport nutrition and exercise metabolism. 2010;20(4):336-49. 49

Lee MG, Sedlock DA, Flynn MG, Kamimori GH. Resting metabolic rate after endurance exercise training. Medicine and science in sports and exercise. 2009;41(7):1444-51. 51

Lester ME, Urso ML, Evans RK, Pierce JR, Spiering BA, Maresh CM, et al. Influence of exercise mode and osteogenic index on bone biomarker responses during short-term physical training. Bone. 2009;45(4):768-76. 53

Brixius K, Schoenberger S, Ladage D, Knigge H, Falkowski G, Hellmich M, et al. Long-term endurance exercise decreases antiangiogenic endostatin signalling in overweight men aged 50-60 years. British journal of sports medicine. 2008;42(2):126-9. 55

Meyer T, Auracher M, Heeg K, Urhausen A, Kindermann W. Effectiveness of low-intensity endurance training. International journal of sports medicine. 2007;28(1):33-9. 57

Ring-Dimitriou S, von Duvillard SP, Paulweber B, Stadlmann M, Lemura LM, Peak K, et al. Nine months aerobic fitness induced changes on blood lipids and lipoproteins in untrained subjects versus controls. European journal of applied physiology. 2007;99(3):291-9. 59

Beneke R, Hutler M. The effect of training on running economy and performance in recreational athletes. Medicine and science in sports and exercise. 2005;37(10):1794-9. 61

Hautala AJ, Makikallio TH, Kiviniemi A, Laukkanen RT, Nissila S, Huikuri HV, et al. Heart rate dynamics after controlled training followed by a home-based exercise program. European journal of applied physiology. 2004;92(3):289-97. 63

Poehlman ET, Dvorak RV, DeNino WF, Brochu M, Ades PA. Effects of resistance training and endurance training on insulin sensitivity in nonobese, young women: a controlled randomised trial. The Journal of clinical endocrinology and metabolism. 2000;85(7):2463-8. 65

Bourque SP, Pate RR, Branch JD. Twelve weeks of endurance exercise training does not affect iron status measures in women. Journal of the American Dietetic Association. 1997;97(10):1116-21. 67

Hubinger L, Mackinnon LT. The effect of endurance training on lipoprotein(a) [Lp(a)] levels in middle-aged males. Medicine and science in sports and exercise. 1996;28(6):757-64. 69

Suter E, Marti B, Gutzwiller F. Jogging or walking--comparison of health effects. Annals of epidemiology. 1994;4(5):375-81. 71

Garber CE, McKinney JS, Carleton RA. Is aerobic dance an effective alternative to walk-jog exercise training? The Journal of sports medicine and physical fitness. 1992;32(2):136-41. 73

Suter E, Marti B. Little effect of long-term, self-monitored exercise on serum lipid levels in middle-aged women. The Journal of sports medicine and physical fitness. 1992;32(4):400-11. 75

Williams PT, Krauss RM, Vranizan KM, Albers JJ, Wood PD. Effects of weight-loss by exercise and by diet on apolipoproteins A-I and A-II and the particle-size distribution of high-density lipoproteins in men. Metabolism: clinical and experimental. 1992;41(4):441-9. 78

Oja P, Laukkanen RM, Kukkonen-Harjula TK, Vuori IM, Pasanen ME, Niittymaki SP, et al. Training effects of cross-country skiing and running on maximal aerobic cycle performance and on blood lipids. European journal of applied physiology and occupational physiology. 1991;62(6):400-4. 80

Marti B, Suter E, Riesen WF, Tschopp A, Wanner HU, Gutzwiller F. Effects of long-term, self-monitored exercise on the serum lipoprotein and apolipoprotein profile in middle-aged men. Atherosclerosis. 1990;81(1):19-31. 82

Suter E, Marti B, Tschopp A, Wanner HU, Wenk C, Gutzwiller F. Effects of self-monitored jogging on physical fitness, blood pressure and serum lipids: a controlled study in sedentary middle-aged men. International journal of sports medicine. 1990;11(6):425-32. 85

Williams PT, Albers JJ, Krauss RM, Wood PD. Associations of lecithin: cholesterol acyltransferase (LCAT) mass concentrations with exercise, weight loss, and plasma lipoprotein subfraction concentrations in men. Atherosclerosis. 1990;82(1-2):53-8. 87

Williams PT, Krauss RM, Vranizan KM, Wood PD. Changes in lipoprotein subfractions during diet-induced and exercise-induced weight loss in moderately overweight men. Circulation. 1990;81(4):1293-304. 89

Moses J, Steptoe A, Mathews A, Edwards S. The effects of exercise training on mental well-being in the normal population: a controlled trial. Journal of psychosomatic research. 1989;33(1):47-61. 92

Williams PT, Krauss RM, Vranizan KM, Albers JJ, Terry RB, Wood PD. Effects of exercise-induced weight loss on low density lipoprotein subfractions in healthy men. Arteriosclerosis. 1989;9(5):623-32. 94

Wood PD, Stefanick ML, Dreon DM, Frey-Hewitt B, Garay SC, Williams PT, et al. Changes in plasma lipids and lipoproteins in overweight men during weight loss through dieting as compared with exercise. The New England journal of medicine. 1988;319(18):1173-9. 97

Juneau M, Rogers F, De Santos V, Yee M, Evans A, Bohn A, et al. Effectiveness of self-monitored, home-based, moderate-intensity exercise training in middle-aged men and women. The American journal of cardiology. 1987;60(1):66-70. 100

Allen D, Freund BJ, Wilmore JH. Interaction of test protocol and horizontal run training on maximal oxygen uptake. Medicine and science in sports and exercise. 1986;18(5):581-7. 103

Gossard D, Haskell WL, Taylor CB, Mueller JK, Rogers F, Chandler M, et al. Effects of low- and high-intensity home-based exercise training on functional capacity in healthy middle-aged men. The American journal of cardiology. 1986;57(6):446-9. 105

Hagan RD, Upton SJ, Wong L, Whittam J. The effects of aerobic conditioning and/or caloric restriction in overweight men and women. Medicine and science in sports and exercise. 1986;18(1):87-94. 107

Mueller JK, Gossard D, Adams FR, Taylor CB, Haskell WL, Kraemer HC, et al. Assessment of prescribed increases in physical activity: application of a new method for microprocessor analysis of heart rate. The American journal of cardiology. 1986;57(6):441-5. 111

Savage MP, Petratis MM, Thomson WH, Berg K, Smith JL, Sady SP. Exercise training effects on serum lipids of prepubescent boys and adult men. Medicine and science in sports and exercise. 1986;18(2):197-204. 113

Thomas TR, Adeniran SB, Iltis PW, Aquiar CA, Albers JJ. Effects of interval and continuous running on HDL-cholesterol, apoproteins A-1 and B, and LCAT. Canadian journal of applied sport sciences Journal canadien des sciences appliquees au sport. 1985;10(1):52-9. 115

Iltis PW, Thomas TR, Adeniran SB, Aguiar CA, Albers JJ. Different running programs: plasma lipids, apoproteins, and lecithin: cholesterol acyltransferase in middle-aged men. Annals of Sports Medicine. 1984;2(1):16-22. 117

Mathur DN, Toriola AL. Twelve weeks jogging effects on selected cardiovascular risk factors in untrained healthy males. The Journal of sports medicine and physical fitness. 1984;24(3):259-62. 119

Thomas TR, Adeniran SB, Etheridge GL. Effects of different running programs on VO2 max, percent fat, and plasma lipids. Canadian journal of applied sport sciences Journal canadien des sciences appliquees au sport. 1984;9(2):55-62. 121

Toriola AL. Influence of 12-week jogging on body fat and serum lipids. British journal of sports medicine. 1984;18(1):13-7. 123

Williams PT, Wood PD, Krauss RM, Haskell WL, Vranizan KM, Blair SN, et al. Does weight loss cause the exercise-induced increase in plasma high density lipoproteins? Atherosclerosis. 1983;47(2):173-85. 125

Wood PD, Haskell WL, Blair SN, Williams PT, Krauss RM, Lindgren FT, et al. Increased exercise level and plasma lipoprotein concentrations: a one-year, randomised, controlled study in sedentary, middle-aged men. Metabolism: clinical and experimental. 1983;32(1):31-9. 127

Williams PT, Wood PD, Haskell WL, Vranizan K. The effects of running mileage and duration on plasma lipoprotein levels. JAMA : the journal of the American Medical Association. 1982;247(19):2674-9. 129

Wilmore JH, Davis JA, O'Brien RS, Vodak PA, Walder GR, Amsterdam EA. Physiological alterations consequent to 20-week conditioning programs of bicycling, tennis, and jogging. Medicine and science in sports and exercise. 1980;12(1):1-8. 131

Electronic Supplementary Material Appendix S3. Biomedical indices of health identified 135

Body composition outcome measures 136

Cardiorespiratory outcome measures 137

Blood serum concentration outcome measures 138

Electronic Supplementary Material Appendix S4. Forest plots and meta-analyses 139

Body composition outcome measures

Body mass

Overall and length of training subgroups 140

Gender subgroups 141

Lean body mass

Overall and length of training subgroups 142

Gender subgroups 143

Body fat

Overall and length of training subgroups 144

Gender subgroups 145

Body mass index (BMI)

Overall and length of training subgroups 146

Gender subgroups 147

Cardiorespiratory outcome measures

Resting heart rate

Overall and length of training subgroups 148

Gender subgroups 149

Maximal oxygen uptake (VO_2_max)

Overall and length of training subgroups 150

Gender subgroups 151

Blood serum concentration outcome measures

Triglycerides

Overall and length of training subgroups 152

Gender subgroups 153

Total cholesterol

Overall and length of training subgroups 154

Gender subgroups 155

High-density lipoprotein (HDL) cholesterol

Overall and length of training subgroups 156

Gender subgroups 157

Low-density lipoprotein (LDL) cholesterol

Overall and length of training subgroups 158

Gender subgroups 159

# Electronic Supplementary Material Appendix S1

**- Search strategy -**

Table of Contents

Description of PICOS: 7

Search Strategy for PubMed: 7

 Part A: Search for studies about running 7

 Part B: Search for studies about biomedical indices of health 7

 Part C: Generic search for randomised controlled trials and longitudinal studies 8

 Full search in PubMed (5734 records) 8

Search Strategy for EMBASE 9

 Part A: Search for studies about running 9

 Part B: Search for studies about biomedical indices of health 9

 Part C: Generic search for randomised controlled trials and longitudinal studies 10

 Full search in EMBASE (2650 records) 11

Search Strategy for Center on Health Sciences Information (CINAHL) [via EBSCO] 11

 Part A: Search for studies about running 11

 Part B: Search for studies about biomedical indices of health 11

 Part C: Generic search for randomised controlled trials and longitudinal studies 13

 Full search in CINAHL (7414 records) 13

Search Strategy for SPORTDiscus [via EBSCO] 13

 Part A: Search for studies about running 13

 Part B: Search for studies about biomedical indices of health 14

 Part C: Generic search for randomised controlled trials and longitudinal studies 15

 Full search in SPORTDiscus (5335 records) 16

Search Strategy for Physiotherapy Evidence Database (PEDro) 16

 Full search in PEDro 16

Search Strategy for The Cochrane Library 16

 Part A: Search for studies about running 16

 Part B: Search for studies about biomedical indices of health 16

 Full search in The Cochrane Library (1122 records) 17

Search Strategy for Latin American and Caribbean Center on Health Sciences Information (LILACS) 18

 Full LILACS search (52 records) 18

# Description of PICOS:

- Population (P): healthy and sedentary adults
- Intervention (I): endurance running
- Comparison (C): people who remain sedentary during the study
- Outcomes (O): biomedical indices of health
- Study design (S): randomised controlled trials (RCT)

# Search Strategy for PubMed:

## Part A: Search for studies about running

1. running/
2. jogging/
3. runner*
4. jogger*
5. marathon*
6. ultramarathon*
7. ultra-marathon*
8. ultra marathon*
9. OR/1-8

## Part B: Search for studies about biomedical indices of health

1. health/
2. health status/
3. health status indicators/
4. health promotion/
5. health records, personal/
6. health impact assessment/
7. health behaviour/
8. public health/
9. public health surveillance/
10. health benefit*
11. hemodynamics/
12. cardiovascular system/
13. cardiovascular diseases/
14. arterial pressure/
15. heart rate/
16. hypertension/
17. hypotension/
18. prehypertension/
19. cholesterol/
20. cholesterol, HDL/
21. cholesterol, LDL/
22. cholesterol, VLDL/
23. triglycerides/
24. diabetes
25. diabetes mellitus/
26. diabetes mellitus, type 1/
27. diabetes mellitus, type 2/
28. blood glucose/
29. insulin/
30. hyperglycemia/
31. hypoglycemia/
32. smoke/
33. alcohol drinking/
34. alcoholism/
35. diet/
36. diet records/
37. diet surveys/
38. drug therapy/
39. medicat*
40. body mass index/
41. weight loss/
42. overweigh/
43. obesity/
44. metabolic syndrome X/
45. abdominal fat/
46. dyslipidemias/
47. waist circumference/
48. waist-hip ratio/
49. hip circumference
50. chest circumference
51. body weights and measures/
52. physical fitness/
53. anaerobic threshold/
54. oxygen consumption/
55. oxygen intake
56. exercise test/
57. mental health/
58. psychology/
59. psychology, social/
60. behavior/
61. health behavior/
62. behavioral medicine/
63. behavioral sciences/
64. quality of life/
65. disability evaluation/
66. disabilit*
67. impairment*
68. handicap*
69. morbidity/
70. comorbidity/
71. longevity/
72. mortality/
73. death/
74. cause of death/
75. OR/10-83

## Part C: Generic search for randomised controlled trials and longitudinal studies

1. randomised controlled trial/
2. randomised controlled trial as topic/
3. randomised
4. controlled clinical trial as topic/
5. placebo
6. placebo effect/
7. randomly
8. trial
9. groups
10. cohort studies/
11. cohort*
12. epidemiologic studies/
13. epidemiologic methods/
14. longitudinal studies/
15. follow up studies/
16. OR/85-99

## Full search in PubMed (5734 records)

1. animal*
2. 9 AND 84 AND 100 NOT 101

# Search Strategy for EMBASE

## Part A: Search for studies about running

1. running/exp
2. marathon runner/exp
3. jogging/exp
4. treadmill exercise/exp
5. runner*
6. run*
7. jog*
8. marathon*
9. ultramarathon*
10. OR/1-9

## Part B: Search for studies about biomedical indices of health

- 1. health/exp
  2. health status/exp
  3. health status indicator/exp
  4. health behavior/exp
  5. health survey/exp
  6. health hazard/exp
  7. health impact assessment/exp
  8. health promotion/exp
  9. health sciences/exp
  10. public health/exp
  11. attitude to health/exp
  12. mental health/exp
  13. framingham risk score/exp
  14. cardiovascular effect/exp
  15. cardiovascular system/exp
  16. cardiovascular system examination/exp
  17. cardiovascular disease/exp
  18. atherosclerotic cardiovascular disease/exp
  19. cardiovascular parameters/exp
  20. cardiovascular function/exp
  21. cardiovascular response/exp
  22. cardiovascular risk/exp
  23. hypertension/exp
  24. hypotension/exp
  25. hemodynamic/exp
  26. arterial pressure/exp
  27. pulse pressure/exp
  28. pulse rate/exp
  29. heart rate/exp
  30. basal metabolic rate/exp
  31. prehypertension/exp
  32. cholesterol/exp
  33. high density lipoprotein/exp
  34. low density lipoprotein/exp
  35. very low density lipoprotein/exp
  36. triacylglycerol/exp
  37. diabetes mellitus/exp
  38. insulin dependent diabetes mellitus/exp
  39. non insulin dependent diabetes mellitus/exp
  40. glucose blood level/exp
  41. insulin/exp
  42. hyperglicemia/exp
  43. hypoglicemia/exp
  44. smoke/exp
  45. cigarette smoke/exp
  46. tobacco smoke/exp
  47. alcohol/exp
  48. alcohol consumption/exp
  49. drinking behavior/exp
  50. alcoholism/exp
  51. diet/exp
  52. fat intake/exp
  53. medical records/exp
  54. drug therapy/exp
  55. medicat*
  56. body mass/exp
  57. weight reduction/exp
  58. weight control/exp
  59. overweight/exp
  60. obesity/exp
  61. metabolic syndrome X/exp
  62. dyslipidemia/exp
  63. abdominal fat/exp
  64. waist hip ratio/exp
  65. waist circumference/exp
  66. hip circumference/exp
  67. chest circumference/exp
  68. fitness/exp
  69. oxygen consumption/exp
  70. anaerobic threshold/exp
  71. psychology/exp
  72. psychological test/exp
  73. sports psychology/exp
  74. motivation/exp
  75. behavior/exp
  76. behavior science/exp
  77. quality of life/exp
  78. quality of life index/exp
  79. disability/exp
  80. handicap*
  81. impairment*
  82. physical disability/exp
  83. motor dysfunction/exp
  84. morbidity/exp
  85. comorbidity/exp
  86. longevity/exp
  87. mortality/exp
  88. death/exp
  89. cause of death/exp
  90. OR/11-99

## Part C: Generic search for randomised controlled trials and longitudinal studies

- 1. clinical article/exp
  2. clinical study/exp
  3. clinical trial/exp
  4. controlled study/exp
  5. controlled clinical trial/exp
  6. randomised controlled trial/exp
  7. major clinical study/exp
  8. double blind procedure/exp
  9. multicenter study/exp
  10. single blind procedure/exp
  11. phase 3 clinical trial/exp
  12. phase 4 clinical trial/exp
  13. crossover procedure/exp
  14. placebo/exp
  15. placebo effect/exp
  16. OR/101-115
  17. allocat*
  18. assign*
  19. blind*
  20. clinic* AND (study/exp or trial)
  21. compar*
  22. control*
  23. crossover
  24. factorial*
  25. follow up/exp
  26. placebo*
  27. prospectiv*
  28. random*
  29. (singl* OR doubl* OR trebl* OR tripl*) AND (blind* OR mask*)
  30. trial
  31. versus OR vs
  32. OR/117-131
  33. cohort analysis/exp
  34. longitudinal study/exp
  35. prospective study/exp
  36. observational study/exp
  37. cohort*
  38. OR/133-137
  39. 116 AND 132
  40. 138 OR 139

## Full search in EMBASE (2650 records)

- - 1. animal/exp
    2. nonhuman/exp
    3. animal experiment/exp
    4. OR/141-143
    5. 10 AND 100 AND 140 NOT 144

# Search Strategy for Center on Health Sciences Information (CINAHL) [via EBSCO]

## Part A: Search for studies about running

1. running/exp
2. running, distance/
3. jogging/
4. run* [apply related words]
5. jog* [apply related words]
6. marathon* [apply related words]
7. ultramarathon* [apply related words]
8. ultra-marathon* [apply related words]
9. ultra marathon* [apply related words]
10. treadmill run*
11. treadmill exercise
12. OR/1-11

## Part B: Search for studies about biomedical indices of health

1. health/exp
2. health benefit* [apply related words]
3. health status/exp
4. health status indicators/exp
5. health promotion/exp
6. risk assessment/
7. health behavior/exp
8. public health/exp
9. population surveillance/exp
10. hemodynamics/exp
11. cardiovascular system/exp
12. cardiovascular diseases/exp
13. cardiovascular risk factors/
14. arterial pressure/exp
15. blood pressure/exp
16. heart rate/exp
17. heart rate variability/
18. hypertension/exp
19. hypotension/exp
20. prehypertension/
21. cholesterol/exp
22. lipoproteins, HDL/exp
23. lipoproteins, LDL/exp
24. triglycerides/
25. diabetes mellitus/exp
26. diabetes mellitus, type 1/exp
27. diabetes mellitus, type 2/
28. blood glucose/
29. insulin/exp
30. hyperglycemia/exp
31. hypoglycemia/exp
32. smoke/exp
33. ethanol/exp
34. alcohol intake [apply related words]
35. alcohol consumption [apply related words]
36. alcoholism/
37. diet/exp
38. diet records/
39. diet, reducing/
40. food intake/exp
41. dietary fats/exp
42. body mass index/
43. fat free mass/
44. overweigh [apply related words]
45. obesity/exp
46. attitude to obesity/
47. metabolic syndrome X/exp
48. weight loss/exp
49. weight control/
50. body weights and measeures/exp
51. medical records/exp
52. abdominal fat/
53. hyperlipidemia/exp
54. drug therapy/exp
55. medicat* [apply related words]
56. physical fitness/exp
57. anaerobic threshold/
58. aerobic exercise/exp
59. oxygen consumption/exp
60. oxygen intake [apply related words]
61. mental health/
62. psychology/exp
63. psychological tests/exp
64. psychology, sports/
65. psychology, social/exp
66. behavior/exp
67. behavior changes/
68. behavior modification/exp
69. behavior rating scales/
70. social hehavior/exp
71. behavioral sciences/exp
72. behavior research/
73. quality of life/exp
74. disability evaluation/exp
75. disabilit* [apply related words]
76. impairment* [apply related words]
77. handicap* [apply related words]
78. damage [apply related words]
79. morbidity/exp
80. comorbidity/
81. longevity/
82. mortality/exp
83. death/exp
84. cause of death/
85. OR/13-96

## Part C: Generic search for randomised controlled trials and longitudinal studies

- 1. clinical trials/exp
  2. randomised controlled trials/
  3. clinical w3 trial
  4. double-blind studies/
  5. single-blind studies/
  6. triple-blind studies/
  7. placebo effect/
  8. placebos/
  9. random* [apply related words]
  10. random sample/exp
  11. study design/exp
  12. comparative studies/
  13. evaluation research/exp
  14. prospective studies/exp
  15. postexposure follow-up/
  16. follow-up stud* [apply related words]
  17. follow up stud* [apply related words]
  18. followup stud* [apply related words]
  19. control*
  20. prospectiv* [apply related words]
  21. volunteer*
  22. cohort* [apply related words]
  23. cohort analysis
  24. concurrent cohort studies/
  25. longitudinal stud* [apply related words]
  26. OR/98-122

## Full search in CINAHL (7414 records)

1. animals/exp
2. 12 AND 97 AND 123 NOT 124 [limit: academic journals]

# Search Strategy for SPORTDiscus [via EBSCO]

## Part A: Search for studies about running

1. RUNNING/exp
2. LONG-distance running/exp
3. MINIMALIST running/
4. RUNNING – Starting/
5. MARATHON running/exp
6. ROAD running/
7. ULTRAMARATHON running/exp
8. 10k race/
9. 5k race/
10. RUNNING for people with disabilities/
11. RUNNING races/exp
12. COLLEGE cross-country running/
13. MIDDLE distance running/
14. RUNNING – Training/exp
15. BAREFOOT running/
16. CROSS-country running/exp
17. CROSS-country running competitions/
18. RUNNERS (Sports)/exp
19. RUNNING speed/
20. run* [apply related words]
21. jog* [apply related words]
22. OR/1-21

## Part B: Search for studies about biomedical indices of health

1. HEALTH/exp
2. health benefit* [apply related words]
3. HEALTH status indicators/exp
4. HEALTH risk assessment/exp
5. HEALTH promotion/
6. HEALTH attitudes/
7. HEALTH behaviour/exp
8. HEALTH surveys/exp
9. PUBLIC health/exp
10. HEMODYNAMICS/exp
11. BLOOD – circulation/exp
12. BLOOD – circulation disorders/exp
13. CARDIOVASCULAR system/exp
14. CARDIOVASCULAR system - diseases/exp
15. CARDIOVASCULAR system - diseases - risk factors/
16. CARDIOVASCULAR fitness/
17. CARDIOVASCULAR agents/exp
18. arterial pressure [apply related words]
19. VASCULAR resistance/
20. BLOOD pressure/
21. HEART beat/exp
22. HEART rate monitoring/
23. HYPERTENSION/exp
24. HYPOTENSION/
25. HYPOTENSION agents/exp
26. ARTERIOSLEROSIS/exp
27. CHOLESTEROL/exp
28. CHOLESTEROL in the body/
29. HYPERCHOLESTEREMIA/
30. BLOOD lipoproteins/exp
31. HIGH density lipoproteins/
32. LOW density lipoproteins/
33. TRIGLYCERIDES/
34. DIABETES/exp
35. DIABETS athletes/
36. NON-insulin-dependent diabetes/
37. INSULIN-dependent diabetes/
38. BLOOD sugar/exp
39. GLUCOSE intolerance/
40. INSULIN/exp
41. HYPERGLYCEMIA/
42. HYPOGLYCEMIA/
43. SMOKING/exp
44. CIGARETTES/
45. ALCOHOL/
46. BLOOD alcohol/
47. DRINKING of alcohol beverages/
48. alcohol intake [apply related words]
49. ALCOHOLISM/exp
50. ALCOHOLISM in sports/
51. DIET/exp
52. FOOD consumption/
53. BODY mass index/exp
54. OVERWEIGH person/exp
55. OBESITY/exp
56. METABOLISM – Disorders/exp
57. METABOLIC syndrome/
58. WEIGHT loss/exp
59. BODY weight/exp
60. BODY weight – regulation/
61. MEDICAL records/
62. FAT/exp
63. abdominal fat [apply related words]
64. HYPERLIPIDEMIA/exp
65. CHEMOTHERAPY/exp
66. medicat* [apply related words]
67. AEROBIC capacity/
68. PHYSICAL fitness/exp
69. PHYSICAL fitness testing/exp
70. ANAEROBIC threshold/
71. ANAEROBIC capacity/exp
72. OXYGEN consumption (Physiology)/exp
73. MENTAL health/exp
74. PSYCHOLOGY/exp
75. PSYCHOLOGICAL tests/exp
76. SPORTS – Psychological aspects/exp
77. SOCIAL psychology/exp
78. ATTITUDE (Psychology)/exp
79. SEDENTARY behavior/
80. SEDENTARY people/exp
81. QUALITY of life/exp
82. PEOPLE with disability/exp
83. DISABILITIES/exp
84. impairment* [apply related words]
85. physical damage [apply related words]
86. handicap* [apply related words]
87. LONGEVITY/exp
88. DISEASES/exp
89. MORTALITY/exp
90. DEATH/exp
91. OR/23-112

## Part C: Generic search for randomised controlled trials and longitudinal studies

1. clinical trial* [apply related words]
2. controlled trial* [apply related words]
3. randomi?ed controlled trial* [apply related words]
4. trial* [apply related words]
5. double blind stud* [apply related words]
6. single blind stud* [apply related words]
7. triple blind stud* [apply related words]
8. placebo* [apply related words]
9. PLACEBOS (medicine)/
10. random* [apply related words]
11. comparative stud* [apply related words]
12. prospective stud* [apply related words]
13. prospectiv* [apply related words]
14. exposure [apply related words]
15. follow up [apply related words]
16. follow-up [apply related words]
17. control* [apply related words]
18. COHORT analysis/
19. cohort stud* [apply related words]
20. cohort* [apply related words]
21. longitudinal [apply related words]
22. longitudinal stud* [apply related words]
23. observational stud* [apply related words]
24. experiment* [apply related words]
25. intervention* [apply related words]
26. OR/114-138

## Full search in SPORTDiscus (5335 records)

- 1. animal* [apply related words]
  2. 22 AND 113 AND 139 NOT 140 [limit: peer reviewed and academic journals]

# Search Strategy for Physiotherapy Evidence Database (PEDro)

## Full search in PEDro

1. runn*
2. health*
3. 1 AND 2 [limit: clinical trials] (46)

# Search Strategy for The Cochrane Library

## Part A: Search for studies about running

1. running/
2. jogging/
3. runner*
4. jogger*
5. marathon*
6. ultramarathon*
7. ultra-marathon*
8. ultra marathon*
9. OR/1-8

## Part B: Search for studies about biomedical indices of health

1. health/exp
2. health status/exp
3. health status indicators/exp
4. health promotion/exp
5. health records, personal/exp
6. health behaviour/exp
7. public health/exp
8. public health surveillance/exp
9. health benefit*
10. hemodynamics/exp
11. cardiovascular system/exp
12. cardiovascular diseases/exp
13. arterial pressure/exp
14. heart rate/exp
15. hypertension/exp
16. hypotension/exp
17. prehypertension/exp
18. cholesterol/exp
19. cholesterol, HDL/exp
20. cholesterol, LDL/exp
21. cholesterol, VLDL/exp
22. triglycerides/exp
23. diabetes
24. diabetes mellitus/exp
25. diabetes mellitus, type 1/exp
26. diabetes mellitus, type 2/exp
27. blood glucose/exp
28. insulin/exp
29. hyperglycemia/exp
30. hypoglycemia/exp
31. smoke/exp
32. smoking/exp
33. alcohol drinking/exp
34. alcoholism/exp
35. diet/exp
36. diet records/exp
37. diet surveys/exp
38. drug therapy/exp
39. medicat*
40. body mass index/exp
41. weight loss/exp
42. overweigh/exp
43. obesity/exp
44. metabolic syndrome X/exp
45. abdominal fat/exp
46. dyslipidemias/exp
47. waist circumference/exp
48. waist-hip ratio/exp
49. hip circumference
50. chest circumference
51. body weights and measures/exp
52. physical fitness/exp
53. anaerobic threshold/exp
54. oxygen consumption/exp
55. oxygen intake
56. exercise test/exp
57. mental health/exp
58. psychology/exp
59. psychology, social/exp
60. behavior/exp
61. health behavior/exp
62. behavioral medicine/exp
63. behavioral sciences/exp
64. quality of life/exp
65. disability evaluation/exp
66. disabilit*
67. impairment
68. handicap
69. morbidity/exp
70. comorbidity/exp
71. longevity/exp
72. mortality/exp
73. death/exp
74. cause of death/exp
75. OR/10-83

## Full search in The Cochrane Library (1122 records)

1. 9 AND 84 [limits: trials, methods studies, technology assessments, economic evaluations and Cochrane groups]

# Search Strategy for Latin American and Caribbean Center on Health Sciences Information (LILACS)

## Full LILACS search (52 records)

( ( ( ( ( ( ( ( ( ( ( "RUNNING" ) or "JOGGING" ) or "RUNNER" ) or "RUNNERS" ) or "MARATHON" ) or "MARATHONERS" ) or "MARATHONS" ) or "ULTRAMARATHON" ) or "ULTRAMARATHONS" ) or "5KM" ) or "10KM" ) or "15KM" [Words] and ( ( ( ( ( ( ( ( ( ( ( ( ( ( ( ( ( ( ( ( ( ( ( ( ( ( ( ( ( ( ( ( ( ( ( ( ( ( ( ( ( ( ( ( ( ( ( ( ( ( ( ( ( ( ( ( ( ( ( ( ( ( ( ( ( ( ( ( ( ( ( ( ( ( ( ( ( ( ( ( ( ( ( "HEALTH" ) or "BENEFIT" ) or "BENEFIT’S" ) or "BENEFIT-RISK" ) or "BENEFIT-RISK ASSESSMENT" ) or "BENEFIT-HARM" ) or "BENEFITINDICATOR" ) or "BENEFITIAL" ) or "CARDIOVASCULAR" ) or "CARDIOVASCULAR ABNORMALITIES" ) or "CARDIOVASCULAR AGENTS" ) or "CARDIOVASCULAR DISEASES" ) or "RISK" ) or "RISK FACTORS" ) or "ARTERIAL-HYPERTENSION" ) or "PRESSURE" ) or "HEART RATE" ) or "HEART RATE CONTROL" ) or "HYPERTENSION" ) or "HYPOTENSION" ) or "CHOLESTEROL" ) or "HDL" ) or "LDL" ) or "VLDL" ) or "TRIGLYCERIDES" ) or "DIABETES" ) or "DIABETES MELLITUS" ) or "HYPERGLICEMIA" ) or "HYPOGLYCEMIA" ) or "HEMODYNAMICS" ) or "BLOOD GLUCOSE" ) or "INSULIN" ) or "SMOKE" ) or "SMOKING" ) or "ALCOHOL" ) or "ALCOHOL DRINKING" ) or "ALCOHOLISM" ) or "DIET" ) or "FOOD CONSUMPTION" ) or "DIET SURVEYS" ) or "DIET THERAPY" ) or "DRUG THERAPY" ) or "MEDICATION" ) or "OVERWEIGHT" ) or "OBESITY" ) or "FAT" ) or "ABDOMINAL FAT" ) or "WEIGHT LOSS" ) or "WEIGHT REDUCTION" ) or "DYSLIPIDEMIAS" ) or "METABOLIC SYNDROME X" ) or "BODY MASS INDEX" ) or "BMI" ) or "CIRCUMFERENCES" ) or "BODY COMPOSITION" ) or "WAIST CIRCUMFERENCE" ) or "WAIST-HIP" ) or "WAIST-HIP RATIO" ) or "CIRCUMFERENCE-HIP" ) or "AEROBIC EXERCISE" ) or "ANAEROBIC THRESHOLD" ) or "PHYSICAL FITNESS" ) or "OXYGEN CONSUMPTION" ) or "VO2MAX" ) or "PSYCHOLOGY" ) or "MENTAL HEALTH" ) or "BEHAVIOR" ) or "BEHAVIOR CONTROL" ) or "BEHAVIOR MODIFICATION" ) or "BEHAVIORAL" ) or "QUALITY OF LIFE" ) or "DISABILITY" ) or "DISABILITY EVALUATION" ) or "IMPAIRMENT" ) or "IMPAIRMENT/DISABILITY" ) or "HANDICAP" ) or "MORBIDITY" ) or "MORBIDITY SURVEYS" ) or "COMORBIDITY" ) or "LONGEVITY" ) or "MORTALITY" ) or "MORTALITY RATE" ) or "DEATH" ) or "CAUSE OF DEATH" [Words] and ( ( ( ( ( ( ( ( ( ( ( ( ( ( ( ( ( ( ( ( ( ( ( ( ( ( ( ( ( ( ( ( ( ( ( ( ( ( ( ( ( ( ( ( ( ( "CLINICAL TRIALS AS TOPIC" ) or "CLINICAL TRIAL OVERVIEWS" ) or "RANDOMISED CONTROLLED TRIALS AS TOPIC" ) or "RANDOM" ) or "RANDOM ALLOCATION" ) or "RANDOM AND SYSTEMATIC SAMPLING" ) or "TRIAL" ) or "SINGLE-BLIND" ) or "DOUBLE-BLIND" ) or "TRIPLE-BLIND" ) or "SINGLE-BLIND METHOD" ) or "SINGLE-BLINDED" ) or "DOUBLE-BLIND METHOD" ) or "DOUBLE-BLINDED" ) or "TRIPLE-BLINDED" ) or "TRIPLE-CROSSOVER" ) or "CROSSOVER" ) or "PLACEBO" ) or "PLACEBO EFFECT" ) or "PLACEBO-CONTROL" ) or "PLACEBO-COMPARED" ) or "PLACEBOS" ) or "COMPARATIVE-PROSPECTIVE" ) or "COMPARATIVESTUDY" ) or "PROSPECTIVE-CLINICAL" ) or "PROSPECTIVE-COMPARATIVE" ) or "PROSPECTIVE-CONTROLLED" ) or "PROSPECTIVE-DESCRIPTIVE" ) or "PROSPECTIVE-LONGITUDINAL" ) or "PROSPECTIVE-OBSERVATIONAL" ) or "PROSPECTIVE-RANDOMISED" ) or "LONGITUDINAL" ) or "LONGITUDINAL-PROSPECTIVE" ) or "COHORT" ) or "COHORT ANALYSIS" ) or "COHORT EFFECT" ) or "COHORT STUDIES" ) or "COHORT-DESIGNED" ) or "FOLLOW-UP " ) or "FOLLOW-UP STUDIES" ) or "EXPOSURE" ) or "EXPOSURE TIME" ) or "OBSERVATIONAL STUDIES" ) or "OBSERVATIONAL" ) or "EXPERIMENT" ) or "INTERVENTION" ) or "INTERVENTION STUDIES" [Words]

# Electronic Supplementary Material Appendix S2

**- Study characteristics -**

Table of Contents

Moghadasi M, Mohammadi Domieh A. Effects of Resistance versus Endurance Training on Plasma Lipocalin-2 in Young Men. Asian Journal of Sports Medicine. 2014;4(6):108-14. 22

Celik O, Salci Y, Ak E, Kalaci A, Korkusuz F. Serum cartilage oligomeric matrix protein accumulation decreases significantly after 12 weeks of running but not swimming and cycling training - a randomised controlled trial. The Knee. 2013;20(1):19-25. 24

Gregory SM, Spiering BA, Alemany JA, Tuckow AP, Rarick KR, Staab JS, et al. Exercise-induced insulin-like growth factor I system concentrations after training in women. Medicine and science in sports and exercise. 2013;45(3):420-8. 26

Asad M, Ravasi AA, Faramarzi M, Pournemati P. The effects of three training methods endurance, resistance and concurrent on adiponectin resting levels in overweighed untrained men. Bratislavske lekarske listy. 2012;113(11):664-8. 28

Hosseini M, Piri M, Agha-Alinejad H, Haj-Sadeghi S. The effect of endurance, resistance and concurrent training on the heart structure of female students. Biology of Sport. 2012;29(1):17-21. 30

Lo MS, Lin LL, Yao WJ, Ma MC. Training and detraining effects of the resistance vs. endurance program on body composition, body size, and physical performance in young men. Journal of strength and conditioning research / National Strength & Conditioning Association. 2011;25(8):2246-54. 32

Andersen LJ, Hansen PR, Sogaard P, Madsen JK, Bech J, Krustrup P. Improvement of systolic and diastolic heart function after physical training in sedentary women. Scandinavian journal of medicine & science in sports. 2010;20 Suppl 1:50-7. 35

Hendrickson NR, Sharp MA, Alemany JA, Walker LA, Harman EA, Spiering BA, et al. Combined resistance and endurance training improves physical capacity and performance on tactical occupational tasks. European journal of applied physiology. 2010;109(6):1197-208. 38

Krustrup P, Hansen PR, Andersen LJ, Jakobsen MD, Sundstrup E, Randers MB, et al. Long-term musculoskeletal and cardiac health effects of recreational football and running for premenopausal women. Scandinavian journal of medicine & science in sports. 2010;20 Suppl 1:58-71. 40

Nindl BC, Alemany JA, Tuckow AP, Rarick KR, Staab JS, Kraemer WJ, et al. Circulating bioactive and immunoreactive IGF-I remain stable in women, despite physical fitness improvements after 8 weeks of resistance, aerobic, and combined exercise training. Journal of applied physiology. 2010;109(1):112-20. 44

Ozdemir RA, Celik O, Asci FH. Exercise interventions and their effects on physical self-perceptions of male university students. International journal of psychology : Journal international de psychologie. 2010;45(3):174-81. 47

Sedlock DA, Lee MG, Flynn MG, Park KS, Kamimori GH. Excess postexercise oxygen consumption after aerobic exercise training. International journal of sport nutrition and exercise metabolism. 2010;20(4):336-49. 49

Lee MG, Sedlock DA, Flynn MG, Kamimori GH. Resting metabolic rate after endurance exercise training. Medicine and science in sports and exercise. 2009;41(7):1444-51. 51

Lester ME, Urso ML, Evans RK, Pierce JR, Spiering BA, Maresh CM, et al. Influence of exercise mode and osteogenic index on bone biomarker responses during short-term physical training. Bone. 2009;45(4):768-76. 53

Brixius K, Schoenberger S, Ladage D, Knigge H, Falkowski G, Hellmich M, et al. Long-term endurance exercise decreases antiangiogenic endostatin signalling in overweight men aged 50-60 years. British journal of sports medicine. 2008;42(2):126-9. 55

Meyer T, Auracher M, Heeg K, Urhausen A, Kindermann W. Effectiveness of low-intensity endurance training. International journal of sports medicine. 2007;28(1):33-9. 57

Ring-Dimitriou S, von Duvillard SP, Paulweber B, Stadlmann M, Lemura LM, Peak K, et al. Nine months aerobic fitness induced changes on blood lipids and lipoproteins in untrained subjects versus controls. European journal of applied physiology. 2007;99(3):291-9. 59

Beneke R, Hutler M. The effect of training on running economy and performance in recreational athletes. Medicine and science in sports and exercise. 2005;37(10):1794-9. 61

Hautala AJ, Makikallio TH, Kiviniemi A, Laukkanen RT, Nissila S, Huikuri HV, et al. Heart rate dynamics after controlled training followed by a home-based exercise program. European journal of applied physiology. 2004;92(3):289-97. 63

Poehlman ET, Dvorak RV, DeNino WF, Brochu M, Ades PA. Effects of resistance training and endurance training on insulin sensitivity in nonobese, young women: a controlled randomised trial. The Journal of clinical endocrinology and metabolism. 2000;85(7):2463-8. 65

Bourque SP, Pate RR, Branch JD. Twelve weeks of endurance exercise training does not affect iron status measures in women. Journal of the American Dietetic Association. 1997;97(10):1116-21. 67

Hubinger L, Mackinnon LT. The effect of endurance training on lipoprotein(a) [Lp(a)] levels in middle-aged males. Medicine and science in sports and exercise. 1996;28(6):757-64. 69

Suter E, Marti B, Gutzwiller F. Jogging or walking--comparison of health effects. Annals of epidemiology. 1994;4(5):375-81. 71

Garber CE, McKinney JS, Carleton RA. Is aerobic dance an effective alternative to walk-jog exercise training? The Journal of sports medicine and physical fitness. 1992;32(2):136-41. 73

Suter E, Marti B. Little effect of long-term, self-monitored exercise on serum lipid levels in middle-aged women. The Journal of sports medicine and physical fitness. 1992;32(4):400-11. 75

Williams PT, Krauss RM, Vranizan KM, Albers JJ, Wood PD. Effects of weight-loss by exercise and by diet on apolipoproteins A-I and A-II and the particle-size distribution of high-density lipoproteins in men. Metabolism: clinical and experimental. 1992;41(4):441-9. 78

Oja P, Laukkanen RM, Kukkonen-Harjula TK, Vuori IM, Pasanen ME, Niittymaki SP, et al. Training effects of cross-country skiing and running on maximal aerobic cycle performance and on blood lipids. European journal of applied physiology and occupational physiology. 1991;62(6):400-4. 80

Marti B, Suter E, Riesen WF, Tschopp A, Wanner HU, Gutzwiller F. Effects of long-term, self-monitored exercise on the serum lipoprotein and apolipoprotein profile in middle-aged men. Atherosclerosis. 1990;81(1):19-31. 82

Suter E, Marti B, Tschopp A, Wanner HU, Wenk C, Gutzwiller F. Effects of self-monitored jogging on physical fitness, blood pressure and serum lipids: a controlled study in sedentary middle-aged men. International journal of sports medicine. 1990;11(6):425-32. 85

Williams PT, Albers JJ, Krauss RM, Wood PD. Associations of lecithin: cholesterol acyltransferase (LCAT) mass concentrations with exercise, weight loss, and plasma lipoprotein subfraction concentrations in men. Atherosclerosis. 1990;82(1-2):53-8. 87

Williams PT, Krauss RM, Vranizan KM, Wood PD. Changes in lipoprotein subfractions during diet-induced and exercise-induced weight loss in moderately overweight men. Circulation. 1990;81(4):1293-304. 89

Moses J, Steptoe A, Mathews A, Edwards S. The effects of exercise training on mental well-being in the normal population: a controlled trial. Journal of psychosomatic research. 1989;33(1):47-61. 92

Williams PT, Krauss RM, Vranizan KM, Albers JJ, Terry RB, Wood PD. Effects of exercise-induced weight loss on low density lipoprotein subfractions in healthy men. Arteriosclerosis. 1989;9(5):623-32. 94

Wood PD, Stefanick ML, Dreon DM, Frey-Hewitt B, Garay SC, Williams PT, et al. Changes in plasma lipids and lipoproteins in overweight men during weight loss through dieting as compared with exercise. The New England journal of medicine. 1988;319(18):1173-9. 97

Juneau M, Rogers F, De Santos V, Yee M, Evans A, Bohn A, et al. Effectiveness of self-monitored, home-based, moderate-intensity exercise training in middle-aged men and women. The American journal of cardiology. 1987;60(1):66-70. 100

Allen D, Freund BJ, Wilmore JH. Interaction of test protocol and horizontal run training on maximal oxygen uptake. Medicine and science in sports and exercise. 1986;18(5):581-7. 103

Gossard D, Haskell WL, Taylor CB, Mueller JK, Rogers F, Chandler M, et al. Effects of low- and high-intensity home-based exercise training on functional capacity in healthy middle-aged men. The American journal of cardiology. 1986;57(6):446-9. 105

Hagan RD, Upton SJ, Wong L, Whittam J. The effects of aerobic conditioning and/or caloric restriction in overweight men and women. Medicine and science in sports and exercise. 1986;18(1):87-94. 107

Mueller JK, Gossard D, Adams FR, Taylor CB, Haskell WL, Kraemer HC, et al. Assessment of prescribed increases in physical activity: application of a new method for microprocessor analysis of heart rate. The American journal of cardiology. 1986;57(6):441-5. 111

Savage MP, Petratis MM, Thomson WH, Berg K, Smith JL, Sady SP. Exercise training effects on serum lipids of prepubescent boys and adult men. Medicine and science in sports and exercise. 1986;18(2):197-204. 113

Thomas TR, Adeniran SB, Iltis PW, Aquiar CA, Albers JJ. Effects of interval and continuous running on HDL-cholesterol, apoproteins A-1 and B, and LCAT. Canadian journal of applied sport sciences Journal canadien des sciences appliquees au sport. 1985;10(1):52-9. 115

Iltis PW, Thomas TR, Adeniran SB, Aguiar CA, Albers JJ. Different running programs: plasma lipids, apoproteins, and lecithin: cholesterol acyltransferase in middle-aged men. Annals of Sports Medicine. 1984;2(1):16-22. 117

Mathur DN, Toriola AL. Twelve weeks jogging effects on selected cardiovascular risk factors in untrained healthy males. The Journal of sports medicine and physical fitness. 1984;24(3):259-62. 119

Thomas TR, Adeniran SB, Etheridge GL. Effects of different running programs on VO2 max, percent fat, and plasma lipids. Canadian journal of applied sport sciences Journal canadien des sciences appliquees au sport. 1984;9(2):55-62. 121

Toriola AL. Influence of 12-week jogging on body fat and serum lipids. British journal of sports medicine. 1984;18(1):13-7. 123

Williams PT, Wood PD, Krauss RM, Haskell WL, Vranizan KM, Blair SN, et al. Does weight loss cause the exercise-induced increase in plasma high density lipoproteins? Atherosclerosis. 1983;47(2):173-85. 125

Wood PD, Haskell WL, Blair SN, Williams PT, Krauss RM, Lindgren FT, et al. Increased exercise level and plasma lipoprotein concentrations: a one-year, randomised, controlled study in sedentary, middle-aged men. Metabolism: clinical and experimental. 1983;32(1):31-9. 127

Williams PT, Wood PD, Haskell WL, Vranizan K. The effects of running mileage and duration on plasma lipoprotein levels. JAMA : the journal of the American Medical Association. 1982;247(19):2674-9. 129

Wilmore JH, Davis JA, O'Brien RS, Vodak PA, Walder GR, Amsterdam EA. Physiological alterations consequent to 20-week conditioning programs of bicycling, tennis, and jogging. Medicine and science in sports and exercise. 1980;12(1):1-8. 131

**Reference**

| Moghadasi M, Mohammadi Domieh A. Effects of Resistance versus Endurance Training on Plasma Lipocalin-2 in Young Men. Asian Journal of Sports Medicine. 2014;4(6):108-14. |
| --- |

**Objectives**

| To determine and compare the effects of resistance training and endurance training on body composition, insulin resistance, C-reactive protein and lipocalin-2 concentration in healthy and sedentary young men. |
| --- |

**Description of participants included in the study**

| - Participants randomised: n=30 sedentary young men, mean age of 25.3 (SD 2.3).  - Participants included in the analysis, n=29:   - Endurance (running) group: n=10. - Resistance group: n=9. - Control group: n=10. |
| --- |

**Follow-up period and time-point assessments after baseline**

| - Follow-up: 8 weeks.  - Time-points: baseline and 8 weeks after baseline. |
| --- |

**Intervention (running program)**

| Endurance (running) group  - Duration: 8 weeks.  - Frequency: 3 times/week.  - Description: running at 65–80% of maximal heart rate for 20–34 min per day. Each training session was started with a warm-up and finished with a cool down. The program started with 20 min running for the first few sessions, and this was then changed to 34 min per session until the end of training. |
| --- |

**Comparison group**

| Resistance group  - Duration: 8 weeks.  - Frequency: 3 times/week.  - Description: 50-60 min of circuit weight training with 8 resistance exercises selected to stress the major muscle groups in the following order: chest press, leg extension, shoulder press, leg curls, latissimus pull down, leg press, arm curls, and triceps extension. General and specific warm-ups were performed prior to each training session and each training session was followed by cool-down. This training was circularly performed in 8 stations and included 2-4 sets with 8-12 maximal repetitions at 65-80% of 1 repetition maximum (1-RM) in each station. Each circuit and set was separated by 2-3 min and 60-90 s rest respectively. | Control group  - Participants were instructed not to participate in any organized or structured exercise during the 12 weeks of intervention. |
| --- | --- |

**Body composition outcomes and between groups comparison**

** statistically significant difference between running group and control group*

^†^ *statistically significant difference between running group and resistance group*

^‡^ *statistically significant difference within groups (pre-post)*

| Mean 8 weeks after baseline | | |
| --- | --- | --- |
| Running group  - Body weight (kg): 72.1 (SE 10.4)  - Body fat (%): 18.8 (SE 4.5)  - BMI (kg/m^2^): 24.6 (SE 2.9)  - Waist-hip ratio: 0.86 (SE 0.3) | Resistance group  - Body weight (kg): 69.6 (SE 9.9)  - Body fat (%): 16.2 (SE 4.8)  - BMI (kg/m^2^): 23.6 (SE 2.7)  - Waist-hip ratio: 0.86 (SE 0.05) | Control group  - Body weight (kg): 68.04 (SE 9.10)  - Body fat (%): 19.31 (SE 5.16)  - BMI (kg/m^2^): 25.02 (SE 3.3)  - Waist-hip ratio: 0.84 (SE 0.07) |

**Cardiorespiratory outcomes and between groups comparison**

** statistically significant difference between running group and control group*

^†^ *statistically significant difference between running group and resistance group*

^‡^ *statistically significant difference within groups (pre-post)*

| Mean 8 weeks after baseline | | |
| --- | --- | --- |
| Running group  - VO_2_max (ml/kg/min): 46.1 (SE 2.8)*^‡^ | Resistance group  - VO_2_max (ml/kg/min): 45.2 (SE 3.3)^‡^ | Control group  - VO_2_max (ml/kg/min): 36.73 (SE 4.13) |

**Blood serum concentration outcomes and between groups comparison**

** statistically significant difference between running group and control group*

^†^ *statistically significant difference between running group and resistance group*

^‡^ *statistically significant difference within groups (pre-post)*

*hs-CPR: high sensitive C-reactive protein*

*HOMA-IR: homeostasis model assessment of insulin resistance*

| Mean 8 weeks after baseline | | |
| --- | --- | --- |
| Running group  - Lipocalin-2 (µg/l): 17.71 (SE 6.89)*^‡^  - hs-CRP (mg/l): 0.78 (SE 0.70)  - Fasting glucose (mg/dl): 81.11 (SE 10.82)^‡^  - Fasting insulin (µU/ml): 9.82 (SE 2.73)^‡^  - HOMA-IR: 2.42 (SE 0.40)^‡^ | Resistance group  - Lipocalin-2 (µg/l): 19.91 (SE 6.50)^‡^  - hs-CRP (mg/l): 0.32 (SE 0.55)  - Fasting glucose (mg/dl): 82.21 (SE 10.64)^‡^  - Fasting insulin (µU/ml): 10.90 (SE 3.83)^‡^  - HOMA-IR: 2.21 (SE 0.84)^‡^ | Control group  - Lipocalin-2 (µg/l): 13.05 (SE 2.04)  - hs-CRP (mg/l): 0.89 (SE 0.80)  - Fasting glucose (mg/dl): 87.12 (SE 12.10)  - Fasting insulin (µU/ml): 10.96 (SE 3.01)  - HOMA-IR: 2.73 (SE 0.63) |

**Reference**

| Celik O, Salci Y, Ak E, Kalaci A, Korkusuz F. Serum cartilage oligomeric matrix protein accumulation decreases significantly after 12 weeks of running but not swimming and cycling training - a randomised controlled trial. The Knee. 2013;20(1):19-25. |
| --- |

**Objectives**

| To compare serum cartilage oligomeric matrix protein (COMP) levels of male university students before and after 12 weeks of regular exercise that will be measured prior to, immediately after and 30 min after moderate swimming, cycling and running exercise. |
| --- |

**Description of participants included in the study**

| - Participants randomised: n=48 sedentary male university students.  - Participants included in the analysis, n=44:   - Running group: n=11, mean age of 20.7 years (1.3). - Cycling group: n=11, mean age of 21.1 years (1.5). - Swimming group: n=11, mean age of 28.8 years (1.9). - Control group: n=11, mean age of 22.6 years (2.1). |
| --- |
| p.s.: The unit/qualifier of the dispersion/uncertainty measure for age were not reported in the article. |

**Follow-up period and time-point assessments after baseline**

| - Follow-up: 12 weeks.  - Time-points: baseline and 12 weeks after baseline. |
| --- |

**Intervention (running program)**

| Running group  - Duration: 12 weeks.  - Frequency: 3 times/week.  - Description: running sessions of 40 min per day. Participants exercised on a treadmill with a 1.5% incline. The speed of the treadmill was determined according to the individual heart-rate zone. Throughout the 12 weeks of training, heart-rate reserves were held constant (60–70%), while the speed increased according to individual progression. Each session began with a 5 min warm-up, continued with a main set of 30 min exercise at their individual target heart rate zone (60–70% of heart rate reserve) and finished with a 5 min cool-down period. |
| --- |

**Comparison group**

| Cycling group  - Duration: 12 weeks.  - Frequency: 3 times/week.  - Description: cycling sessions of 40 min per day. Cyclers exercised between 60 and 80 RPM on an ergometer. Each session began with a 5 min warm-up, continued with a main set of 30 min exercise at their individual target heart rate zone (60–70% of heart rate reserve) and finished with a 5 min cool-down period. | Swimming group  - Duration: 12 weeks.  - Frequency: 3 times/week.  - Description: swimming sessions of 40 min per day. Swimming exercises were composed of front-crawl swimming and kicking drills. Each session began with a 5 min warm-up, continued with a main set of 30 min exercise at their individual target heart rate zone (60–70% of heart rate reserve) and finished with a 5 min cool-down period. | Control group  - Participants were instructed not to participate in any organized or structured exercise during the 12 weeks of intervention. |
| --- | --- | --- |

**Body composition outcomes and between groups comparison**

** statistically significant difference between groups*

^‡^ *statistically significant difference within groups (pre-post)*

| Mean 12 weeks after baseline | | | |
| --- | --- | --- | --- |
| Running group  - BMI (kg/m^2^): result reported in a graph^‡^ | Cycling group  - BMI (kg/m^2^): result reported in a graph^‡^ | Swimming group  - BMI (kg/m^2^): result reported in a graph^‡^ | Control group  - BMI (kg/m^2^): result reported in a graph |

**Cardiorespiratory outcomes and between groups comparison**

** statistically significant difference between groups*

^‡^ *statistically significant difference within groups (pre-post)*

| Mean 12 weeks after baseline | | | |
| --- | --- | --- | --- |
| Running group  - VO_2_max (ml/kg/min): results reported in a graph^‡^ | Cycling group  - VO_2_max (ml/kg/min): results reported in a graph^‡^ | Swimming group  - VO_2_max (ml/kg/min): results reported in a graph^‡^ | Control group  - VO_2_max (ml/kg/min): results reported in a graph |

**Blood serum concentration outcomes and between groups comparison**

** statistically significant difference between groups*

^‡^ *statistically significant difference within groups (pre-post)*

*COMP: serum cartilage oligometric matrix protein*

| Mean difference between recovery minus fatigue states 12 weeks after baseline | | | |
| --- | --- | --- | --- |
| Running group (mean after 12 weeks)  - COMP: -1.57 (SE 0.67) | Cycling group (mean after 12 weeks)  - COMP: -2.42 (SE 0.36) | Swimming group (mean after 12 weeks)  - COMP: -2.88 (SE 0.39) | Control group (mean after 12 weeks)  - COMP: -2.74 (SE 0.32) |
| Mean difference between recovery minus rest states 12 weeks after baseline | | | |
| Running group (mean after 12 weeks)  - COMP: -0.55 (SE 0.29) | Cycling group (mean after 12 weeks)  - COMP: -0.88 (SE 0.26) | Swimming group (mean after 12 weeks)  - COMP: -0.99 (SE 0.33) | Control group (mean after 12 weeks)  - COMP: -0.86 (SE 0.23) |
| Mean difference between fatigue minus rest states 12 weeks after baseline | | | |
| Running group (mean after 12 weeks)  - COMP: 1.02 (SE 0.42) | Cycling group (mean after 12 weeks)  - COMP: 1.54 (SE 0.24) | Swimming group (mean after 12 weeks)  - COMP: 1.90 (SE 0.28) | Control group (mean after 12 weeks)  - COMP: 1.89 (SE 0.30) |

**Reference**

| Gregory SM, Spiering BA, Alemany JA, Tuckow AP, Rarick KR, Staab JS, et al. Exercise-induced insulin-like growth factor I system concentrations after training in women. Medicine and science in sports and exercise. 2013;45(3):420-8. |
| --- |

**Objectives**

| To examine changes in circulating growth hormone (GH) and insulin-like growth factor I (IGF-I) system component concentrations during acute heavy resistance exercise before and after 8 weeks of exercise training in women. |
| --- |

**Description of participants included in the study**

| - Participants randomised: n=58 women not engaged in structured physical activity more than 2 times/week in the previous 6 months.  - Source: university community.  - Participants included in the analysis, n=46, mean age of 20.3 years (SD 0.3):   - Running (endurance) group: n=12 - Resistance group: n=17 - Combined group (endurance + resistance): n=9 - Control group: n=8 |
| --- |

**Follow-up period and time-point assessments after baseline**

| - Follow-up: 8 weeks.  - Time-points: baseline and 8 weeks after baseline. |
| --- |

**Intervention (running program)**

| Running group  - Duration: 8 weeks.  - Frequency: 3 times/week.  - Aim: endurance training program aimed to improve the 3.2 km run time.  - Description: all sessions began with a 5-10 min warm-up consisting of light jogging and dynamic range of motion exercises and finished with a cool-down of similar duration. The 20-30 min training period alternated on different days between continuous running and sprint-type interval training. Continuous running was performed at ~70% (day 1) or ~80%–85% (day 3) of maximum heart rate. Interval training (day 2) involved a progression of near-maximal effort intervals of 400, 800, 1200, and 1600 m with a 1:1 effort-to-recovery ratio. |
| --- |

**Comparison group**

| Resistance group  - Duration: 8 weeks.  - Frequency: 3 times/week.  - Description: alternating days per week using a nonlinear periodized program in which intensity varied on a daily basis. Participants trained using 3 sets of moderate-to-heavy loads (3-RM to 12-RM) with 90–180 s of rest between sets. Exercises for the upper and lower body were performed during each session and included a combination of the following exercises: squat, stiff-leg dead lift, bench press, lat pulldown, upright row or high pull, calf exercises, abdominal work, leg press, incline bench press, seated row, and shoulder press or push press. The exercise session lasted between 40 and 63 min. | Combined group  - Duration: 8 weeks.  - Frequency: 3 times/week.  - Description: the same periodization schedules were followed as implemented in the running and resistance groups. The sessions were sequenced so that resistance training always preceded running training and that ‘‘light’’ resistance training days preceded ‘‘heavy’’ interval training. Participants trained at the same time of day as subjects in the running and resistance groups and strictly adhered to the exact protocol (including warm-up and cool down) for each training modality to limit variability in training stimuli or motivation. Combined exercise sessions lasted between 60 and 90 min. | Control group  - Participants were instructed to maintain their habitual physical activity level and did not participate in any formal training program. |
| --- | --- | --- |

**Body composition outcomes and between groups comparison**

** statistically significant difference between groups*

^‡^ *statistically significant difference within groups (pre-post)*

| Not applicable | Not applicable | Not applicable | Not applicable |
| --- | --- | --- | --- |

**Cardiorespiratory outcomes and between groups comparison**

** statistically significant difference between groups*

^‡^ *statistically significant difference within groups (pre-post)*

| Pre-post mean difference 8 weeks after baseline | | | |
| --- | --- | --- | --- |
| Running group  - ΔVO_2_max (ml/kg/min): 2.1 (SD 2.9)^‡^ | Resistance group  - ΔVO_2_max (ml/kg/min): not reported | Combined group  - ΔVO_2_max (ml/kg/min): 3.1 (SD 2.5)^‡^ | Control group  - ΔVO_2_max (ml/kg/min): not reported |

**Blood serum concentration outcomes and between groups comparison**

** statistically significant difference between groups*

^‡^ *statistically significant difference within groups (pre-post)*

*GH: growth hormone*

*IGF-I: insulin-like growth factor I*

*IGFBP-1: IGF blinding protein-1*

*IGFBP-2: IGF blinding protein-2*

*IGFBP-3: IGF blinding protein-3*

| Pre-post mean difference 8 weeks after baseline | | | |
| --- | --- | --- | --- |
| Running group  - ΔGH (µg/l): result reported in a graph  - ΔIGF-I (µg/l): result reported in a graph  - ΔFree-IGF-I (µg/l): result reported in a graph  - ΔIGFIBP-1 (µg/l): result reported in a graph  - ΔIGFIBP-2 (µg/l): result reported in a graph  - ΔIGFIBP-3 (µg/l): result reported in a graph | Resistance group  - ΔGH (µg/l): result reported in a graph  - ΔIGF-I (µg/l): result reported in a graph  - ΔFree-IGF-I (µg/l): result reported in a graph  - ΔIGFIBP-1 (µg/l): result reported in a graph  - ΔIGFIBP-2 (µg/l): result reported in a graph  - ΔIGFIBP-3 (µg/l): result reported in a graph | Combined group  - ΔGH (µg/l): result reported in a graph  - ΔIGF-I (µg/l): result reported in a graph  - ΔFree-IGF-I (µg/l): result reported in a graph  - ΔIGFIBP-1 (µg/l): result reported in a graph  - ΔIGFIBP-2 (µg/l): result reported in a graph  - ΔIGFIBP-3 (µg/l): result reported in a graph | Control group  - ΔGH (µg/l): result reported in a graph  - ΔIGF-I (µg/l): result reported in a graph  - ΔFree-IGF-I (µg/l): result reported in a graph  - ΔIGFIBP-1 (µg/l): result reported in a graph  - ΔIGFIBP-2 (µg/l): result reported in a graph  - ΔIGFIBP-3 (µg/l): result reported in a graph |
| p.s.: Results to these variables were reported in graphs combining part of all groups. | | | |

**More publications related to this study**

| Lester ME, Urso ML, Evans RK, Pierce JR, Spiering BA, Maresh CM, et al. Influence of exercise mode and osteogenic index on bone biomarker responses during short-term physical training. Bone. 2009;45(4):768-76. |
| --- |

**Reference**

| Asad M, Ravasi AA, Faramarzi M, Pournemati P. The effects of three training methods endurance, resistance and concurrent on adiponectin resting levels in overweighed untrained men. Bratislavske lekarske listy. 2012;113(11):664-8. |
| --- |

**Objectives**

| To investigate the effect of three training methods, endurance, resistance and concurrent on adiponectin in sedentary healthy men. |
| --- |

**Description of participants included in the study**

| - Participants randomised: n=45 sedentary male college students.  - Participants included in the analysis, n=44:   - Endurance (running) group: n=12, mean age of 22.0 years (SD 0.89). - Resistance group: n=9, mean age of 21.0 years (SD 1.57). - Concurrent group: n=13, mean age of 21.38 years (SD 2.06). - Control group: n=10, mean age of 21.44 years (SD 1.13). |
| --- |

**Follow-up period and time-point assessments after baseline**

| - Follow-up: 8 weeks.  - Time-points: baseline and 8 weeks after baseline. |
| --- |

**Intervention (running program)**

| Endurance (running) group  - Duration: 8 weeks.  - Frequency: 3 times/week.  - Description: running program with duration and intensity increased gradually at the end of each level. At 1^st^ and 2^nd^ weeks the participants did exercises with 65% of maximal heart rate for 25 min. For 3^rd^ until 6^th^ weeks 35 min with 65-75% maximal heart rate and in 7^th^ till 8^th^ weeks 40 min with 75-85% maximal heart rate. |
| --- |

**Comparison group**

| Resistance group  - Duration: 8 weeks.  - Frequency: 3 times/week.  - Description: resistance training program with machines and free loads. The training program contained upper body training and lower body training, such as: bench press, sitting and standing up with halter, leg extension, leg flexion, and leg press and rowing. In the first 2 weeks, the participants did 3 sets with 10-15 repetition and in the next 6 weeks training was set in a way that they couldn't do 10-12 repetition for 1^st^ set, 8-10 repetition for 2^nd^ set and 4-8 repatriation for 3^rd^ set. | Concurrent group  - Duration: 8 weeks.  - Frequency: 3 times/week.  - Description: training program that was exactly sum of endurance and resistance training. For this group endurance and resistance training programs was done exactly in the same days with 2 other groups but always resistance training was done first. | Control group  - Not described. |
| --- | --- | --- |

**Body composition outcomes and between groups comparison**

** statistically significant difference between groups*

^‡^ *statistically significant difference within groups (pre-post)*

| Mean 8 weeks after baseline | | | |
| --- | --- | --- | --- |
| Running group  - Body weight (kg): 87.50 (SD 11.0)  - BMI (kg/m^2^): 28.70 (SD 3.59) | Resistance group  - Body weight (kg): 89.17 (SD 15.29)  - BMI (kg/m^2^): 30.44 (SD 4.80) | Concurrent group  - Body weight (kg): 83.46 (SD 14.2)  - BMI (kg/m^2^): 27.38 (SD 3.67) | Control group  - Body weight (kg): 90.33 (SD 14.6)  - BMI (kg/m^2^): 29.29 (SD 4.40) |

**Cardiorespiratory outcomes and between groups comparison**

** statistically significant difference between groups*

^‡^ *statistically significant difference within groups (pre-post)*

| Mean 8 weeks after baseline | | | |
| --- | --- | --- | --- |
| Running group  - VO_2_max (unit not reported): 33.75 (SD 5.38) | Resistance group  - VO_2_max (unit not reported): 32.49 (SD 4.04) | Concurrent group  - VO_2_max (unit not reported): 35.64 (SD 6.44) | Control group  - VO_2_max (unit not reported): 31.02 (SD 5.00) |

**Blood serum concentration outcomes and between groups comparison**

** statistically significant difference between groups*

^‡^ *statistically significant difference within groups (pre-post)*

| Mean 8 weeks after baseline | | | |
| --- | --- | --- | --- |
| Running group  - Adiponectin (unit not reported): 17.56 (SD 1.51) | Resistance group  - Adiponectin (unit not reported): 17.56 (SD 1.51) | Concurrent group  - Adiponectin (unit not reported): 20.38 (SD 7.61) | Control group  - Adiponectin (unit not reported): 18.80 (SD 2.69) |

**Reference**

| Hosseini M, Piri M, Agha-Alinejad H, Haj-Sadeghi S. The effect of endurance, resistance and concurrent training on the heart structure of female students. Biology of Sport. 2012;29(1):17-21. |
| --- |

**Objectives**

| To assess the effects of endurance, resistance, and concurrent training on female cardiac structure. |
| --- |

**Description of participants included in the study**

| - Participants randomised: n=39 female students with lack of previous regular physical training.  - Participants included in the analysis, n=39:   - Endurance (running) group: n=10, mean age of 25.3 years (3.6). - Resistance group: n=10, mean age of 23.4 (1.6). - Concurrent group: n=10, mean age of 23.2 (2.5). - Control group: n=9, mean age of 25.2 (2.5). |
| --- |
| p.s.: The article did not describe the unit for the dispersion or uncertainty measure. |

**Follow-up period and time-point assessments after baseline**

| - Follow-up: 8 weeks.  - Time-points: baseline and 8 weeks after baseline. |
| --- |

**Intervention (running program)**

| Endurance (running) group  - Duration: 8 weeks.  - Frequency: 3 times/week.  - Description: jogging 3 days/week with the intensity of 65% of maximum heart rate on a treadmill for 16 min/session during the 1^st^ week, reaching 80% of maximum heart rate for 30 min during the 8^th^ week. Participants warmed up for 10 min before and cooled down for 10 min after the main program. |
| --- |

**Comparison group**

| Resistance group  - Duration: 8 weeks.  - Frequency: 3 times/week.  - Description: leg press, bench press, pull down curls and leg curls. During the 1^st^ week the exercises were performed with 50% of one repetition maximum (1RM) in 2 sets with 10 repetitions and a recovery period of 1-2 min. the intensity increased to 80% of 1RM in 3 sets with 6 repetitions during the 8^th^ week. Participants warmed up for 10 min before and cooled down for 10 min after the main program. | Concurrent group  - Duration: 8 weeks.  - Frequency: 3 times/week.  - Description: sum of the endurance and resistance programs in each session. The resistance training was performed before the endurance training. Participants warmed up for 10 min before and cooled down for 10 min after the main program. | Control group  - Not described. |
| --- | --- | --- |

**Body composition outcomes and between groups comparison**

** statistically significant difference between groups*

^‡^ *statistically significant difference within groups (pre-post)*

| Not applicable | Not applicable | Not applicable | Not applicable |
| --- | --- | --- | --- |

**Cardiorespiratory outcomes and between groups comparison**

** statistically significant difference between groups*

^‡^ *statistically significant difference within groups (pre-post)*

*LVEDD: left ventricular end diastolic diameter*

*LVESD: left ventricular end systolic diameter*

*SWT: septum wall thickness*

*PWT: posterior wall thickness*

*LVM: left ventricular mass*

*LVMI: left ventricular mass index*

| Mean 8 weeks after baseline | | | |
| --- | --- | --- | --- |
| Running group  - LVEDD (mm): 44.4 (3.1)^‡^  - LVESD (mm): 26.7 (3.1)  - SWT (mm): 7.9 (1.8)  - PWT (mm): 5.8 (1.2)  - LVM (g): 98.8 (36.5)  - LVMI (g/m^2^): 61.8 (20.4) | Resistance group  - LVEDD (mm): 44.4 (4.0)  - LVESD (mm): 26.4 (2.3)  - SWT (mm): 6.9 (0.6)  - PWT (mm): 6.2 (1.5)  - LVM (g): 93.1 (23.0)  - LVMI (g/m^2^): 56.0 (9.1) | Concurrent group  - LVEDD (mm): 45.4 (5.7)^‡^  - LVESD (mm): 27.0 (3.4)^‡^  - SWT (mm): 6.8 (0.6)  - PWT (mm): 6.4 (1.4)  - LVM (g): 91.5 (23.7)^‡^  - LVMI (g/m^2^): 56.3 (10.2)^‡^ | Control group  - LVEDD (mm): 41.0 (2.2)  - LVESD (mm): 24.5 (2.0)  - SWT (mm): 6.7 (1.9)  - PWT (mm): 5.4 (1.1)  - LVM (g): 70.7 (11.8)  - LVMI (g/m^2^): 46.0 (6.7) |
| p.s.: The article did not describe the unit for the dispersion or uncertainty measure. | | | |

**Blood serum concentration outcomes and between groups comparison**

** statistically significant difference between groups*

^‡^ *statistically significant difference within groups (pre-post)*

| Not applicable | Not applicable | Not applicable | Not applicable |
| --- | --- | --- | --- |

**Reference**

| Lo MS, Lin LL, Yao WJ, Ma MC. Training and detraining effects of the resistance vs. endurance program on body composition, body size, and physical performance in young men. Journal of strength and conditioning research / National Strength & Conditioning Association. 2011;25(8):2246-54. |
| --- |

**Objectives**

| To investigate changes in the body composition, body size, muscle strength, and VO_2_max of untrained young men during training and detraining in response to resistance and endurance programs. |
| --- |

**Description of participants included in the study**

| - Participants randomised: n=34 nonathletic male students who had not been exercising regularly for the past year.  - Participants included in the analysis, n=30:   - Endurance (running) group: n=10, mean age of 20.0 years (SD 0.67). - Resistance: n=10, mean age of 20.2 years (SD 1.40). - Control group: n=10, mean age of 21.1 years (SD 1.66). |
| --- |

**Follow-up period and time-point assessments after baseline**

| - Follow-up: 24 weeks.  - Time-points: baseline and 24 weeks after baseline. |
| --- |

**Intervention (running program)**

| Endurance (running) group  - Duration: 24 weeks.  - Frequency: 3 times/week.  - Description: 30 min run on a treadmill machine, maintained at an intensity of 70–85% heart rate reserve. |
| --- |

**Comparison group**

| Resistance group  - Duration: 24 weeks.  - Frequency: 3 times/week.  - Description: gradually progressive, supervised strength training with at least 48 hours of rest between the training sessions. Five minutes of brisk walking on the treadmill before resistance training and a whole body stretch before and after training were employed. Participants exercised on selected resistance machines to focus on 10 major muscle groups in the following order: seated chest press, lat pull down, seated shoulder press, seated biceps curl, seated triceps extension, seated leg extension, lying leg curl, seated back extension, seated abdominal curl, and standing calf raise. Participants performed at a weight that they could lift easily in a circuit training workout of 15 repetitions for the first 8 weeks, then 1 set at 75% of 1 repetition maximum (RM) for 10 repetitions for the next 8 weeks, and 2 sets at 90% 1RM for 4 repetitions thereafter. The weight lifted was increased by 5% when subjects could perform the last repetition with ease and good form. | Control group  - Participants were instructed to continue their habitual physical activities and reminded not to do any extra exercise during the course of the study. |
| --- | --- |

**Body composition outcomes and between groups comparison**

** statistically significant difference between running group and control group*

^†^ *statistically significant difference between running group and resistance group*

^‡^ *statistically significant difference within groups (pre-post)*

| Mean 24 weeks after baseline | | |
| --- | --- | --- |
| Running group  - Body weight (kg): 67.2 (SD 8.66)  - BMI (kg/m^2^): 22.3 (SD 2.34)  - Waist-hip ratio: 0.8 (SD 0.04)  - Waist circumference (cm): 76.6 (SD 6.56)  - Hip circumference (cm): 95.4 (SD 6.45)  - Upper arm circumference (cm): 28.7 (SD 2.06)^†^  - Thigh circumference (cm): 56.8 (SD 3.25)  - Body fat (%): 19.4 (SD 6.30)  - Body lean mass (kg): 50.91 (SD 3.567)^†^  - Body fat (kg): 13.52 (SD 5.928)  - Arm fat (%): 15.4 (SD 6.19)  - Arm lean mass (kg): 6.3 (SD 0.55)^†^  - Arm fat (kg): 1.3 (SD 0.70)  - Leg fat (%): 19.8 (SD 4.71)  - Leg lean mass (kg): 18.60 (SD 1.50)  - Leg fat (kg): 5.0 (SD 1.72) | Resistance group  - Body weight (kg): 63.4 (SD 8.10)  - BMI (kg/m^2^): 21.7 (SD 3.36)  - Waist-hip ratio: 0.8 (SD 0.04)  - Waist circumference (cm): 75.0 (SD 5.43)  - Hip circumference (cm): 95.1 (SD 4.96)  - Upper arm circumference (cm): 29.7 (SD 3.46)  - Thigh circumference (cm): 56.3 (SD 4.10)  - Body fat (%): 16.4 (SD 7.03)  - Body lean mass (kg): 50.77 (SD 3.279)  - Body fat (kg): 11.00 (SD 5.990)  - Arm fat (%): 12.4 (SD 6.69)  - Arm lean mass (kg): 6.5 (SD 0.63)  - Arm fat (kg): 1.0 (SD 0.70)  - Leg fat (%): 17.9 (SD 6.86)  - Leg lean mass (kg): 18.3 (SD 1.41)  - Leg fat (kg): 4.5 (SD 2.29) | Control group  - Body weight (kg): 67.7 (SD 11.23)  - BMI (kg/m^2^): 22.7 (SD 4.82)  - Waist-hip ratio: 0.8 (SD 0.08)  - Waist circumference (cm): 77.0 (SD 8.81)  - Hip circumference (cm): 98.4 (SD 8.10)  - Upper arm circumference (cm): 28.6 (SD 4.28)  - Thigh circumference (cm): 56.5 (SD 4.76)  - Body fat (%): 22.3 (SD 9.67)  - Body lean mass (kg): 48.77 (SD 3.259)  - Body fat (kg): 15.78 (SD 10.049)  - Arm fat (%): 19.4 (SD 10.47)  - Arm lean mass (kg): 5.9 (SD 0.63)  - Arm fat (kg): 1.7 (SD 1.38)  - Leg fat (%): 22.6 (SD 8.84)  - Leg lean mass (kg): 17.9 (SD 1.40)  - Leg fat (kg): 6.0 (SD 3.91) |
| Pre-post mean difference 24 weeks after baseline | | |
| Running group  - ΔWaist-hip ratio: -0.03 (SD 0.07)  - ΔWaist circumference (cm): -0.53 (SD 2.13)  - ΔHip circumference (cm): 1.0 (SD 5.00)  - ΔUpper arm circumference (cm): -0.4 (SD 1.07)^†^  - ΔThigh circumference (cm): 1.1 (SD 1.80)  - ΔBody fat (%): -0.6 (SD 3.29)  - ΔBody lean mass (kg): -0.552 (SD 0.472)^†^  - ΔBody fat (kg): -0.452 (SD 2.973)  - ΔArm fat (%): -0.6 (SD 2.98)  - ΔArm lean mass (kg): -0.1 (SD 0.16)^†^  - ΔArm fat (kg): -0.1 (SD 0.30)  - ΔLeg fat (%): -0.3 (SD 2.73)  - ΔLeg lean mass (kg): -0.4 (SD 0.47)  - ΔLeg fat (kg): -0.2 (SD 0.90) | Resistance group  - ΔWaist-hip ratio: -0.01 (SD 0.02)  - ΔWaist circumference (cm): 0.28 (SD 2.61)  - ΔHip circumference (cm): 0.9 (SD 2.49)  - ΔUpper arm circumference (cm): 1.9 (SD 1.76)  - ΔThigh circumference (cm): 2.4 (SD 2.51)  - ΔBody fat (%): -0.4 (SD 1.99)  - ΔBody lean mass (kg): 0.308 (SD 1.590)  - ΔBody fat (kg): -0.219 (SD 1.565)  - ΔArm fat (%): -0.9 (SD 1.58)  - ΔArm lean mass (kg): 0.4 (SD 0.29)  - ΔArm fat (kg): -0.02 (SD 0.13)  - ΔLeg fat (%): 0.1 (SD 2.03)  - ΔLeg lean mass (kg): 0.02 (SD 0.60)  - ΔLeg fat (kg): 0.04 (SD 0.59) | Control group  - ΔWaist-hip ratio: -0.01 (SD 0.03)  - ΔWaist circumference (cm): 0.67 (SD 3.71)  - ΔHip circumference (cm): 2.3 (SD 3.04)  - ΔUpper arm circumference (cm): 0.5 (SD 1.78)  - ΔThigh circumference (cm): 1.3 (SD 2.53)  - ΔBody fat (%): 1.1 (SD 3.01)  - ΔBody lean mass (kg): -0.421 (SD 0.588)  - ΔBody fat (kg): 1.00 (SD 2.470)  - ΔArm fat (%): 0.4 (SD 3.00)  - ΔArm lean mass (kg): -0.1 (SD 0.34)  - ΔArm fat (kg): 0.1 (SD 0.32)  - ΔLeg fat (%): 1.1 (SD 3.10)  - ΔLeg lean mass (kg): 0.02 (SD 0.70)  - ΔLeg fat (kg): 0.5 (SD 1.01) |

**Cardiorespiratory outcomes and between groups comparison**

** statistically significant difference between running group and control group*

^†^ *statistically significant difference between running group and resistance group*

^‡^ *statistically significant difference within groups (pre-post)*

| Mean 24 weeks after baseline | | |
| --- | --- | --- |
| Running group  - Max heart rate (bpm): 195.1 (SD 7.45)  - VO_2_max (unit not reported): result reported in a graph | Resistance group  - Max heart rate (bpm): 196.2 (SD 9.16)  - VO_2_max (unit not reported): result reported in a graph | Control group  - Max heart rate (bpm): 192.1 (SD 10.09)  - VO_2_max (unit not reported): result reported in a graph |
| Pre-post mean difference 24 weeks after baseline | | |
| Running group  - ΔVO_2_max (%): 17%* | Resistance group  - ΔVO_2_max (%): 12% | Control group  - ΔVO_2_max (%): not reported |

**Blood serum concentration outcomes and between groups comparison**

** statistically significant difference between running group and control group*

^†^ *statistically significant difference between running group and resistance group*

^‡^ *statistically significant difference within groups (pre-post)*

| Not applicable | Not applicable | Not applicable |
| --- | --- | --- |

**Reference**

| Andersen LJ, Hansen PR, Sogaard P, Madsen JK, Bech J, Krustrup P. Improvement of systolic and diastolic heart function after physical training in sedentary women. Scandinavian journal of medicine & science in sports. 2010;20 Suppl 1:50-7. |
| --- |

**Objectives**

| To investigate the effect of two different types of aerobic physical training on cardiac morphology and function in sedentary young and middle-aged women. |
| --- |

**Description of participants included in the study**

| - Participants randomised: n=47 women who had not been involved in any type of physical training for at least 2 years.  - Participants included in the analysis, n=47, mean age of 36.5 years (SD 8.2):   - Running group: n=18. - Football (soccer) group: n=19. - Control group: n=10. |
| --- |

**Follow-up period and time-point assessments after baseline**

| - Follow-up: 16 weeks.  - Time-points: baseline and 16 weeks after baseline. |
| --- |

**Intervention (running program)**

| Running group  - Duration: 16 weeks.  - Frequency: 2-3 times/week.  - Description: 1 h running sessions consisted of continuous moderate intensity running at the same average intensity as the football group (82% of individual maximal heart rate).  - Total running sessions: 29.5 (1.9/week) |
| --- |
| p.s.: As the running group had problems coping with 1 h of continuous running, both groups had their training sessions split into 3-4 segments over the first 6 weeks. |

**Comparison group**

| Football group  - Duration: 16 weeks.  - Frequency: 2-3 times/week.  - Description: the training (1 h/session) consisted of ordinary five-a-side, seven-a-side or nine- a-side matches on a 30–40 m wide and 45–60 m long natural grass pitch. The intensity was at the same average intensity as the running group (82% of individual maximal heart rate).  - Total football sessions: 28.8 (1.8/week) | Control group  - Participants continued their daily life activities during the observation period. |
| --- | --- |

**Body composition outcomes and between groups comparison**

** statistically significant difference between running group and control group*

^†^ *statistically significant difference between running group and football group*

^‡^ *statistically significant difference within groups (pre-post)*

| Not applicable | Not applicable | Not applicable |
| --- | --- | --- |

**Cardiorespiratory outcomes and between groups comparison**

** statistically significant difference between running group and control group*

^†^ *statistically significant difference between running group and football group*

^‡^ *statistically significant difference within groups (pre-post)*

*E: early diastole*

*E’: early diastolic velocity*

*A: atrial contraction*

*A’: late diastolic velocity*

*S’: peak systolic velocity*

*IVRT: isovulometric relaxation time*

*RVDD: right ventricular end-diastolic diameter*

*TAPSE: tricuspid annular plane systolic excursion*

*TT_index_: systolic longitudinal shortening*

| Mean 16 weeks after baseline | | |
| --- | --- | --- |
| Running group  - Mean arterial blood pressure (mmHg): 81^‡^  - Resting heart rate (bpm): 56^‡^  - VO_2_max (ml/kg/min): 39.1 (SD 5.5)^‡^  - Left ventricular diameter, end-diastole (mm): 46 (SD 3)  - Left ventricular diameter, end-systole (mm): 29 (SD 4)  - Septum thickness, end-diastole (mm): 8.5 (SD 1.0)  - Posterior wall thickness, end-diastole (mm): 8.4 (SD 1.3)  - Left ventricular volume, biplane (ml): 97 (SD 18)*^‡^  - Left ventricular ejection fraction (%): 63 (SD 7)  - E (m/s): 0.94 (SD 0.20)  - Early diastole deceleration time (ms): 229 (SD 58)^‡^  - A (m/s): 0.43 (SD 0.08)*^‡^  - *E/A* ratio: 2.3 (SD 0.6)*^‡^  - IVRT_global_ (ms): 52 (SD 9)*^†‡^  - RVDD (unit/qualifier not reported): 29 (SD 3)^‡^  - TAPSE (unit/qualifier not reported): 25 (SD 3)*^‡^  - E’ (cm/s): 18.2 (SD 3.3)^‡^  - A’ (cm/s): 8.2 (SD 2.1)  - *S’* (cm/s): 11.3 (SD 1.9)^‡^  - *E/E’*: 5.4 (SD 1.5)  - *S’*_ColorTDI_ (cm/s): 6.6 (SD 0.8)*  - *TT_index_* (mm): 12.7 (SD 1.9)*^‡^  - IVRT_averaged_ (ms): 47 (SD 10)*^‡^ | Football group  - Mean arterial blood pressure (mmHg): 82^‡^  - Resting heart rate (bpm): 57^‡^  - VO_2_max (ml/kg/min): 37.5 (SD 4.9)^‡^  - Left ventricular diameter, end-diastole (mm): 46 (SD 3)  - Left ventricular diameter, end-systole (mm): 28 (SD 4)  - Septum thickness, end-diastole (mm): 8.0 (SD 1.3)  - Posterior wall thickness, end-diastole (mm): 9.0 (SD 1.3)^‡^  - Left ventricular volume, biplane (ml): 100 (SD 18)^‡^  - Left ventricular ejection fraction (%): 60 (SD 5)  - E (m/s): 0.96 (SD 0.16)^‡^  - Early diastole deceleration time (ms): 206 (SD 32)  - A (m/s): 0.46 (SD 0.13)^‡^  - *E/A* ratio: 2.2 (SD 0.6)^‡^  - IVRT_global_ (ms): 46 (SD 10)^‡^  - RVDD (unit/qualifier not reported): 29 (SD 4)^‡^  - TAPSE (unit/qualifier not reported): 25 (SD 4)^‡^  - E’ (cm/s): 19.3 (SD 4.0)^‡^  - A’ (cm/s): 8.2 (SD 2.1)  - *S’* (cm/s): 11.7 (SD 2.6)^‡^  - *E/E’*: 5.0 (SD 0.9)  - *S’*_ColorTDI_ (cm/s): 6.4 (SD 0.8)^‡^  - *TT_index_* (mm): 11.9 (SD 1.1)^‡^  - IVRT_averaged_ (ms): 46 (SD 9)^‡^ | Control group  - Mean arterial blood pressure (mmHg): not reported  - Resting heart rate (bpm): not reported  - VO_2_max (ml/kg/min): not reported  - Left ventricular diameter, end-diastole (mm): 47 (SD 4)  - Left ventricular diameter, end-systole (mm): 30 (SD 5)  - Septum thickness, end-diastole (mm): 8.1 (SD 1.2)  - Posterior wall thickness, end-diastole (mm): 8.4 (SD 1.1)  - Left ventricular volume, biplane (ml): 98 (SD 25)  - Left ventricular ejection fraction (%): 58 (SD 7)  - E (m/s): 0.80 (SD 0.11)  - Early diastole deceleration time (ms): 227 (SD 40)  - A (m/s): 0.43 (SD 0.11)  - *E/A* ratio: 1.9 (SD 0.5)  - IVRT_global_ (ms): 60 (SD 10)  - RVDD (unit/qualifier not reported): 28 (SD 5)  - TAPSE (unit/qualifier not reported): 22 (SD 2)^‡^  - E’ (cm/s): 18.2 (SD 3.0)  - A’ (cm/s): 8.8 (SD 2.3)  - *S’* (cm/s): 10.7 (SD 1.2)  - *E/E’*: 4.5 (SD 0.7)  - *S’*_ColorTDI_ (cm/s): 6.2 (SD 0.9)  - *TT_index_* (mm): 10.4 (SD 1.3)^‡^  - IVRT_averaged_ (ms): 59 (SD 6) |

**Blood serum concentration outcomes and between groups comparison**

** statistically significant difference between running group and control group*

^†^ *statistically significant difference between running group and football group*

^‡^ *statistically significant difference within groups (pre-post)*

| Not applicable | Not applicable | Not applicable |
| --- | --- | --- |

**More publications related to this study**

| Krustrup P, Hansen PR, Andersen LJ, Jakobsen MD, Sundstrup E, Randers MB, et al. Long-term musculoskeletal and cardiac health effects of recreational football and running for premenopausal women. Scandinavian journal of medicine & science in sports. 2010;20 Suppl 1:58-71. |
| --- |

**Reference**

| Hendrickson NR, Sharp MA, Alemany JA, Walker LA, Harman EA, Spiering BA, et al. Combined resistance and endurance training improves physical capacity and performance on tactical occupational tasks. European journal of applied physiology. 2010;109(6):1197-208. |
| --- |

**Objectives**

| 1- To evaluate the effectiveness of aerobic endurance, strength, and the additive effect of strength and endurance training for improving the performance of common tactical occupational tasks among recreationally active women;  2- To determine if combined training interfered with improvements in muscular strength, power or endurance afforded by either strength or aerobic endurance training alone;  3- To identify relationships between changes in maximal physical performance and occupational task performance. |
| --- |

**Description of participants included in the study**

| - Participants randomised: n=not reported.  - Source: state university community.  - Participants included in the analysis, n=56 women not participating in any competitive sports or structured physical training program more than 2 times/week for the preceding 6 months:   - Running group: n=13, mean of age 21 years (SD 0.4). - Resistance group: n=18, mean of age 21 years (SD 0.5). - Combined group: n=15, mean of age 20 years (SD 0.4). - Control group: n=10, mean of age 20 years (SD 0.5). |
| --- |

**Follow-up period and time-point assessments after baseline**

| - Follow-up: 12 weeks.  - Time-points: baseline and 12 weeks after baseline. |
| --- |

**Intervention (running program)**

| Running group  - Duration: 12 weeks.  - Frequency: 3 times/week.  - Aim: to improve 3.2 km running time.  - Description: 3 alternating sessions/week periodized between continuous running and sprint-type interval training on different days. All sessions began with a 5–10 min warm up that included light jogging and dynamic range of motion exercises before training and ended with a 5–10 min cool down. The weekly training was comprised of 20–30 min of continuous running at a prescribed target heart rate of 70–85% maximum heart rate, or interval running consisting of 400, 800, 1,200, and 1,600 m runs conducted close to maximal intensity with a 1:1 recovery time. |
| --- |

**Comparison group**

| Resistance group  - Duration: 12 weeks.  - Frequency: 3 times/week  - Description: 3 alternating days, with training sessions lasting between 40 and 63 min. Following a 2-week pre-testing and familiarization period, the initial training (weeks 3–6) consisted of ‘‘light’’ days using 12 RM loads, ‘‘moderate’’ days using 8–10 RM loads, and ‘‘heavy’’ days using 6–8 RM loads. During weeks 8–11, ‘‘light’’ days utilized 12 RM loads, ‘‘moderate’’ days utilized 6–8 RM loads, and ‘‘heavy’’ days utilized 3–5 RM loads. | Combined group  - Duration: 12 weeks.  - Frequency: 3 times/week.  - Description: both the endurance and resistance programs on the same day, during the same session. “Light” resistance training days always corresponded to the interval sprint days in an attempt to not limit the intensity of either training session. Strength training sessions were performed first and were immediately followed by the endurance training session. Subjects trained at the same time as the respective endurance and resistance groups in order to ensure that they received the same encouragement and coaching. | Control group  - This group did not undergo any formalized physical training. |
| --- | --- | --- |

**Body composition outcomes and between groups comparison**

** statistically significant difference between running group and control group*

^†^ *statistically significant difference between running group and resistance group*

*^‡^ statistically significant difference within groups (pre-post)*

| Pre-post mean difference 12 weeks after baseline | | | |
| --- | --- | --- | --- |
| Running group  - ΔBody fat (%): -3.4 (SD 4.8)^†^ | Resistance group  - ΔBody fat (%): 0.5 (SD 4.4) | Combined group  - ΔBody fat (%): not reported | Control group  - ΔBody fat (%): not reported |
| Mean 12 weeks after baseline | | | |
| - Body fat (%): 32.6 (SD 5.9)  - Lean mass (kg): 41.6 (SD 3.8) | - Body fat (%): 31.3 (SD 5.6)  - Lean mass (kg): 42.2 (SD 6.2) | - Body fat (%): 32.4 (SD 7.0)  - Lean mass (kg): 41.8 (SD 4.3) | - Body fat (%): 31.8 (SD 9.8)  - Lean mass (kg): 42.5 (SD 2.2) |

**Cardiorespiratory outcomes and between groups comparison**

** statistically significant difference between running group and control group*

^†^ *statistically significant difference between running group and resistance group*

*^‡^ statistically significant difference within groups (pre-post)*

| Pre-post mean difference 12 weeks after baseline | | | |
| --- | --- | --- | --- |
| Running group  - ΔVO_2_peak (ml/kg/min): 6.2 (SD reported in a graph)*^†‡^ | Resistance group  - ΔVO_2_peak (ml/kg/min): 2.1 (SD reported in a graph) | Combined group  - ΔVO_2_peak (ml/kg/min): 7.6 (SD reported in a graph)^‡^ | Control group  - ΔVO_2_peak (ml/kg/min): 1.0 (SD reported in a graph) |
| Mean 12 weeks after baseline | | | |
| Running group  - VO_2_peak (ml/kg/min): 42.4 (SE 1.3) | Resistance group  - VO_2_peak (ml/kg/min): 38.9 (SE 1.1) | Combined group  - VO_2_peak (ml/kg/min): 41.5 (SE 1.2) | Control group  - VO_2_peak (ml/kg/min): 38.3 (SE 1.4) |

**Blood serum concentration outcomes and between groups comparison**

** statistically significant difference between running group and control group*

^†^ *statistically significant difference between running group and resistance group*

*^‡^ statistically significant difference within groups (pre-post)*

| Not applicable | Not applicable | Not applicable | Not applicable |
| --- | --- | --- | --- |

**More publications related to this study**

| Nindl BC, Alemany JA, Tuckow AP, Rarick KR, Staab JS, Kraemer WJ, et al. Circulating bioactive and immunoreactive IGF-I remain stable in women, despite physical fitness improvements after 8 weeks of resistance, aerobic, and combined exercise training. Journal of applied physiology. 2010;109(1):112-20. |
| --- |

**Reference**

| Krustrup P, Hansen PR, Andersen LJ, Jakobsen MD, Sundstrup E, Randers MB, et al. Long-term musculoskeletal and cardiac health effects of recreational football and running for premenopausal women. Scandinavian journal of medicine & science in sports. 2010;20 Suppl 1:58-71. |
| --- |

**Objectives**

| To investigate the musculoskeletal and cardiac adaptations elicited by 4 and 16 months of recreational football and running in previously untrained premenopausal women. |
| --- |

**Description of participants included in the study**

| - Participants randomised: n=28 females who had not been involved in regular physical training activities for at least 2 years.  - Participants included in the analysis, n=22:   - Running group: n=8, mean age of 40 (SE 3). - Football group: n=7, mean age of 40 (SE 2). - Control group: n=7, mean age of 38 (SE 4). |
| --- |

**Follow-up period and time-point assessments after baseline**

| - Follow-up: 16 months.  - Time-points: baseline, 4 and 16 months after baseline. |
| --- |

**Intervention (running program)**

| Running group  - Duration: 16 months.  - Frequency: 2 times/week.  - Description: running training for 1 hours/session. The running training was performed outdoor during the entire period. The running speed was individually adjusted to elicit the heart rate of 81% (SE 1) and 82% (SE 1) of maximal heart rate during the first 4 and last 12 months, respectively. |
| --- |

**Comparison group**

| Football group  - Duration: 16 months.  - Frequency: 2 times/week.  - Description: football training for 1 hours/session. The football training sessions consisted of ordinary four-a-side or five-a-side games on a pitch that was 20–30 m wide and 30–40 m long. During the first 8 months and the last 4 months, the football training was performed outdoor on natural grass and during the months 9–12, the training was performed indoor on a wooden floor. The average training intensity during the football training sessions was 82% (SE 2) of maximal heart rate for the first 4 month and 81% (SE 1) of maximal heart rate for the next 12 months. | Control group  - Participants performed no physical training. |
| --- | --- |

**Body composition outcomes and between groups comparison**

** statistically significant difference between running group and control group*

^†^ *statistically significant difference between running group and football group*

^‡^ *statistically significant difference within groups (from baseline)*

^§^ *statistically significant difference within groups (from 4 months)*

| Mean 4 months after baseline | | |
| --- | --- | --- |
| Running group  - Body weight (kg): 70.2 (SE 2.9)  - BMI (kg/m^2^): 25.1 (SE 1.0)  - Body fat (%): 32.2 (SE 2.3)  - Body fat mass (kg): 23.35 (SE 2.54)  - Android fat mass (%): 37.8 (SE 3.0)  - Gynoid fat mass (%): 43.0 (SE 1.9)  - Lean body mass – total (kg): 43.3 (SE 1.0)^‡^  - Lean body mass – legs (kg): 14.6 (SE 0.2)  - Body mineral density – total (g/cm^2^): 1.159 (SE 0.023)  - Body mineral density – legs (g/cm^2^): 1.235 (SE 0.019)  - Bone mineral content – total (kg): 2.51 (SE 0.01)  - Bone mineral content – legs (kg): 0.92 (SE 0.00) | Football group  - Body weight (kg): 72.5 (SE 3.4)  - BMI (kg/m^2^): 24.9 (SE 1.2)  - Body fat (%): 32.1 (SE 1.7)^‡^  - Body fat mass (kg): 23.00 (SE 1.99)  - Android fat mass (%): 35.2 (SE 2.7)  - Gynoid fat mass (%): 42.6 (SE 1.4)  - Lean body mass – total (kg): 45.2 (SE 2.0)^‡^  - Lean body mass – legs (kg): 16.4 (SE 0.6)^‡^  - Body mineral density – total (g/cm^2^): 1.206 (SE 0.026)^‡^  - Body mineral density – legs (g/cm^2^): 1.339 (SE 0.036)  - Bone mineral content – total (kg): 2.76 (SE 0.11)  - Bone mineral content – legs (kg): 1.06 (SE 0.03) | Control group  - Body weight (kg): 68.0 (SE 4.4)  - BMI (kg/m^2^): 23.5 (SE 1.5)  - Body fat (%): 29.0 (SE 3.2)  - Body fat mass (kg): 20.81 (SE 3.45)  - Android fat mass (%): 30.2 (SE 4.7)  - Gynoid fat mass (%): 39.1 (SE 2.7)  - Lean body mass – total (kg): 45.4 (SE 1.3)  - Lean body mass – legs (kg): 14.7 (SE 0.6)  - Body mineral density – total (g/cm^2^): 1.207 (SE 0.042)  - Body mineral density – legs (g/cm^2^): 1.321 (SE 0.038)  - Bone mineral content – total (kg): 2.69 (SE 0.13)  - Bone mineral content – legs (kg): 1.03 (SE 0.05) |
| Mean 16 months after baseline | | |
| Running group  - Body weight (kg): 68.4 (SE 2.4)  - BMI (kg/m^2^): 24.4 (SE 0.8)  - Body fat (%): 33.0 (SE 2.7)  - Body fat mass (kg): 22.36 (SE 2.46)  - Android fat mass (%): 35.7 (SE 2.8)  - Gynoid fat mass (%): 41.4 (SE 2.5)  - Lean body mass – total (kg): 42.3 (SE 1.2)^§^  - Lean body mass – legs (kg): 14.5 (SE 0.5)  - Body mineral density – total (g/cm^2^): 1.161 (SE 0.022)  - Body mineral density – legs (g/cm^2^): 1.258 (SE 0.013)^‡§^  - Bone mineral content – total (kg): 2.69 (SE 0.13)  - Bone mineral content – legs (kg): 0.95 (SE 0.02)^§^ | Football group  - Body weight (kg): 70.9 (SE 3.0)  - BMI (kg/m^2^): 24.3 (SE 1.2)  - Body fat (%): 32.7 (SE 1.9)  - Body fat mass (kg): 23.32 (SE 2.08)  - Android fat mass (%): 34.5 (SE 3.1)  - Gynoid fat mass (%): 41.6 (SE 1.7)^‡^  - Lean body mass – total (kg): 44.4 (SE 1.7)^‡^  - Lean body mass – legs (kg): 15.8 (SE 0.6)  - Body mineral density – total (g/cm^2^): 1.233 (SE 0.026)^‡§^  - Body mineral density – legs (g/cm^2^): 1.356 (SE 0.031)  - Bone mineral content – total (kg): 2.87 (SE 0.12)  - Bone mineral content – legs (kg): 1.09 (SE 0.04) | Control group  - Body weight (kg): 68.7 (SE 3.7)  - BMI (kg/m^2^): 23.8 (SE 1.3)  - Body fat (%): 28.6 (SE 2.5)  - Body fat mass (kg): 19.89 (SE 2.64)  - Android fat mass (%): 29.8 (SE 3.6)  - Gynoid fat mass (%): 39.3 (SE 2.1)  - Lean body mass – total (kg): 45.4 (SE 1.5)  - Lean body mass – legs (kg): 16.1 (SE 0.7)  - Body mineral density – total (g/cm^2^): 1.208 (SE 0.035)  - Body mineral density – legs (g/cm^2^): 1.330 (SE 0.036)  - Bone mineral content – total (kg): 2.72 (SE 0.13)  - Bone mineral content – legs (kg): 1.03 (SE 0.04) |

**Cardiorespiratory outcomes and between groups comparison**

** statistically significant difference between running group and control group*

^†^ *statistically significant difference between running group and football group*

^‡^ *statistically significant difference within groups (from baseline)*

^§^ *statistically significant difference within groups (from 4 months)*

*LVDD: left ventricular end-diastolic diameter*

*LVSD: left ventricular end-systolic diameter*

*LV_volume_: left ventricular end-diastolic volume*

*LVEF: left ventricular ejection fraction*

*IVSd: interventricular septum thickness in diastole*

*LVPWd: left ventricular posterior wall thickness in diastole*

*E: early diastole*

*A: late diastole*

*E’: early diastolic velocity*

*S’: peak systolic velocity*

*A’: late diastolic velocity*

*IVRT_global_: global isovulometric relaxation time*

*TT_index_: systolic longitudinal displacement of the left ventricle*

*RVDD: right ventricular end-diastolic diameter*

*TAPSE: tricuspid annular plane systolic excursion*

| Mean 4 months after baseline | | |
| --- | --- | --- |
| Running group  - VO_2_max (ml/min/kg): 37.7 (SE 2.1)^‡^  - Peak ventilation (l/min): 99 (SE 3)^‡^  - Resting heart rate (bpm): 59 (SE 2)  - Systolic blood pressure (mmHg): 106 (SE 4)  - Diastolic blood pressure (mmHg): 70 (SE 3)  - LVDD (mm): 46.2 (SE 1.2)  - LVSD (mm): 30.5 (SE 2.3)  - LV_volume_ (ml): 99 (SE 9)  - LVEF (%): 59 (SE 2)  - IVSd (mm): 7.8 (SE 0.5)  - LVPWd (mm): 7.8 (SE 0.5)  - E (m/s): 0.88 (SE 0.05)  - A (m/s): 0.49 (SE 0.04)  - E/A ratio: 1.86 (SE 0.20)  - E’ (m/s): 17.4 (SE 0.6)  - S’ (m/s): 11.0 (SE 0.5)^‡^  - A’ (m/s): 7.7 (SE 0.6)  - IVRT_global_ (ms): 57 (SE 4)  - S’ TDI_color_ (m/s): 6.3 (SE 0.2)  - TT_index_ (mm): 11.6 (SE 0.9)  - RVDD (ml): 26.5 (SE 0.6)  - TAPSE (mm): 25.0 (SE 1.5) | Football group  - VO_2_max (ml/min/kg): 39.8 (SE 2.2)^‡^  - Peak ventilation (l/min): 107 (SE 4)^‡^  - Resting heart rate (bpm): 57 (SE 2)^‡^  - Systolic blood pressure (mmHg): 111 (SE 3)  - Diastolic blood pressure (mmHg): 71 (SE 3)  - LVDD (mm): 48.6 (SE 1.8)  - LVSD (mm): 30.0 (SE 1.2)  - LV_volume_ (ml): 113 (SE 7)  - LVEF (%): 62 (SE 2)  - IVSd (mm): 8.3 (SE 0.6)  - LVPWd (mm): 9.0 (SE 0.7)  - E (m/s): 0.93 (SE 0.05)  - A (m/s): 0.46 (SE 0.03)^‡^  - E/A ratio: 2.14 (SE 0.32)^‡^  - E’ (m/s): 16.9 (SE 1.3)  - S’ (m/s): 10.6 (SE 0.9)^‡^  - A’ (m/s): 7.6 (SE 0.5)  - IVRT_global_ (ms): 50 (SE 5)^‡^  - S’ TDI_color_ (m/s): 6.3 (SE 0.4)^‡^  - TT_index_ (mm): 11.4 (SE 0.4)^‡^  - RVDD (ml): 29.9 (SE 1.4)  - TAPSE (mm): 23.9 (SE 0.8)^‡^ | Control group  - VO_2_max (ml/min/kg): 37.5 (SE 1.9)  - Peak ventilation (l/min): 94 (SE 6)  - Resting heart rate (bpm): 61 (SE 4)  - Systolic blood pressure (mmHg): 107 (SE 4)  - Diastolic blood pressure (mmHg): 63 (SE 3)  - LVDD (mm): 45.3 (SE 1.2)  - LVSD (mm): 30.3 (SE 2.9)  - LV_volume_ (ml): 105 (SE 12)  - LVEF (%): 57 (SE 1)  - IVSd (mm): 7.7 (SE 0.9)  - LVPWd (mm): 8.0 (SE 0.6)  - E (m/s): 0.75 (SE 0.05)  - A (m/s): 0.46 (SE 0.04)  - E/A ratio: 1.67 (SE 0.20)  - E’ (m/s): 17.7 (SE 2.2)  - S’ (m/s): 10.0 (SE 0.6)  - A’ (m/s): 8.7 (SE 1.8)^‡^  - IVRT_global_ (ms): 69 (SE 4)^‡^  - S’ TDI_color_ (m/s): 6.5 (SE 0.8)  - TT_index_ (mm): 11.6 (SE 0.4)  - RVDD (ml): 30.3 (SE 1.8)  - TAPSE (mm): 21.7 (SE 1.5) |
| Mean 16 months after baseline | | |
| Running group  - VO_2_max (ml/min/kg): 39.0 (SE 2.4)^‡^  - Peak ventilation (l/min): 103 (SE 3)  - Resting heart rate (bpm): 58 (SE 3)  - Systolic blood pressure (mmHg): 104 (SE 4)  - Diastolic blood pressure (mmHg): 69 (SE 3)  - LVDD (mm): 47.8 (SE 0.5)  - LVSD (mm): 32.0 (SE 1.9)  - LV_volume_ (ml): 96 (SE 8)  - LVEF (%): 60 (SE 2)  - IVSd (mm): 7.7 (SE 0.5)  - LVPWd (mm): 8.0 (SE 0.3)  - E (m/s): 0.85 (SE 0.05)  - A (m/s): 0.46 (SE 0.06)  - E/A ratio: 2.00 (SE 0.24)  - E’ (m/s): 17.2 (SE 0.9)  - S’ (m/s): 10.8 (SE 0.5)^‡^  - A’ (m/s): 9.5 (SE 0.8)  - IVRT_global_ (ms): 52 (SE 4)  - S’ TDI_color_ (m/s): 6.5 (SE 0.3)  - TT_index_ (mm): 11.3 (SE 1.0)  - RVDD (ml): 26.8 (SE 1.2)  - TAPSE (mm): 24.0 (SE 1.2) | Football group  - VO_2_max (ml/min/kg): 39.1 (SE 1.9)^‡^  - Peak ventilation (l/min): 101 (SE 4)^§^  - Resting heart rate (bpm): 54 (SE 2)^‡^  - Systolic blood pressure (mmHg): 111 (SE 3)  - Diastolic blood pressure (mmHg): 71 (SE 4)  - LVDD (mm): 51.3 (SE 1.3)^‡§^  - LVSD (mm): 30.9 (SE 1.7)  - LV_volume_ (ml): 114 (SE 7)  - LVEF (%): 63 (SE 2)  - IVSd (mm): 8.4 (SE 0.5)  - LVPWd (mm): 8.4 (SE 0.4)  - E (m/s): 0.89 (SE 0.09)  - A (m/s): 0.43 (SE 0.02)^‡^  - E/A ratio: 2.08 (SE 0.23)^‡^  - E’ (m/s): 18.0 (SE 1.5)  - S’ (m/s): 10.6 (SE 0.7)^‡^  - A’ (m/s): 9.1 (SE 0.9)  - IVRT_global_ (ms): 53 (SE 3)^‡^  - S’ TDI_color_ (m/s): 6.1 (SE 0.4)  - TT_index_ (mm): 11.7 (SE 0.5)^‡^  - RVDD (ml): 33.3 (SE 1.7)^‡^  - TAPSE (mm): 27.3 (SE 1.2)^‡§^ | Control group  - VO_2_max (ml/min/kg): 37.3 (SE 2.1)  - Peak ventilation (l/min): 91 (SE 8)  - Resting heart rate (bpm): 60 (SE 4)  - Systolic blood pressure (mmHg): 103 (SE 3)  - Diastolic blood pressure (mmHg): 63 (SE 3)  - LVDD (mm): 48.0 (SE 2.1)  - LVSD (mm): 32.7 (SE 0.9)  - LV_volume_ (ml): 104 (SE 15)  - LVEF (%): 55 (SE 2)  - IVSd (mm): 8.7 (SE 0.7)  - LVPWd (mm): 8.0 (SE 0.1)  - E (m/s): 0.67 (SE 0.10)  - A (m/s): 0.44 (SE 0.08)  - E/A ratio: 1.70 (SE 0.49)  - E’ (m/s): 17.3 (SE 1.2)  - S’ (m/s): 11.7 (SE 1.2)  - A’ (m/s): 8.3 (SE 1.5)^‡^  - IVRT_global_ (ms): 62 (SE 3)^§^  - S’ TDI_color_ (m/s): 6.5 (SE 0.5)  - TT_index_ (mm): 13.0 (SE 0.3)  - RVDD (ml): 31.0 (SE 1.0)  - TAPSE (mm): 24.3 (SE 0.3) |

**Blood serum concentration outcomes and between groups comparison**

** statistically significant difference between running group and control group*

^†^ *statistically significant difference between running group and football group*

^‡^ *statistically significant difference within groups (from baseline)*

^§^ *statistically significant difference within groups (from 4 months)*

| Mean 4 months after baseline | | |
| --- | --- | --- |
| Running group  - Fasting glucose ABL (mmol/l): 5.26 (SE 0.14)  - Fasting glucose YSI (mmom/l): 5.24 (SE 0.31)  - Blood lactate at 6.5 km/h (mmol/l): 1.01 (SE 0.12)  - Blood lactate at 8.0 km/h (mmol/l): 2.12 (SE 0.21) | Football group  - Fasting glucose ABL (mmol/l): 5.19 (SE 0.13)  - Fasting glucose YSI (mmom/l): 5.76 (SE 0.28)  - Blood lactate at 6.5 km/h (mmol/l): 0.92 (SE 0.11)^‡^  - Blood lactate at 8.0 km/h (mmol/l): 2.58 (SE 0.53)^‡^ | Control group  - Fasting glucose ABL (mmol/l): 5.30 (SE 0.14)  - Fasting glucose YSI (mmom/l): 5.45 (SE 0.28)  - Blood lactate at 6.5 km/h (mmol/l): 1.47 (SE 0.33)  - Blood lactate at 8.0 km/h (mmol/l): 2.42 (SE 0.43) |
| Mean 16 months after baseline | | |
| Running group  - Fasting glucose ABL (mmol/l): 5.09 (SE 0.29)  - Fasting glucose YSI (mmol/l): 5.53 (SE 0.29)  - Blood lactate at 6.5 km/h (mmol/l): 1.37 (SE 0.21)^‡§^  - Blood lactate at 8.0 km/h (mmol/l): 2.50 (SE 0.51) | Football group  - Fasting glucose ABL (mmol/l): 5.07 (SE 0.28)  - Fasting glucose YSI (mmol/l): 6.03 (SE 0.38)  - Blood lactate at 6.5 km/h (mmol/l): 0.80 (SE 0.12)^‡^  - Blood lactate at 8.0 km/h (mmol/l): 1.45 (SE 0.04)^‡^ | Control group  - Fasting glucose ABL (mmol/l): 5.16 (SE 0.04)  - Fasting glucose YSI (mmol/l): 5.60 (SE 0.29)  - Blood lactate at 6.5 km/h (mmol/l): 1.37 (SE 0.40)  - Blood lactate at 8.0 km/h (mmol/l): 2.62 (SE 0.42) |

**More publications related to this study**

| Andersen LJ, Hansen PR, Sogaard P, Madsen JK, Bech J, Krustrup P. Improvement of systolic and diastolic heart function after physical training in sedentary women. Scandinavian journal of medicine & science in sports. 2010;20 Suppl 1:50-7. |
| --- |

**Reference**

| Nindl BC, Alemany JA, Tuckow AP, Rarick KR, Staab JS, Kraemer WJ, et al. Circulating bioactive and immunoreactive IGF-I remain stable in women, despite physical fitness improvements after 8 weeks of resistance, aerobic, and combined exercise training. Journal of applied physiology. 2010;109(1):112-20. |
| --- |

**Objectives**

| 1- To determine the extent to which an insulin-like growth factor-I (IGF-I) bisphasic response pattern could be observed in three 8-wk exercise training programs consisting of different metabolic and neuromuscular demands;  2- To assess aspects of the circulating IGF-I system: total immunoreactive IGF-I, free immunoreactive IGF-I, bioactive IGF-I, IGF-binding proteins (IGFBP)-1, -2, and -3, and the proportion of IGFBP-1 complexed to IGF-I. |
| --- |

**Description of participants included in the study**

| - Participants randomised: n=58 women who had not been participating in any structured physical training program more than 2 times/week for the preceding 6 months.  - Source: university community.  - Participants included in the analysis, n=56 , mean age of 20.2 years (SE 2.1):   - Endurance exercise (running) group: n=13. - Resistance exercise group: n=18. - Combined resistance and aerobic exercise group: n=15. - Control group: n=10. |
| --- |

**Follow-up period and time-point assessments after baseline**

| - Follow-up: 12 weeks.  - Time-points:   - Baseline and 12 weeks after baseline: VO_2_max. - Baseline, 6 and 12 weeks after baseline: IGF-I system components and IGF-binding proteins. |
| --- |
| p.s.: The results corresponding to follow-up after 6 weeks from baseline were not extracted because 8 weeks or more of running training is an inclusion criterion of the systematic review. |

**Intervention (running program)**

| Endurance exercise (running) group  - Duration: 12 weeks.  - Frequency: 3 times/week.  - Description: running-based endurance exercise sessions on 3 alternating days/week. Endurance sessions were periodized between continuous running and sprint-type interval training on different days. All sessions began with a 5-10 min warm-up that included light jogging and dynamic range of motion exercises before training and ended with a 5-10 min cool-down. The weekly training was composed of 20–30 min of continuous running at a prescribed target of 70–85% of maximum heart rate, or interval running consisting of 400, 800, 1200, and 1600 m runs conducted close to maximal intensity with a 1:1 recovery time. This program was designed specifically to improve 3.2-km run time. |
| --- |

**Comparison group**

| Resistance group  - Duration: 12 weeks.  - Frequency: 3 times/week.  - Description: participants trained on 3 alternating days/week, and each session lasted approximately 60 min. Following a 2-week pretesting and familiarization period, the initial training (weeks 3–5) consisted of “light” days using 12-repetition maximum (RM) loads, “moderate” days using 8-10 RM loads, and “heavy” days using 6-8 RM loads. During weeks 6–12, “light” days utilized 12 RM loads, “moderate” days utilized 6-8 RM loads, and “heavy” days utilized 3-5 RM loads. | Combined group  - Duration: 12 weeks.  - Frequency: 3 times/week.  - Description: participants performed both the aerobic and the resistance exercise programs on the same day, during the same session, and exercised 3 alternating days/week utilizing the same programs described in the endurance exercise (running) group and the resistance group. Light resistance training days always corresponded to the interval sprint days to not limit the intensity of the resistance training session. Resistance training sessions were performed first and were immediately followed by the aerobic training session. | Control group  - Participants did not undergo any formalized physical training while maintaining their current activity levels. |
| --- | --- | --- |

**Body composition outcomes and between groups comparison**

** statistically significant difference between groups*

^‡^ *statistically significant difference within groups (pre-post)*

| The body weight (kg) and fat-free mass (kg) results were not described by each group, however there were not statistically significant difference between them. |
| --- |

**Cardiorespiratory outcomes and between groups comparison**

** statistically significant difference between groups*

^‡^ *statistically significant difference within groups (pre-post)*

| Mean 12 weeks after baseline | | | |
| --- | --- | --- | --- |
| Running group  - VO_2_peak (ml/kg/min): 42.4 (SE 1.4)^‡^ | Resistance group  - VO_2_peak (ml/kg/min): 38.9 (SE 1.1) | Combined group  - VO_2_peak (ml/kg/min): 41.5 (SE 1.2)^‡^ | Control group  - VO_2_peak (ml/kg/min): 38.3 (SE 1.4) |

**Blood serum concentration outcomes and between groups comparison**

** statistically significant difference between groups*

^‡^ *statistically significant difference within groups (pre-post)*

*IGF-I: total insulin-like growth factor I*

*IGFBP-1, 2 and 3: insulin-like growth factor binding proteins 1, 2 and 3.*

| Mean 12 weeks after baseline | | | |
| --- | --- | --- | --- |
| Running group  - Total IGF-I (µg/l): 250.2 (SE 17.0)  - Bioactive IGF-I (µg/l): 2.3 (SE 0.3)  - Free IGF-I (µg/l): 0.6 (SE 0.2)  - IGFBP-1 (µg/l): 64.9 (SE 11.6)  - Binary IGFBP-1 (µg/l): 26.9 (SE 5.2)  - IGFBP-1 saturation (%): 28.6 (SE 4.2)  - IGFBP-2 (µg/l): 105.0 (SE 15.6)  - IGFBP-3 (µg/l): 4.6 (SE 0.2) | Resistance group  - Total IGF-I (µg/l): 291.3 (SE 15.0)  - Bioactive IGF-I (µg/l): 2.5 (SE 0.3)  - Free IGF-I (µg/l): 0.5 (SE 0.2)  - IGFBP-1 (µg/l): 60.0 (SE 10.1)  - Binary IGFBP-1 (µg/l): 23.3 (SE 4.9)  - IGFBP-1 saturation (%): 36.4 (SE 3.8)  - IGFBP-2 (µg/l): 101.8 (SE 13.7)  - IGFBP-3 (µg/l): 4.9 (SE 0.2) | Combined group  - Total IGF-I (µg/l): 276.1 (SE 15.0)  - Bioactive IGF-I (µg/l): 2.5 (SE 0.3)  - Free IGF-I (µg/l): 0.8 (SE 0.1)  - IGFBP-1 (µg/l): 70.2 (SE 10.1)  - Binary IGFBP-1 (µg/l): 15.4 (SE 4.7)  - IGFBP-1 saturation (%): 24.2 (SE 3.8)  - IGFBP-2 (µg/l): 94.2 (SE 13.7)  - IGFBP-3 (µg/l): 4.7 (SE 0.1) | Control group  - Total IGF-I (µg/l): 269.0 (SE 20.4)  - Bioactive IGF-I (µg/l): 2.0 (SE 0.3)  - Free IGF-I (µg/l): 0.6 (SE 0.2)  - IGFBP-1 (µg/l): 84.7 (SE 13.9)  - Binary IGFBP-1 (µg/l): 15.8 (SE 7.0)  - IGFBP-1 saturation (%): 21.3 (SE 5.5)  - IGFBP-2 (µg/l): 69.0 (SE 18.8)  - IGFBP-3 (µg/l): 4.7 (SE 0.2) |

**More publications related to this study**

| Hendrickson NR, Sharp MA, Alemany JA, Walker LA, Harman EA, Spiering BA, et al. Combined resistance and endurance training improves physical capacity and performance on tactical occupational tasks. European journal of applied physiology. 2010;109(6):1197-208. |
| --- |

**Reference**

| Ozdemir RA, Celik O, Asci FH. Exercise interventions and their effects on physical self-perceptions of male university students. International journal of psychology : Journal international de psychologie. 2010;45(3):174-81. |
| --- |

**Objectives**

| 1- To examine the effects of 12-week exercise interventions on physical self-perceptions of male university students.  2- To investigate the magnitude of associations between the changes in physical self-perceptions and the changes in related physiological parameters after 12 weeks of exercise intervention programs. |
| --- |

**Description of participants included in the study**

| - Participants randomised: 48 male university students (aged from 19-25 years) who had no regular exercise background.  - Participants included in the analysis, n=46:   - Running group: n=12. - Cycling group: n=11. - Swimming group: n=11. - Control group: n=12. |
| --- |

**Follow-up period and time-point assessments after baseline**

| - Follow-up: 12 weeks.  - Time-points: baseline and 12 weeks after baseline. |
| --- |

**Intervention (running program)**

| Running group  - Duration: 12 weeks.  - Frequency: 3 times/week.  - Description: each session started with a 5 min warm-up, continuing with a main set for 30 min at their individual target heart rate zone (60-70% of heart rate reserve), and finished with a 5 min cool-down period. Sessions were performed on the treadmill with 1.5% incline, while the speed of the treadmill was determined according to individual heart rate zone. Throughout the 12 weeks, heart rate levels were held constant (60–70%), whereas speed of the treadmill was increased according to individual progression. |
| --- |

**Comparison group**

| Cycling group  - Duration: 12 weeks.  - Frequency: 3 times/week.  - Descriptions: each session started with a 5 min warm-up, continuing with a main set for 30 min at their individual target heart rate zone (60-70% of heart rate reserve), and finished with a 5 min cool-down period. Cyclists had to continue exercise between 60–80 repetitions per minute, and resistance of cycle ergometers was adjusted individually to keep each participant in his target heart rate zone. | Swimming group  - Duration: 12 weeks.  - Frequency: 3 times/week.  - Descriptions: each session started with a 5 min warm-up, continuing with a main set for 30 min at their individual target heart rate zone (60-70% of heart rate reserve), and finished with a 5 min cool-down period. Exercises were composed of the front crawl swimming and kick drills. Swimmers had at least beginner-level experience. | Control group  - Description: participants did not participate in any organized or structured exercise and to continue their daily activities. |
| --- | --- | --- |

**Body composition outcomes and between groups comparison**

** statistically significant difference between groups*

*^‡^ statistically significant difference within groups (pre-post)*

| Mean 12 weeks after baseline | | | |
| --- | --- | --- | --- |
| Running group  - Body fat (%): 16.5 (SD 5.9) | Cycling group  - Body fat (%): 16.4 (SD 4.3) | Swimming group  - Body fat (%): 20.1 (SD 5.1) | Control group  - Body fat (%): 20.2 (SD 3.0) |

**Cardiorespiratory outcomes and between groups comparison**

** statistically significant difference between running group and control group*

^†^ *statistically significant difference between running group and swimming group*

*^‡^ statistically significant difference within groups (pre-post)*

| Mean 12 weeks after baseline | | | |
| --- | --- | --- | --- |
| Running group  - VO_2_max (ml/kg/min): 50.6 (SD 5.8)^†^ | Cycling group  - VO_2_max (ml/kg/min): 50.0 (SD 4.9) | Swimming group  - VO_2_max (ml/kg/min): 51.1 (SD 4.3) | Control group  - VO_2_max (ml/kg/min): 48.8 (SD 3.3) |

**Blood serum concentration outcomes and between groups comparison**

** statistically significant difference between groups*

*^‡^ statistically significant difference within groups (pre-post)*

| Not applicable | Not applicable | Not applicable | Not applicable |
| --- | --- | --- | --- |

**Reference**

| Sedlock DA, Lee MG, Flynn MG, Park KS, Kamimori GH. Excess postexercise oxygen consumption after aerobic exercise training. International journal of sport nutrition and exercise metabolism. 2010;20(4):336-49. |
| --- |

**Objectives**

| To examine the effects of endurance exercise training on excess post-exercise oxygen consumption (EPOC). |
| --- |

**Description of participants included in the study**

| - Participants randomised: n=20 men not engaged in any systematic exercise training.  - Participants included in the analysis, n=19:   - Running group: n=9, mean age of 26.2 years (SE 1.4). - Control group: n=10, mean age of 26.2 years (SE 0.9). |
| --- |

**Follow-up period and time-point assessments after baseline**

| - Follow-up: 12 weeks  - Time-points: baseline and 12 weeks after baseline. |
| --- |

**Intervention (running program)**

| Running group  - Duration: 12 weeks.  - Frequency: 3-4 times/week.  - Description: each training session consisted of 10 min of warm-up, the main exercise (jogging/running) performed at an individually prescribed duration and intensity, and 10 min of cool-down. Frequency, intensity, and duration initially were 3 days/week at 60% VO_2_max for 25 min/session (~230–280 kcal/session). Training was gradually increased. By the 12^th^ week participants were exercising 4 days/week at an intensity eliciting 80% VO_2_max for 40 min/session (~520–580 kcal/session). |
| --- |

**Comparison group**

| Control group  - Participants did not engage in exercise training, and were asked to maintain their normal daily physical activity and dietary behaviours. |
| --- |

**Body composition outcomes and between groups comparison**

** statistically significant difference between groups*

*^‡^ statistically significant difference within groups (pre-post)*

| Mean 12 weeks after baseline | |
| --- | --- |
| Running group  - Body weight (kg): 73.3 (SE 2.0)  - Body fat (%): 15.2 (SE 1.8)*^‡^  - Body fat mass (kg): 11.2 (SE 1.4)*^‡^  - Body fat-free mass (kg): 62.1 (SE 2.0)* | Comparison group  - Body weight (kg): 71.3 (SE 2.6)  - Body fat (%): 18.1 (SE 1.0)  - Body fat mass (kg): 13.1 (SE 1.1)  - Body fat-free mass (kg): 58.2 (SE 1.7) |

**Cardiorespiratory outcomes and between groups comparison**

** statistically significant difference between groups*

*^‡^ statistically significant difference within groups (pre-post)*

*EPOC: excess post-exercise oxygen consumption*

*RER: net respiratory-exchange ratio*

| Mean 12 weeks after baseline | |
| --- | --- |
| Running group  - Exercise heart rate (bpm): results reported in a graph^‡^  - VO_2_max (l/min): 3.73 (SE 0.11)*^‡^  - VO_2_max (ml/kg/min): 51.0 (SE 1.3)*^‡^  - VO_2_max (ml/kg fat-free mass/min): 60.2 (SE 0.9)*^‡^  - EPOC (kcal/2h): 31.7 (SE 2.2)^‡^  - EPOC (kcal/kg): 0.43 (SE 0.03)^‡^  - EPOC (kcal/kg fat-free mass): 0.51 (SE 0.03)^‡^  - RER (VCO_2_/VO_2_): result reported in a graph*^‡^*  - RER (VO_2_/VCO_2_): result reported in a graph*^‡^* | Comparison group  - Exercise heart rate (bpm): results reported in a graph  - VO_2_max (l/min): 3.16 (SE 0.12)  - VO_2_max (ml/kg/min): 44.5 (SE 1.3)  - VO_2_max (ml/kg fat-free mass/min): 54.3 (SE 1.2)  - EPOC (kcal/2h): 38.6 (SE 3.1)  - EPOC (kcal/kg): 0.54 (SE 0.03)  - EPOC (kcal/kg fat-free mass): 0.66 (SE 0.04)  - RER (VCO_2_/VO_2_): result reported in a graph  - RER (VO_2_/VCO_2_): result reported in a graph |

**Blood serum concentration outcomes and between groups comparison**

** statistically significant difference between groups*

*^‡^ statistically significant difference within groups (pre-post)*

| Mean 12 weeks after baseline | |
| --- | --- |
| Running group  - Epinephrine (pg/ml): results reported in a graph^‡^  - Norepinephrine (pg/ml): results reported in a graph  - Blood lactate (mmol/l): results reported in a graph^‡^  - Free fat acid at end of exercise (mmol/l): 0.81 (SE 0.14)^‡^  - Free fat acid 30 min post-exercise (mmol/l): 0.94 (SE 0.11)^‡^  - Insulin (µU/ml): results reported in a graph^‡^  - Glucose (mmol/l): results reported in a graph^‡^ | Comparison group  - Epinephrine (pg/ml): results reported in a graph  - Norepinephrine (pg/ml): results reported in a graph  - Bool lactate (mmol/l): results reported in a graph  - Free fat acid at end of exercise (mmol/l): results reported in a graph  - Free fat acid 30 min post-exercise (mmol/l): results reported in a graph  - Insulin (µU/ml): results reported in a graph  - Glucose (mmol/l): results reported in a graph |

**More publications related to this study**

| Lee MG, Sedlock DA, Flynn MG, Kamimori GH. Resting metabolic rate after endurance exercise training. Medicine and science in sports and exercise. 2009;41(7):1444-51. |
| --- |

**Reference**

| Lee MG, Sedlock DA, Flynn MG, Kamimori GH. Resting metabolic rate after endurance exercise training. Medicine and science in sports and exercise. 2009;41(7):1444-51. |
| --- |

**Objectives**

| 1- To examine the effect of a 12-wk endurance exercise training program on resting metabolic rate;  2- To provide insight into the mechanisms responsible for alterations in resting metabolic rate that may occur after exercise training. |
| --- |

**Description of participants included in the study**

| - Participants randomised: n=20 male volunteers who were not engaged in any systematic training.  - Participants included in the analysis, n=19:   - Running group: n=9, mean age of 26.2 years (SE 1.4). - Control group: n= 10, mean age of 26.2 years (SE 0.9). |
| --- |

**Follow-up period and time-point assessments after baseline**

| - Follow-up: 12 weeks.  - Time-points: baseline and 12 weeks after baseline. |
| --- |

**Intervention (running program)**

| - Running group  - Duration: 12 weeks.  - Frequency: 3-4 times/week.  - Description: Each session consisted of 10 min warm-up, jogging and/or running and a 5 to 10 min cool-down. Initially, participants trained 3 times/week at an intensity calculated to elicit 60% VO_2_max for 25 min per session. Training was gradually increased, so that by the 12^th^ week, participants were exercising 4 times/week at 80% VO_2_max for 40 min per session. |
| --- |

**Comparison group**

| Control group  - Participants were asked to maintain their normal activity patterns. |
| --- |

**Body composition outcomes and between groups comparison**

** statistically significant difference between groups*

^‡^ *statistically significant difference within groups (pre-post)*

| Mean 12 weeks after baseline | |
| --- | --- |
| Running group  - Body weight (kg): 73.3 (SE 2.0)  - Body fat (%): 15.2 (SE 1.8)*^‡^  - Fat weight (kg): 11.2 (SE 1.4)*^‡^  - Fat-free weight (kg): 62.1 (SE 2.0) | Control group  - Body weight (kg): 71.3 (SE 2.6)  - Body fat (%): 18.1 (SE 1.0)  - Fat weight (kg): 13.1 (SE 1.1)  - Fat-free weight (kg): 58.2 (SE 1.7) |

**Cardiorespiratory outcomes and between groups comparison**

** statistically significant difference between groups*

^‡^ *statistically significant difference within groups (pre-post)*

*BW: body weight*

*FFW: fat-free weight*

*FW: fat weight*

*RMR: resting metabolic rate*

*RER: respiratory exchange ratio*

| Mean 12 weeks after baseline | |
| --- | --- |
| Running group  - Resting heart rate (bpm): 51.2 (SE 1.9)*  - VO_2_max (l/min): 3.73 (SE 0.11)*  - VO_2_max (ml/kgBW/min): 51.0 (SE 1.3)*  - VO_2_max (ml/kgFFW/min): 60.2 (SE 0.9)*  - RMR (l/min): 0.22 (SE 0.01)*  - RMR (ml/kg/min): 3.04 (SE 0.08)*  - RMR (kcal/min): 1.09 (SE 0.04)  - RMR (kJ/min): 4.55 (SE 0.16)  - RMR (kJ/kgBW/h): 3.73 (SE 0.09)  - RMR (kJ/kgFFW/h): 4.41 (SE 0.12)  - RMR (kJ/kgFW/h): 28.16 (SE 4.12)*  - RER: 0.87 (SE 0.02) | Control group  - Resting heart rate (bpm): 57.7 (SE 1.3)  - VO_2_max (l/min): 3.16 (SE 0.12)  - VO_2_max (ml/kg/min): 44.5 (SE 1.3)  - VO_2_max (ml/kgFFW/min): 54.3 (SE 1.2)  - RMR (l/min): 0.21 (SE 0.01)^‡^  - RMR (ml/kg/min): 2.94 (SE 0.06)^‡^  - RMR (kcal/min): 1.03 (SE 0.03)^‡^  - RMR (kJ/min): 4.31 (SE 0.14)^‡^  - RMR (kJ/kgBW/h): 3.64 (SE 0.08)^‡^  - RMR (kJ/kgFFW/h): 4.44 (SE 0.06)^‡^  - RMR (kJ/kgFW/h): 20.89 (SE 1.70)^‡^  - RER: 0.90 (SE 0.01) |

**Blood serum concentration outcomes and between groups comparison**

** statistically significant difference between groups*

^‡^ *statistically significant difference within groups (pre-post)*

*HOMA-IR: homeostasis model assessment of insulin resistance*

| Mean 12 weeks after baseline | |
| --- | --- |
| Running group  - Epinephrine (pg/ml): 23.9 (SE 1.9)  - Norepinephrine (pg/ml): 202.3 (SE 19.0)  - Total thyroxine (nmol/l): 83.1 (SE 2.3)  - Free thyroxine (pmol/l): 13.8 (SE 0.5)^‡^  - Free fatty acids (mmol/l): 0.48 (SE 0.04)*^‡^  - Insulin (µIU/ml): 5.2 (SE 0.9)  - Glucose (mmol/l): 5.0 (SE 0.1)  - HOMA-IR: 1.18 (SE 0.22) | Control group  - Epinephrine (pg/ml): 18.7 (SE 1.6)  - Norepinephrine (pg/ml): 129.4 (SE 9.8)  - Total thyroxine (nmol/l): 89.2 (SE 4.2)  - Free thyroxine (pmol/l): 15.5 (SE 0.4)  - Free fatty acids (mmol/l): 0.41 (SE 0.04)  - Insulin (µIU/ml): 5.7 (SE 0.4)  - Glucose (mmol/l): 5.4 (SE 0.1)  - HOMA-IR: 1.38 (SE 0.10) |

**More publications related to this study**

| Sedlock DA, Lee MG, Flynn MG, Park KS, Kamimori GH. Excess postexercise oxygen consumption after aerobic exercise training. International journal of sport nutrition and exercise metabolism. 2010;20(4):336-49. |
| --- |

**Reference**

| Lester ME, Urso ML, Evans RK, Pierce JR, Spiering BA, Maresh CM, et al. Influence of exercise mode and osteogenic index on bone biomarker responses during short-term physical training. Bone. 2009;45(4):768-76. |
| --- |

**Objectives**

| 1- To determine whether three types of controlled, periodized eight week exercise programs could safely promote bone formation in young, healthy women.  2- To determine whether the osteogenic index associated with a particular program was predictive of changes in biochemical markers. |
| --- |

**Description of participants included in the study**

| - Participants randomised: n=69 young college women who were not participating in physical training activities more than 2 times/week in the preceding 6 months.  - Participants included in the analysis, n=58, mean age of 20.0 years (SD 1.7):   - Endurance exercise (running) group: n=15. - Resistance exercise group: n=17. - Combined resistance and aerobic exercise group: n=16. - Control group: n=10. |
| --- |

**Follow-up period and time-point assessments after baseline**

| - Follow-up: 8 weeks.  - Time-points: baseline, 4 and 8 weeks after baseline. |
| --- |
| p.s.: The results corresponding to follow-up after 4 weeks from baseline were not extracted because 8 weeks or more of running training is an inclusion criterion of the systematic review. |

**Intervention (running program)**

| Running group  - Duration: 8 weeks.  - Frequency: 3 times/week.  - Description: running training 3 alternating days/week with each exercise session lasting between 30 and 90 min. The training program contained continuous and interval exercises. Interval workouts had a 1:1 recovery time based on time to complete the interval. All sessions began and ended with a 5–10 min warm-up and cool down which included light jogging, calisthenics and stretching. |
| --- |

**Comparison group**

| Resistance group  - Duration: 8 weeks.  - Frequency: 3 times/week.  - Description: participants trained 3 alternating days/week with each exercise session lasting between 30 and 90 min, following a non-linear periodized model in which load and repetition were varied on a daily basis (light, moderate or heavy) in order to optimize adaptations. | Combined group  - Duration: 8 weeks.  - Frequency: 3 times/week.  - Description: participants performed the entire training regimen of both the aerobic and the resistance exercise programs during a single session on 3 alternating days/week. In order to maximize force production capabilities during resistance exercise without prior fatigue, the resistance exercise was performed first, followed by the aerobic exercise. | Control group  - Participants were asked o refrain from participating in any structured exercise program for the duration of the study. |
| --- | --- | --- |

**Body composition outcomes and between groups comparison**

** statistically significant difference between groups*

^‡^ *statistically significant difference within groups (pre-post)*

| Mean 8 weeks after baseline | | | |
| --- | --- | --- | --- |
| Running group  - Body weight (kg): 60.0 (SD 5.9)  - Body lean mass (kg): 41.0 (SD 3.8)  - Body fat mass (kg): 19.0 (SD 4.5) | Resistance group  - Body weight (kg): 62.6 (SD 7.9)  - Body lean mass (kg): 42.8 (SD 4.9)  - Body fat mass (kg): 19.8 (SD 5.6) | Combined group  - Body weight (kg): 64.5 (SD 11.9)  - Body lean mass (kg): 42.3 (SD 4.3)  - Body fat mass (kg): 22.2 (SD 9.1) | Control group  - Body weight (kg): 63.3 (SD 8.1)  - Body lean mass (kg): 42.5 (SD 2.2)  - Body fat mass (kg): 20.8 (SD 9.1) |

**Cardiorespiratory outcomes and between groups comparison**

** statistically significant difference between groups*

^‡^ *statistically significant difference within groups (pre-post)*

| Not applicable | Not applicable | Not applicable | Not applicable |
| --- | --- | --- | --- |

**Blood serum concentration outcomes and between groups comparison**

** statistically significant difference between groups*

^‡^ *statistically significant difference within groups (pre-post)*

*BAP: bone alkaline phosphatase*

*CTx: serum C-terminal telopeptide fragment of type I collagen*

*TRAP: tartrate-resistant acid phosphatase*

| Mean 8 weeks after baseline | | | |
| --- | --- | --- | --- |
| Running group  - Serum vitamin D concentration (nmol/l): result reported in a graph  - Parathyroid hormone concentration (pg/ml): result reported in a graph  - BAP (U/l): result reported in a graph  - Osteocalcin (ng/ml): result reported in a graph  - CTx (ng/ml): result reported in a graph  - TRAP (U/l): result reported in a graph^‡^  - Deoxypyridinoline (nmol/l): result reported in a graph | Resistance group  - Serum vitamin D concentration (nmol/l): result reported in a graph  - Parathyroid hormone concentration (pg/ml): result reported in a graph  - BAP (U/l): result reported in a graph^‡^  - Osteocalcin (ng/ml): result reported in a graph^‡^  - CTx (ng/ml): result reported in a graph  - TRAP (U/l): result reported in a graph^‡^  - Deoxypyridinoline (nmol/l): result reported in a graph | Combined group  - Serum vitamin D concentration (nmol/l): result reported in a graph  - Parathyroid hormone concentration (pg/ml): result reported in a graph  - BAP (U/l): result reported in a graph^‡^  - Osteocalcin (ng/ml): result reported in a graph^‡^  - CTx (ng/ml): result reported in a graph  - TRAP (U/l): result reported in a graph^‡^  - Deoxypyridinoline (nmol/l): result reported in a graph | Control group  - Serum vitamin D concentration (nmol/l): result reported in a graph  - Parathyroid hormone concentration (pg/ml): result reported in a graph  - BAP (U/l): result reported in a graph  - Osteocalcin (ng/ml): result reported in a graph  - CTx (ng/ml): result reported in a graph  - TRAP (U/l): result reported in a graph^‡^  - Deoxypyridinoline (nmol/l): result reported in a graph |

**More publications related to this study**

| Gregory SM, Spiering BA, Alemany JA, Tuckow AP, Rarick KR, Staab JS, et al. Exercise-induced insulin-like growth factor I system concentrations after training in women. Medicine and science in sports and exercise. 2013;45(3):420-8. |
| --- |

**Reference**

| Brixius K, Schoenberger S, Ladage D, Knigge H, Falkowski G, Hellmich M, et al. Long-term endurance exercise decreases antiangiogenic endostatin signalling in overweight men aged 50-60 years. British journal of sports medicine. 2008;42(2):126-9. |
| --- |

**Objectives**

| To investigate whether long-term physical activity may result in an increase in circulating serum levels of vascular endothelial growth factor or endostatin. |
| --- |

**Description of participants included in the study**

| - Participants randomised: n=21 overweight middle-aged men (BMI>26) who had not taken regular physical exercise before the study.  - Participants included in the analysis, n=21:   - Running group: n=7, mean age of 52.6 years (SE 1.1). - Cycling group: n=7, mean age of 58.9 years (SE 1.1). - Control group: n=7, mean age of 58.7 years (SE 1.1). |
| --- |

**Follow-up period and time-point assessments after baseline**

| - Follow-up: 6 months.  - Time-points: baseline and 6 months after baseline. |
| --- |

**Intervention (running program)**

| Running group  - Duration: 6 months.  - Frequency: 3 times/week.  - Description: running training for 60 min/session. Intensity was set at 2-4 mmol/l lactate. |
| --- |

**Comparison group**

| Cycling group  - Duration: 6 months.  - Frequency: 3 times/week.  - Description: cycling exercise for 90 min/session. Intensity was set at 2-4 mmol/l lactate. | Control group  - Non-active, sedentary group. |
| --- | --- |

**Body composition outcomes and between groups comparison**

** statistically significant difference between running group and control group*

^†^ *statistically significant difference between running group and cycling group*

^‡^ *statistically significant difference within groups (pre-post)*

| Mean 6 months after baseline | | |
| --- | --- | --- |
| Running group  - BMI (kg/m^2^): 29.1 (SE 0.6)^‡^ | Cycling group  - BMI (kg/m^2^): 30.1 (SE 0.9)^‡^ | Control group  - BMI (kg/m^2^): 30.1 (SE 0.7) |

**Cardiorespiratory outcomes and between groups comparison**

** statistically significant difference between running group and control group*

^†^ *statistically significant difference between running group and cycling group*

^‡^ *statistically significant difference within groups (pre-post)*

| Mean 6 months after baseline | | |
| --- | --- | --- |
| Running group  - Heart rate (bpm): 61.9 (SE 1.7)  - Systolic blood pressure (mmHg): 126.0 (SE 1.6)  - Diastolic blood pressure (mmHg): 82.4 (SE 1.4) | Cycling group  - Heart rate (bpm): 59.9 (SE 3.6)  - Systolic blood pressure (mmHg): 126.4 (SE 4.1)  - Diastolic blood pressure (mmHg): 95.7 (SE 3.1) | Control group  - Heart rate (bpm): 67.3 (SE 2.8)  - Systolic blood pressure (mmHg): 125.6 (SE 4.0)  - Diastolic blood pressure (mmHg): 81.5 (SE 2.3) |

**Blood serum concentration outcomes and between groups comparison**

** statistically significant difference between running group and control group*

^†^ *statistically significant difference between running group and cycling group*

^‡^ *statistically significant difference within groups (pre-post)*

*VEGF: vascular endothelial growth factor*

| Mean 6 months after baseline | | |
| --- | --- | --- |
| Running group  - VEGF (ng/ml): 1.5 (SE 0.2)  - Endostatin (ng/ml): 17.5 (SE reported in a graph)^‡^ | Cycling group  - VEGF (ng/ml): 1.5 (SE 0.2)  - Endostatin (ng/ml): 18.0 (SE reported in a graph)^‡^ | Control group  - VEGF (ng/ml): 2.1 (SE 0.7)  - Endostatin (ng/ml): 17.7 (SE reported in a graph) |
| Pre-post mean difference 6 months after baseline | | |
| Running group  - ΔEndostatin (ng/ml): 20.9 (SE 1.0)*^‡^ | Cycling group  - ΔEndostatin (ng/ml): 21.3 (SE 1.6)^‡^ | Control group  - ΔEndostatin (ng/ml): 19.7 (SE 1.1) |

**Reference**

| Meyer T, Auracher M, Heeg K, Urhausen A, Kindermann W. Effectiveness of low-intensity endurance training. International journal of sports medicine. 2007;28(1):33-9. |
| --- |

**Objectives**

| To compare two exercise intensities from the moderate to lower range for differences in their effectiveness. |
| --- |

**Description of participants included in the study**

| - Participants randomised: n=45 participants who had no regular endurance training within the last years and absence of activities with known endurance effects within the last 6 months.  - Participants included in the analysis, n=39:   - Moderate intensity exercise group: n=13, mean age of 44 years (SD 8), 7 males and 6 females. - Low intensity exercise group: n=13, mean age of 42 years (SD 7), 6 males and 7 females. - Control group: n=13, mean age of 46 years (SD 7), 7 males and 6 females. |
| --- |

**Follow-up period and time-point assessments after baseline**

| - Follow-up: 12 weeks.  - Time-points: baseline and 12 weeks after baseline. |
| --- |

**Intervention (running program)**

| Moderate intensity exercise group  - Duration: 12 weeks.  - Frequency: 5 times/week.  - Description: training was conducted on 5 freely chosen days/week. One session per week was supervised by one of the investigators. After 10 min warm-up (calisthenics), the program consisted of walking or running within the prescribed heart rate range for 30 min. Energy consumption was about 1400 kcal. | Low intensity exercise group  - Duration: 12 weeks.  - Frequency: 5 times/week.  - Description: training was conducted on 5 freely chosen days/week. One session per week was supervised by one of the investigators. After 10 min warm-up (calisthenics), the program consisted of walking or running within the prescribed heart rate range for 30 minutes + “x” min. “x” was calculated with the following equation whereby lengthening the duration in proportion to its lower oxygen uptake: x=30.(VO_2_mod/VO_2_low), with VO_2_mod and VO_2_low representing the VO_2_ during treadmill testing corresponding to the heart rate prescription in moderate (mod) and low groups, respectively. Energy consumption was about 1400 kcal. |
| --- | --- |

**Comparison group**

| Control group  - Unchanged activity. |
| --- |

**Body composition outcomes and between groups comparison**

** statistically significant difference between moderate intensity group and control group*

^†^ *statistically significant difference between low intensity group and control group*

^§^ *statistically significant difference between moderate intensity group and low intensity group*

^‡^ *statistically significant difference within groups (pre-post)*

| Mean 12 weeks after baseline | | |
| --- | --- | --- |
| Moderate intensity group  - Body weight (kg): 82.5 (SD 18.1) | Low intensity group  - Body weight (kg): 80.9 (SD 19.3) | Control group  - Body weight (kg): 78.7 (SD 18.5) |

**Cardiorespiratory outcomes and between groups comparison**

** statistically significant difference between moderate intensity group and control group*

^†^ *statistically significant difference between low intensity group and control group*

^§^ *statistically significant difference between moderate intensity group and low intensity group*

^‡^ *statistically significant difference within groups (pre-post)*

| Mean 12 weeks after baseline | | |
| --- | --- | --- |
| Moderate intensity group  - Resting heart rate (bpm): result reported in a graph  - VO_2_max (ml/kg/min): 39.5 (SD 10.9)  - VO_2_max (ml/⅔kg/min): 169 (SD 40)  - VO_2_ at the anaerobic threshold (ml/kg/min): 29.6 (SD 8.08)* | Low intensity group  - Resting heart rate (bpm): result reported in a graph  - VO_2_max (ml/kg/min): 37.7 (SD 7.2)^†^  - VO_2_max (ml/⅔kg/min): 163 (SD 35)  - VO_2_ at the anaerobic threshold (ml/kg/min): 25.2 (SD 5.1)^†^ | Control group  - Resting heart rate (bpm): result reported in a graph  - VO_2_max (ml/kg/min): 34.7 (SD 9.8)  - VO_2_max (ml/⅔kg/min): 147 (SD 42)  - VO_2_ at the anaerobic threshold (ml/kg/min): 26.6 (SD 7.0) |

**Blood serum concentration outcomes and between groups comparison**

** statistically significant difference between moderate intensity group and control group*

^†^ *statistically significant difference between low intensity group and control group*

^§^ *statistically significant difference between moderate intensity group and low intensity group*

^‡^ *statistically significant difference within groups (pre-post)*

| Mean 12 weeks after baseline | | |
| --- | --- | --- |
| Moderate intensity group  - Max lactate concentration (mmol/l): 7.5 (SD 1.8) | Low intensity group  - Max lactate concentration (mmol/l): 8.6 (SD 2.4) | Control group  - Max lactate concentration (mmol/l): 7.3 (SD 2.0) |

**Reference**

| Ring-Dimitriou S, von Duvillard SP, Paulweber B, Stadlmann M, Lemura LM, Peak K, et al. Nine months aerobic fitness induced changes on blood lipids and lipoproteins in untrained subjects versus controls. European journal of applied physiology. 2007;99(3):291-9. |
| --- |

**Objectives**

| 1- To investigate if low intensity exercise resulting from 9 months of running training can change aerobic fitness and blood lipid concentration;  2- To investigate what magnitude of the effect in aerobic fitness can positively affect health-enhancing changes in selected blood lipids and lipoproteins. |
| --- |

**Description of participants included in the study**

| - Participants randomised: n=52 adults who had not been practising physical activities more than 2 h/week, and with a VO_2_peak ≤ 40.0 ml/kg/min.  - Source: adults recruited in local area (Salzburg, Austria).  - Participants included in the analysis, n=30:   - Running group: n=20, mean age of 39 years (SD 3.4), 9 males, 11 females. - Control group: n=10, mean age of 40.8 years (SD 5.8), 5 males, 5 females. |
| --- |

**Follow-up period and time-point assessments after baseline**

| - Follow-up: 9 months.  - Time-points: baseline and 9 months after baseline. |
| --- |

**Intervention (running program)**

| Running group  - Duration: 9 months.  - Frequency: not described.  - Aim: half-marathon or marathon at the end of the study.  - Description: running and stretching techniques provided by experienced personnel once a week. A total of 92% of total training volume was completed at an exercise intensity corresponding to a blood lactate concentration of ≤ 2.0 mmol/l. The weekly volume increased every 3 weeks:   - 3 months: 2.0 hours, 18.0 (SD=7.0) km/week. - 6 months: 2.5 hours, 23.0 (SD 10.0) km/week. - 9 months: 3.0 hours, 25.0 (SD 11.0) km/week.   Before the competition they participated in 2–3 running events to become familiar with the competition environment. |
| --- |

**Comparison group**

| Control group  - They were instructed to maintain their usual daily activity. None of the subjects participated in a structured or regular exercise program. |
| --- |

**Body composition outcomes and between groups comparison**

** statistically significant difference between groups*

*^‡^ statistically significant difference within groups (pre-post)*

| Pre-post mean differences 9 months after baseline | |
| --- | --- |
| Running group  - ΔBody weight (kg): -2.1 (95%CI -9.8 to 5.7)  - ΔBMI (kg/m^2^): -0.6 (95%CI -3.4 to 2.2)  - ΔBody fat (%): -1.4 (95%CI -6.1 to 3.3) | Control group  - ΔBody weight (kg): 0.4 (95%CI -15.1 to 15.9)  - ΔBMI (kg/m^2^): 0.2 (95%CI -3.2 to 3.5)  - ΔBody fat (%): -1.2 (95%CI -5.4 to 3.0) |

**Cardiorespiratory outcomes and between groups comparison**

** statistically significant difference between groups*

*^‡^ statistically significant difference within groups (pre-post)*

| Pre-post mean differences 9 months after baseline | |
| --- | --- |
| Running group  - ΔVO_2_peak (ml/kg/min): 8.6 (95%CI 5.0 to 12.2)**^‡^*  - Δ%VO_2_ at blood lactate concentration of 2.0 mmol/l (%): -0.3 (95%CI -0.9 to 8.3)  - Δ%VO_2_ at blood lactate concentration of 4.0 mmol/l (%): 1.1 (95%CI -9.0 to 11.2) | Control group  - ΔVO_2_peak (ml/kg/min): -2.8 (95%CI -7.6 to 2.1)  - Δ%VO_2_ at blood lactate concentration of 2.0 mmol/l (%): -3.4 (95%CI -12.9 to 6.0)  - Δ%VO_2_ at blood lactate concentration of 4.0 mmol/l (%): -2.7 (95%CI -10.8 to 5.4) |

**Blood serum concentration outcomes and between groups comparison**

** statistically significant difference between groups*

*^‡^ statistically significant difference within groups (pre-post)*

| Pre-post mean differences 9 months after baseline | |
| --- | --- |
| Running group  - ΔTotal cholesterol (mg/dl): -5.6 (95%CI -38.1 to 26.9)  - ΔLDL (mg/dl): -4.6 (95%CI -33.4 to 24.2)  - ΔHDL (mg/dl): -6.2 (95%CI -17.8 to 5.4)  - ΔTriglycerides (mg/dl): 1.9 (95%CI -34.5 to 38.4)  - ΔApolipoprotein B (mg/dl): -20.3 (95%CI -39.6 to -1.0)**^‡^*  - ΔApolipoprotein A-1 (mg/dl): 1.6 (95%CI -15.2 to 18.3)*  - ΔLipoprotein (a) (mg/dl): -0.1 (95%CI -12.8 to 12.7) | Control group  - ΔTotal cholesterol (mg/dl): -3.4 (95%CI -22.5 to 15.7)  - ΔLDL (mg/dl): 9.1 (95%CI -10.7 to 29.0)  - ΔHDL (mg/dl): -1.9 (95%CI -20.6 to 16.9)  - ΔTriglycerides (mg/dl): 0.3 (95%CI -77.7 to 78.2)  - ΔApolipoprotein B (mg/dl): -6.9 (95%CI -24.9 to 11.1)  - ΔApolipoprotein A-1 (mg/dl): -21.6 (95%CI -53.6 to 10.4)  - ΔLipoprotein (a) (mg/dl): 3.5 (95%CI -28.9 to 35.8) |

**Reference**

| Beneke R, Hutler M. The effect of training on running economy and performance in recreational athletes. Medicine and science in sports and exercise. 2005;37(10):1794-9. |
| --- |

**Objectives**

| To analyse the effect of an 8-week training program on the energy cost of running and the performance of recreational males. |
| --- |

**Description of participants included in the study**

| - Participants randomised: n=16 not physically active males who had not participated in any kind of running training for at least 2 years before the study commenced.  - Participants included in the analysis, n=16:   - Running group: n=8, mean of age of 25.3 years (SD 2.9). - Control group: n=8, mean of age of 24.3 years (SD 3.7). |
| --- |

**Follow-up period and time-point assessments after baseline**

| - Follow-up: 8 weeks.  - Time-points: baseline, 4 and 8 weeks after baseline. |
| --- |
| p.s.: The results were extracted only after 8 weeks from baseline because 8 weeks or more of running training is an inclusion criterion of the systematic review. |

**Intervention (running program)**

| Running group  - Duration: 8 weeks.  - Frequency: 3-5 times/week.  - Description: 3 running session in week 1, 4 running sessions in weeks 2-6 and 5 running sessions in weeks 7 and 8. Participants never completed more than one training session in a single day. During the initial 4 weeks, each training session lasted 20–30 min. The duration was increased to 45–60 min during weeks 5–8. Training intensity began at 50% of the heart rate reserve during week 1 and was progressively increased to an intensity of 60 –75% of the heart rate reserve during week 8. Most of the training was prolonged running at almost constant speeds. Only one training session/week contained repeated short-duration bouts of faster running up to 300 m distances. |
| --- |

**Comparison group**

| Control group  - Participants of this group did not train. |
| --- |

**Body composition outcomes and between groups comparison**

** statistically significant difference between groups*

^‡^ *statistically significant difference within groups (pre-post)*

| Not applicable | Not applicable |
| --- | --- |

**Cardiorespiratory outcomes and between groups comparison**

** statistically significant difference between groups*

^‡^ *statistically significant difference within groups (pre-post)*

*VO_2_def: oxygen deficit*

*VO_2_ss: oxygen uptake of the final 30 s*

| Mean 8 weeks after baseline | |
| --- | --- |
| Running group  - VO_2_ (l): 12.093 (SD 1.464)  - VO_2_def (l): 1.044 (SD 0.141)  - VO_2_ss (l/min): result reported in a graph  - Metabolic energy (kJ): 257.9 (SD 38.0)^‡^  - Energy cost of running (J/kg/m): 3.7 (SD 0.4)*^‡^ | Control group  - VO_2_ (l): 13.622 (SD 1.712)  - VO_2_def (l): 1.114 (SD 0.154)  - VO_2_ss (l/min): result reported in a graph  - Metabolic energy (kJ): 294.1 (SD 38.9)  - Energy cost of running (J/kg/m): 4.3 (SD 0.6) |

**Blood serum concentration outcomes and between groups comparison**

** statistically significant difference between groups*

^‡^ *statistically significant difference within groups (pre-post)*

*BLC: increase of blood lactate concentration*

| Mean 8 weeks after baseline | |
| --- | --- |
| Running group  - BLC (mmol/l): 1.2 (SD 0.4)*^‡^ | Control group  - BLC (mmol/l): 2.1 (SD 1.0) |

**Reference**

| Hautala AJ, Makikallio TH, Kiviniemi A, Laukkanen RT, Nissila S, Huikuri HV, et al. Heart rate dynamics after controlled training followed by a home-based exercise program. European journal of applied physiology. 2004;92(3):289-97. |
| --- |

**Objectives**

| To assess the effects of aerobic training on heart rate indices over a 24-h period after an intervention of 8 weeks of highly controlled aerobic training followed by home-based training for 10 months in a population of healthy males. |
| --- |

**Description of participants included in the study**

| - Participants randomised: n=24 men who did not participate in regular physical training more than twice a week.  - Participants included in the analysis, n=24:   - Running group: n=18, mean age of 39 years (SD 10). - Control group: n=6, mean age of 44 years (SD 6). |
| --- |

**Follow-up period and time-point assessments after baseline**

| - Follow-up: 1 year.  - Time-points: baseline, 8 weeks and 1 year after baseline. |
| --- |

**Intervention (running program)**

| Running group  - Duration: 8 weeks of controlled training and 10 months of home-based training.  - Frequency: daily basis.  - Description: walking and running highly controlled training for 8 weeks (5.8 sessions/week, SD 0.2), including six 30-60 min training sessions a week (45 min, SD 15) at an intensity of 70–80% (75%, SD 2) of maximum heart rate. After the 8 weeks of controlled training the participants were encouraged to continue aerobic training independently, preferably on a daily basis for 10 months at an intensity level of 70–80% of their individual maximum heart rate. The individual maximum heart rate was established based on the maximum heart rate achieved during the VO_2_peak test after 8 weeks of training. The recommended mode of training was jogging, but cross-country skiing, roller-skating, and ball games were also allowed if the target heart rate and a single training session duration of 30–60 min was achieved. |
| --- |

**Comparison group**

| Control group  - Not described. |
| --- |

**Body composition outcomes and between groups comparison**

** statistically significant difference between groups*

^‡^ *statistically significant difference within groups (pre-post)*

| Mean 8 weeks after baseline | |
| --- | --- |
| Running group  - Body weight (kg): 78.6 (SD 9.6)  - BMI (kg/m^2^): 24.4 (SD 2.0) | Comparison group  - Body weight (kg): 80.8 (SD 6.7)  - BMI (kg/m^2^): 25.5 (SD 2.3) |
| p.s.: The results between 8 weeks and 1 year of follow-up (10-month home-based training) were not extracted because these results reflected effects of mixed physical activities (there were nor running specific). | |

**Cardiorespiratory outcomes and between groups comparison**

** statistically significant difference between groups*

^‡^ *statistically significant difference within groups (pre-post)*

*SDNN: standard deviation of all R-R intervals*

| Mean 8 weeks after baseline | |
| --- | --- |
| Running group  - VO_2_peak (l/min): 3.6 (SD 0.5)^‡^  - VO_2_peak (ml/kg/min): 46 (SD 5)^‡^ | Control group  - VO_2_peak (l/min): 3.5 (SD 0.5)  - VO_2_peak (ml/kg/min): 44 (SD 5) |
| Pre-post mean difference 8 weeks after baseline | |
| Running group  - ΔVO_2_peak (%): 10.2 (SD 4.4)^‡^ | Control group  - ΔVO_2_peak (%): not reported |
| Mean variability indices over 24-h 8 weeks after baseline | |
| Running group  - Heart rate max (bpm): 64 (SD 6)^‡^  - SDNN (ms): 197 (SD 40)  - High frequency power (ln/ms^2^): 6.9 (SD 1.1)^‡^  - Low frequency power (ln/ms^2^): 7.8 (SD 0.7)^‡^  - Very low frequency power (ln/ms^2^): 8.2 (SD 0.6)^‡^  - Low/high frequency power ration: 2.7 (SD 1.2)^‡^ | Control group  - Heart rate max (bpm): 66 (SD 6)  - SDNN (ms): 198 (SD 36)  - High frequency power (ln/ms^2^): 6.5 (SD 1.1)  - Low frequency power (ln/ms^2^): 7.5 (SD 0.7)  - Very low frequency power (ln/ms^2^): 7.9 (SD 0.7)  - Low/high frequency power ration: 3.1 (SD 1.6) |
| Mean variability indices over night 8 weeks after baseline | |
| Running group  - Heart rate max (bpm): results reported in a graph^‡^  - High frequency power (ln/ms^2^): results reported in a graph^‡^  - Low frequency power (ln/ms^2^): results reported in a graph^‡^  - Low/high frequency power ration: results reported in a graph | Control group  - Heart rate max (bpm): not reported  - High frequency power (ln/ms^2^): not reported  - Low frequency power (ln/ms^2^): not reported  - Low/high frequency power ration: not reported |
| p.s.: the results between 8 weeks and 1 year of follow-up (10-month home-based training) were not extracted because these results reflected effects of mixed physical activities (there were nor running specific). | |

**Blood serum concentration outcomes and between groups comparison**

** statistically significant difference between groups*

^‡^ *statistically significant difference within groups (pre-post)*

| Not applicable | Not applicable |
| --- | --- |

**Reference**

| Poehlman ET, Dvorak RV, DeNino WF, Brochu M, Ades PA. Effects of resistance training and endurance training on insulin sensitivity in nonobese, young women: a controlled randomised trial. The Journal of clinical endocrinology and metabolism. 2000;85(7):2463-8. |
| --- |

**Objectives**

| To compare the effects of resistance training and aerobic training on insulin sensitivity in non-obese, younger women. |
| --- |

**Description of participants included in the study**

| - Participants randomised: n=78 non-obese (BMI < 26), younger women who have had no regular participation in exercise for 6 months before the study.  - Source: Burlington (Vermont) and the University of Vermont community, USA.  - Participants included in the analysis, n=51:   - Endurance (running) training group: n=14, mean age of 29 years (SD 5). - Resistance training group: n=17, mean age of 28 years (SD 3). - Control group: n=20, mean age of 28 years (SD 4). |
| --- |

**Follow-up period and time-point assessments after baseline**

| - Follow-up: 6 months.  - Time-points: baseline and 6 months after baseline. |
| --- |

**Intervention (running program)**

| Endurance (running) training group  - Duration: 6 months (28 weeks).  - Frequency: 3 times/week.  - Description: all workouts were preceded by a 10-min warm-up, which consisted of stretching of the major muscle groups and slow walking around the track. There were 4 phases of base training. The first phase (first 4 weeks) began with an exercise prescription of 25 min of slow jogging. Thereafter, the aerobic training program of each 4-week phase increased by 5 min. By the fourth phase (i.e. 16 weeks), women were jogging for approximately 40 min. Within the phases, the exercise intensity was increased by 5% of maximum heart rate each week, so that by the end of the fourth week of the fourth phase, the training was 40 min at 90% of maximum heart rate. The second part (weeks 16–28) used interval training sessions. Women followed a detailed program of specific workouts aimed at increasing exercise duration and intensity. The interval sessions consisted of 45 min of 80% of maximum heart rate training on Monday, four 5-min periods at 95% of maximum heart rate with 3-min rests on Wednesday, and 45 min at 75–80% of maximum heart rate on Friday. By the final week of training, women successfully completed 60-min sessions at 85% of maximum heart rate. |
| --- |

**Comparison group**

| Resistance training group  - Duration: 6 months (28 weeks).  - Frequency: 3 times/week.  - Description: all workouts were preceded by a 10-min warm-up, which consisted of stretching of the major muscle groups and slow walking around the track. Participants exercised on 3 non-consecutive days during the week. The training was approximately 80% of 1 repetition maximum (1-RM). Each training session included a warm-up of low intensity cycling for 5 min, followed by 10 min of static stretching of all of the major muscle groups used in training. Each exercise session was individually monitored for optimal progression by two trainers. The resistance program consisted of the following exercises: 1) leg press, 2) bench press, 3) leg extensions, 4) shoulder press, 5) sit-ups, 6) seated rows, 7) triceps extensions, 8) arm curls, and 9) leg curls. The exercises provided a total body resistance training program for all of the major muscle groups of the body. The volunteer was given a target load range and attempted to keep each set (n=3) within the target range by adjusting the load to allow the prescribed number (n=10) of repetitions. Resting periods were 1–1.5 min between sets. | Control group  - Not described. |
| --- | --- |

**Body composition outcomes and between groups comparison**

** statistically significant difference between running group and control group*

^†^ *statistically significant difference between running group and resistance group*

^‡^ *statistically significant difference within groups (pre-post)*

*SAT: subcutaneous adiposity tissue*

*VAT: visceral adiposity tissue*

| Mean 6 months after baseline | | |
| --- | --- | --- |
| Running group  - Body weight (kg): 59 (SD 5)  - BMI (kg/m^2^): 22 (SD 2)  - Body fat (kg): 15 (SD 4)  - Fat-free mass (kg): 40 (SD 4)  - SAT area (L4-L5, cm^2^): 193 (SD 80)  - VAT area (L4-L5, cm^2^): 41 (SD 13)  - Thigh fat area (cm^2^): 90 (SD 24) | Resistance group  - Body weight (kg): 60 (SD 6)^‡^  - BMI (kg/m^2^): 23 (SD 2)^‡^  - Body fat (kg): 17 (SD 4)  - Fat-free mass (kg): 41 (SD 3)^‡^  - SAT area (L4-L5, cm^2^): 186 (SD 85)  - VAT area (L4-L5, cm^2^): 36 (SD 13)  - Thigh fat area (cm^2^): 102 (SD 38) | Control group  - Body weight (kg): 61 (SD 8)  - BMI (kg/m^2^): 22 (SD 2)  - Body fat (kg): 17 (SD 6)  - Fat-free mass (kg): 40 (SD 3)  - SAT area (L4-L5, cm^2^): 210 (SD 95)  - VAT area (L4-L5, cm^2^): 41 (SD 15)  - Thigh fat area (cm^2^): 101 (SD 31) |

**Cardiorespiratory outcomes and between groups comparison**

** statistically significant difference between running group and control group*

^†^ *statistically significant difference between running group and resistance group*

^‡^ *statistically significant difference within groups (pre-post)*

| Mean 6 months after baseline | | |
| --- | --- | --- |
| Running group  - VO_2_max (l/min): 2.7 (SD 0.5)^‡^ | Resistance group  - VO_2_max (l/min): 2.2 (SD 0.3) | Control group  - VO_2_max (l/min): 2.3 (SD 0.4) |

**Blood serum concentration outcomes and between groups comparison**

** statistically significant difference between running group and control group*

^†^ *statistically significant difference between running group and resistance group*

^‡^ *statistically significant difference within groups (pre-post)*

| Mean 6 months after baseline | | |
| --- | --- | --- |
| Running group  - Insulin sensitivity (mg/min): 490 (SD 133)^‡^  - Insulin sensitivity (mg/kgFFM/min): 12.1 (SD 3.3)^‡^ | Resistance group  - Insulin sensitivity (mg/min): 417 (SD 89)^‡^  - Insulin sensitivity (mg/kgFFM/min): 10.2 (SD 1.8) | Control group  - Insulin sensitivity (mg/min): 480 (SD 168)  - Insulin sensitivity (mg/kgFFM/min): 11.8 (SD 3.5) |
| p.s.: Despite the authors interpreted the within result for insulin sensitivity expressed by mg/min in the resistance group as statistically significant different after 6 months from baseline, the p-value was p=0.06. | | |

**Reference**

| Bourque SP, Pate RR, Branch JD. Twelve weeks of endurance exercise training does not affect iron status measures in women. Journal of the American Dietetic Association. 1997;97(10):1116-21. |
| --- |

**Objectives**

| To examine the effects of 12 weeks of walking/running and cycling on iron status in previously inactive women. |
| --- |

**Description of participants included in the study**

| - Participants randomised: n=31 women with no regular exercise routine during the past 6 months.  - Source: Columbia, South Carolina, USA.  - Participants included in the analysis, n=21:   - Running group: n=8, mean age of 33 years (SD 4). - Cycling group: n=6, mean age of 32 years (SD 7). - Control group: n=7, mean age of 30 years (SD 4). |
| --- |

**Follow-up period and time-point assessments after baseline**

| - Follow-up: 12 weeks.  - Time-points:   - Baseline, 2, 4, 8 and 12 weeks after baseline: blood measurements and body weight. - Baseline and 12 weeks after baseline: other body composition measures and VO_2_max. |
| --- |
| p.s.: The results corresponding to follow-up after 2 and 4 weeks from baseline were not extracted because 8 weeks or more of running training is an inclusion criterion of the systematic review. |

**Intervention (running program)**

| Running group  - Duration: 12 weeks.  - Frequency: 3-4 times/week.  - Description: walking/running exercises at an intensity corresponding to 80% of VO_2_max. Exercise duration was adjusted so that average estimated energy expenditure increased from 150 kcal/session during 1^st^ week to 375 kcal/session during the last 4 weeks of the study. |
| --- |

**Comparison group**

| Cycling group  - Duration: 12 weeks.  - Frequency: 3-4 times/week.  - Description: cycling at an intensity corresponding to 80% of VO_2_max. Exercise duration was adjusted so that average estimated energy expenditure increased from 150 kcal/session during 1^st^ week to 375 kcal/session during the last 4 weeks of the study. | Control group  - Participants were instructed to maintain their normal activity patterns during the study. |
| --- | --- |

**Body composition outcomes and between groups comparison**

** statistically significant difference between running group and control group*

^†^ *statistically significant difference between running group and cycling group*

^‡^ *statistically significant difference within groups (pre-post)*

| Mean 12 weeks after baseline | | |
| --- | --- | --- |
| Running group  - Body weight (kg): 63 (SD 8)  - Body fat (%): 27 (SD 6)  - BMI (kg/m^2^): 23.3 (SD 2.9) | Cycling group  - Body weight (kg): 58 (SD 11)  - Body fat (%): 24 (SD 9)  - BMI (kg/m^2^): 21.7 (SD 3.8) | Control group  - Body weight (kg): 67 (SD 8)  - Body fat (%): 27 (SD 7)  - BMI (kg/m^2^): 24.5 (SD 4.2) |

**Cardiorespiratory outcomes and between groups comparison**

** statistically significant difference between running group and control group*

^†^ *statistically significant difference between running group and cycling group*

^‡^ *statistically significant difference within groups (pre-post)*

| Mean 12 weeks after baseline | | |
| --- | --- | --- |
| Running group  - VO_2_max (ml/kg/min): 38.8 (SD 7.4) | Cycling group  - VO_2_max (ml/kg/min): 38.8 (SD 5.9)^‡^ | Control group  - VO_2_max (ml/kg/min): 33.1 (SD 8.3) |
| Pre-post mean difference 12 weeks after baseline | | |
| Running group  - VO_2_max (%): 7.2 (not reported) | Cycling group  - VO_2_max (%): 19.4 (not reported)^‡^ | Control group  - VO_2_max (%): not reported |

**Blood serum concentration outcomes and between groups comparison**

** statistically significant difference between running group and control group*

^†^ *statistically significant difference between running group and cycling group*

^‡^ *statistically significant difference within groups (pre-post)*

| Mean 8 weeks after baseline | | |
| --- | --- | --- |
| Running group  - Serum ferritin (µg/l): 30 (SD 28)^†^  - Serum iron (µmol/l): 11 (SD 6)  - Total iron-binding capacity (µmol/l): 45 (SD 7)  - Transferrin saturation (%): 25 (SD 11)  - Hemoglobin (g/l): 122 (SD 5)*  - Hematocrit (unit not reported): 0.37 (SD 0.01)  - Serum haptoglobin (g/l): 1.44 (SD 0.56) | Cycling group  - Serum ferritin (µg/l): 37 (SD 23)  - Serum iron (µmol/l): 18 (SD 5)  - Total iron-binding capacity (µmol/l): 48 (SD 9)  - Transferrin saturation (%): 37 (SD 12)  - Hemoglobin (g/l): 130 (SD 9)  - Hematocrit (unit not reported): 0.39 (SD 0.02)  - Serum haptoglobin (g/l): 0.99 (SD 0.20) | Control group  - Serum ferritin (µg/l): 36 (SD 12)  - Serum iron (µmol/l): 11 (SD 8)  - Total iron-binding capacity (µmol/l): 46 (SD 9)  - Transferrin saturation (%): 25 (SD 14)  - Hemoglobin (g/l): 122 (SD 9)  - Hematocrit (unit not reported): 0.37 (SD 0.03)  - Serum haptoglobin (g/l): 1.47 (SD 0.59) |
| Mean 12 weeks after baseline | | |
| Running group  - Serum ferritin (µg/l): 27 (SD 10)  - Serum iron (µmol/l): 13 (SD 5)^†^  - Total iron-binding capacity (µmol/l): 45 (SD 3)  - Transferrin saturation (%): 28 (SD 8)^†^  - Hemoglobin (g/l): 122 (SD 7)*  - Hematocrit (unit not reported): 0.37 (SD 0.02)  - Serum haptoglobin (g/l): 1.29 (SD 0.53) | Cycling group  - Serum ferritin (µg/l): 41 (SD 26)  - Serum iron (µmol/l): 12 (SD 4)  - Total iron-binding capacity (µmol/l): 43 (SD 3)  - Transferrin saturation (%): 27 (SD 9)  - Hemoglobin (g/l): 127 (SD 6)  - Hematocrit (unit not reported): 0.38 (SD 0.01)  - Serum haptoglobin (g/l): 0.87 (SD 0.27) | Control group  - Serum ferritin (µg/l): 32 (SD 11)  - Serum iron (µmol/l): 13 (SD 4)  - Total iron-binding capacity (µmol/l): 47 (SD 8)  - Transferrin saturation (%): 30 (SD 12)  - Hemoglobin (g/l): 120 (SD 5)  - Hematocrit (unit not reported): 0.36 (SD 0.01)  - Serum haptoglobin (g/l): 1.30 (SD 0.70) |

**Reference**

| Hubinger L, Mackinnon LT. The effect of endurance training on lipoprotein(a) [Lp(a)] levels in middle-aged males. Medicine and science in sports and exercise. 1996;28(6):757-64. |
| --- |

**Objectives**

| To determine whether the level of lipoprotein(a) was altered by a 12-week of moderate or intensity endurance training program in previously sedentary middle-aged males. |
| --- |

**Description of participants included in the study**

| - Participants randomised: n=50 sedentary (no regular exercise, either occupationally or recreationally, for the past 3 years) middle-aged male.  - Participants included in the analysis, n=28:   - Running group: n=17, mean age of 51.57 years (SE 1.25). - Control group: n=11, mean age of 50.0 years (SE 1.15). |
| --- |

**Follow-up period and time-point assessments after baseline**

| - Follow-up: 12 weeks.  - Time-points: baseline and 12 weeks after baseline. |
| --- |

**Intervention (running program)**

| Running group  - Duration: 12 weeks.  - Frequency: 3 times/week.  - Description: participants were requested to walk and/or jog at low to moderate intensity (60-85% HR_max_ reserve) for at least 30 min each session. They were instructed about the components of each training session (i.e., warm-up, workout, and cool-down). |
| --- |

**Comparison group**

| Control group  - Not described. |
| --- |

**Body composition outcomes and between groups comparison**

** statistically significant difference between groups*

^‡^ *statistically significant difference within groups (pre-post)*

| Mean 12 weeks after baseline | |
| --- | --- |
| Running group  - Body weight (kg): 84.49 (SE 1.06)  - BMI (kg/m^2^): 27.16 (SE 0.77)  - Body fat (%): 15.91 (SE 0.75)^‡^  - Suprailiac skinfold (mm): 12.24 (SE 0.94)^‡^  - Abdomen skinfold (mm): 21.08 (SE 1.19)^‡^  - Thigh skinfold (mm): 13.33 (SE 1.02)^‡^  - Sum of 6 skinfolds (mm): 88.85 (SE 4.63)^‡^  - Umbilicus circumference (cm): 96.03 (SE 2.34)^‡^  - Trochanter circumference (cm): 100.00 (SE 1.45)^‡^  - Waist circumference (cm): 93.60 (SE 2.10)  - Hip circumference (cm): 102.62 (SE 2.51)  - Waist/hip ratio: 0.91 (SE 0.01)  - Umb/Troch ratio: 0.96 (SE 0.02) | Control group  - Body weight (kg): 84.71 (SE 1.17)^‡^  - Other results after 12 weeks (or change differences) from baseline for the control group were not reported in the article. |

**Cardiorespiratory outcomes and between groups comparison**

** statistically significant difference between groups*

^‡^ *statistically significant difference within groups (pre-post)*

| Mean 12 weeks after baseline | |
| --- | --- |
| Running group  - VO_2_max (ml/kg/min): 37.70 (SE 1.75)^‡^ | Control group  - All results after 12 weeks (or change differences) from baseline for the control group were not reported in the article. |

**Blood serum concentration outcomes and between groups comparison**

** statistically significant difference between groups*

^‡^ *statistically significant difference within groups (pre-post)*

*TC: total cholesterol*

| Mean 12 weeks after baseline | |
| --- | --- |
| Running group  - TC (unit nor reported): 5.22 (SE 0.25)^‡^  - HDL (unit not reported): 1.21 (SE 0.09)  - LDL (unit not reported): 3.60 (SE 0.22)  - Triglycerides (unit not reported): 1.29 (SE 0.12)  - Apolipoprotein A (g/l): 1.39 (SE 0.05)  - Apolipoprotein B (g/l): 0.98 (SE 0.04)  - TC/HDL: 4.31 (SE 0.39)  - Apo B/ Apo A: 0.70 (SE 0.04)  - Lipoprotein(a) (mg/dl): 28.26 (SE 5.73) | Control group  - Lipoprotein(a) (mg/dl): 12.0 (dispersion or uncertainty not reported)  - Other results after 12 weeks (or change differences) from baseline for the control group were not reported in the article. |

**Reference**

| Suter E, Marti B, Gutzwiller F. Jogging or walking--comparison of health effects. Annals of epidemiology. 1994;4(5):375-81. |
| --- |

**Objectives**

| To investigate changes in endurance capacity, body fat and serum lipids after 6 months of jogging and walking. |
| --- |

**Description of participants included in the study**

| - Participants randomised: n=75 males (most of them being bank employees recruited from different banks in Zurich) who did not spend more than 1 hour a week on endurance-type activities.  - Participants included in the analysis, n=75:   - Running group: n=28, mean age of 39.7 (SD 8.5). - Walking group: n=28, mean age of 42.2 (SD 7.4). - Control group: n=19, mean age of 42.1 (SD 7.6). |
| --- |

**Follow-up period and time-point assessments after baseline**

| - Follow-up: 6 months.  - Time-points: baseline and 6 months after baseline. |
| --- |

**Intervention (running program)**

| Running group  - Duration: 6 months.  - Frequency: 4 times/week.  - Description: home-based training program of either 4x30 min of jogging/week at a heart rate corresponding to 75% VO_2_max (90 min/week, SD 41). Running and walking groups were chosen in such a way that they resulted in similar weekly energy expenditure. |
| --- |

**Comparison group**

| Walking group  - Duration: 6 months.  - Frequency: 6 times/week.  - Description: home-based training program of either 6x30 min of walking/week at a heart rate corresponding to 50% VO_2_max (121 min/week, SD 72). Walking and running groups were chosen in such a way that they resulted in similar weekly energy expenditure. | Control group  - Participants were asked to maintain their normal physical activities but to refrain from taking up any regular exercise. |
| --- | --- |

**Body composition outcomes and between groups comparison**

** statistically significant difference between running group and control group*

^†^ *statistically significant difference between running group and walking group*

*^‡^ statistically significant difference within groups (pre-post)*

| Pre-post mean difference 6 months after baseline | | |
| --- | --- | --- |
| Running group  - ΔBMI (kg/m^2^): -0.28 (SD 0.74)  - ΔSum of 4 skinfolds (mm): -3.7 (SD 11.1)  - ΔWaist-hip ratio: 0.03 (SD 0.03) | Walking group  - ΔBMI (kg/m^2^): -0.07 (SD 0.90)  - ΔSum of 4 skinfolds (mm): -1.5 (SD 14.3)  - ΔWaist-hip ratio: 0.04 (SD 0.04) | Control group  - ΔBMI (kg/m^2^): -0.09 (SD 0.75)  - ΔSum of 4 skinfolds (mm): -3.3 (SD 8.8)  - ΔWaist-hip ratio: 0.03 (SD 0.04) |

**Cardiorespiratory outcomes and between groups comparison**

** statistically significant difference between running group and control group*

^†^ *statistically significant difference between running group and walking group*

*^‡^ statistically significant difference within groups (pre-post)*

| Pre-post mean difference 6 months after baseline | | |
| --- | --- | --- |
| Running group  - ΔVO_2_max (ml/kg/min): 2.9 (SD 4.1)* | Walking group  - ΔVO_2_max (ml/kg/min): 2.5 (SD 5.7) | Control group  - ΔVO_2_max (ml/kg/min): -1.2 (SD 3.3) |

**Blood serum concentration outcomes and between groups comparison**

** statistically significant difference between running group and control group*

^†^ *statistically significant difference between running group and walking group*

*^‡^ statistically significant difference within groups (pre-post)*

| Pre-post mean difference 6 months after baseline | | |
| --- | --- | --- |
| Running group  - ΔTotal cholesterol (mmol/l): 0.44 (SD 0.67)  - ΔTotal cholesterol (mg/dl): 17 (SD 26)  - ΔHDL (mmol/l): -0.00 (SD 0.17)  - ΔHDL (mg/dl): -0.2 (SD 6.5)  - ΔTotal cholesterol/HDL ratio: 0.31 (SD 0.66)  - ΔTotal triglycerides (mmol/l): 0.24 (SD 0.85)  - ΔTotal triglycerides (mg/dl): 21 (SD 75)  - ΔApolipoprotein A-I (g/l): 0.11 (SD 0.18)  - ΔApolipoprotein B (g/l): 0.14 (SD 0.18) | Walking group  - ΔTotal cholesterol (mmol/l): 0.16 (SD 0.72)  - ΔTotal cholesterol (mg/dl): 6 (SD 28)  - ΔHDL (mmol/l): 0.01 (SD 0.15)  - ΔHDL (mg/dl): 0.4 (SD 5.9)  - ΔTotal cholesterol/HDL ratio: 0.05 (SD 0.88)  - ΔTotal triglycerides (mmol/l): 0.32 (SD 0.96)  - ΔTotal triglycerides (mg/dl): 28 (SD 84)  - ΔApolipoprotein A-I (g/l): 0.12 (SD 0.19)  - ΔApolipoprotein B (g/l): 0.09 (SD 0.17) | Control group  - ΔTotal cholesterol (mmol/l): 0.15 (SD 0.74)  - ΔTotal cholesterol (mg/dl): 6 (SD 29)  - ΔHDL (mmol/l): -0.04 (SD 0.14)  - ΔHDL (mg/dl): -1.5 (SD 5.4)  - ΔTotal cholesterol/HDL ratio: 0.31 (SD 0.66)  - ΔTotal triglycerides (mmol/l): 0.06 (SD 0.67)  - ΔTotal triglycerides (mg/dl): 5 (SD 59)  - ΔApolipoprotein A-I (g/l): 0.07 (SD 0.13)  - ΔApolipoprotein B (g/l): 0.07 (SD 0.14) |

**Reference**

| Garber CE, McKinney JS, Carleton RA. Is aerobic dance an effective alternative to walk-jog exercise training? The Journal of sports medicine and physical fitness. 1992;32(2):136-41. |
| --- |

**Objectives**

| To compare the relative alterations in maximal aerobic power resulting from aerobic dance and walk-jog exercise training. |
| --- |

**Description of participants included in the study**

| - Participants randomised: n=60 university employees who had not been participating in any regular physical activity for the previous 3 months.  - Participants included in the analysis, n=35   - Running group: n=11, mean age of 39 years (SD 5), 5 men and 6 women. - Aerobic dance group: n=14, mean age of 35 years (SD 7), 7 men and 7 women. - Control group: n=10, mean age of 35 years (SD 6), 5 men and 5 women. |
| --- |

**Follow-up period and time-point assessments after baseline**

| - Follow-up: 8 weeks.  - Time-points: baseline and 8 weeks after baseline. |
| --- |

**Intervention (running program)**

| Running group  - Duration: 8 weeks.  - Frequency: 3 times/week.  - Description: walking and/or jogging 50 min/day, 3 alternate days/week. The exercise sessions consisted of 10 min of warm-up and stretching, 15-25 min of activity, 10-15 min of calisthenics and 5 min of cool-down. Intensity was set at the heart rate of 60-80% of the peak oxygen uptake, and was gradually increased throughout the training period. |
| --- |

**Comparison group**

| Aerobic dance group  - Duration: 8 weeks.  - Frequency: 3 times/week.  - Description: Series of exercise routines which included jumping motions accompanied by a variety of arm motions, 50 min/day, 3 alternate days/week. The exercise sessions consisted of 10 min of warm-up and stretching, 15-25 min of activity, 10-15 min of calisthenics and 5 min of cool-down. Intensity was set at the heart rate of 60-80% of the peak oxygen uptake, and was gradually increased throughout the training period. | Control group  - Participants were advised to maintain their usual sedentary activity patterns during the 8 weeks. |
| --- | --- |

**Body composition outcomes and between groups comparison**

** statistically significant difference running group and control group*

^†^ *statistically significant difference between running group and aerobic dance group*

^‡^ *statistically significant difference within groups (pre-post)*

| Mean 8 weeks after baseline | | |
| --- | --- | --- |
| Running group  - Body weight (kg): 160 (SD 19) | Aerobic dance group  - Body weight (kg): 147 (SD 34) | Control group  - Body weight (kg): 160 (SD 38) |

**Cardiovascular outcomes and between groups comparison**

** statistically significant difference running group and control group*

^†^ *statistically significant difference between running group and aerobic dance group*

^‡^ *statistically significant difference within groups (pre-post)*

| Mean 8 weeks after baseline | | |
| --- | --- | --- |
| Running group  - VO_2_peak (ml/kg/min): 34.7 (SD 9.6)^‡^  - Peak minute ventilation (l/min): 87.4 (SD 24.5)  - Peak respiratory exchange ratio: 1.1 (SD 0.1) | Aerobic dance group  - VO_2_peak (ml/kg/min): 37.9 (SD 8.4)^‡^  - Peak minute ventilation (l/min): 96.9 (SD 24.3)  - Peak respiratory exchange ratio: 1.1 (SD 0.1) | Control group  - VO_2_peak (ml/kg/min): 40.7 (SD 11.2)  - Peak minute ventilation (l/min): 90.3 (SD 22.3)  - Peak respiratory exchange ratio: 1.1 (SD 0.2) |
| Pre-post mean difference 8 weeks after baseline | | |
| Running group  - ΔVO_2_peak (ml/kg/min): 3.4 (dispersion or uncertainty not reported)^‡^ | Aerobic dance group  - ΔVO_2_peak (ml/kg/min): 3.9 (dispersion or uncertainty not reported)^‡^ | Control group  - ΔVO_2_peak (ml/kg/min): not reported |

**Metabolic outcomes and between groups comparison**

** statistically significant difference running group and control group*

^†^ *statistically significant difference between running group and aerobic dance group*

^‡^ *statistically significant difference within groups (pre-post)*

| Not applicable | Not applicable | Not applicable |
| --- | --- | --- |

**Reference**

| Suter E, Marti B. Little effect of long-term, self-monitored exercise on serum lipid levels in middle-aged women. The Journal of sports medicine and physical fitness. 1992;32(4):400-11. |
| --- |

**Objectives**

| To determine whether a self-monitored, home-exercise program of 4 months would produce changes in serum lipid levels in sedentary middle-aged women. |
| --- |

**Description of participants included in the study**

| - Participants randomised: n=33 women who had the pre-study involvement in regular endurance sports activities less than 1 hour/week (most of them were upper middle-class bank employees).  - Participants included in the analysis, n=32:   - Running group: n=16. The baseline mean age of the 17 participants randomly assigned to this group was 38.2 years (SD 9.4). - Control group: n=16, mean age of 42.2 years (SD 8.2). |
| --- |

**Follow-up period and time-point assessments after baseline**

| - Follow-up: 4 months.  - Time-points: baseline and 4 months after baseline. |
| --- |

**Intervention (running program)**

| Running group  - Duration: 4 months.  - Frequency: 2-6 times/week.  - Description: home-based program of at least 120 min jogging, or walking/jogging per week. Participants were free to divide the target activity of 120 min/week into 2-6 sessions. Intensity was set to be at approximately 80% of the maximum heart rate. |
| --- |

**Comparison group**

| Control group  - Not described. |
| --- |

**Body composition outcomes and between groups comparison**

** statistically significant difference between groups*

^‡^ *statistically significant difference within groups (pre-post)*

| Pre-post mean difference 4 months after baseline | |
| --- | --- |
| Running group  - ΔBMI (kg/m^2^): -0.02 (dispersion or uncertainty not described)  - ΔBody fat (%): -0.45 (dispersion or uncertainty not described)  - ΔSum of 4 skinfolds (mm): -4.39 (dispersion or uncertainty not described)  - ΔWaist-hip ratio: -0.00 (dispersion or uncertainty not described) | Control group  - ΔBMI (kg/m^2^): 0.02 (dispersion or uncertainty not described)  - ΔBody fat (%): -0.80 (dispersion or uncertainty not described)  - ΔSum of 4 skinfolds (mm): 0.46 (dispersion or uncertainty not described)  - ΔWaist-hip ratio: 0.01 (dispersion or uncertainty not described) |
| Running-control group difference after 4 months of the changes from baseline between groups (net change)  - ΔBMI (kg/m^2^): -0.04 (95%CI -0.42 to 0.34)  - ΔBody fat (%): 0.35 (95%CI -1.55 to 2.26)  - ΔSum of 4 skinfolds (mm): -4.85 (95%CI -9.47 to -0.23)*  - ΔWaist-hip ratio: -0.01 (95%CI -0.04 to 0.02) | |

**Cardiorespiratory outcomes and between groups comparison**

** statistically significant difference between groups*

^‡^ *statistically significant difference within groups (pre-post)*

| Not applicable | Not applicable |
| --- | --- |

**Blood serum concentration outcomes and between groups comparison**

** statistically significant difference between groups*

^‡^ *statistically significant difference within groups (pre-post)*

| Pre-post mean difference 4 months after baseline | |
| --- | --- |
| Running group  - ΔTotal cholesterol (mmol/l): -0.23 (dispersion or uncertainty not described)  - ΔHDL (mmol/l): 0.06 (dispersion or uncertainty not described)  - ΔHDL_2_ (mmol/l): 0.12 (dispersion or uncertainty not described)  - ΔHDL_3_ (mmol/l): -0.06 (dispersion or uncertainty not described)  - ΔLDL (mmol/l): 0.02 (dispersion or uncertainty not described)  - ΔVLDL (mmol/l): -0.23 (dispersion or uncertainty not described)  - ΔTotal triglycerides (mmol/l): -0.13 (dispersion or uncertainty not described)  - ΔApolipoprotein A-I (g/l): 0.11 (dispersion or uncertainty not described)  - ΔApolipoprotein B (g/l): 0.04 (dispersion or uncertainty not described)  - ΔHDL/total cholesterol: 0.02 (dispersion or uncertainty not described)  - ΔApolipoprotein B/ apolipoprotein A-I: -0.02 (dispersion or uncertainty not described) | Control group  - ΔTotal cholesterol (mmol/l): -0.27 (dispersion or uncertainty not described)  - ΔHDL (mmol/l): 0.06 (dispersion or uncertainty not described)  - ΔHDL_2_ (mmol/l): 0.10 (dispersion or uncertainty not described)  - ΔHDL_3_ (mmol/l): 0.04 (dispersion or uncertainty not described)  - ΔLDL (mmol/l): 0.00 (dispersion or uncertainty not described)  - ΔVLDL (mmol/l): -0.32 (dispersion or uncertainty not described)  - ΔTotal triglycerides (mmol/l): 0.04 (dispersion or uncertainty not described)  - ΔApolipoprotein A-I (g/l): 0.00 (dispersion or uncertainty not described)  - ΔApolipoprotein B (g/l): 0.05 (dispersion or uncertainty not described)  - ΔHDL/total cholesterol: 0.03 (dispersion or uncertainty not described)  - ΔApolipoprotein B/ apolipoprotein A-I: 0.03 (dispersion or uncertainty not described) |
| Running-control group difference after 4 months considering the changes from baseline (net change)  - ΔTotal cholesterol (mmol/l): 0.04 (95%CI -0.47 to 0.57)  - ΔHDL (mmol/l): 0.00 (95%CI -0.24 to 0.24)  - ΔHDL_2_ (mmol/l): 0.02 (95%CI -0.06 to 0.10)  - ΔHDL_3_ (mmol/l): -0.02 (95%CI -0.11 to 0.07)  - ΔLDL (mmol/l): 0.02 (95%CI -0.46 to 0.49)  - ΔVLDL (mmol/l): 0.09 (95%CI -0.13 to 0.32)  - ΔTotal triglycerides (mmol/l): -0.17 (95%CI -0.37 to 0.05)  - ΔApolipoprotein A-I (g/l): 0.11 (95%CI -0.06 to 0.27)  - ΔApolipoprotein B (g/l): -0.01 (95%CI -0.51 to 0.49)  - ΔHDL/total cholesterol: -0.01 (95%CI -0.04 to 0.04)  - ΔApolipoprotein B/ apolipoprotein A-I: -0.05 (95%CI -0.12 to 0.02) | |

**More publications related to this study**

| Marti B, Suter E, Riesen WF, Tschopp A, Wanner HU, Gutzwiller F. Effects of long-term, self-monitored exercise on the serum lipoprotein and apolipoprotein profile in middle-aged men. Atherosclerosis. 1990;81(1):19-31. |
| --- |
| Suter E, Marti B, Tschopp A, Wanner HU, Wenk C, Gutzwiller F. Effects of self-monitored jogging on physical fitness, blood pressure and serum lipids: a controlled study in sedentary middle-aged men. International journal of sports medicine. 1990;11(6):425-32. |

**Reference**

| Williams PT, Krauss RM, Vranizan KM, Albers JJ, Wood PD. Effects of weight-loss by exercise and by diet on apolipoproteins A-I and A-II and the particle-size distribution of high-density lipoproteins in men. Metabolism: clinical and experimental. 1992;41(4):441-9. |
| --- |

**Objectives**

| To investigate the effects of weight-loss by dieting or by running on apolipoprotein A-l, apolipoprotein A-II, and HDL subfractions in sedentary, moderately overweight men. |
| --- |

**Description of participants included in the study**

| - Participants randomised: n=155 sedentary overweight men aged 30-59.  - Participants included in the analysis, n=130 for apolipoproteins, body weight, lean and fat body mass; n=97 for HDL:   - Running group: - n=46 for apolipoproteins A-I and A-II; - n=35 for HDL. - Diet group: - n=42 for apolipoproteins A-I and A-II; - n=31 for HDL. - Control group: - n=42 for apolipoproteins A-I and A-II; - n=31 for HDL. |
| --- |

**Follow-up period and time-point assessments after baseline**

| - Follow-up: 1 year.  - Time-points: baseline, 7 and 12 months after baseline. |
| --- |

**Intervention (running program)**

| Running group  - Duration: 1 year.  - Frequency: not described.  - Description: the participants ran 15.6 km/week (SD 9.1).  p.s.: additional information in *Wood PD, Stefanick ML, Dreon DM, Frey-Hewitt B, Garay SC, Williams PT, et al. Changes in plasma lipids and lipoproteins in overweight men during weight loss through dieting as compared with exercise. The New England journal of medicine. 1988;319(18):1173-9,* and *Williams PT, Krauss RM, Vranizan KM, Wood PD. Changes in lipoprotein subfractions during diet-induced and exercise-induced weight loss in moderately overweight men. Circulation. 1990;81(4):1293-304*. |
| --- |

**Comparison group**

| Diet group  - Calorie restriction without increasing exercise. | Control group  - No change in diet or exercise. |
| --- | --- |
| p.s.: additional information in *Wood PD, Stefanick ML, Dreon DM, Frey-Hewitt B, Garay SC, Williams PT, et al. Changes in plasma lipids and lipoproteins in overweight men during weight loss through dieting as compared with exercise. The New England journal of medicine. 1988;319(18):1173-9,* and *Williams PT, Krauss RM, Vranizan KM, Wood PD. Changes in lipoprotein subfractions during diet-induced and exercise-induced weight loss in moderately overweight men. Circulation. 1990;81(4):1293-304*. | |

**Body composition outcomes and between groups comparison**

** statistically significant difference between running group and control group*

^†^ *statistically significant difference between running group and diet group*

*^‡^ statistically significant difference within groups (pre-post)*

| Pre-post mean difference 1 year after baseline | | |
| --- | --- | --- |
| Running group  - ΔBody weight (kg): -4.0 (SD 3.9)*  - ΔBody fat mass (kg): -4.6 (SD 3.5)  - ΔLean body mass (kg): -0.1 (SD 2.1) | Diet group  - ΔBody weight (kg): -7.2 (SD 4.1)  - ΔBody fat mass (kg): -6.2 (SD 4.1)  - ΔLean body mass (kg): -1.2 (SD 2.4) | Control group  - ΔBody weight (kg): 0.6 (SD 3.7)  - ΔBody fat mass (kg): not reported  - ΔLean body mass (kg): 1.3 (SD 2.0) |

**Cardiorespiratory outcomes and between groups comparison**

** statistically significant difference between running group and control group*

^†^ *statistically significant difference between running group and diet group*

*^‡^ statistically significant difference within groups (pre-post)*

| Not applicable | Not applicable | Not applicable |
| --- | --- | --- |

**Blood serum concentration outcomes and between groups comparison**

** statistically significant difference between running group and control group*

^†^ *statistically significant difference between running group and diet group*

*^‡^ statistically significant difference within groups (pre-post)*

| Pre-post mean difference 7 months after baseline | |
| --- | --- |
| Running – Control group  - ΔApolipoprotein A-I (mg/dl): 2.45 (SE 3.25)  - ΔApolipoprotein A-II (mg/dl): -0.20 (SE 0.85)  - ΔHDL subclasses: results reported in graphs* | Running – Diet group  - ΔApolipoprotein A-I (mg/dl): 5.49 (SE 3.27)  - ΔApolipoprotein A-II (mg/dl): 2.10 (SE 0.85)^†^  - ΔHDL subclasses: results reported in graphs |
| Pre-post mean difference 1 year after baseline | |
| Running – Control group  - ΔApolipoprotein A-I (mg/dl): 8.49 (SE 3.07)*  - ΔApolipoprotein A-II (mg/dl): 1.45 (SE 0.89)  - ΔHDL subclasses: results reported in graphs* | Running – Diet group  - ΔApolipoprotein A-I (mg/dl): 4.43 (SE 3.09)  - ΔApolipoprotein A-II (mg/dl): 2.61 (SE 0.90)^†^  - ΔHDL subclasses: results reported in graphs |
| p.s.: the article did not reported the results of running and diet groups independently (actual or change in time) in any time-point of follow-up. | |

**More publications related to this study**

| Wood PD, Stefanick ML, Dreon DM, Frey-Hewitt B, Garay SC, Williams PT, et al. Changes in plasma lipids and lipoproteins in overweight men during weight loss through dieting as compared with exercise. The New England journal of medicine. 1988;319(18):1173-9. |
| --- |
| Williams PT, Krauss RM, Vranizan KM, Wood PD. Changes in lipoprotein subfractions during diet-induced and exercise-induced weight loss in moderately overweight men. Circulation. 1990;81(4):1293-304. |

**Reference**

| Oja P, Laukkanen RM, Kukkonen-Harjula TK, Vuori IM, Pasanen ME, Niittymaki SP, et al. Training effects of cross-country skiing and running on maximal aerobic cycle performance and on blood lipids. European journal of applied physiology and occupational physiology. 1991;62(6):400-4. |
| --- |

**Objectives**

| To determine whether cross-country skiing and running training results in different effects on the cardiorespiratory response in maximal cycle ergometer exercise or on serum lipids of untrained middle-aged men. |
| --- |

**Description of participants included in the study**

| - Participants randomised: n=96 men with 40 years old who had been engaged in vigorous aerobic exercise less than twice a week during the previous year.  - Participants included in the analysis, n=93:   - Running group: n=32. - Skiing group: n=29. - Control group: n=32. |
| --- |

**Follow-up period and time-point assessments after baseline**

| - Follow-up: 9-10 weeks.  - Time-points: before and 9-10 weeks after baseline. |
| --- |

**Intervention (running program)**

| Running group  - Duration: 9-10 weeks.  - Frequency: 3 times/week.  - Description: the road-running training lasted 9 weeks for 16 participants during the 1^st^ winter of the study, and 10 weeks for the remaining 16 participants during the 2^nd^ winter (consecutive). There were 3 sessions/week, each session lasting 40 min. The training intensity was set to correspond to 75%-85% of the maximal oxygen consumption (V0_2_max). |
| --- |

**Comparison group**

| Skiing group  - Duration: 9-10 weeks.  - Frequency: 3 times/week.  - Description: the skiing training lasted 9 weeks for 14 participants (1^st^ winter) and 10 weeks for 15 participants (2^nd^ consecutive winter). There were 3 sessions/week, each session lasting 40 min. The training intensity was set to correspond to 75%-85% of the maximal oxygen consumption (V0_2_max). The diagonal skiing style was predominantly practised during the study. All the participants had previous skiing experience. | Control group  - Participants were asked to continue their usual dietary and smoking habits and to maintain their pre-study exercise routine, which was no more than once a week. |
| --- | --- |

**Body composition outcomes and between groups comparison**

** statistically significant difference between running group and control group*

^†^ *statistically significant difference between running group and skiing group*

^‡^ *statistically significant difference within groups (pre-post)*

| p.s.: The results for body mass were not reported, however there was no statistically significant difference between or within groups. |
| --- |

**Cardiorespiratory outcomes and between groups comparison**

** statistically significant difference between running group and control group*

^†^ *statistically significant difference between running group and skiing group*

^‡^ *statistically significant difference within groups (pre-post)*

*V_E_: pulmonary ventilation*

| Mean 9-10 weeks after baseline | | |
| --- | --- | --- |
| Running group  - VO_2_max (l/min): 3.16 (SD 0.55)*  - VO_2_max (ml/kg/min): 41.3 (SD 5.3)*  - V_E_max (l/min): 116.3 (SD 23.6)*  - Max power output (W): 224 (SD 29)* | Skiing group  - VO_2_max (l/min): 3.25 (SD 0.39)  - VO_2_max (ml/kg/min): 40.9 (SD 5.0)  - V_E_max (l/min): 121.4 (SD 26.4)  - Max power output (W): 224 (SD 40) | Control group  - VO_2_max (l/min): 2.94 (SD 0.45)  - VO_2_max (ml/kg/min): 36.6 (SD 5.5)  - V_E_max (l/min): 102.5 (SD 18.2)  - Max power output (W): 212 (SD 42) |
| p.s.: The results for respiratory exchange ratio were not reported, however there was no statistically significant difference between or within groups. | | |

**Blood serum concentration outcomes and between groups comparison**

** statistically significant difference between running group and control group*

^†^ *statistically significant difference between running group and skiing group*

^‡^ *statistically significant difference within groups (pre-post)*

| Mean 9-10 weeks after baseline | | |
| --- | --- | --- |
| Running group  - Phospholipids (mmol/l): 3.00 (SD 0.39)^†^ | Skiing group  - Phospholipids (mmol/l): 2.93 (SD 0.37) | Control group  - Phospholipids (mmol/l): not reported |
| p.s.: The results for peak capillary la concentration, cholesterol and HDL-C after the 9-10 weeks of follow-up were not reported, however there was no statistically significant difference between or within groups. | | |

**Reference**

| Marti B, Suter E, Riesen WF, Tschopp A, Wanner HU, Gutzwiller F. Effects of long-term, self-monitored exercise on the serum lipoprotein and apolipoprotein profile in middle-aged men. Atherosclerosis. 1990;81(1):19-31. |
| --- |

**Objectives**

| 1- To evaluate the effects of a randomised program of 4 months of individually prescribed, self-monitored jogging on body fat content, endurance capacity, and serum apolipoprotein and lipoprotein concentrations, with particular reference to HDL and its subfractions;  2- To evaluate the effectiveness of an individually ‘tailored’ exercise prescription in increasing the level of physical activity of habitually sedentary subjects in an unsupervised setting. |
| --- |

**Description of participants included in the study**

| - Participants randomised: n=61 men, most of them bank employees, with no more than 1 h/week of endurance sports activity.  - Participants included in the analysis, n=61:   - Running group: n=39, mean age of 38.8 years (8.9). - Control group: n=22, mean age of 35.2 years (7.3). |
| --- |
| p.s.: The unit for the dispersion or uncertainty measure for age was not described. |

**Follow-up period and time-point assessments after baseline**

| - Follow-up: 4 months.  - Time-points: baseline and 4 months after baseline. |
| --- |

**Intervention (running program)**

| Running group  - Duration: 4 months.  - Frequency: 2-6 times/week.  - Description: participants were encouraged to adhere to a principally home-based program of at least 120 min/week of jogging, or walking/jogging. Participants were free to divide the target activity of 120 min/week into 2-6 sessions. The individually prescribed training intensity was 85% of the heart rate at the anaerobic threshold. |
| --- |

**Comparison group**

| Control group  - Sedentary control group. |
| --- |

**Body composition outcomes and between groups comparison**

** statistically significant difference between groups*

^‡^ *statistically significant difference within groups (pre-post)*

| Pre-post mean difference 4 months after baseline | |
| --- | --- |
| Running group  - ΔBMI (kg/m^2^): -0.08 (dispersion or uncertainty not reported)  - ΔSum of 4 skinfolds (mm): -1.92 (dispersion or uncertainty not reported)  - ΔBody fat (%): -1.30 (dispersion or uncertainty not reported)  - ΔWaist/hip ratio: -0.006 (dispersion or uncertainty not reported) | Control group  - ΔBMI (kg/m^2^): 0.12 (dispersion or uncertainty not reported)  - ΔSum of 4 skinfolds (mm): 0.89 (dispersion or uncertainty not reported)  - ΔBody fat (%): -0.22 (dispersion or uncertainty not reported)  - ΔWaist/hip ratio: 0.014 (dispersion or uncertainty not reported) |
| Running-control group difference of the changes from baseline between groups after 4 months (net change) | |
| - ΔBMI (kg/m^2^): -0.20 (95%CI -0.43 to 0.03)  - ΔSum of 4 skinfolds (mm): -2.81 (95%CI -6.04 to 0.42)  - ΔBody fat (%): -1.08 (95%CI -2.71 to 0.54)  - ΔWaist/hip ratio: -0.020 (95%CI -0.03 to 0.01)* | |

**Cardiorespiratory outcomes and between groups comparison**

** statistically significant difference between groups*

^‡^ *statistically significant difference within groups (pre-post)*

| Pre-post mean difference 4 months after baseline | |
| --- | --- |
| Running group  - ΔResting heart rate (bpm): -3.5 (dispersion or uncertainty not reported) | Control group  - ΔResting heart rate (bpm): 1.3 (dispersion or uncertainty not reported) |
| Running-control group difference of the changes from baseline between groups after 4 months (net change) | |
| - ΔResting heart rate (bpm): -4.8 (95%CI -8.4 to -1.1)* | |

**Blood serum concentration outcomes and between groups comparison**

** statistically significant difference between groups*

^‡^ *statistically significant difference within groups (pre-post)*

*TG: triglycerides*

| Pre-post mean difference 4 months after baseline | |
| --- | --- |
| Running group  - ΔCholesterol (mmol/l): -0.38 (dispersion or uncertainty not reported)  - ΔVLDL (mmol/l): -0.33 (dispersion or uncertainty not reported)  - ΔLDL (mmol/l): -0.07 (dispersion or uncertainty not reported)  - ΔHDL (mmol/l): 0.08 (dispersion or uncertainty not reported)  - ΔHDL_2_ (mmol/l): 0.06 (dispersion or uncertainty not reported)  - ΔHDL_3_ (mmol/l): 0.01 (dispersion or uncertainty not reported)  - ΔApolipoprotein B (g/l): 0.07 (dispersion or uncertainty not reported)  - ΔApolipoprotein A-I (g/l): 0.10 (dispersion or uncertainty not reported)  - ΔApolipoprotein E (g/l): -0.01 (dispersion or uncertainty not reported)  - ΔTriglycerides (mmol/l): -0.28 (dispersion or uncertainty not reported)  - ΔVLDL-TG (mmol/l): -0.09 (dispersion or uncertainty not reported)  - ΔLDL-TG (mmol/l): -0.13 (dispersion or uncertainty not reported)  - ΔHDL-TG (mmol/l): -0.02 (dispersion or uncertainty not reported)  - ΔHDL/cholesterol ratio: 0.031 (dispersion or uncertainty not reported)  - ΔHDL_2_/HDL_3_ ratio: 0.06 (dispersion or uncertainty not reported) | Control group  - ΔCholesterol (mmol/l): -0.37 (dispersion or uncertainty not reported)  - ΔVLDL (mmol/l): -0.08 (dispersion or uncertainty not reported)  - ΔLDL (mmol/l): -0.15 (dispersion or uncertainty not reported)  - ΔHDL (mmol/l): -0.04 (dispersion or uncertainty not reported)  - ΔHDL_2_ (mmol/l): 0.00 (dispersion or uncertainty not reported)  - ΔHDL_3_ (mmol/l): -0.04 (dispersion or uncertainty not reported)  - ΔApolipoprotein B (g/l): 0.10 (dispersion or uncertainty not reported)  - ΔApolipoprotein A-I (g/l): 0.09 (dispersion or uncertainty not reported)  - ΔApolipoprotein E (g/l): -0.01 (dispersion or uncertainty not reported)  - ΔTriglycerides (mmol/l): -0.07 (dispersion or uncertainty not reported)  - ΔVLDL-TG (mmol/l): -0.05 (dispersion or uncertainty not reported)  - ΔLDL-TG (mmol/l): 0.06 (dispersion or uncertainty not reported)  - ΔHDL-TG (mmol/l): -0.07 (dispersion or uncertainty not reported)  - ΔHDL/cholesterol ratio: 0.008 (dispersion or uncertainty not reported)  - ΔHDL_2_/HDL_3_ ratio: 0.07 (dispersion or uncertainty not reported) |
| Running-control group difference of the changes from baseline between groups after 4 months (net change) | |
| - ΔCholesterol (mmol/l): -0.01 (95%CI -0.48 to 0.45)  - ΔVLDL (mmol/l): -0.25 (95%CI -0.44 to -0.07)*  - ΔLDL (mmol/l): 0.08 (95%CI -0.29 to 0.44)  - ΔHDL (mmol/l): 0.12 (95%CI 0.01 to 0.22)*  - ΔHDL_2_ (mmol/l): 0.06 (95%CI -0.03 to 0.15)  - ΔHDL_3_ (mmol/l): 0.05 (95%CI -0.05 to 0.14)  - ΔApolipoprotein B (g/l): -0.03 (95%CI -0.13 to 0.07)  - ΔApolipoprotein A-I (g/l): 0.01 (95%CI -0.11 to 0.13)  - ΔApolipoprotein E (g/l): -0.00 (95%CI -0.08 to 0.07)  - ΔTriglycerides (mmol/l): -0.21 (95%CI -0.54 to 0.12)  - ΔVLDL-TG (mmol/l): -0.04 (95%CI -0.35 to 0.27)  - ΔLDL-TG (mmol/l): -0.19 (95%CI -0.28 to -0.08)*  - ΔHDL-TG (mmol/l): 0.05 (95%CI 0.02 to 0.08)*  - ΔHDL/cholesterol ratio: 0.023 (95%CI 0.00 to 0.05)*  - ΔHDL_2_/HDL_3_ ratio: -0.01 (95%CI -0.48 to 0.46) | |

**More publications related to this study**

| Suter E, Marti B, Tschopp A, Wanner HU, Wenk C, Gutzwiller F. Effects of self-monitored jogging on physical fitness, blood pressure and serum lipids: a controlled study in sedentary middle-aged men. International journal of sports medicine. 1990;11(6):425-32. |
| --- |
| Suter E, Marti B. Little effect of long-term, self-monitored exercise on serum lipid levels in middle-aged women. The Journal of sports medicine and physical fitness. 1992;32(4):400-11. |

**Reference**

| Suter E, Marti B, Tschopp A, Wanner HU, Wenk C, Gutzwiller F. Effects of self-monitored jogging on physical fitness, blood pressure and serum lipids: a controlled study in sedentary middle-aged men. International journal of sports medicine. 1990;11(6):425-32. |
| --- |

**Objectives**

| To examine in sedentary healthy middle-aged men the effects of 4 months of individually prescribed, heart rate controlled and home-based jogging on endurance capacity, body fat content and cardiovascular risk factors. |
| --- |

**Description of participants included in the study**

| - Participants randomised: n=61 men with no more than one weekly hour of regular endurance sports activity (most of them were bank employees).  - Participants included in the analysis, n=61:   - Running group: n=39, mean age of 38.8 years (SD 8.9). - Control group: n=22, mean age of 35.2 years (SD 7.3). |
| --- |

**Follow-up period and time-point assessments after baseline**

| - Follow-up: 4 months.  - Time-points: baseline and 4 months after baseline. |
| --- |

**Intervention (running program)**

| Running group  - Duration: 4 months.  - Frequency: 2-6 times/week.  - Description: home-based program of at least 120 min of jogging or walking/jogging per week (2-6 sessions depending on participants choice). The prescribed intensity was 85% of the heart rate. |
| --- |

**Comparison group**

| Control group  - Sedentary group. |
| --- |

**Body composition outcomes and between groups comparison**

** statistically significant difference between groups*

*^‡^ statistically significant difference within groups (pre-post)*

| Pre-post mean difference 4 months after baseline | |
| --- | --- |
| Running group  - ΔBMI (kg/m^2^): -0.08 (dispersion or uncertainty not reported)  - Body fat (%): -1.30 (dispersion or uncertainty not reported)  - ΔSum of 4 skin-folds (mm): -1.92 (dispersion or uncertainty not reported)  - ΔWaist-hip ratio: -0.006 (dispersion or uncertainty not reported) | Comparison group  - ΔBMI (kg/m^2^): -0.12 (dispersion or uncertainty not reported)  - Body fat (%): -0.22 (dispersion or uncertainty not reported)  - ΔSum of 4 skin-folds (mm): -0.89 (dispersion or uncertainty not reported)  - ΔWaist-hip ratio: -0.014 (dispersion or uncertainty not reported) |
| Running-control group difference after 4 months of the change from baseline between groups (net change)  - ΔBMI (kg/m^2^): -0.20 (95%CI -0.43 to 0.03)  - Body fat (%): -1.08 (95%CI -2.71 to 0.54)  - ΔSum of skin-folds (mm): -2.81 (95%CI -6.04 to 0.42)  - ΔWaist-hip ratio: -0.020 (95%CI -0.03 to -0.01)* | |

**Cardiorespiratory outcomes and between groups comparison**

** statistically significant difference between groups*

*^‡^ statistically significant difference within groups (pre-post)*

| Pre-post mean difference 4 months after baseline | |
| --- | --- |
| Running group  - ΔResting heart rate (/min): -3.5 (dispersion or uncertainty not reported)  - ΔSystolic blood pressure (mmHg): -3.4 (dispersion or uncertainty not reported)  - ΔDiastolic blood pressure (mmHg): -2.1 (dispersion or uncertainty not reported) | Comparison group  - ΔResting heart rate (/min): -1.3 (dispersion or uncertainty not reported)  - ΔSystolic blood pressure (mmHg): -5.9 (dispersion or uncertainty not reported)  - ΔDiastolic blood pressure (mmHg): -0.4 (dispersion or uncertainty not reported) |
| Running-control group difference after 4 months of the change from baseline between groups (net change)  - ΔResting heart rate (/min): -4.8 (95%CI -8.4 to -1.1)*  - ΔSystolic blood pressure (mmHg): -2.5 (95%CI -1.3 to 6.3)  - ΔDiastolic blood pressure (mmHg): -2.4 (95%CI -6.8 to 1.8) | |

**Blood serum concentration outcomes and between groups comparison**

** statistically significant difference between groups*

*^‡^ statistically significant difference within groups (pre-post)*

| Pre-post mean difference 4 months after baseline | |
| --- | --- |
| Running group  - ΔTotal cholesterol (mmol/l): -0.38 (dispersion or uncertainty not reported)  - ΔHDL (mmol/l): -0.08 (dispersion or uncertainty not reported)  - ΔTotal triglycerides (mmol/l): -0.28 (dispersion or uncertainty not reported)  - ΔHDL/total cholesterol ratio: -0.031 (dispersion or uncertainty not reported) | Comparison group  - ΔTotal cholesterol (mmol/l): -0.37 (dispersion or uncertainty not reported)  - ΔHDL (mmol/l): -0.04 (dispersion or uncertainty not reported)  - ΔTotal triglycerides (mmol/l): -0.07 (dispersion or uncertainty not reported)  - ΔHDL/total cholesterol ratio: -0.008 (dispersion or uncertainty not reported) |
| Running-control group difference after 4 months of the change from baseline between groups (net change)  - ΔTotal cholesterol (mmol/l): -0.01 (95%CI -0.48 to 0.45)  - ΔHDL (mmol/l): -0.12 (95%CI -0.00 to 0.22)*  - ΔTotal triglycerides (mmol/l): -0.21 (95%CI -0.54 to 0.12)  - ΔHDL/total cholesterol ratio: -0.023 (95%CI -0.00 to 0.05) | |

**More publications related to this study**

| Marti B, Suter E, Riesen WF, Tschopp A, Wanner HU, Gutzwiller F. Effects of long-term, self-monitored exercise on the serum lipoprotein and apolipoprotein profile in middle-aged men. Atherosclerosis. 1990;81(1):19-31. |
| --- |
| Suter E, Marti B. Little effect of long-term, self-monitored exercise on serum lipid levels in middle-aged women. The Journal of sports medicine and physical fitness. 1992;32(4):400-11. |

**Reference**

| Williams PT, Albers JJ, Krauss RM, Wood PD. Associations of lecithin: cholesterol acyltransferase (LCAT) mass concentrations with exercise, weight loss, and plasma lipoprotein subfraction concentrations in men. Atherosclerosis. 1990;82(1-2):53-8. |
| --- |

**Objectives**

| 1- To compare changes in lecithin cholesterol acyltransferase (LCAT) concentrations in men who participated in a 1-year running program and in non-exercising controls;  2- To correlate l-year changes in LCAT concentrations with distance run, improved fitness, weight loss, lipids, lipoprotein and apolipoproteins within the exercise group. |
| --- |

**Description of participants included in the study**

| - Participants randomised: n=81 sedentary men (30-55 years old).  - Participants included in the analysis, n=72:   - Running group: n=42. - Control group: n=30.   - The article refers to other study to addition information (*Wood PD, Haskell WL, Blair SN, Williams PT, Krauss RM, Lindgren FT, et al. Increased exercise level and plasma lipoprotein concentrations: a one-year, randomised, controlled study in sedentary, middle-aged men. Metabolism: clinical and experimental. 1983;32(1):31-9*). |
| --- |

**Follow-up period and time-points after baseline**

| - Follow-up: 1 year.  - Time-points: baseline and 1 year after baseline. |
| --- |

**Intervention (running program)**

| Running group  - Duration: 1 year.  - Frequency: 3-5 times/week.  - Description: supervised exercise program involving calisthenics and muscle stretching, a 5-min warm-up of walking or slow jogging, a 25 min work phase of running at 70-85% of capacity, as determined during baseline, and a 5-min cool-down phase. After 2-3 weeks, participants were asked to add a 4^th^ day of exercise, and by the 8-10^th^ week, a 5^th^ day and longer sessions were recommended. Exercise intensity during the work phase was maintained at 70-85% of capacity by pulse rate monitoring. |
| --- |

**Comparison group**

| Control group  - To remain sedentary for the year. |
| --- |

**Body composition outcomes and between groups comparison**

** statistically significant difference between groups*

*^‡^ statistically significant difference within groups (pre-post)*

| Pre-post mean difference 1 year after baseline | |
| --- | --- |
| Running group  - ΔBody weight (kg): -1.89 (SD 3.84)* | Control group  - ΔBody weight (kg): 0.56 (SD 3.87) |

**Cardiorespiratory outcomes and between groups comparison**

** statistically significant difference between groups*

*^‡^ statistically significant difference within groups (pre-post)*

| Pre-post mean difference 1 year after baseline | |
| --- | --- |
| Running group  - ΔVO_2_max (ml/kg/min): 7.52 (SD 8.38)*** | Control group  - ΔVO_2_max (ml/kg/min): -1.43 (SD 4.05) |

**Blood serum concentration outcomes and between groups comparison**

** statistically significant difference between groups*

*^‡^ statistically significant difference within groups (pre-post)*

*LCTA: lecithin:cholesterol acyltransferase*

| Pre-post mean difference 1 year after baseline | |
| --- | --- |
| Running group  - ΔLCTA (μg/ml): -0.19 (SD 0.64)  - ΔTriglycerides (mg/dl): -10.79 (SD 41.51)  - Total cholesterol (mg/dl): -5.51 (SD 18.91)  - ΔHDL (mg/dl): 2.08 (SD 8.25)  - ΔLDL (mg/dl): -5.76 (SD 17.10)  - ΔVLDL (mg/dl): -1.83 (SD 10.15)  - ΔApolipoprotein A-I (mg/dl): 4.62 (SD 12.86)  - ΔApolipoprotein A-II (mg/dl): -1.16 (SD 6.32)  - ΔApolipoprotein B (mg/dl): -1.28 (SD 12.87)  - ΔSerum mass HDL_2_ (mg/dl): 18.02 (SD 37.18)  - ΔSerum mass HDL_3_ (mg/dl): 3.49 (SD 31.30)  - ΔSerum mass small LDL (mg/dl): -6.81 (SD 53.87)  - ΔSerum mass large LDL (mg/dl): 12.97 (SD 40.21)  - ΔSerum mass IDL (mg/dl): 0.85 (SD 14.27)  - ΔSerum mass VLDL (mg/dl): 7.91 (SD 47.04)  - ΔLDL-peak flotation rate (S_f_): 0.17 (SD 0.75) | Control group  - ΔLCTA (μg/ml): 0.08 (SD 0.94)  - ΔTriglycerides (mg/dl): 3.77 (SD 37.91)  - Total cholesterol (mg/dl): 1.98 (SD 25.85)  - ΔHDL (mg/dl): 0.22 (SD 6.02)  - ΔLDL (mg/dl): 1.72 (SD 20.90)  - ΔVLDL (mg/dl): 0.05 (SD 10.29)  - ΔApolipoprotein A-I (mg/dl): 2.97 (SD 12.10)  - ΔApolipoprotein A-II (mg/dl): -0.71 (SD 5.71)  - ΔApolipoprotein B (mg/dl): 0.04 (SD 12.07)  - ΔSerum mass HDL_2_ (mg/dl): 3.25 (SD 25.76)  - ΔSerum mass HDL_3_ (mg/dl): 0.22 (SD 33.43)  - ΔSerum mass small LDL (mg/dl): 7.13 (SD 39.47)  - ΔSerum mass large LDL (mg/dl): 14.01 (SD 41.26)  - ΔSerum mass IDL (mg/dl): 3.77 (SD 13.98)  - ΔSerum mass VLDL (mg/dl): 16.38 (SD 46.96)  - ΔLDL-peak flotation rate (S_f_): -0.06 (SD 0.65) |

**More publications related to this study**

| Williams PT, Wood PD, Krauss RM, Haskell WL, Vranizan KM, Blair SN, et al. Does weight loss cause the exercise-induced increase in plasma high density lipoproteins? Atherosclerosis. 1983;47(2):173-85. |
| --- |
| Williams PT, Krauss RM, Vranizan KM, Albers JJ, Terry RB, Wood PD. Effects of exercise-induced weight loss on low density lipoprotein subfractions in healthy men. Arteriosclerosis. 1989;9(5):623-32. |
| Wood PD, Haskell WL, Blair SN, Williams PT, Krauss RM, Lindgren FT, et al. Increased exercise level and plasma lipoprotein concentrations: a one-year, randomised, controlled study in sedentary, middle-aged men. Metabolism: clinical and experimental. 1983;32(1):31-9. |
| Williams PT, Wood PD, Haskell WL, Vranizan K. The effects of running mileage and duration on plasma lipoprotein levels. JAMA : the journal of the American Medical Association. 1982;247(19):2674-9. |

**Reference**

| Williams PT, Krauss RM, Vranizan KM, Wood PD. Changes in lipoprotein subfractions during diet-induced and exercise-induced weight loss in moderately overweight men. Circulation. 1990;81(4):1293-304. |
| --- |

**Objectives**

| To compare the 1-year changes in lipoprotein subfractions in men assigned to one of the following three experimental conditions: weight loss by exercise (primarily, running), weight loss by calorie restriction, and control. |
| --- |

**Description of participants included in the study**

| - Participants randomised: n=155 sedentary men aged 30-59 years.  - Participants included in the analysis:   - Running group: - n=45 for VO2max. - n=46 for other variables. - Diet group: - n=41 for VO2max. - n=42 for other variables. - Control group: - n=40 for VO2max. - n=42 for other variables. |
| --- |

**Follow-up period and time-point assessments after baseline**

| - Follow-up: 1 year.  - Time-points: baseline, 7 months and 1 year after baseline. |
| --- |

**Intervention (running program)**

| Running group  - Duration: 1 year.  - Frequency: 3-5 times/week.  - Description: the target was to reduce the men's body fat by one third over a 9-month period. Participants were asked to begin calisthenics and to walk, jog, or run for 25 min, 3 times/week at 60-80% of maximal heart rate. The periods of continuous jogging were increased to 40-50 minutes, 5 days/week. |
| --- |

**Comparison group**

| Diet group  - Duration: 1 year.  - Description: the target was to reduce the men's body fat by one third over a 9-month period. Participants were asked to reduce total calorie intake without changing the proportions of fat, carbohydrates, protein, or alcohol consumed. Their diets were individually prescribed, assuming that a 7,762-kcal reduction in energy intake would produce a 1-kg fat loss. | Control group  - Participants were asked to remain sedentary and to not change their diets. |
| --- | --- |

**Body composition outcomes and between groups comparison**

** statistically significant difference between groups*

^‡^ *statistically significant difference within groups (pre-post)*

| Pre-post mean difference 1 year after baseline | | |
| --- | --- | --- |
| Running group  - ΔFat body mass (kg): -4.15 (SD 3.70)  - ΔLean body mass (kg): 0.11 (SD 2.22) | Diet group  - ΔFat body mass (kg): -5.93 (SD 4.14)  - ΔLean body mass (kg) -1.31 (SD 2.55) | Control group  - ΔFat body mass (kg): not reported  - ΔLean body mass (kg): not reported |
| Running-control group difference after 7 months of the changes from baseline between groups (net change) | | |
| - ΔBMI (kg/m^2^): -0.98 (SE 0.18)* | | |
| Running-diet group difference after 7 months of the changes from baseline between groups (net change) | | |
| - ΔBMI (kg/m^2^): 1.47 (SE 0.23)* | | |
| Running-control group difference after 1 year of the changes from baseline between groups (net change) | | |
| - ΔBMI (kg/m^2^): -1.41 (SE 0.26)* | | |
| Running-diet group difference after 1 year of the changes from baseline between groups (net change) | | |
| - ΔBMI (kg/m^2^): 1.04 (SE 0.27)* | | |

**Cardiorespiratory outcomes and between groups comparison**

** statistically significant difference between groups*

^‡^ *statistically significant difference within groups (pre-post)*

| Running-control group difference after 1 year of the changes from baseline between groups (net change) |
| --- |
| - ΔVO_2_max (ml/kg/min): 6.64 (SE 0.81)*  - ΔVO_2_max (l/min): 0.42 (0.08)* |
| Running-diet group difference after 1 year of the changes from baseline between groups (net change) |
| - ΔVO_2_max (ml/kg/min): 4.15 (SE 1.07)*  - ΔVO_2_max (l/min): 0.44 (0.08)* |

**Blood serum concentration outcomes and between groups comparison**

** statistically significant difference between groups*

^‡^ *statistically significant difference within groups (pre-post)*

*IDL: intermediate density lipoprotein*

| Running-control group difference after 7 months of the changes from baseline between groups (net change) |
| --- |
| - ΔHDL_2_ (mg/dl): 16.5 (SE 7.1)*  - ΔHDL_3_ (mg/dl): 11.1 (SE 6.6)  - ΔSmall LDL (mg/dl): -22.5 (SE 11.3)*  - ΔLarge LDL (mg/dl): 14.9 (SE 7.5)  - ΔIDL (mg/dl): -1.0 (SE 2.7)  - ΔVLDL (mg/dl): -24.9 (SE 12.7)  - ΔLDL peak flotation rate: 0.47 (SE 0.15)*  - ΔLDL peak particle diameter: 3.43 (SE 1.40)* |
| Running-diet group difference after 7 months of the changes from baseline between groups (net change) |
| - ΔHDL_2_ (mg/dl): 0.1 (SE 7.2)  - ΔHDL_3_ (mg/dl): -1.7 (SE 6.9)  - ΔSmall LDL (mg/dl): -12.7 (SE 20.0)  - ΔLarge LDL (mg/dl): 2.1 (SE 8.0)  - ΔIDL (mg/dl): 3.9 (SE 3.4)  - ΔVLDL (mg/dl): 6.4 (SE 13.6)  - ΔLDL peak flotation rate: 0.01 (SE 0.16)  - ΔLDL peak particle diameter: -0.25 (SE 1.42) |
| Running-control group difference after 1 year of the changes from baseline between groups (net change) |
| - ΔHDL_2_ (mg/dl): 12.7 (SE 5.4)*  - ΔHDL_3_ (mg/dl): 16.7 (SE 6.8)*  - ΔSmall LDL (mg/dl): -16.2 (SE 9.3)  - ΔLarge LDL (mg/dl): 5.4 (SE 7.4)  - ΔIDL (mg/dl): -0.9 (SE 2.8)  - ΔVLDL (mg/dl): -27.3 (SE 11.4)*  - ΔLDL peak flotation rate: 0.19 (SE 0.14)  - ΔLDL peak particle diameter: 2.36 (SE 1.33)* |
| Running-diet group difference after 1 year of the changes from baseline between groups (net change) |
| - ΔHDL_2_ (mg/dl): 0.8 (SE 5.5)  - ΔHDL_3_ (mg/dl): -2.7 (SE 6.8)  - ΔSmall LDL (mg/dl): 11.0 (SE 10.7)  - ΔLarge LDL (mg/dl): -1.9 (SE 7.6)  - ΔIDL (mg/dl): 4.7 (SE 3.0)  - ΔVLDL (mg/dl): -1.7 (SE 12.9)  - ΔLDL peak flotation rate: -0.10 (SE 0.16)  - ΔLDL peak particle diameter: -0.83 (SE 1.44) |

**More publications related to this study**

| Wood PD, Stefanick ML, Dreon DM, Frey-Hewitt B, Garay SC, Williams PT, et al. Changes in plasma lipids and lipoproteins in overweight men during weight loss through dieting as compared with exercise. The New England journal of medicine. 1988;319(18):1173-9. |
| --- |
| Williams PT, Krauss RM, Vranizan KM, Albers JJ, Wood PD. Effects of weight-loss by exercise and by diet on apolipoproteins A-I and A-II and the particle-size distribution of high-density lipoproteins in men. Metabolism: clinical and experimental. 1992;41(4):441-9. |

**Reference**

| Moses J, Steptoe A, Mathews A, Edwards S. The effects of exercise training on mental well-being in the normal population: a controlled trial. Journal of psychosomatic research. 1989;33(1):47-61. |
| --- |

**Objectives**

| To compare the effects of two aerobic training programs of differing intensities on mood and mental well-being with those of a credible attention-placebo condition. |
| --- |

**Description of participants included in the study**

| - Participants randomised: n=109 adults with low levels of habitual activity.  - Source: local residents (London).  - Participants included in the analysis, n=75 after 10 weeks from baseline, n=58 after 3 months from baseline:   - High exercise group: - After 10 weeks: n=18, mean age of 37.7 years (dispersion or uncertainty nor reported), 6 males and 12 females. - After 3 months: n=16 - Moderate exercise group: - After 10 weeks: n=19, mean age of 39.1 years (dispersion or uncertainty nor reported), 4 males and 15 females. - After 3 months: n=17 - Attention-placebo group: - After 10 weeks: n=18, mean age of 39.9 years (dispersion or uncertainty nor reported), 5 males and 13 females. - After 3 months: n=15 - Waiting list group: - After 10 weeks: n=20, mean age of 39.4 years (dispersion or uncertainty nor reported), 5 males and 15 females. - After 3 months: n=10 |
| --- |

**Follow-up period and time-point assessments after baseline**

| - Follow-up: 10 weeks.  - Time-points: baseline, 10 weeks and 3 months after baseline. |
| --- |

**Intervention (running program)**

| High exercise group  - Duration: 10 weeks.  - Frequency: 4 times/week.  - Description: 1 supervised and 3 unsupervised walking and/or jogging sessions/week. Each was structured so that participants entered the schedule at a level appropriate for their initial fitness and progressed towards more demanding levels over the program. Sessions included warm-up and coo-down exercises lasting 5-10 min, and a walk-jog program involving continuous exercise of 30 min at an intensity sufficient to elevate HR to 70-75% of maximum heart rate. | Moderate exercise group  - Duration: 10 weeks.  - Frequency: 4 times/week.  - Description: 1 supervised and 3 unsupervised walking and/or jogging sessions/week. Each was structured so that participants entered the schedule at a level appropriate for their initial fitness and progressed towards more demanding levels over the program. Sessions included warm-up and coo-down exercises lasting 5-10 min, and a walking or jogging program involving continuous exercise of 20 min at an intensity sufficient to elevate HR to 60% of maximum heart rate. |
| --- | --- |

**Comparison group**

| Attention-placebo group  - Duration: 10 weeks.  - Description: participants performed strength, mobility and flexibility exercises during the training phase of each session, and carried out slow, discontinuous exercise for at least 30 min at an intensity that did not elevate HR above 50% of maximum heart rate. | Waiting list group  - Control group without any structure exercise during the study. |
| --- | --- |

**Body composition outcomes and between groups comparison**

** statistically significant difference between high exercise group and waiting list group*

^†^ *statistically significant difference between high exercise group and attention-placebo group*

^‡^ *statistically significant difference within groups (pre-post)*

| p.s.: Results for body weight and percentage body fat were not reported, however there were no statistically significant difference between or within groups. |
| --- |

**Cardiorespiratory outcomes and between groups comparison**

** statistically significant difference between high exercise group and waiting list group*

^†^ *statistically significant difference between high exercise group and attention-placebo group*

^‡^ *statistically significant difference within groups (pre-post)*

| Pre-post mean difference after 10 weeks from baseline | | | |
| --- | --- | --- | --- |
| High exercise group  - ΔVO_2_max (ml/kg/min): result reported in a graph^†^ | Moderate exercise group  - ΔVO_2_max (ml/kg/min): result reported in a graph | Attention-placebo group  - ΔVO_2_max (ml/kg/min): result reported in a graph | Waiting list group  - ΔVO_2_max (ml/kg/min): result reported in a graph |
| Pre-post mean difference after 3 months from baseline | | | |
| High exercise group  - ΔVO_2_max (ml/kg/min): result reported in a graph | Moderate exercise group  - ΔVO_2_max (ml/kg/min): result reported in a graph | Attention-placebo group  - ΔVO_2_max (ml/kg/min): result reported in a graph | Waiting list group  - ΔVO_2_max (ml/kg/min): result reported in a graph |
| p.s.: Results for exertion rating were not reported, however there were no statistically significant difference between or within groups. | | | |

**Blood serum concentration outcomes and between groups comparison**

** statistically significant difference between high exercise group and waiting list group*

^†^ *statistically significant difference between high exercise group and attention-placebo group*

^‡^ *statistically significant difference within groups (pre-post)*

| Not applicable | Not applicable | Not applicable | Not applicable |
| --- | --- | --- | --- |

**Reference**

| Williams PT, Krauss RM, Vranizan KM, Albers JJ, Terry RB, Wood PD. Effects of exercise-induced weight loss on low density lipoprotein subfractions in healthy men. Arteriosclerosis. 1989;9(5):623-32. |
| --- |

**Objectives**

| To compare the 1-year changes in LDL and VLDL concentrations between runners and sedentary controls. |
| --- |

**Description of participants included in the study**

| - Participants randomised: n=81 sedentary men aged 30-55.  - Participants included in the analysis: n=77 for LDL peak floatation rate; n=76 for lipoproteins; n=73 for body weight; n=70 for lean and fat body mass; n=65 for VO_2_max; n=64 for waist-hip ratio:   - Running group: - n=46 for lipoproteins; - n=45 for LDL peak floatation rate; - n=43 for body weight; - n=40 for lean, fat body mass and skinfolds; - n=38 for VO_2_max; - n=35 for waist-hip ratio and circumference measurements. - Control group: - n=32 for LDL peak floatation rate; - n=30 for lipoproteins, body weight, lean mass, body fat and skinfolds; - n=29 for waist-hip ratio and circumference measurements; - n=27 for VO_2_max. |
| --- |

**Follow-up period and time-point assessments after baseline**

| - Follow-up: 1 year.  - Time-points: baseline and 3, 6, 9 and 12 months after baseline. |
| --- |

**Intervention (running program)**

| Running group  - Duration: 1 year.  - Frequency: 5 times/week.  - Description: participants were encouraged to run 5 days a week, 45 minutes per day after 6 weeks. |
| --- |

**Comparison group**

| Control group  - Participants were asked to remain sedentary throughout the year. |
| --- |

**Body composition outcomes and between groups comparison**

** statistically significant difference between groups*

*^‡^ statistically significant difference within groups (pre-post)*

| Pre-post mean difference 1 year after baseline | |
| --- | --- |
| Running group  - ΔBody weight (kg): -1.9 (SD 3.8)  - ΔLean body mass (kg): -0.2 (SD 2.2)  - ΔBody fat (kg): -1.9 (SD 3.5)  - ΔAbdominal skinfold (mm): -5.3 (SD 8.2)  - ΔAbdominal circumference (cm): -3.0 (SD 3.7)  - ΔThigh skinfold (mm): -3.1 (SD 4.2)  - ΔThigh circumference (cm): -1.9 (SD 1.9)  - ΔWaist-hip circumference ratio (%): -1.3 (SD 2.1) | Control group  - ΔBody weight (kg): 0.6 (SD 3.9)  - ΔLean body mass (kg): -1.4 (SD 2.5)  - ΔBody fat (kg): 1.9 (SD 4.1)  - ΔAbdominal skinfold (mm): -0.2 (SD 6.1)  - ΔAbdominal circumference (cm): -0.1 (SD 3.5)  - ΔThigh skinfold (mm): -0.4 (SD 3.5)  - ΔThigh circumference (cm): -1.0 (SD 2.6)  - ΔWaist-hip circumference ratio (%): 0.2 (SD 2.0) |
| Running-control group difference after 1 year of the change from baseline between groups (net change)  - ΔBody weight (kg): -2.5 (SE 0.9)*  - ΔLean body mass (kg): 1.2 (SE 0.6)*  - ΔBody fat (kg): -3.7 (SE 0.9)*  - ΔAbdominal skinfold (mm): -5.1 (SE 1.7)*  - ΔAbdominal circumference (cm): -2.9 (SE 0.9)*  - ΔThigh skinfold (mm): -2.7 (SE 0.9)*  - ΔThigh circumference (cm): -0.9 (SE 0.6)  - ΔWaist-hip circumference ratio (%): -1.5 (SE 0.5)* | |

**Cardiorespiratory outcomes and between groups comparison**

** statistically significant difference between groups*

*^‡^ statistically significant difference within groups (pre-post)*

| Pre-post mean difference 1 year after baseline | |
| --- | --- |
| Running group  - ΔVO_2_max (ml/kg/min): 7.5 (SD 8.4) | Control group  - ΔVO_2_max (ml/kg/min): -1.4 (SD 4.1) |
| Running-control group difference after 1 year of the change from baseline between groups (net change)  - ΔVO_2_max (ml/kg/min): 9.0 (SE 1.6)* | |

**Blood serum concentration outcomes and between groups comparison**

** statistically significant difference between groups*

*^‡^ statistically significant difference within groups (pre-post)*

*IDL: intermediate density lipoprotein*

| Pre-post mean difference 1 year after baseline | |
| --- | --- |
| Running group  - ΔSmall LDL {S_f_ 0-7} (mg/dl): -8.6 (SD 53.4)  - ΔLarge LDL {S_f_ 7-12} (mg/dl): 10.5 (SD 39.5)  - ΔIDL {S_f_ 12-20} (mg/dl): 0.1 (SD 14.9)  - ΔSmall VLDL {S_f_ 20-60} (mg/dl): 5.6 (21.8)  - ΔLarge VLDL {S_f_ 60-400} (mg/dl): 3.1 (SD 32.8)  - ΔLDL peak floatation rate (S_f_): 0.1 (SD 0.7) | Control group  - ΔSmall LDL {S_f_ 0-7} (mg/dl): 8.2 (SD 38.4)  - ΔLarge LDL {S_f_ 7-12} (mg/dl): 14.7 (SD 40.0)  - ΔIDL {S_f_ 12-20} (mg/dl): 3.9 (SD 14.4)  - ΔSmall VLDL {S_f_ 20-60} (mg/dl): 14.2 (26.8)  - ΔLarge VLDL {S_f_ 60- 400} (mg/dl): 3.7 (SD 43.4)  - ΔLDL peak floatation rate (S_f_): 0.0 (SD 0.6) |
| Running-control group difference after 1 year of the change from baseline between groups (net change)  - ΔSmall LDL {S_f_ 0-7} (mg/dl): -16.8 (SE 10.4)  - ΔLarge LDL {S_f_ 7-12} (mg/dl): -4.2 (SE 9.2)  - ΔIDL {S_f_ 12-20} (mg/dl): -3.8 (SE 3.4)  - ΔSmall VLDL {S_f_ 20-60} (mg/dl): -8.6 (SE 5.3)  - ΔLarge VLDL {S_f_ 60-400} (mg/dl): -0.6 (SE 9.1)  - ΔLDL peak floatation rate (S_f_): 0.2 (SE 0.2) | |

**More publications related to this study**

| Williams PT, Albers JJ, Krauss RM, Wood PD. Associations of lecithin: cholesterol acyltransferase (LCAT) mass concentrations with exercise, weight loss, and plasma lipoprotein subfraction concentrations in men. Atherosclerosis. 1990;82(1-2):53-8. |
| --- |
| Williams PT, Wood PD, Krauss RM, Haskell WL, Vranizan KM, Blair SN, et al. Does weight loss cause the exercise-induced increase in plasma high density lipoproteins? Atherosclerosis. 1983;47(2):173-85. |
| Wood PD, Haskell WL, Blair SN, Williams PT, Krauss RM, Lindgren FT, et al. Increased exercise level and plasma lipoprotein concentrations: a one-year, randomised, controlled study in sedentary, middle-aged men. Metabolism: clinical and experimental. 1983;32(1):31-9. |
| Williams PT, Wood PD, Haskell WL, Vranizan K. The effects of running mileage and duration on plasma lipoprotein levels. JAMA : the journal of the American Medical Association. 1982;247(19):2674-9. |

**Reference**

| Wood PD, Stefanick ML, Dreon DM, Frey-Hewitt B, Garay SC, Williams PT, et al. Changes in plasma lipids and lipoproteins in overweight men during weight loss through dieting as compared with exercise. The New England journal of medicine. 1988;319(18):1173-9. |
| --- |

**Objectives**

| To investigate the effects of two methods for losing body fat weight on the levels of plasma lipids and lipoproteins in overweight sedentary men. |
| --- |

**Description of participants included in the study**

| - Participants randomised: n=155 overweight sedentary men.  - Source: Stanford area, USA.  - Participants included in the analysis:   - Running group: - n=46 for VO_2_max. - n=47 for other variables. Mean age of 44.1 years (SD 7.8). - Diet group: - n=39 for VO_2_max. - n=41 for HDL, HDL_2_, HDL_3_, and total cholesterol/HDL ratio. - n=42 for other variables. Mean age of 44.2 years (SD 8.2). - Control group: - n=39 for VO_2_max. - n=41 for HDL, HDL_2_, HDL_3_, and total cholesterol/HDL ratio. - n=42 for other variables. Mean age of 45.2 years (SD 7.2). |
| --- |

**Follow-up period and time-point assessments after baseline**

| - Follow-up: 1 year.  - Time-points:   - Baseline, 7 months and 1 year after baseline: plasma lipids and lipoprotein cholesterol, weight and body composition. - Baseline and 1 year after baseline: graded maximal treadmill exercise tests. |
| --- |

**Intervention (running program)**

| Running group  - Duration: 1 year.  - Frequency: 3-5 times/week.  - Description: supervised exercise program and individual prescriptions based on estimate of the amount of energy necessary to decrease total body fat progressively by 1/3 over 9 months. During the first 3 months, group exercise sessions consisted of calisthenics and muscle stretching, a 5-min warm-up period of walking, a 25-min work phase of brisk walking or jogging at 60-80% of maximal heart rate, and a 5 min cool-down period. The period of continuous jogging was gradually increased to 40-50 min, and it was encouraged to add 2 more days/week of unsupervised exercise. The exercise level was adjusted (if necessary) to keep weight stable during the final 6-week weight-stabilization period. In the end the participants jogged on average 15.3 km/week (SD 7.4) over the first 7 months, and 18.9 km/week (SD 13.1) over the entire year. Participants were instructed to increase their general activity level by increasing walking distance, bicycling, stair climbing and recreational activities. |
| --- |

**Comparison group**

| Diet group  - Duration: 1 year.  - Description: individual prescriptions designed to reduce baseline total body fat by 1/3 over a 9-month period. Participants met regularly with nutritionists, either individually or as a group, for instructions on diet and reinforcement of behavioural strategies. Food intake was adjusted (if necessary) to keep weight stable during the final 6-week weight-stabilization period. | Control group  - Participants were instructed to maintain their usual diet and exercise patterns throughout the study. |
| --- | --- |

**Body composition outcomes and between groups comparison**

** statistically significant difference between running group and control group*

^†^ *statistically significant difference between running group and diet group*

^‡^ *statistically significant difference within groups (pre-post)*

| Pre-post mean difference 7 months after baseline | | |
| --- | --- | --- |
| Running group  - ΔBody weight (kg): -3.0 (SD 2.8)*^†^  - ΔNon-fat body mass (kg): 0.4 (SD 1.9)^†^  - ΔFat body mass (kg): -3.4 (SD 2.6)*^†^ | Diet group  - ΔBody weight (kg): -7.6 (SD 3.9)  - ΔNon-fat body mass (kg): -1.3 (SD 2.6)  - ΔFat body mass (kg): -6.3 (SD 3.2) | Control group  - ΔBody weight (kg): 0.2 (SD 2.5)  - ΔNon-fat body mass (kg): 0.7 (SD 2.0)  - ΔFat body mass (kg): -0.5 (SD 2.7) |
| Pre-post mean difference 1 year after baseline | | |
| Running group  - ΔBody weight (kg): -4.0 (SD 3.9)*^†^  - ΔNon-fat body mass (kg): 0.1 (SD 2.2)^†^  - ΔFat body mass (kg): -4.1 (SD 3.7)* | Diet group  - ΔBody weight (kg): -7.2 (SD 3.7)  - ΔNon-fat body mass (kg): -1.3 (SD 2.6)  - ΔFat body mass (kg): -5.9 (SD 4.1) | Control group  - ΔBody weight (kg): 0.6 (SD 3.7)  - ΔNon-fat body mass (kg): 0.8 (SD 2.0)  - ΔFat body mass (kg): -0.3 (SD 3.3) |

**Cardiorespiratory outcomes and between groups comparison**

** statistically significant difference between running group and control group*

^†^ *statistically significant difference between running group and diet group*

^‡^ *statistically significant difference within groups (pre-post)*

| Pre-post mean difference 1 year after baseline | | |
| --- | --- | --- |
| Running group  - ΔVO_2_max (ml/kg/min): 4.1 (SD 5.9)*^†^ | Diet group  - ΔVO_2_max (ml/kg/min): 0.0 (SD 3.2) | Control group  - ΔVO_2_max (ml/kg/min): -2.4 (SD 3.2) |

**Blood serum concentration outcomes and between groups comparison**

** statistically significant difference between running group and control group*

^†^ *statistically significant difference between running group and diet group*

^‡^ *statistically significant difference within groups (pre-post)*

| Pre-post mean difference 7 months after baseline | | |
| --- | --- | --- |
| Running group  - ΔTriglycerides (mmol/l): -0.25 (SD 0.61)*  - ΔCholesterol (mmol/l): -0.21 (SD 0.63)  - ΔLDL (mmol/l): -0.11 (SD 0.54)  - ΔHDL (mmol/l): 0.09 (SD 0.21)*  - ΔHDL_2_ (mmol/l): 0.06 (SD 0.18)*  - ΔHDL_3_ (mmol/l): 0.03 (SD 0.11)  - ΔTotal cholesterol/HDL ratio: -0.49 (SD 0.90)* | Diet group  - ΔTriglycerides (mmol/l): -0.40 (SD 0.61)  - ΔCholesterol (mmol/l): -0.40 (SD 0.55)  - ΔLDL (mmol/l): -0.27 (SD 0.59)  - ΔHDL (mmol/l): 0.06 (SD 0.14)  - ΔHDL_2_ (mmol/l): 0.06 (SD 0.11)  - ΔHDL_3_ (mmol/l): 0.00 (SD 0.11)  - ΔTotal cholesterol/HDL ratio: -0.67 (SD 0.87) | Control group  - ΔTriglycerides (mmol/l): -0.01 (SD 0.51)  - ΔCholesterol (mmol/l): -0.21 (SD 0.48)  - ΔLDL (mmol/l): -0.15 (SD 0.46)  - ΔHDL (mmol/l): 0.00 (SD 0.10)  - ΔHDL_2_ (mmol/l): 0.00 (SD 0.06)  - ΔHDL_3_ (mmol/l): 0.01 (SD 0.08)  - ΔTotal cholesterol/HDL ratio: -0.18 (SD 0.60) |
| Pre-post mean difference 1 year after baseline | | |
| Running group  - ΔTriglycerides (mmol/l): -0.16 (SD 0.53)*  - ΔCholesterol (mmol/l): -0.25 (SD 0.64)  - ΔLDL (mmol/l): -0.25 (SD 0.61)  - ΔHDL (mmol/l): 0.11 (SD 0.15)*  - ΔHDL_2_ (mmol/l): 0.07 (SD 0.12)*  - ΔHDL_3_ (mmol/l): 0.03 (SD 0.12)*  - ΔTotal cholesterol/HDL ratio: -0.62 (SD 0.86)* | Diet group  - ΔTriglycerides (mmol/l): -0.27 (SD 0.72)  - ΔCholesterol (mmol/l): -0.36 (SD 0.56)  - ΔLDL (mmol/l): -0.31 (SD 0.64)  - ΔHDL (mmol/l): 0.12 (SD 0.16)  - ΔHDL_2_ (mmol/l): 0.07 (SD 0.10)  - ΔHDL_3_ (mmol/l): 0.05 (SD 0.12)  - ΔTotal cholesterol/HDL ratio: -0.79 (SD 0.69) | Control group  - ΔTriglycerides (mmol/l): 0.08 (SD 0.60)  - ΔCholesterol (mmol/l): -0.23 (SD 0.65)  - ΔLDL (mmol/l): -0.21 (SD 0.67)  - ΔHDL (mmol/l): -0.02 (SD 0.11)  - ΔHDL_2_ (mmol/l): 0.01 (SD 0.07)  - ΔHDL_3_ (mmol/l): -0.02 (SD 0.08)  - ΔTotal cholesterol/HDL ratio: -0.04 (SD 0.84) |

**More publications related to this study**

| Williams PT, Krauss RM, Vranizan KM, Wood PD. Changes in lipoprotein subfractions during diet-induced and exercise-induced weight loss in moderately overweight men. Circulation. 1990;81(4):1293-304. |
| --- |
| Williams PT, Krauss RM, Vranizan KM, Albers JJ, Wood PD. Effects of weight-loss by exercise and by diet on apolipoproteins A-I and A-II and the particle-size distribution of high-density lipoproteins in men. Metabolism: clinical and experimental. 1992;41(4):441-9. |

**Reference**

| Juneau M, Rogers F, De Santos V, Yee M, Evans A, Bohn A, et al. Effectiveness of self-monitored, home-based, moderate-intensity exercise training in middle-aged men and women. The American journal of cardiology. 1987;60(1):66-70. |
| --- |

**Objectives**

| To evaluate the physiologic effects of a 6-month, self-monitored, home-based, moderate-intensity (65-77% of peak heart rate) exercise training program in 120 healthy, sedentary, middle-aged men and women. |
| --- |

**Description of participants included in the study**

| - Participants randomised: n=120 employees who had not been involved in regular, vigorous physical activity in the prior 6 months, i.e., had not walked or jogged 20 or more min continuously 3 or more times/week or participated in an active sport more than once a week.  - Source: Lockheed Missiles and Space Corporation in Sunnyvale California, USA.  - Participants included in the analysis, n=120 (mean age of 49 years, SD 6, for men and 47 years, SD 5, for females) for 12 weeks after baseline, n=113 for 24 weeks after baseline:   - Running group: - n=52 for body weight, lean body mass and body fat at 12 weeks after baseline, 28 males and 24 females. - n=60 for other variables at 12 weeks after baseline, 30 males and 30 females. - n=52 for body weight, lean body mass and body fat at 24 weeks after baseline, 28 males and 24 females. - n=57 for other variables at 24 weeks after baseline, 29 males and 28 females. - Control group: - n=51 for body weight, lean body mass and body fat at 12 weeks after baseline, 25 males and 26 females. - n=60 for other variables at 12 weeks after baseline, 30 males and 30 females. - n=51 for body weight, lean body mass and body fat at 24 weeks after baseline, 25 males and 26 females. - n=56 for other variables at 24 weeks after baseline, 28 males and 28 females. |
| --- |

**Follow-up period and time-point assessments after baseline**

| - Follow-up: 24 weeks.  - Time-points: baseline, 12 and 24 weeks after baseline. |
| --- |

**Intervention (running program)**

| Running group  - Duration: 24 weeks.  - Frequency: 5 times/week.  - Description: participants were oriented to the home-based training program by the project staff in a 15 min face-to-face session, which followed participants’ viewing of a 15 min videotape on exercise training. Beginning with the first session, all exercise training was carried out at home. Prescribed, moderate intensity exercise training occurred within a 20-beat range of heart rates above 65% of the peak heart rate. The duration of walking or slow jogging within the training heart rate range was tailored to produce an energy expenditure of 4 kcal/kg for each training session. |
| --- |

**Comparison group**

| Control group  - Not described. |
| --- |

**Body composition outcomes and between groups comparison**

** statistically significant difference between groups*

^‡^ *statistically significant difference within groups (pre-post)*

| Mean 12 weeks after baseline for males | |
| --- | --- |
| Running group  - Body weight (kg): 78.7 (SD 10)  - Lean body mass (kg): 60.1 (SD 7)  - Body fat (%): 22.0 (SD 5) | Control group  - Body weight (kg): 82.5 (SD 12)  - Lean body mass (kg): 63.5 (SD 9)  - Body fat (%): 22.3 (SD 6) |
| Mean 24 weeks after baseline for males | |
| Running group  - Body weight (kg): 77.9 (SD 10)*  - Lean body mass (kg): 63.9 (SD 13)  - Body fat (%): 20.5 (SD 5) | Control group  - Body weight (kg): 81.1 (SD 12)  - Lean body mass (kg): 67.5 (SD 14)  - Body fat (%): 19.7 (SD 4) |
| Mean 12 weeks after baseline for females | |
| Running group  - Body weight (kg): 64.1 (SD 9)  - Lean body mass (kg): 45.6 (SD 5)  - Body fat (%): 27.5 (SD 7) | Control group  - Body weight (kg): 61.0 (SD 8)  - Lean body mass (kg): 44.4 (SD 5)  - Body fat (%): 28.0 (SD 7) |
| Mean 24 weeks after baseline for females | |
| Running group  - Body weight (kg): 63.4 (SD 8)  - Lean body mass (kg): 46.8 (SD 4)  - Body fat (%): 25.5 (SD 7) | Control group  - Body weight (kg): 60.7 (SD 8)  - Lean body mass (kg): 44.1 (SD 4)  - Body fat (%): 26.5 (SD 7) |

**Cardiorespiratory outcomes and between groups comparison**

** statistically significant difference between groups*

^‡^ *statistically significant difference within groups (pre-post)*

| Mean 12 weeks after baseline for males | |
| --- | --- |
| Running group  - Rest heart rate (bpm): 71 (SD 7)  - Submax heart rate (bpm): 127 (SD 15)  - VO_2_max (ml/kg/min): 35.8 (SD 3.9)* | Control group  - Rest heart rate (bpm): 69 (SD 9)  - Submax heart rate (bpm): 125 (SD 14)  - VO_2_max (ml/kg/min): 32.6 (SD 5.0) |
| Mean 24 weeks after baseline for males | |
| Running group  - Rest heart rate (bpm): 68 (SD 10)  - Submax heart rate (bpm): 127 (SD 12)  - VO_2_max (ml/kg/min): 36.2 (SD 3.7)* | Control group  - Rest heart rate (bpm): 67 (SD 8)  - Submax heart rate (bpm): 127 (SD 13)  - VO_2_max (ml/kg/min): 33.0 (SD 4.4) |
| Mean 12 weeks after baseline for females | |
| Running group  - Rest heart rate (bpm): 70 (SD 7)  - Submax heart rate (bpm): 134 (SD 17)  - VO_2_max (ml/kg/min): 28.2 (SD 4.7)* | Control group  - Rest heart rate (bpm): 68 (SD 10)  - Submax heart rate (bpm): 134 (SD 16)  - VO_2_max (ml/kg/min): 25.9 (SD 4.9) |
| Mean 24 weeks after baseline for females | |
| Running group  - Rest heart rate (bpm): 67 (SD 8)  - Submax heart rate (bpm): 134 (SD 15)  - VO_2_max (ml/kg/min): 28.2 (SD 5.2)* | Control group  - Rest heart rate (bpm): 66 (SD 8)  - Submax heart rate (bpm): 133 (SD 17)  - VO_2_max (ml/kg/min): 26.0 (SD 3.9) |
| Pre-post mean difference 12 weeks after baseline for males | |
| Running group  - VO_2_max (%): 13.7 (SD 0.1)* | Control group  - VO_2_max (%): not reported |
| Pre-post mean difference 24 weeks after baseline for males | |
| Running group  - VO_2_max (%): 14.7 (SD 0.1)* | Control group  - VO_2_max (%): not reported |
| Pre-post mean difference 12 weeks after baseline for females | |
| Running group  - VO_2_max (%): 9.2 (SD 0.1)* | Control group  - VO_2_max (%): not reported |
| Pre-post mean difference 24 weeks after baseline for females | |
| Running group  - VO_2_max (%): 9.4 (SD 0.1)* | Control group  - VO_2_max (%): not reported |
| p.s.: The blood pressure results were not described, however there was no statistically significant difference between groups for systolic blood pressure. | |

**Blood serum concentration outcomes and between groups comparison**

** statistically significant difference between groups*

^‡^ *statistically significant difference within groups (pre-post)*

| Mean 24 weeks after baseline for males | |
| --- | --- |
| Running group  - Cholesterol (mg/dl): 219 (SD 31)  - LDL (mg/dl): 146 (SD 32)  - HDL (mg/dl): 46 (SD 13)  - Triglycerides (mg/dl): 140 (SD 102) | Control group  - Cholesterol (mg/dl): 214 (SD 37)  - LDL (mg/dl): 143 (SD 28)  - HDL (mg/dl): 49 (SD 12)  - Triglycerides (mg/dl): 118 (SD 56) |
| Mean 24 weeks after baseline for females | |
| Running group  - Cholesterol (mg/dl): 223 (SD 46)  - LDL (mg/dl): 138 (SD 44)  - HDL (mg/dl): 68 (SD 23)  - Triglycerides (mg/dl): 90 (SD 58) | Control group  - Cholesterol (mg/dl): 207 (SD 36)  - LDL (mg/dl): 131 (SD 37)  - HDL (mg/dl): 60 (SD 12)  - Triglycerides (mg/dl): 82 (SD 41) |

**Reference**

| Allen D, Freund BJ, Wilmore JH. Interaction of test protocol and horizontal run training on maximal oxygen uptake. Medicine and science in sports and exercise. 1986;18(5):581-7. |
| --- |

**Objectives**

| To analyse the interaction between a subject’s VO_2_max on an inclined protocol vs. a horizontal protocol before and after the subject was trained exclusively on a flat terrain. |
| --- |

**Description of participants included in the study**

| - Participants randomised: n=30 college-age males who were not engaged in aerobic conditioning activities.  - Participants included in the analysis, n=27.   - Running group: n=17, mean age of 23.7 years (SD 0.79). - Control group: n=10, mean age of 22.2 years (SD 0.81). |
| --- |

**Follow-up period and time-point assessments after baseline**

| - Follow-up: 12 weeks.  - Time-points: baseline and 12 weeks after baseline. |
| --- |

**Intervention (running program)**

| Running group  - Duration: 12 weeks.  - Frequency: 4 times/week.  - Description: training in flat terrain (grassy, paved and track with no more than 2° of inclination), 37 min/day and in average 4.5 miles each session. Participants maintained their individual heart rate within 65-85% of maximum heart rate reserve. 40% of the training sessions incorporated some form of speed-play or interval training. |
| --- |

**Comparison group**

| Control group  - Participants maintained sedentary. |
| --- |

**Body composition outcomes and between groups comparison**

** statistically significant difference groups*

^‡^ *statistically significant difference within groups (pre-post)*

| Mean 12 weeks after baseline | |
| --- | --- |
| Running group  - Body weight (kg): 72.7 (SE 2.47)^‡^ | Control group  - Body weight (kg): 73.2 (SE 3.22) |
| Pre-post mean difference 12 weeks after baseline | |
| Running group  - ΔBody weight (kg): -1.2 (dispersion or uncertainty not reported)^‡^ | Control group  - ΔBody weight (kg): -0.3 (dispersion or uncertainty not reported) |

**Cardiorespiratory outcomes and between groups comparison**

** statistically significant difference groups*

^‡^ *statistically significant difference within groups (pre-post)*

| Mean 12 weeks after baseline | |
| --- | --- |
| Running group  - Resting heart rate (bpm): 66.5 (SE 2.30)  - VO_2_max inclined protocol (l/min): 4.22 (SE 0.12)*^‡^  - VO_2_max horizontal protocol (l/min): 4.07 (SE 0.12)*^‡^  - VO_2_max inclined protocol (ml/kg/min): reported in a graph*^‡^  - VO_2_max horizontal protocol (ml/kg/min): reported in a graph*^‡^  - Maximal ventilation inclined protocol (l/min): 170.3 (SE 4.10)*^‡^  - Maximal ventilation horizontal protocol (l/min): 159.5 (SE 4.07)*^‡^  - Respiratory exchange ratio inclined protocol: 1.20 (SE 0.02)^‡^  - Respiratory exchange ratio horizontal protocol: 1.11 (SE 0.01) | Control group  - Resting heart rate (bpm): 71.8 (SE 2.99)  - VO_2_max inclined protocol (l/min): 3.75 (SE 0.16)  - VO_2_max horizontal protocol (l/min): 3.68 (SE 0.16)^‡^  - VO_2_max inclined protocol (ml/kg/min): reported in a graph  - VO_2_max horizontal protocol (ml/kg/min): reported in a graph^‡^  - Maximal ventilation inclined protocol (l/min): 148.8 (SE 5.35)  - Maximal ventilation horizontal protocol (l/min): 138.6 (SE 5.31)  - Respiratory exchange ratio inclined protocol: 1.21 (SE 0.03)  - Respiratory exchange ratio horizontal protocol: 1.13 (SE 0.01) |
| Pre-post mean difference 12 weeks after baseline | |
| Running group  - ΔResting heart rate (bpm): -3.5  - ΔVO_2_max inclined protocol (l/min): 0.27*^‡^  - ΔVO_2_max horizontal protocol (l/min): 0.27*^‡^  - ΔMaximal ventilation inclined protocol (l/min): 10.1*^‡^  - ΔMaximal ventilation horizontal protocol (l/min): 8.6*^‡^  - ΔRespiratory exchange ratio inclined protocol: -0.03^‡^  - ΔRespiratory exchange ratio horizontal protocol: -0.01 | Control group  - ΔResting heart rate (bpm): 2.3  - ΔVO_2_max inclined protocol (l/min): -0.09  - ΔVO_2_max horizontal protocol (l/min): -0.15^‡^  - ΔMaximal ventilation inclined protocol (l/min): -3.7  - ΔMaximal ventilation horizontal protocol (l/min): -4.6  - ΔRespiratory exchange ratio inclined protocol: -0.04  - ΔRespiratory exchange ratio horizontal protocol: 0.01 |
| p.s.: Dispersion or uncertainty measures were not reported for the pre-post mean differences. | |

**Blood serum concentration outcomes and between groups comparison**

** statistically significant difference groups*

^‡^ *statistically significant difference within groups (pre-post)*

| Not applicable | Not applicable |
| --- | --- |

**Reference**

| Gossard D, Haskell WL, Taylor CB, Mueller JK, Rogers F, Chandler M, et al. Effects of low- and high-intensity home-based exercise training on functional capacity in healthy middle-aged men. The American journal of cardiology. 1986;57(6):446-9. |
| --- |

**Objectives**

| To evaluate the effects of 12 weeks of home-based exercise training on peak oxygen consumption (VO_2_max) in healthy sedentary middle-aged men. |
| --- |

**Description of participants included in the study**

| - Participants randomised: n=64 men, mean age of 49 years (6), who had not performed regular, vigorous physical activity in the 3 months before the study (not walked or jogged for 20 min or more, continuously, 3 or more times/week, and had not participated in an active sport more than once per week).  - Participants included in the analysis, n=63:   - High intensity exercise group: n=23. - Low intensity exercise group: n=20. - Control group: n=20. |
| --- |
| p.s.: The unit for the dispersion or uncertainty measure for age was not described. |

**Follow-up period and time-point assessments after baseline**

| - Follow-up: 12 weeks.  - Time-points: baseline, 6 and 12 weeks after baseline. |
| --- |
| p.s.: The results corresponding to follow-up after 6 weeks from baseline were not extracted because 8 weeks or more of running training is an inclusion criterion of the systematic review. |

**Intervention (running program)**

| High intensity exercise group  - Duration: 12 weeks.  - Frequency: 5 times/week.  - Description: participants trained on a 20-beat range of heart rates above a threshold of 75% (128-148 bpm) of the peak heart rate. Participants jogged for an average of 37 minutes (7, unit of dispersion or uncertainty not described) per session in order to expend at least 300 kcal during each session, equivalent to an average caloric expenditure of 8.1 kcal/min. | Low intensity exercise group  - Duration: 12 weeks.  - Frequency: 5 times/week.  - Description: participants trained on a 26-beat range of heart rates above a threshold of 60% (102-122 bpm) of the peak heart rate. Participants were given a duration of walking or slow jogging sufficient to expend at least 300 kcal per training session. This produced an average training duration of 52 minutes (5, unit of dispersion or uncertainty not described), equivalent to an average caloric expenditure of 5.8 kcal/min. |
| --- | --- |

**Comparison group**

| Control group  - Not described. |
| --- |

**Body composition outcomes and between groups comparison**

** statistically significant difference between high intensity group and control group*

^†^ *statistically significant difference between low intensity group and control group*

^§^ *statistically significant difference between high intensity group and low intensity group*

^‡^ *statistically significant difference within groups (pre-post)*

| Not applicable | Not applicable | Not applicable |
| --- | --- | --- |

**Cardiorespiratory outcomes and between groups comparison**

** statistically significant difference between high intensity group and control group*

^†^ *statistically significant difference between low intensity group and control group*

^§^ *statistically significant difference between high intensity group and low intensity group*

^‡^ *statistically significant difference within groups (pre-post)*

| Mean 12 weeks after baseline | | |
| --- | --- | --- |
| High intensity group  - Resting heart rate (bpm): 66 (13)  - Submax heart rate (bpm): 113 (15)  - VO_2_ at 6 METs (ml/kg/min): 20.6 (3.1)  - VO_2_max (ml/kg/min): 37.6 (4.8)* | Low intensity group  - Resting heart rate (bpm): 70 (13)  - Submax heart rate (bpm): 119 (14)  - VO_2_ at 6 METs (ml/kg/min): 20.7 (2.4)  - VO_2_max (ml/kg/min): 36.0 (4.6)^†^ | Control group  - Resting heart rate (bpm): 74 (9)  - Submax heart rate (bpm): 123 (13)  - VO_2_ at 6 METs (ml/kg/min): 19.4 (2.7)  - VO_2_max (ml/kg/min): 31.1 (4.5) |
| p.s.: The unit for the dispersion or uncertainty measure was not described. | | |

**Blood serum concentration outcomes and between groups comparison**

** statistically significant difference between high intensity group and control group*

^†^ *statistically significant difference between low intensity group and control group*

^§^ *statistically significant difference between high intensity group and low intensity group*

^‡^ *statistically significant difference within groups (pre-post)*

| Not applicable | Not applicable | Not applicable |
| --- | --- | --- |

**Reference**

| Hagan RD, Upton SJ, Wong L, Whittam J. The effects of aerobic conditioning and/or caloric restriction in overweight men and women. Medicine and science in sports and exercise. 1986;18(1):87-94. |
| --- |

**Objectives**

| To evaluate the effects of aerobic conditioning and a 1200 kcal/day diet on body weight, body composition, maximal aerobic power, serum lipids and cholesterol lipoproteins fractions in overweight adult men and women. |
| --- |

**Description of participants included in the study**

| - Participants randomised: n=96 (48 males and 48 females) participants who had not engaged in regular exercise programs at the time of the study.  - Participants included in the analysis, n=96:   - Exercise (running) group: - n=12 males, mean age of 33.9 years (SD 7.6). - n=12 females, mean age of 37.2 years (SD 7.4). - Diet group: - n=12 males, mean age of 40.1 years (SD 6.5). - n=12 females, mean age of 41.3 years (SD 7.9). - Exercise (running) plus diet group: - n=12 males, mean age of 34.4 years (SD 5.6). - n=12 females, mean age of 34.2 years (SD 6.5). - Control group: - n=12 males, mean age of 34.2 years (SD 8.4). - n=12 females, mean age of 33.2 years (SD 9.8). |
| --- |

**Follow-up period and time-point assessments after baseline**

| - Follow-up: 12 weeks.  - Time-points:   - Baseline and 12 weeks after baseline: body composition, resting blood pressure, forced vital capacity and maximal aerobic power. - Baseline, 4, 8 and 12 weeks after baseline: blood measurements. |
| --- |
| p.s.: The results corresponding to follow-up after 4 weeks from baseline were not extracted because 8 weeks or more of running training is an inclusion criterion of the systematic review. |

**Intervention (running program)**

| Exercise (running) group  - Duration: 12 weeks.  - Frequency: 5 times/week.  - Description: walking and/or running for 30 min/session in a 1-mile outdoor track, and maintained their normal pattern of dietary consumption. |
| --- |

**Comparison group**

| Diet group  - Duration: 12 weeks.  - Description: participants consumed 1200 kcal/day of the Shaklee diet. Participants consumed one package drink mixed with water for breakfast (210 kcal) and a second drink at lunch (210 kcal). The remaining balance of calories was consumed at dinner from a mixed diet. Participants were also required to attend to a weekly 30-45 min meeting during which their progress was evaluated and discussed. | Exercise (running) plus diet group  - Duration: 12 weeks.  - Description: walking and/or running 5 days/week for 30 min/session in a 1-mile outdoor track and consumed 1200 kcal/day of the Shaklee diet (see details in the diet group). Participants were also required to attend to a weekly 30-45 min meeting during which their progress was evaluated and discussed. | Control group  - Participants maintained their normal pattern of dietary consumption and performed no regular exercise conditioning. |
| --- | --- | --- |

**Body composition outcomes and between groups comparison**

** statistically significant difference between running group and control group*

^†^ *statistically significant difference between running group and diet group*

^§^ *statistically significant difference between running group and running/diet group*

^‡^ *statistically significant difference within groups (pre-post)*

| Mean after 12 weeks for males | | | |
| --- | --- | --- | --- |
| Running group  - Body weight (kg): 95.5 (SD 9.6)^†§^  - Fat weight (kg): 23.4 (SD 6.0)^†§^  - Fat-free weight (kg): 72.1 (SD 7.0)^†§^ | Diet group  - Body weight (kg): 83.2 (SD 8.2)  - Fat weight (kg): 17.5 (SD 2.6)  - Fat-free weight (kg): 65.7 (SD 6.5) | Running/diet group  - Body weight (kg): 84.6 (SD 9.6)  - Fat weight (kg): 17.2 (SD 5.6)  - Fat-free weight (kg): 67.4 (SD 6.3) | Control group  - Body weight (kg): 91.3 (SD 11.5)  - Fat weight (kg): 22.1 (SD 4.8)  - Fat-free weight (kg): 69.2 (SD 9.4) |
| Mean after 12 weeks for females | | | |
| Running group  - Body weight (kg): 70.8 (SD 7.1)^†§^  - Fat weight (kg): 23.6 (SD 4.6)^†§^  - Fat-free weight (kg): 47.2 (SD 5.7)^†§^ | Diet group  - Body weight (kg): 64.6 (SD 7.0)  - Fat weight (kg): 19.3 (SD 4.5)  - Fat-free weight (kg): 45.3 (SD 3.4) | Running/diet group  - Body weight (kg): 64.4 (SD 5.4)  - Fat weight (kg): 18.8 (SD 3.7)  - Fat-free weight (kg): 45.6 (SD 4.0) | Control group  - Body weight (kg): 69.2 (SD 5.8)  - Fat weight (kg): 22.6 (SD 4.5)  - Fat-free weight (kg): 46.6 (SD 3.2) |
| Pre-post mean and percentage differences (dispersion or uncertainty measurements not reported) 12 weeks after baseline for males | | | |
| Running group  - ΔBody weight (kg): -0.3 (%Δ -0.3)^†§^  - ΔFat weight (kg): -0.2 (%Δ -0.8)^†§^  - ΔFat-free weight (kg): -0.1 (%Δ -0.1)^†§^ | Diet group  - ΔBody weight (kg): -8.4 (%Δ -9.1)  - ΔFat weight (kg): -5.9 (%Δ -25.2)  - ΔFat-free weight (kg): -2.5 (%Δ -4.0) | Running/diet group  - ΔBody weight (kg): -11.4 (%Δ -11.8)  - ΔFat weight (kg): -7.9 (%Δ -31.5)  - ΔFat-free weight (kg): -3.5 (%Δ -5.0) | Control group  - ΔBody weight (kg): 0.9 (%Δ 1.0)  - ΔFat weight (kg): 0.8 (%Δ 3.8)  - ΔFat-free weight (kg): 0.1 (%Δ 0.1) |
| Pre-post mean and percentage differences (dispersion or uncertainty measurements not reported) 12 weeks after baseline for females | | | |
| Running group  - ΔBody weight (kg): -0.6 (%Δ -0.9)^†§^  - ΔFat weight (kg): -1.2 (%Δ -4.8)^†§^  - ΔFat-free weight (kg): 0.6 (%Δ 1.3)^†§^ | Diet group  - ΔBody weight (kg): -5.5 (%Δ -7.8)  - ΔFat weight (kg): -4.9 (%Δ -20.2)  - ΔFat-free weight (kg): -0.6 (%Δ -1.3) | Running/diet group  - ΔBody weight (kg): -7.5 (%Δ -10.4)  - ΔFat weight (kg): -5.9 (%Δ -23.9)  - ΔFat-free weight (kg): -1.6 (%Δ -3.4) | Control group  - ΔBody weight (kg): 0.8 (%Δ 1.2)  - ΔFat weight (kg): 0.2 (%Δ 0.9)  - ΔFat-free weight (kg): 0.7 (%Δ 1.5) |

**Cardiorespiratory outcomes and between groups comparison**

** statistically significant difference between running group and control group*

^†^ *statistically significant difference between running group and diet group*

^§^ *statistically significant difference between running group and running/diet group*

^‡^ *statistically significant difference within groups (pre-post)*

*FFW: fat-free weight*

| Mean after 12 weeks for males | | | |
| --- | --- | --- | --- |
| Running group  - VO_2_max (l/min): 3.87 (SD 0.51)*^†^  - VO_2_max (ml/kg/min): 40.7 (SD 5.7)*^§^  - VO_2_max (ml/kgFFW/min): 53.6 (SD 5.2)*^†^ | Diet group  - VO_2_max (l/min): 3.35 (SD 0.41)  - VO_2_max (ml/kg/min): 40.4 (SD 5.2)  - VO_2_max (ml/kgFFW/min): 51.2 (SD 6.6) | Running/diet group  - VO_2_max (l/min): 3.83 (SD 0.52)  - VO_2_max (ml/kg/min): 45.4 (SD 5.0)  - VO_2_max (ml/kgFFW/min): 56.7 (SD 4.7) | Control group  - VO_2_max (l/min): 3.55 (SD 0.43)  - VO_2_max (ml/kg/min): 39.2 (SD 4.6)  - VO_2_max (ml/kgFFW/min): 51.7 (SD 6.1) |
| Mean after 12 weeks for females | | | |
| Running group  - VO_2_max (l/min): 2.20 (SD 0.41)*^†^  - VO_2_max (ml/kg/min): 31.0 (SD 3.9)*^§^  - VO_2_max (ml/kgFFW/min): 46.5 (SD 5.0)*^†^ | Diet group  - VO_2_max (l/min): 1.99 (SD 0.35)  - VO_2_max (ml/kg/min): 30.8 (SD 4.6)  - VO_2_max (ml/kgFFW/min): 43.7 (SD 5.5) | Running/diet group  - VO_2_max (l/min): 2.38 (SD 0.38)  - VO_2_max (ml/kg/min): 36.9 (SD 5.6)  - VO_2_max (ml/kgFFW/min): 52.0 (SD 7.0) | Control group  - VO_2_max (l/min): 1.96 (SD 0.32)  - VO_2_max (ml/kg/min): 28.3 (SD 4.1)  - VO_2_max (ml/kgFFW/min): 42.0 (SD 5.6) |
| Pre-post mean and percentage differences (dispersion or uncertainty measurements not reported) 12 weeks after baseline for males | | | |
| Running group  - ΔVO_2_max (l/min): 0.47 (%Δ 14)*^†^  - ΔVO_2_max (ml/kg/min): 5.1 (%Δ 14)*^§^  - ΔVO_2_max (ml/kgFFW/min): 6.7 (%Δ 14)*^†^ | Diet group  - ΔVO_2_max (l/min): 0.00 (%Δ 0)  - ΔVO_2_max (ml/kg/min): 3.7 (%Δ 10)  - ΔVO_2_max (ml/kgFFW/min): 1.9 (%Δ 4) | Running/diet group  - ΔVO_2_max (l/min): 0.36 (%Δ 10)  - ΔVO_2_max (ml/kg/min): 9.3 (%Δ 26)  - ΔVO_2_max (ml/kgFFW/min): 8.0 (%Δ 16) | Control group  - ΔVO_2_max (l/min): 0.10 (%Δ 3)  - ΔVO_2_max (ml/kg/min): 1.0 (%Δ 3)  - ΔVO_2_max (ml/kgFFW/min): 1.7 (%Δ 3) |
| Pre-post mean and percentage differences (dispersion or uncertainty measurements not reported) 12 weeks after baseline for females | | | |
| Running group  - ΔVO_2_max (l/min): 0.29 (%Δ 15)*^†^  - ΔVO_2_max (ml/kg/min): 4.5 (%Δ 17)*^§^  - ΔVO_2_max (ml/kgFFW/min): 5.8 (%Δ 14)*^†^ | Diet group  - ΔVO_2_max (l/min): 0.07 (%Δ 4)  - ΔVO_2_max (ml/kg/min): 3.3 (%Δ 12)  - ΔVO_2_max (ml/kgFFW/min): 1.8 (%Δ 4) | Running/diet group  - ΔVO_2_max (l/min): 0.27 (%Δ 13)  - ΔVO_2_max (ml/kg/min): 7.5 (%Δ 25)  - ΔVO_2_max (ml/kgFFW/min): 7.3 (%Δ 16) | Control group  - ΔVO_2_max (l/min): -0.02 (%Δ -1)  - ΔVO_2_max (ml/kg/min): -0.9 (%Δ -3)  - ΔVO_2_max (ml/kgFFW/min): -1.1 (%Δ -3) |
| p.s.: The results for blood pressure (systolic and diastolic) and heart rate (resting and maximal during exercise) were not reported for each group, however there were not statistically significant difference between or within groups. | | | |

**Blood serum concentration outcomes and between groups comparison**

** statistically significant difference between running group and control group*

^†^ *statistically significant difference between running group and diet group*

^§^ *statistically significant difference between running group and running/diet group*

^‡^ *statistically significant difference within groups (pre-post)*

| Mean after 8 weeks for males | | | |
| --- | --- | --- | --- |
| Running group  - Triglycerides (mg/dl): 200 (SD 114)^§^  - Cholesterol (mg/dl): 186 (SD 33)^§^  - LDL (mg/dl): 116 (SD 30)  - VLDL (mg/dl): 40 (SD 23)^§^  - HDL (mg/dl): 30 (SD 6)  - Cholesterol/HDL: 6.4 (SD 1.9)^§^ | Diet group  - Triglycerides (mg/dl): 101 (SD 38)  - Cholesterol (mg/dl): 176 (SD 27)  - LDL (mg/dl): 116 (SD 23)  - VLDL (mg/dl): 21 (SD 8)  - HDL (mg/dl): 39 (SD 9)  - Cholesterol/HDL: 4.6 (SD 1.0) | Running/diet group  - Triglycerides (mg/dl): 89 (SD 31)  - Cholesterol (mg/dl): 155 (SD 21)  - LDL (mg/dl): 103 (SD 18)  - VLDL (mg/dl): 17 (SD 6)  - HDL (mg/dl): 35 (SD 4)  - Cholesterol/HDL: 4.5 (SD 0.9) | Control group  - Triglycerides (mg/dl): 185 (SD 149)  - Cholesterol (mg/dl): 192 (SD 45)  - LDL (mg/dl): 122 (SD 42)  - VLDL (mg/dl): 36 (SD 30)  - HDL (mg/dl): 34 (SD 7)  - Cholesterol/HDL: 6.0 (SD 2.0) |
| Mean after 8 weeks for females | | | |
| Running group  - Triglycerides (mg/dl): 132 (SD 113)  - Cholesterol (mg/dl): 174 (SD 28)  - LDL (mg/dl): 103 (SD 27)  - VLDL (mg/dl): 23 (SD 19)  - HDL (mg/dl): 48 (SD 11)  - Cholesterol/HDL: 3.9 (SD 1.5) | Diet group  - Triglycerides (mg/dl): 73 (SD 15)  - Cholesterol (mg/dl): 172 (SD 34)  - LDL (mg/dl): 113 (SD 25)  - VLDL (mg/dl): 15 (SD 3)  - HDL (mg/dl): 44 (SD 9)  - Cholesterol/HDL: 3.9 (SD 0.5) | Running/diet group  - Triglycerides (mg/dl): 89 (SD 42)  - Cholesterol (mg/dl): 189 (SD 40)  - LDL (mg/dl): 126 (SD 32)  - VLDL (mg/dl): 19 (SD 8)  - HDL (mg/dl): 44 (SD 8)  - Cholesterol/HDL: 4.3 (SD 0.9) | Control group  - Triglycerides (mg/dl): 109 (SD 49)  - Cholesterol (mg/dl): 196 (SD 27)  - LDL (mg/dl): 128 (SD 34)  - VLDL (mg/dl): 21 (SD 10)  - HDL (mg/dl): 47 (SD 12)  - Cholesterol/HDL: 4.6 (SD 1.7) |
| Mean after 12 weeks for males | | | |
| Running group  - Triglycerides (mg/dl): 242 (SD 160)^§^  - Cholesterol (mg/dl): 211 (SD 29)^§^  - LDL (mg/dl): 130 (SD 35)  - VLDL (mg/dl): 48 (SD 32)^§^  - HDL (mg/dl): 33 (SD 8)  - Cholesterol/HDL: 6.8 (SD 1.9)^§^ | Diet group  - Triglycerides (mg/dl): 98 (SD 28)  - Cholesterol (mg/dl): 191 (SD 29)  - LDL (mg/dl): 132 (SD 27)  - VLDL (mg/dl): 20 (SD 5)  - HDL (mg/dl): 39 (SD 10)  - Cholesterol/HDL: 5.2 (SD 1.2) | Running/diet group  - Triglycerides (mg/dl): 83 (SD 32)  - Cholesterol (mg/dl): 165 (SD 29)  - LDL (mg/dl): 111 (SD 23)  - VLDL (mg/dl): 16 (SD 6)  - HDL (mg/dl): 38 (SD 7)  - Cholesterol/HDL: 4.5 (SD 0.9) | Control group  - Triglycerides (mg/dl): 152 (SD 83)  - Cholesterol (mg/dl): 198 (SD 41)  - LDL (mg/dl): 133 (SD 37)  - VLDL (mg/dl): 30 (SD 17)  - HDL (mg/dl): 35 (SD 6)  - Cholesterol/HDL: 5.8 (SD 1.5) |
| Mean after 12 weeks for females | | | |
| Running group  - Triglycerides (mg/dl): 87 (SD 54)  - Cholesterol (mg/dl): 185 (SD 38)  - LDL (mg/dl): 118 (SD 33)  - VLDL (mg/dl): 18 (SD 11)  - HDL (mg/dl): 49 (SD 11)  - Cholesterol/HDL: 4.0 (SD 1.3) | Diet group  - Triglycerides (mg/dl): 77 (SD 24)  - Cholesterol (mg/dl): 186 (SD 34)  - LDL (mg/dl): 122 (SD 28)  - VLDL (mg/dl): 15 (SD 5)  - HDL (mg/dl): 49 (SD 8)  - Cholesterol/HDL: 3.8 (SD 0.6) | Running/diet group  - Triglycerides (mg/dl): 86 (SD 41)  - Cholesterol (mg/dl): 197 (SD 49)  - LDL (mg/dl): 135 (SD 39)  - VLDL (mg/dl): 18 (SD 8)  - HDL (mg/dl): 44 (SD 11)  - Cholesterol/HDL: 4.6 (SD 1.3) | Control group  - Triglycerides (mg/dl): 104 (SD 47)  - Cholesterol (mg/dl): 198 (SD 31)  - LDL (mg/dl): 129 (SD 33)  - VLDL (mg/dl): 22 (SD 9)  - HDL (mg/dl): 47 (SD 14)  - Cholesterol/HDL: 4.5 (SD 1.6) |
| p.s.: The results for glucose, sodium, potassium, chloride, total protein, albumin, globulin, bilirubin, creatinine, uric acid and blood urea nitrogen were not reported for each group, however there were not statistically significant difference between or within groups. | | | |

**Reference**

| Mueller JK, Gossard D, Adams FR, Taylor CB, Haskell WL, Kraemer HC, et al. Assessment of prescribed increases in physical activity: application of a new method for microprocessor analysis of heart rate. The American journal of cardiology. 1986;57(6):441-5. |
| --- |

**Objectives**

| 1- To identify the best measures for characterizing daily physical activity as reflected in heart rate;  2- To investigate if these measures are sufficiently reproducible and sensitive to detect a prescribed increase in physical activity? |
| --- |

**Description of participants included in the study**

| - Participants randomised: n=64 sedentary middle-aged men (mean age of 49 years [SD 6]).  - Participants included in the analysis, n=64:   - High-intensity exercise training group: n=23. - Low-intensity exercise training group: n=21. - Control group: n=20. |
| --- |

**Follow-up period and time-point assessments after baseline**

| - Follow-up: 3 months.  - Time-points: baseline, 6 and 12 weeks after baseline. |
| --- |
| p.s.: The results corresponding to follow-up after 6 weeks from baseline were not extracted because 8 weeks or more of running training is an inclusion criterion of the systematic review. |

**Intervention (running program)**

| High-intensity exercise training group  - Duration: 12 weeks.  - Frequency: 5 times/week.  - Description: walking or jogging at home without supervision. Participants trained within a 20-beat range above 75-87% of peak heart rate. The duration of each training session was individually prescribed to elicit an energy expenditure of approximately 300 kcal. The average length of training sessions was 37 min (7; dispersion or uncertainty type of measure not described). | Low-intensity exercise training group  - Duration: 12 weeks.  - Frequency: 5 times/week.  - Description: walking or jogging at home without supervision. Participants trained within a 20-beat range above 60-72% of peak heart rate. The duration of each training session was individually prescribed to elicit an energy expenditure of approximately 300 kcal. The average length of training sessions was 52 min (5; dispersion or uncertainty type of measure not described). |
| --- | --- |

**Comparison group**

| Control group  - Participants were instructed to carry out their customary physical activity during the study. |
| --- |

**Body composition outcomes and between groups comparison**

** statistically significant difference between high-intensity group and control group*

^†^ *statistically significant difference between low-intensity group and control group*

^§^ *statistically significant difference between high-intensity group and low-intensity group*

^‡^ *statistically significant difference within groups (pre-post)*

| Not applicable | Not applicable | Not applicable |
| --- | --- | --- |

**Cardiorespiratory outcomes and between groups comparison**

** statistically significant difference between high-intensity group and control group*

^†^ *statistically significant difference between low-intensity group and control group*

^§^ *statistically significant difference between high-intensity group and low-intensity group*

^‡^ *statistically significant difference within groups (pre-post)*

| Mean after 12 weeks | | |
| --- | --- | --- |
| High-intensity group  - Heart rate (bpm): result reported in a graph  - VO_2_max (ml/kg/min): 37.6 (4.8)^§‡^ | Low-intensity group  - Heart rate (bpm): result reported in a graph  - VO_2_max (ml/kg/min): 36.0 (4.6)^‡^ | Control group  - Heart rate (bpm): result reported in a graph  - VO_2_max (ml/kg/min): 31.1 (4.5) |
| Pre-post mean difference 12 weeks after baseline | | |
| High-intensity group  - ΔHeart rate (%): 3.8% (dispersion or uncertainty not reported) | Low-intensity group  - ΔHeart rate (%): 4.7% (dispersion or uncertainty not reported) | Control group  - ΔHeart rate (%): not reported |
| p.s.: Dispersion or uncertainty measure unit/qualifier was not reported. | | |

**Blood serum concentration outcomes and between groups comparison**

** statistically significant difference between high-intensity group and control group*

^†^ *statistically significant difference between low-intensity group and control group*

^§^ *statistically significant difference between high-intensity group and low-intensity group*

^‡^ *statistically significant difference within groups (pre-post)*

| Not applicable | Not applicable | Not applicable |
| --- | --- | --- |

**Reference**

| Savage MP, Petratis MM, Thomson WH, Berg K, Smith JL, Sady SP. Exercise training effects on serum lipids of prepubescent boys and adult men. Medicine and science in sports and exercise. 1986;18(2):197-204. |
| --- |

**Objectives**

| To compare the effects of 10 weeks of low and high intensity exercise training on the serum lipids and lipoproteins of prepubescent boys and adult men. |
| --- |

**Description of participants included in the study**

| - Participants randomised: n=34 men who did not participate in regular aerobic exercise program (3 or more times/week) within the last 3 months.  - Source: Omaha, NE metropolitan area.  - Participants included in the analysis, n=30:   - High intensity group: n=12, mean age of 36.6 years (SD 4.09). - Low intensity group: n=8, mean age of 36.6 years (SD 3.18). - Control group: n=10, mean of age of 36.7 years (SD 4.82). |
| --- |

**Follow-up period and time-point assessments after baseline**

| - Follow-up: 11 weeks.  - Time-points: baseline and 11 weeks after baseline. |
| --- |

**Intervention (running program)**

| High intensity group  - Duration: 10 weeks.  - Frequency: 3 times/week.  - Description: after 1 week of accommodation, participants performed walking/jogging/running with warm-up and stretching for 10 weeks. Training began with 2.4 km/session and was gradually increased to 4.8 km per session. Training intensity heart rate was 75% (76.6%, SE 4.83) of VO_2_max. | Low intensity group  - Duration: 10 weeks.  - Frequency: 3 times/week.  - Description: after 1 week of accommodation, participants performed walking/jogging/running with warm-up and stretching for 10 weeks. Training began with 2.4 km/session and was gradually increased to 4.8 km per session. Training intensity heart rate was 40% (41.3%, SE 1.82) of VO_2_max. |
| --- | --- |

**Comparison group**

| Control group  - Participants were requested to maintain their current activity pattern throughout the study. |
| --- |

**Body composition outcomes and between groups comparison**

** statistically significant difference between high intensity group and control group*

^†^ *statistically significant difference between low intensity group and control group*

*^‡^ statistically significant difference within groups (pre-post)*

| Mean 11 weeks after baseline | | |
| --- | --- | --- |
| High intensity group  - Body weight (kg): 79.6 (SE 2.68)  - Body density (g/ml): 1.051 (SE 0.003)  - Sum of 6 skinfolds (mm): 97.0 (SE 6.63)* | Low intensity group  - Body weight (kg): 96.2 (SE 6.66)  - Body density (g/ml): 1.040 (SE 0.006)  - Sum of 6 skinfolds (mm): 115.8 (SE 14.70)^†^ | Control intensity group  - Body weight (kg): 79.4 (SE 2.75)  - Body density (g/ml): 1.047 (SE 0.004)  - Sum of 6 skinfolds (mm): 106.6 (SE 7.99) |

**Cardiorespiratory outcomes and between groups comparison**

** statistically significant difference between high intensity group and control group*

^†^ *statistically significant difference between low intensity group and control group*

*^‡^ statistically significant difference within groups (pre-post)*

| Mean 11 weeks after baseline | | |
| --- | --- | --- |
| High intensity group  - VO_2_max (ml/kg/min): 46.6 (SE 1.37)*  - Respiratory exchange ratio max: 1.30 (SE 0.016) | Low intensity group  - VO_2_max (ml/kg/min): 38.4 (SE 1.24)  - Respiratory exchange ratio max: 1.34 (SE 0.026) | Control intensity group  - VO_2_max (ml/kg/min): 44.1 (SE 2.30)  - Respiratory exchange ratio max: 1.27 (SE 0.014) |

**Blood serum concentration outcomes and between groups comparison**

** statistically significant difference between high intensity group and control group*

^†^ *statistically significant difference between low intensity group and control group*

*^‡^ statistically significant difference within groups (pre-post)*

| Mean 11 weeks after baseline | | |
| --- | --- | --- |
| High intensity group  - HDL/cholesterol (%): 23 (SE 1.8)  - Triglycerides (mg/dl): 99 (SE 11.6)  - LDL (mg/dl): 133 (SE 15.4)  - HDL (mg/dl): 42 (SE 4.5)*  - Cholesterol (mg/dl): results reported in a graph | Low intensity group  - HDL/cholesterol (%): 26 (SE 1.8)  - Triglycerides (mg/dl): 118 (SE 20.7)  - LDL (mg/dl): 116 (SE 7.9)  - HDL (mg/dl): 47 (SE 5.3)  - Cholesterol (mg/dl): results reported in a graph | Control intensity group  - HDL/cholesterol (%): 26 (SE 2.8)  - Triglycerides (mg/dl): 138 (SE 21.8)  - LDL (mg/dl): 127 (SE 11.4)  - HDL (mg/dl): 53 (SE 4.9)  - Cholesterol (mg/dl): results reported in a graph |

**Reference**

| Thomas TR, Adeniran SB, Iltis PW, Aquiar CA, Albers JJ. Effects of interval and continuous running on HDL-cholesterol, apoproteins A-1 and B, and LCAT. Canadian journal of applied sport sciences Journal canadien des sciences appliquees au sport. 1985;10(1):52-9. |
| --- |

**Objectives**

| To examine the effects of continuous and interval exercise training on plasma HDL-C, apoproteins A-I and B, and lecithin:cholesterol acyltransferase (LCAT). |
| --- |

**Description of participants included in the study**

| - Participants randomised: n=48 male college students who did not participate in a systematic aerobic exercise program for 6 months prior to the study.  - Participants included in the analysis: n=36:   - 5-mile continuous group: n=11, mean age of 23.0 years (SD 1.2) - 4-min interval group, n=8, mean age of 23.1 years (SD 1.9) - 2-min interval group, n=9, mean age of 22.8 years (SD 1.1) - Control group, n=8, mean age of 21.9 years (SD 1.0) |
| --- |

**Follow-up period and time-point assessments after baseline**

| - Follow-up: 11 weeks.  - Time-points:   - baseline and 11 weeks after baseline for lipoproteins variables; - baseline, midpoint and 11 weeks after baseline for LCAT. |
| --- |
| p.s.: The results corresponding to midpoint from baseline were not extracted because 8 weeks or more of running training is an inclusion criterion of the systematic review. |

**Intervention (running program)**

| 5-mile group  - Duration: 11 weeks.  - Frequency: 3 times/week.  - Description: the participants progressed to running the mileage continuously at an 8:00/mile pace for 1 hour. They ran at intensities between 75-85% maximal heart rate. |
| --- |

**Comparison group**

| 4-min group  - Duration: 11 weeks.  - Frequency: 3 times/week.  - Description: the participants ran work bouts of 4 min followed by an equal recovery time (1:1 work:rest) for 1 hour. They progressed to running 6 work:rest bouts each exercise session. They ran at intensities between 90-100% maximal heart rate. | 2-min group  - Duration: 11 weeks.  - Frequency: 3 times/week.  - Description: the participants ran work bouts of 2 min followed by 3 min of recovery (1:1-1/2 work:rest) for 1 hour. They progressed to running 8 work:rest bouts each exercise session. They ran at intensities between 90-100% maximal heart rate. | Control group  - No description. |
| --- | --- | --- |

**Body composition outcomes and between groups comparison**

** statistically significant difference between 5-mile group and control group*

*^‡^ statistically significant difference within groups (pre-post)*

| Mean 11 weeks after baseline | | | |
| --- | --- | --- | --- |
| 5-mile group  - Body weight (kg): 73.05 (SD 8.45) | 4-min group  - Body weight (kg): 74.05 (SD 15.35) | 2-min group  - Body weight (kg): 81.10 (SD 11.35) | Control group  - Body weight (kg): 69.75 (SD 6.35) |

**Cardiorespiratory outcomes and between groups comparison**

** statistically significant difference between 5-mile group and control group*

*^‡^ statistically significant difference within groups (pre-post)*

| Mean 11 weeks after baseline | | | |
| --- | --- | --- | --- |
| 5-mile group  - VO_2_max (l/min): 4.04 (SD 0.44)  - VO_2_max (ml/kg/min): 55.9 (SD 4.8)*^‡^ | 4-min group  - VO_2_max (l/min): 3.91 (SD 0.36)^‡^  - VO_2_max (ml/kg/min): 53.7 (SD 6.8)^†‡^ | 2-min group  - VO_2_max (l/min): 4.04 (SD 0.35)  - VO_2_max (ml/kg/min): 50.3 (SD 5.3) | Control group  - VO_2_max (l/min): 3.34 (SD 0.44)  - VO_2_max (ml/kg/min): 47.8 (SD 2.8) |

**Blood serum concentration outcomes and between groups comparison**

** statistically significant difference between 5-mile group and control group*

*^‡^ statistically significant difference within groups (pre-post)*

| Mean 11 weeks after baseline | | | |
| --- | --- | --- | --- |
| 5-mile group  - Total cholesterol (mg%): 175 (SD 37)  - HDL (mg%): 49 (SD 9)  - Apoprotein A-I (mg%): reported in a graph  - Apoprotein B (mg%): reported in a graph  - LCAT (%^3^H cholesterol acylated/hr): 6.1 (SD not reported) | 4-min group  - Total cholesterol (mg%): 158 (SD 17)  - HDL (mg%): 49 (SD 11)  - Apoprotein A-I (mg%): reported in a graph  - Apoprotein B (mg%): reported in a graph  - LCAT (%^3^H cholesterol acylated/hr): 6.1 (SD not reported) | 2-min group  - Total cholesterol (mg%): 158 (SD 17)  - HDL (mg%): 40 (SD 5)  - Apoprotein A-I (mg%): reported in a graph  - Apoprotein B (mg%): reported in a graph  - LCAT (%^3^H cholesterol acylated/hr): 7.2 (SD not reported) | Control group  - Total cholesterol (mg%): 143 (SD 23)  - HDL (mg%): 42 (SD 9)  - Apoprotein A-I (mg%): reported in a graph  - Apoprotein B (mg%): reported in a graph  - LCAT (%^3^H cholesterol acylated/hr): 7.4 (SD not reported) |

**Reference**

| Iltis PW, Thomas TR, Adeniran SB, Aguiar CA, Albers JJ. Different running programs: plasma lipids, apoproteins, and lecithin: cholesterol acyltransferase in middle-aged men. Annals of Sports Medicine. 1984;2(1):16-22. |
| --- |

**Objectives**

| To investigate the effects of different running programs on total cholesterol, HDL, total cholesterol/HDL ratios, apolipoprotein A-I, apolipopretein B, lecithin:cholesterol acyltransferase activity in previous untrained, middle-aged men. |
| --- |

**Description of participants included in the study**

| - Participants randomised: n=48 men without any formal training in the previous 6 months.  - Participants included in the analysis, n=40:   - 4-mile group: n=11, mean age of 38.1 years (SD 4.3). - 2-mile group: n=12, mean age of 41.8 years (SD 4.7). - Interval group: n=7, mean age of 38.9 years (SD 5.4). - Control group: n=10, mean age of 37.8 years (SD 5.2). |
| --- |

**Follow-up period and time-point assessments after baseline**

| - Follow-up: 13 weeks.  - Time-points: baseline, middle of the training and after 13 weeks after baseline. |
| --- |
| p.s.: The results corresponding to midpoint from baseline were not extracted because 8 weeks or more of running training is an inclusion criterion of the systematic review. |

**Intervention (running program)**

| 4-mile group  - Duration: 13 weeks.  - Frequency: 3 times/week.  - Description: training progression (1 h/day) began with running 1.5 miles per session for the first 2 weeks, followed by weekly increments of 0.5 miles until training distance achieved. Intensity was prescribed at 80% of age predicted maximal heart rate. Subjects achieved 12 miles/week by the 7^th^ week. | 2-mile group  - Duration: 13 weeks.  - Frequency: 3 times/week.  - Description: training progression (1 h/day) began with running 1.5 miles per session for the first 2 weeks, followed by weekly increments of 0.5 miles until training distance achieved. Intensity was prescribed at 80% of age predicted maximal heart rate. Subjects achieved 6 miles/week by the 3^rd^ week. |
| --- | --- |

**Comparison group**

| Interval group  - Duration: 13 weeks.  - Frequency: 3 times/week.  - Description: 2-minute intervals at a run/walk ratio of one to one (1 h/day). Intensity was prescribed at 85% (weeks 1-3), 90% (weeks 3-7) and 95% (weeks 7-13) of maximal oxygen consumption. The number of intervals/week was adjusted to match the total caloric expenditure of each 4-mile group stage progression. | Control group  - Participants were instructed to not alter their current activity levels and not change their current diets. |
| --- | --- |

**Body composition outcomes and between groups comparison**

** statistically significant difference between groups*

*^‡^ statistically significant difference within groups (pre-post)*

| Mean 13 weeks after baseline | | | |
| --- | --- | --- | --- |
| 4-mile group  - Body weight (kg): 79.4 (SD 12.12) | 2-mile group  - Body weight (kg): 81.27 (SD 10.66) | Interval group  - Body weight (kg): 82.11 (SD 13.29) | Control group  - Body weight (kg): 82.77 (SD 9.13) |

**Cardiorespiratory outcomes and between groups comparison**

** statistically significant difference between groups*

*^‡^ statistically significant difference within groups (pre-post)*

| Mean 13 weeks after baseline | | | |
| --- | --- | --- | --- |
| 4-mile group  - TT90% (%): 19.2 | 2-mile group  - TT90% (%): 25.1 | Interval group  - TT90% (%): 26.7 | Control group  - TT90% (%): not reported |
| p.s.: Dispersion and/or uncertainty measures were not described. | | | |

**Blood serum concentration outcomes and between groups comparison**

** statistically significant difference between groups*

*^‡^ statistically significant difference within groups (pre-post)*

*LCAT: lecithin:cholesterol acyltransferase*

| Mean 13 weeks after baseline | | | |
| --- | --- | --- | --- |
| 4-mile group  - Total cholesterol (mg%): 206.2 (SD 38.5)  - HDL (mg%): 42.8 (SD 9.8)  - TC/HDL (mg%): 4.97 (SD 1.1)  - Apolopoprotein AI (mg%): reported in a graph  - Apolipoprotein B (mg%): reported in a graph  - LCAT (%^3^H): reported in a graph | 2-mile group  - Total cholesterol (mg%): 214.6 (SD 48.6)  - HDL (mg%): 45.9 (SD 12.0)  - TC/HDL (mg%): 5.02 (SD 1.9)  - Apolopoprotein AI (mg%): reported in a graph  - Apolipoprotein B (mg%): reported in a graph  - LCAT (%^3^H): reported in a graph | Interval group  - Total cholesterol (mg%): 194.1 (SD 25.8)  - HDL (mg%): 39.9 (SD 7.2)  - TC/HDL (mg%): 4.99 (SD 1.1)  - Apolopoprotein AI (mg%): reported in a graph  - Apolipoprotein B (mg%): reported in a graph  - LCAT (%^3^H): reported in a graph | Control group  - Total cholesterol (mg%): 218.0 (SD 40.5)  - HDL (mg%): 46.5 (SD 15.4)  - TC/HDL (mg%): 5.16 (SD 1.8)  - Apolopoprotein AI (mg%): reported in a graph  - Apolipoprotein B (mg%): reported in a graph  - LCAT (%^3^H): reported in a graph |

**More publications related to this study**

| Thomas TR, Adeniran SB, Etheridge GL. Effects of different running programs on VO2 max, percent fat, and plasma lipids. Canadian journal of applied sport sciences Journal canadien des sciences appliquees au sport. 1984;9(2):55-62. |
| --- |

**Reference**

| Mathur DN, Toriola AL. Twelve weeks jogging effects on selected cardiovascular risk factors in untrained healthy males. The Journal of sports medicine and physical fitness. 1984;24(3):259-62. |
| --- |

**Objectives**

| 1- To determine the pre-training levels of selected CHD risk factors (blood pressure, blood glucose and serum uric acid) in young male undergraduates;  2- To investigate the effectiveness of different levels of jogging programs on these CHD risk factors. |
| --- |

**Description of participants included in the study**

| - Participants randomised: n= 40 untrained male undergraduate students.  - Source: University of Ife, Nigeria.  - Participants included in the analysis, n=40:   - 4.8 km running group: n=10, mean age of 25.6 years (SD 2.6). - 3.2 km running group: n=10, mean age of 23.3 years (SD 3.0). - 1.6 km running group: n=10, mean age of 23.5 years (SD 2.8). - Control group: n=10, mean age of 24.1 years (SD 3.4). |
| --- |

**Follow-up period and time-point assessments after baseline**

| - Follow- up: 12 weeks.  - Time-points: baseline and 12 weeks after baseline. |
| --- |

**Intervention (running program)**

| 4.8 km running group  - Duration: 12 weeks.  - Frequency: 3 times/week.  - Description: jogging 4.8 km with a intensity about 85% of maximal heart rate. Each jogging session consisted in 5 min warm-up involving calisthenics, jogging 18-25 min and 5 min of cool-down activities. Participants ran in a 400 m track. | 3.2 km running group  - Duration: 12 weeks.  - Frequency: 3 times/week.  - Description: jogging 3.2 km with a intensity about 85% of maximal heart rate. Each jogging session consisted in 5 min warm-up involving calisthenics, jogging 12-18 min and 5 min of cool-down activities. Participants ran in a 400 m track. | 1.6 km running group  - Duration: 12 weeks.  - Frequency: 3 times/week.  - Description: jogging 1.6 km with a intensity about 85% of maximal heart rate. Each jogging session consisted in 5 min warm-up involving calisthenics, jogging 5-10 min and 5 min of cool-down activities. Participants ran in a 400 m track. |
| --- | --- | --- |

**Comparison group**

| Control group  - Participants were not involved in any kind of vigorous physical activity during the study. |
| --- |

**Body composition outcomes and between groups comparison**

** statistically significant difference between 4.8 km group and control group*

^†^ *statistically significant difference between 3.2 km group and control group*

^§^ *statistically significant difference between 1.6 km group and control group*

^‡^ *statistically significant difference within groups (pre-post)*

| Not applicable | Not applicable | Not applicable | Not applicable |
| --- | --- | --- | --- |

**Cardiorespiratory outcomes and between groups comparison**

** statistically significant difference between 4.8 km group and control group*

^†^ *statistically significant difference between 3.2 km group and control group*

^§^ *statistically significant difference between 1.6 km group and control group*

^‡^ *statistically significant difference within groups (pre-post)*

| Mean 12 weeks after baseline | | | |
| --- | --- | --- | --- |
| 4.8 km group  - Systolic blood pressure (mmHg): 117.6 (SD 5.9)  - Diastolic blood pressure (mmHg): 74.8 (SD 2.3) | 3.2 km group  - Systolic blood pressure (mmHg): 119.6 (SD 5.1)  - Diastolic blood pressure (mmHg): 78.4 (SD 3.6) | 1.6 km group  - Systolic blood pressure (mmHg): 121.2 (SD 5.8)  - Diastolic blood pressure (mmHg): 76.2 (SD 2.0) | Control group  - Systolic blood pressure (mmHg): 125.0 (SD 8.8)  - Diastolic blood pressure (mmHg): 77.8 (SD 2.7) |
| Pre-post mean difference 12 weeks after baseline | | | |
| 4.8 km group  - ΔSystolic blood pressure (mmHg): -9.2 (SD 8.7)  - ΔDiastolic blood pressure (mmHg): -2.4 (SD 5.9) | 3.2 km group  - ΔSystolic blood pressure (mmHg): -8.0 (SD 7.8)  - ΔDiastolic blood pressure (mmHg): -2.4 (SD 6.0) | 1.6 km group  - ΔSystolic blood pressure (mmHg): -4.8 (SD 6.5)  - ΔDiastolic blood pressure (mmHg): -0.8 (SD 3.9) | Control group  - ΔSystolic blood pressure (mmHg): 2.6 (SD 6.3)  - ΔDiastolic blood pressure (mmHg): 2.8 (SD 5.4) |

**Blood serum concentration outcomes and between groups comparison**

** statistically significant difference between 4.8 km group and control group*

^†^ *statistically significant difference between 3.2 km group and control group*

^§^ *statistically significant difference between 1.6 km group and control group*

^‡^ *statistically significant difference within groups (pre-post)*

| Mean 12 weeks after baseline | | | |
| --- | --- | --- | --- |
| 4.8 km group  - Blood glucose (mg%): 73.6 (SD 6.1)  - Serum uric acid (mg%): 5.0 (SD 0.8) | 3.2 km group  - Blood glucose (mg%): 76.5 (SD 5.5)  - Serum uric acid (mg%): 5.2 (SD 0.8) | 1.6 km group  - Blood glucose (mg%): 81.7 (SD 3.4)  - Serum uric acid (mg%): 5.3 (SD 0.9) | Control group  - Blood glucose (mg%): 82.8 (SD 4.7)  - Serum uric acid (mg%): 5.4 (SD 0.8) |
| Pre-post mean difference 12 weeks after baseline | | | |
| 4.8 km group  - ΔBlood glucose (mg%): -9.6 (SD 6.1)*  - ΔSerum uric acid (mg%): -0.7 (SD 0.9)^‡^ | 3.2 km group  - ΔBlood glucose (mg%): -4.7 (SD 3.2)  - ΔSerum uric acid (mg%): -0.6 (SD 1.1) | 1.6 km group  - ΔBlood glucose (mg%): -1.8 (SD 5.2)  - ΔSerum uric acid (mg%): -0.2 (SD 0.9) | Control group  - ΔBlood glucose (mg%): -1.1 (SD 4.0)  - ΔSerum uric acid (mg%): -0.1 (SD 0.7) |

**More publications related to this study**

| Toriola AL. Influence of 12-week jogging on body fat and serum lipids. British journal of sports medicine. 1984;18(1):13-7. |
| --- |

**Reference**

| Thomas TR, Adeniran SB, Etheridge GL. Effects of different running programs on VO2 max, percent fat, and plasma lipids. Canadian journal of applied sport sciences Journal canadien des sciences appliquees au sport. 1984;9(2):55-62. |
| --- |

**Objectives**

| To determine the type of running program which most beneficially affects parameters associated with cardiovascular health. |
| --- |

**Description of participants included in the study**

| - Participants randomised: n=80 students (18-32 years of age) who had not participated in a systematic exercise program for the past year.  - Participants included in the analysis, n=59:   - 4-mile group: n=14, 5 males and 9 females. - 2-mile group: n=18, 7 males and 11 females. - Interval group: n=15, 6 males and 9 females. - Control group: n=12, 6 males and 6 females. |
| --- |

**Follow-up period and time-point assessments after baseline**

| - Follow-up: 12 weeks  - Time-points: baseline and 12 weeks after baseline. |
| --- |

**Intervention (running program)**

| 4-mile group  - Duration: 12 weeks.  - Frequency: 3 times/week.  - Description: Progression to running 4 miles per session at 75% maximal heart rate or about 7:30-8:00/mile pace for men and 8:00-9:00/mile pace for women (about 500 Cal/session). | 2-mile group  - Duration: 12 weeks.  - Frequency: 3 times/week.  - Description: They ran their mileage at the same pace as the 4-mile group but expended only one-half the energy (about 250 Cal/session). |
| --- | --- |

**Comparison group**

| Interval group  - Duration: 12 weeks.  - Frequency: 3 times/week.  - Description: They ran at 90% of maximal heart rate for 1 min followed by 3 min of walking. Subjects started with 2 sets and added one set every other workout period until, after 4 weeks, each member was running 8 sets of the work:rest bouts (about 500 Cal/session). | Control group  - The participants were engaged in no exercise program and were asked to maintain their regular living habits. |
| --- | --- |

**Body composition outcomes and between groups comparison**

** statistically significant difference between groups*

*^‡^ statistically significant difference within groups (pre-post)*

| Mean 12 weeks after baseline | | | |
| --- | --- | --- | --- |
| 4-mile group  - Body fat (%): reported in a graph | 2-mile group  - Body fat (%): reported in a graph | Interval group  - Body fat (%): reported in a graph | Control group  - Body fat (%): reported in a graph |

**Cardiorespiratory outcomes and between groups comparison**

** statistically significant difference between groups*

*^‡^ statistically significant difference within groups (pre-post)*

| Mean 12 weeks after baseline | | | |
| --- | --- | --- | --- |
| 4-mile group  - VO_2_max (l/min): 3.2 (SD 1.0)  - VO_2_max (ml/kg/min): 49.0 (SD 8.4) | 2-mile group  - VO_2_max (l/min): 3.1 (SD 0.8)  - VO_2_max (ml/kg/min): 47.5 (SD 9.0) | Interval group  - VO_2_max (l/min): 3.3 (SD 0.8)  - VO_2_max (ml/kg/min): 49.5 (SD 7.0) | Control group  - VO_2_max (l/min): 3.3 (SD 1.2)  - VO_2_max (ml/kg/min): 48.0 (SD 11.0) |

**Blood serum concentration outcomes and between groups comparison**

** statistically significant difference between groups*

*^‡^ statistically significant difference within groups (pre-post)*

| Mean 12 weeks after baseline for men | | | |
| --- | --- | --- | --- |
| 4-mile group  - Total cholesterol (mg%): 174 (SD 18)  - Triglycerides (mg%): 70 (SD 34)  - HDL (mg%): 48 (SD 7) | 2-mile group  - Total cholesterol (mg%): 158 (SD 24)  - Triglycerides (mg%): 75 (SD 20)  - HDL (mg%): 42 (SD 10) | Interval group  - Total cholesterol (mg%): 165 (SD 19)  - Triglycerides (mg%): 67 (SD 14)  - HDL (mg%): 47 (SD 8) | Control group  - Total cholesterol (mg%): 169 (SD 34)  - Triglycerides (mg%): 108 (SD 60)  - HDL (mg%): 42 (SD 5) |
| Mean 12 weeks after baseline for women | | | |
| 4-mile group  - Total cholesterol (mg%): 174 (SD 18)  - Triglycerides (mg%): 81 (SD 24)  - HDL (mg%): 60 (SD 11) | 2-mile group  - Total cholesterol (mg%): 162 (SD 24)  - Triglycerides (mg%): 81 (SD 23)  - HDL (mg%): 51 (SD 12) | Interval group  - Total cholesterol (mg%): 171 (SD 19)  - Triglycerides (mg%): 71 (SD 17)  - HDL (mg%): 57 (SD 8) | Control group  - Total cholesterol (mg%): 165 (SD 35)  - Triglycerides (mg%): 73 (SD 20)  - HDL (mg%): 49 (SD 3) |

**More publications related to this study**

| Iltis PW, Thomas TR, Adeniran SB, Aguiar CA, Albers JJ. Different running programs: plasma lipids, apoproteins, and lecithin: cholesterol acyltransferase in middle-aged men. Annals of Sports Medicine. 1984;2(1):16-22. |
| --- |

**Reference**

| Toriola AL. Influence of 12-week jogging on body fat and serum lipids. British journal of sports medicine. 1984;18(1):13-7. |
| --- |

**Objectives**

| To examine the influence of different levels of distance running on percentage body fat and serum lipids in a group of male students. |
| --- |

**Description of participants included in the study**

| - Participants randomised: n=40 untrained male students.  - Source: University of Ife, Nigeria (students).  - Participants included in the analysis, n=40, mean age of 24.1 years (SD 3.0).   - 4.8 km running group: n=10. - 3.2 km running group: n=10. - 1.6 km running group: n=10. - Control group: n=10. |
| --- |

**Follow-up period and time-point assessments after baseline**

| - Follow-up: 12 weeks.  - Time-points: baseline and 12 weeks after baseline. |
| --- |

**Intervention (running program)**

| 4.8 km running group  - Duration: 12 weeks.  - Frequency: 3 times/week.  - Description: running 4.8 km at 85% of their maximal heart rate. Each jogging session was conducted as follows: initial warm-up exercise involving calisthenics (5 minutes), jogging 4.8 kilometres (18-25 minutes) and cool-down (5 minutes). | 3.2 km running group  - Duration: 12 weeks.  - Frequency: 3 times/week.  - Description: running 3.2 km at 85% of their maximal heart rate. Each jogging session was conducted as follows: initial warm-up exercise involving calisthenics (5 minutes), jogging 3.2 kilometres (12-18 minutes) and cool-down (5 minutes). | 1.6 km running group  - Duration: 12 weeks.  - Frequency: 3 times/week.  - Description: running 1.6 km at 85% of their maximal heart rate. Each jogging session was conducted as follows: initial warm-up exercise involving calisthenics (5 minutes), jogging 1.6 kilometres (5-10 minutes) and cool-down (5 minutes). |
| --- | --- | --- |

**Comparison group**

| Control group  - Participants were instructed not to participate in any vigorous physical activity during the training programme. |
| --- |

**Body composition outcomes and between groups comparison**

** statistically significant difference between 4.8 km group and control group*

^†^ *statistically significant difference between 3.2 km group and control group*

^§^ *statistically significant difference between 1.6 km group and control group*

^‡^ *statistically significant difference within groups (pre-post)*

| Mean 12 weeks after baseline | | | |
| --- | --- | --- | --- |
| 4.8 km group  - Body fat (%): 15.6 (SD 2.2)*^‡^ | 3.2 km group  - Body fat (%): 15.7 (SD 1.4)^†‡^ | 1.6 km group  - Body fat (%): 15.8 (SD 1.7)^§‡^ | Control group  - Body fat (%): 16.5 (SD 1.3) |

**Cardiorespiratory outcomes and between groups comparison**

** statistically significant difference between 4.8 km group and control group*

^†^ *statistically significant difference between 3.2 km group and control group*

^§^ *statistically significant difference between 1.6 km group and control group*

^‡^ *statistically significant difference within groups (pre-post)*

| Not applicable | Not applicable | Not applicable | Not applicable |
| --- | --- | --- | --- |

**Blood serum concentration outcomes and between groups comparison**

** statistically significant difference between 4.8 km group and control group*

^†^ *statistically significant difference between 3.2 km group and control group*

^§^ *statistically significant difference between 1.6 km group and control group*

^‡^ *statistically significant difference within groups (pre-post)*

| Mean 12 weeks after baseline | | | |
| --- | --- | --- | --- |
| 4.8 km group  - Cholesterol (mmol/l): 4.66 (SD 0.22)  - Triglycerides (mmol/l): 2.0 (0.17) | 3.2 km group  - Cholesterol (mmol/l): 4.69 (SD 0.17)  - Triglycerides (mmol/l): 2.1 (0.20)^†‡^ | 1.6 km group  - Cholesterol (mmol/l): 4.60 (SD 0.25)  - Triglycerides (mmol/l): 2.13 (0.28) | Control group  - Cholesterol (mmol/l): 4.79 (SD 0.24)  - Triglycerides (mmol/l): 2.13 (0.34) |

**More publications related to this study**

| Mathur DN, Toriola AL. Twelve weeks jogging effects on selected cardiovascular risk factors in untrained healthy males. The Journal of sports medicine and physical fitness. 1984;24(3):259-62. |
| --- |

**Reference**

| Williams PT, Wood PD, Krauss RM, Haskell WL, Vranizan KM, Blair SN, et al. Does weight loss cause the exercise-induced increase in plasma high density lipoproteins? Atherosclerosis. 1983;47(2):173-85. |
| --- |

**Objectives**

| To investigate if change in body composition is an antecedent to the increases in plasma concentrations of HDL-C and HDL2 and the decreases of HDL3 acquired by the higher mileage runners in the exercise group. |
| --- |

**Description of participants included in the study**

| - Participants randomised: n=81 sedentary middle-aged (30-55 years of age) men  - Participants included in the analysis, n=64:   - Running group: n=36. - Control group: n=28.   - The article refers to other study to addition information (*Wood PD, Haskell WL, Blair SN, Williams PT, Krauss RM, Lindgren FT, et al. Increased exercise level and plasma lipoprotein concentrations: a one-year, randomised, controlled study in sedentary, middle-aged men. Metabolism: clinical and experimental. 1983;32(1):31-9*). |
| --- |

**Follow-up period and time-point assessments after baseline**

| - Follow-up: 1 year.  - Time-points: baseline, 3, 6, 9 and 12 months after baseline. |
| --- |
| p.s.: Results related to the 3 and 9 months of follow-up were not described. |

**Intervention (running program)**

| Running group  - Duration: 1 year.  - Frequency: 3-5 times/week.  - Description: the exercise participants joined a supervised exercise program based on jogging or running. Training heart rates were set at 70-85% of the maximal heart rate achieved during the baseline treadmill test and were maintained by pulse rate monitoring.  - The article refers to other study to addition information (*Wood PD, Haskell WL, Blair SN, Williams PT, Krauss RM, Lindgren FT, et al. Increased exercise level and plasma lipoprotein concentrations: a one-year, randomised, controlled study in sedentary, middle-aged men. Metabolism: clinical and experimental. 1983;32(1):31-9*). |
| --- |

**Comparison group**

| Control group  - Participants were asked to maintain their sedentary lifestyle for 1 year. |
| --- |

**Body composition outcomes and between groups comparison**

** statistically significant difference between groups*

*^‡^ statistically significant difference within groups (pre-post)*

| Pre-post mean difference 6 months after baseline | |
| --- | --- |
| Running group  - ΔBody weight (kg): -1.3 (SD 2.7) | Control group  - ΔBody weight (kg): not reported |
| Mean difference between 6 months and 1 year from baseline | |
| Running group  - ΔBody weight (kg): -0.2 (SD 1.6) | Control group  - ΔBody weight (kg): not reported |
| Pre-post mean difference 1 year after baseline | |
| Running group  - ΔBody weight (kg): -1.4 (SD 3.1)*  - ΔBody fat (%): -1.3 (SD 2.9)* | Control group  - ΔBody weight (kg): 1.1 (SD 2.9)  - ΔBody fat (%): 2.8 (SD 3.3) |
| p.s.: Lean body mass results and additional information of body weight and body fat were reported in graphs. | |

**Cardiorespiratory outcomes and between groups comparison**

** statistically significant difference between groups*

*^‡^ statistically significant difference within groups (pre-post)*

| Not applicable | Not applicable |
| --- | --- |

**Blood serum concentration outcomes and between groups comparison**

** statistically significant difference between groups*

*^‡^ statistically significant difference within groups (pre-post)*

| Pre-post mean difference 1 year after baseline | |
| --- | --- |
| Running group  - ΔHDL (mg/dl): 1.3 (SD 7.2) | Control group  - ΔHDL (mg/dl): 0.1 (SD 5.8) |

**More publications related to this study**

| Williams PT, Albers JJ, Krauss RM, Wood PD. Associations of lecithin: cholesterol acyltransferase (LCAT) mass concentrations with exercise, weight loss, and plasma lipoprotein subfraction concentrations in men. Atherosclerosis. 1990;82(1-2):53-8. |
| --- |
| Williams PT, Krauss RM, Vranizan KM, Albers JJ, Terry RB, Wood PD. Effects of exercise-induced weight loss on low density lipoprotein subfractions in healthy men. Arteriosclerosis. 1989;9(5):623-32. |
| Wood PD, Haskell WL, Blair SN, Williams PT, Krauss RM, Lindgren FT, et al. Increased exercise level and plasma lipoprotein concentrations: a one-year, randomised, controlled study in sedentary, middle-aged men. Metabolism: clinical and experimental. 1983;32(1):31-9. |
| Williams PT, Wood PD, Haskell WL, Vranizan K. The effects of running mileage and duration on plasma lipoprotein levels. JAMA : the journal of the American Medical Association. 1982;247(19):2674-9. |

**Reference**

| Wood PD, Haskell WL, Blair SN, Williams PT, Krauss RM, Lindgren FT, et al. Increased exercise level and plasma lipoprotein concentrations: a one-year, randomised, controlled study in sedentary, middle-aged men. Metabolism: clinical and experimental. 1983;32(1):31-9. |
| --- |

**Objectives**

| To determine the dose-response effect of exercise training in relation to plasma lipoprotein concentrations, with particular reference to HDL and its subfractions. |
| --- |

**Description of participants included in the study**

| - Participants randomised: n=81 sedentary men.  - Source: Stanford University (employees).  - Participants included in the analysis, n=78:   - Running group: n=46, mean age of 45.3 years (dispersion or uncertainty not reported). - Control group: n=32, mean age of 46.2 years (dispersion or uncertainty not reported). |
| --- |

**Follow-up period and time-point assessments after baseline**

| - Follow-up: 1 year  - Time-points: baseline, 3, 6, 9 and 12 months after baseline (however this article only reported results at baseline and after 12 months). |
| --- |

**Intervention (running program)**

| Running group  - Duration: 1 year.  - Frequency: 3-5 times/week.  - Description: supervised exercise program, with meetings held initially 3 days/week. At first these sessions consisted of a period of calisthenics and muscle stretching, a 5 min warm-up period of walking or slow jogging, a 25 min work phase of running at 70-85% of capacity, 16 and a 5 min cool-down period. After 2-3 weeks, the participants were asked to add a fourth day of exercise, and by 8-10 weeks a fifth day, and longer exercise sessions, were recommended. Exercise intensity during the work phase was maintained at 70-85% of capacity throughout the study. |
| --- |

**Comparison group**

| Control group  - Participants were asked to remain sedentary for 1 year. |
| --- |

**Body composition outcomes and between groups comparison**

** statistically significant difference between groups*

*^‡^ statistically significant difference within groups (pre-post)*

| Pre-post mean difference 1 year after baseline | |
| --- | --- |
| Running group  - ΔBody weight (kg): -1.9 (SD 3.8)  - ΔBody fat (%): -1.8 (SD 3.6) | Control group  - ΔBody weight (kg): 0.6 (SD 3.9)  - ΔBody fat (%): 2.1 (SD 4.4) |
| Running-control group difference after 1 year of the change from baseline between groups (net change)  - ΔBody weight (kg): -2.5 (SE 0.9)*  - ΔBody fat (%): -3.8 (SE 1.0)* | |

**Cardiorespiratory outcomes and between groups comparison**

** statistically significant difference between groups*

*^‡^ statistically significant difference within groups (pre-post)*

| Pre-post mean difference 1 year after baseline | |
| --- | --- |
| Running group  - ΔResting heart rate (bpm): -9.2 (SD 7.2)  - ΔVO_2_max (ml/kg/min): 7.5 (SD 8.4) | Control group  - ΔResting heart rate (bpm): -2.4 (SD 9.7)  - ΔVO_2_max (ml/kg/min): -1.4 (SD 4.1) |
| Running-control group difference after 1 year of the change from baseline between groups (net change)  - ΔResting heart rate (bpm): -6.8 (SE 2.1)*  - ΔVO_2_max (ml/kg/min): 9.0 (SE 1.6)* | |

**Blood serum concentration outcomes and between groups comparison**

** statistically significant difference between groups*

*^‡^ statistically significant difference within groups (pre-post)*

| Running group (pre-post mean difference after 1 year)  - ΔTotal cholesterol (mg/dl): -4.9 (SD 19.0)  - ΔTotal triglycerides (mg/dl): -8.0 (SD 42.9)  - ΔHDL (mg/dl): 1.8 (SD 8.0)  - ΔHDL_2_ mass (mg/dl): 26.1 (SD 55.8)  - ΔHDL_3_ mass (mg/dl): -7.2 (SD 25.3)  - ΔLDL (mg/dl): -5.2 (SD 16.9)  - ΔVLDL (mg/dl): -1.5 (SD 10.3)  - ΔApolipoprotein A-I (mg/dl): 4.9 (SD 12.6)  - ΔApolipoprotein A-II (mg/dl): -1.2 (SD 6.0)  - ΔApolipoprotein B (mg/dl): -1.4 (SD 12.6) | Control group (pre-post mean difference after 1 year)  - ΔTotal cholesterol (mg/dl): 1.8 (SD 25.3)  - ΔTotal triglycerides (mg/dl): 3.8 (SD 39.5)  - ΔHDL (mg/dl): 0.5 (SD 6.1)  - ΔHDL_2_ mass (mg/dl): 10.5 (SD 55.0)  - ΔHDL_3_ mass (mg/dl): 2.7 (SD 39.4)  - ΔLDL (mg/dl): 1.3 (SD 20.4)  - ΔVLDL (mg/dl): -0.1 (SD 10.9)  - ΔApolipoprotein A-I (mg/dl): 3.7 (SD 12.6)  - ΔApolipoprotein A-II (mg/dl): -0.1 (SD 6.1)  - ΔApolipoprotein B (mg/dl): -0.7 (SD 12.2) |
| --- | --- |
| Running-control group difference after 1 year of the change from baseline between groups (net change)  - ΔTotal cholesterol (mg/dl): -6.6 (SE 5.3)  - ΔTotal triglycerides (mg/dl): -11.7 (SE 9.4)  - ΔHDL (mg/dl): 1.4 (SE 1.6)  - ΔHDL_2_ mass (mg/dl): 15.6 (SE 12.8)  - ΔHDL_3_ mass (mg/dl): -9.9 (SE 7.8)  - ΔLDL (mg/dl): -6.6 (SE 4.4)  - ΔVLDL (mg/dl): -1.4 (SE 2.5)  - ΔApolipoprotein A-I (mg/dl): 1.2 (SE 2.9)  - ΔApolipoprotein A-II (mg/dl): -1.1 (SE 1.4)  - ΔApolipoprotein B (mg/dl): -0.7 (SE 2.8) | |

**More publications related to this study**

| Williams PT, Albers JJ, Krauss RM, Wood PD. Associations of lecithin: cholesterol acyltransferase (LCAT) mass concentrations with exercise, weight loss, and plasma lipoprotein subfraction concentrations in men. Atherosclerosis. 1990;82(1-2):53-8. |
| --- |
| Williams PT, Wood PD, Krauss RM, Haskell WL, Vranizan KM, Blair SN, et al. Does weight loss cause the exercise-induced increase in plasma high density lipoproteins? Atherosclerosis. 1983;47(2):173-85. |
| Williams PT, Krauss RM, Vranizan KM, Albers JJ, Terry RB, Wood PD. Effects of exercise-induced weight loss on low density lipoprotein subfractions in healthy men. Arteriosclerosis. 1989;9(5):623-32. |
| Williams PT, Wood PD, Haskell WL, Vranizan K. The effects of running mileage and duration on plasma lipoprotein levels. JAMA : the journal of the American Medical Association. 1982;247(19):2674-9. |

**Reference**

| Williams PT, Wood PD, Haskell WL, Vranizan K. The effects of running mileage and duration on plasma lipoprotein levels. JAMA : the journal of the American Medical Association. 1982;247(19):2674-9. |
| --- |

**Objectives**

| To investigate the relationship between running dose and physiological response (lipid profile) over time. |
| --- |

**Description of participants included in the study**

| - Participants randomised: n=81 sedentary male university employees, aged 30 to 55 years.  - Source: Stanford University, California, USA.  - Participants included in the analysis, n=78:   - Running group: - For other outcomes: n=46. - Percentage body fat: n=40. - Treadmill variables: n=39. - Control group: - Other outcomes: n=32. - Percentage body fat: n=30. - Treadmill variables: n=27. |
| --- |

**Follow-up period and time-point assessments after baseline**

| - Follow-up: 1 year.  - Time-points: baseline, 3, 6, 9 and 12 months after baseline. |
| --- |

**Intervention (running program)**

| Running group  - Duration: 1 year.  - Frequency: 3-5 times/week.  - Description: training 3 days/week with 5 min warm-up period of slow walking or jogging, a 20-minute work-phase of jogging, and a 5 min cool-down period. After 2-3 weeks, the participants were asked to add an additional day of exercise, and at 8-10 weeks a 5^th^ day was recommended. At approximately 6 weeks, the exercise group was encouraged to increase gradually the duration of running until 45 min each session. During the entire training program, exercise intensity was maintained at 70% to 85% of the maximal heart rate. |
| --- |

**Comparison group**

| Control group  - Sedentary control group. |
| --- |

**Body composition outcomes and between groups comparison**

** statistically significant difference between groups*

*^‡^ statistically significant difference within groups (pre-post)*

| Pre-post mean difference after 3 moths from baseline | |
| --- | --- |
| Running group  - ΔBody fat (%): -0.20 (SD 2.78)* | Control group  - ΔBody fat (%): 1.67 (SD 3.45) |
| Pre-post mean difference after 6 moths from baseline | |
| Running group  - ΔBody fat (%): -0.30 (SD 3.46)* | Control group  - ΔBody fat (%): 2.51 (SD 3.17) |
| Pre-post mean difference after 9 moths from baseline | |
| Running group  - ΔBody fat (%): result not reported | Control group  - ΔBody fat (%): result not reported |
| Pre-post mean difference after 12 moths from baseline | |
| Running group  - ΔBody fat (%): -1.77 (SD 3.63)* | Control group  - ΔBody fat (%): 2.06 (SD 4.40) |

**Cardiorespiratory outcomes and between groups comparison**

** statistically significant difference between groups*

*^‡^ statistically significant difference within groups (pre-post)*

| Pre-post mean difference after 3 moths from baseline | |
| --- | --- |
| Running group  - ΔVO_2_max (ml/kg/min): 5.83 (SD 5.73)* | Control group  - ΔVO_2_max (ml/kg/min): 1.78 (SD 4.20) |
| Pre-post mean difference after 6 moths from baseline | |
| Running group  - ΔVO_2_max (ml/kg/min): 6.82 (SD 7.73)* | Control group  - ΔVO_2_max (ml/kg/min): 0.91 (SD 4.94) |
| Pre-post mean difference after 9 moths from baseline | |
| Running group  - ΔVO_2_max (ml/kg/min): result not reported | Control group  - ΔVO_2_max (ml/kg/min): result not reported |
| Pre-post mean difference after 12 moths from baseline | |
| Running group  - ΔVO_2_max (ml/kg/min): 7.52 (SD 8.38)* | Control group  - ΔVO_2_max (ml/kg/min): -1.43 (SD 4.05) |

**Blood serum concentration outcomes and between groups comparison**

** statistically significant difference between groups*

*^‡^ statistically significant difference within groups (pre-post)*

| p.s.: Results for plasma lipid and lipoprotein cholesterol (HDL-C and LDL-C) concentrations were not reported for any time-point, however there were no statistically significant difference between groups for changes during 1 year of these concentrations. |
| --- |

**More publications related to this study**

| Williams PT, Albers JJ, Krauss RM, Wood PD. Associations of lecithin: cholesterol acyltransferase (LCAT) mass concentrations with exercise, weight loss, and plasma lipoprotein subfraction concentrations in men. Atherosclerosis. 1990;82(1-2):53-8. |
| --- |
| Williams PT, Krauss RM, Vranizan KM, Albers JJ, Terry RB, Wood PD. Effects of exercise-induced weight loss on low density lipoprotein subfractions in healthy men. Arteriosclerosis. 1989;9(5):623-32. |
| Wood PD, Haskell WL, Blair SN, Williams PT, Krauss RM, Lindgren FT, et al. Increased exercise level and plasma lipoprotein concentrations: a one-year, randomised, controlled study in sedentary, middle-aged men. Metabolism: clinical and experimental. 1983;32(1):31-9. |
| Williams PT, Wood PD, Krauss RM, Haskell WL, Vranizan KM, Blair SN, et al. Does weight loss cause the exercise-induced increase in plasma high density lipoproteins? Atherosclerosis. 1983;47(2):173-85. |

**Reference**

| Wilmore JH, Davis JA, O'Brien RS, Vodak PA, Walder GR, Amsterdam EA. Physiological alterations consequent to 20-week conditioning programs of bicycling, tennis, and jogging. Medicine and science in sports and exercise. 1980;12(1):1-8. |
| --- |

**Objectives**

| To investigate the physiological benefits of free-wheel bicycling and tennis. |
| --- |

**Description of participants included in the study**

| - Participants randomised: n=38 sedentary male employees.  - Source: University of California, Davis Police and Fire Departments, USA.  - Participants included in the analysis, n=38:   - Running group: n=9, mean age of 35.6 years (SD 8.3). - Bicycling group: n=9, mean age of 37.0 years (SD 8.9). - Tennis group: n=10, mean age of 29.0 years (SD 6.6). - Control group: n=10, mean age of 32.3 years (SD 8.4). |
| --- |

**Follow-up period and time-point assessments after baseline**

| - Follow-up: 20 weeks.  - Time-points: baseline and 20 weeks after baseline. |
| --- |

**Intervention (running program)**

| Running group  - Duration: 20 weeks.  - Frequency: 3 times/week.  - Description: jogging for 30 min/day, 3 days/week (4.2 km/session, SD 0.5; 9.0 km/h, SD 1.0). A 15 min warm-up period of calisthenics preceded each training session. Subjects attempted to maintain individual training heart rates during sessions, which were obtained by adding 75% of the difference between maximal and resting heart rate (85%, SD 3.6). Participants trained on both paved and grassy terrain. |
| --- |

**Comparison group**

| Bicycling group  - Duration: 20 weeks.  - Frequency: 3 times/week.  - Description: cycling for 30 min/day, 3 days/week (11.7 km/session, SD 1.6; 22.7 km/h, SD 2.6). A 15 min warm-up period of calisthenics preceded each training session. Subjects attempted to maintain individual training heart rates during sessions, which were obtained by adding 75% of the difference between maximal and resting heart rate (83%, SD 1.9). Participants trained primarily on a flat asphalt terrain. | Tennis group  - Duration: 20 weeks.  - Frequency: 3 times/week.  - Description: jogging for 30 min/day, 3 days/week. A 15 min warm-up period of practice volleying and serving preceded each training session. Subjects attempted to maintain individual training heart rates during sessions, which were obtained by adding 75% of the difference between maximal and resting heart rate (64%, SD 5.3). | Control group  Not described. |
| --- | --- | --- |

**Body composition outcomes and between groups comparison**

** statistically significant difference between running group and control group*

^†^ *statistically significant difference between running group and bicycling group*

^§^ *statistically significant difference between running group and tennis group*

^‡^ *statistically significant difference within groups (pre-post)*

| Mean 20 weeks after baseline | | | |
| --- | --- | --- | --- |
| Running group  - Body weight (kg): 77.9  - Lean weight (kg): 63.5  - Fat weight (kg): 14.5  - Relative fat (%): 17.8  - Sum of 5 skinfolds (mm): 80.4 | Bicycling group  - Body weight (kg): 85.3  - Lean weight (kg): 67.4  - Fat weight (kg): 18.0  - Relative fat (%): 19.6  - Sum of 5 skinfolds (mm): 87.5 | Tennis group  - Body weight (kg): 87.4  - Lean weight (kg): 67.3  - Fat weight (kg): 20.1  - Relative fat (%): 21.8  - Sum of 5 skinfolds (mm): 114.0 | Control group  - Body weight (kg): 79.4  - Lean weight (kg): 63.3  - Fat weight (kg): 16.1  - Relative fat (%): 19.8  - Sum of 5 skinfolds (mm): 89.5 |
| Pre-post mean difference 20 weeks after baseline | | | |
| Running group  - ΔBody weight (kg): -2.4  - ΔLean weight (kg): -0.2  - ΔFat weight (kg): -10.5  - ΔRelative fat (%): -10.1  - ΔSum of 5 skinfolds (mm): -14.2 | Bicycling group  - ΔBody weight (kg): -0.5  - ΔLean weight (kg): 1.5  - ΔFat weight (kg): -6.7  - ΔRelative fat (%): -7.5  - ΔSum of 5 skinfolds (mm): -9.8 | Tennis group  - ΔBody weight (kg): -1.0  - ΔLean weight (kg): -0.7  - ΔFat weight (kg): -2.0  - ΔRelative fat (%): -0.5  - ΔSum of 5 skinfolds (mm): -4.5 | Control group  - ΔBody weight (kg): -0.9  - ΔLean weight (kg): -0.2  - ΔFat weight (kg): -3.6  - ΔRelative fat (%): -2.5  - ΔSum of 5 skinfolds (mm): -10.6 |
| p.s.: Dispersion or uncertainty measures were not reported. | | | |

**Cardiorespiratory outcomes and between groups comparison**

** statistically significant difference between running group and control group*

^†^ *statistically significant difference between running group and bicycling group*

^§^ *statistically significant difference between running group and tennis group*

^‡^ *statistically significant difference within groups (pre-post)*

*R: respiratory exchange ratio*

*V_E_: pulmonary ventilation*

| Mean 20 weeks after baseline  - Submaximal cycle ergometer test at 49 W (300 kpm/min) (dispersion of uncertainty not reported for any outcome) | | | |
| --- | --- | --- | --- |
| Running group  - Sub heart rate (bpm): 96.1  - VO_2_submax (l/min): 0.96  - V_E_ (l/min): 22.9  - R: 0.78 | Bicycling group  - Sub heart rate (bpm): 99.4  - VO_2_submax (l/min): 0.99  - V_E_ (l/min): 23.7  - R: 0.76 | Tennis group  - Sub heart rate (bpm): 100.5  - VO_2_submax (l/min): 0.99  - V_E_ (l/min): 24.5  - R: 0.82 | Control group  - Sub heart rate (bpm): 97.8  - VO_2_submax (l/min): 0.96  - V_E_ (l/min): 24.6  - R: 0.81 |
| Pre-post mean difference 20 weeks after baseline  - Submaximal cycle ergometer test at 49 W (300 kpm/min) (dispersion of uncertainty not reported for any outcome) | | | |
| Running group  - ΔSub heart rate (bpm): -8.4  - ΔVO_2_submax (l/min): -6.8  - ΔV_E_ (l/min): -12.3  - ΔR: -4.9*^§^ | Bicycling group  - ΔSub heart rate (bpm): -7.0  - ΔVO_2_submax (l/min): -7.5  - ΔV_E_ (l/min): -4.4  - ΔR: -6.2 | Tennis group  - ΔSub heart rate (bpm): -8.5  - ΔVO_2_submax (l/min): -10.0  - ΔV_E_ (l/min): -4.3  - ΔR: 1.2 | Control group  - ΔSub heart rate (bpm): -8.4  - ΔVO_2_submax (l/min): -10.3  - ΔV_E_ (l/min): -5.0  - ΔR: -2.4 |
| Mean 20 weeks after baseline  - Submaximal cycle ergometer test at 98 W (600 kpm/min) (dispersion of uncertainty not reported for any outcome) | | | |
| Running group  - Sub heart rate (bpm): 119.1  - VO_2_submax (l/min): 1.57  - V_E_ (l/min): 38.2  - R: 0.86 | Bicycling group  - Sub heart rate (bpm): 120.9  - VO_2_submax (l/min): 1.63  - V_E_ (l/min): 38.3  - R: 0.81 | Tennis group  - Sub heart rate (bpm): 126.3  - VO_2_submax (l/min): 1.53  - V_E_ (l/min): 37.0  - R: 0.86 | Control group  - Sub heart rate (bpm): 125.9  - VO_2_submax (l/min): 1.59  - V_E_ (l/min): 42.8  - R: 0.91 |
| Pre-post mean difference 20 weeks after baseline  - Submaximal cycle ergometer test at 98 W (600 kpm/min) (dispersion of uncertainty not reported for any outcome) | | | |
| Running group  - ΔSub heart rate (bpm): -8.6  - ΔVO_2_submax (l/min): -6.0  - ΔV_E_ (l/min): -5.7  - ΔR: -2.3 | Bicycling group  - ΔSub heart rate (bpm): -9.0  - ΔVO_2_submax (l/min): -4.7  - ΔV_E_ (l/min): -9.0  - ΔR: -10.0 | Tennis group  - ΔSub heart rate (bpm): -7.8  - ΔVO_2_submax (l/min): -8.9  - ΔV_E_ (l/min): -8.6  - ΔR: -4.4 | Control group  - ΔSub heart rate (bpm): -6.7  - ΔVO_2_submax (l/min): -4.8  - ΔV_E_ (l/min): -0.5  - ΔR: -4.2 |
| Mean 20 weeks after baseline  - Maximal cycle ergometer test (dispersion of uncertainty not reported for any outcome) | | | |
| Running group  - VO_2_max (l/min): 3.45  - VO_2_max (ml/kg/min): 44.5  - V_E_ (l/min): 119.1  - R: 1.15 | Bicycling group  - VO_2_max (l/min): 3.56  - VO_2_max (ml/kg/min): 43.1  - V_E_ (l/min): 117.8  - R: 1.12 | Tennis group  - VO_2_max (l/min): not reported  - VO_2_max (ml/kg/min): not reported  - V_E_ (l/min): not reported  - R: not reported | Control group  - VO_2_max (l/min): not reported  - VO_2_max (ml/kg/min): not reported  - V_E_ (l/min): not reported  - R: not reported |
| Pre-post mean difference 20 weeks after baseline  - Maximal cycle ergometer test (dispersion of uncertainty not reported for any outcome) | | | |
| Running group  - ΔVO_2_max (l/min): 10.6^‡^  - ΔVO_2_max (ml/kg/min): 13.5^‡^  - ΔV_E_ (l/min): 16.0^‡^  - ΔR: 0.9 | Bicycling group  - ΔVO_2_max (l/min): 15.2^‡^  - ΔVO_2_max (ml/kg/min): 15.9^‡^  - ΔV_E_ (l/min): 20.7^‡^  - ΔR: 2.8 | Tennis group  - ΔVO_2_max (l/min): not reported  - ΔVO_2_max (ml/kg/min): not reported  - ΔV_E_ (l/min): not reported  - ΔR: not reported | Control group  - ΔVO_2_max (l/min): not reported  - ΔVO_2_max (ml/kg/min): not reported  - ΔV_E_ (l/min): not reported  - ΔR: not reported |
| Mean 20 weeks after baseline  - Maximal treadmill test (dispersion of uncertainty not reported for any outcome) | | | |
| Running group  - VO_2_max (l/min): 3.70  - VO_2_max (ml/kg/min): 47.8  - V_E_max (l/min): 127.1  - R: 1.12 | Bicycling group  - VO_2_max (l/min): 3.68  - VO_2_max (ml/kg/min): 44.3  - V_E_max (l/min): 129.8  - R: 1.08 | Tennis group  - VO_2_max (l/min): 3.82  - VO_2_max (ml/kg/min): 44.4  - V_E_max (l/min): 128.2  - R: 1.13 | Control group  - VO_2_max (l/min): 3.26  - VO_2_max (ml/kg/min): 41.1  - V_E_max (l/min): 111.8  - R: 1.09 |
| Pre-post mean difference 20 weeks after baseline  - Maximal treadmill test (dispersion of uncertainty not reported for any outcome) | | | |
| Running group  - ΔVO_2_max (l/min): 11.1*  - ΔVO_2_max (ml/kg/min): 13.3*  - ΔV_E_max (l/min): 23.8  - ΔR: 3.7 | Bicycling group  - ΔVO_2_max (l/min): 13.6  - ΔVO_2_max (ml/kg/min): 27.0  - ΔV_E_max (l/min): 20.7  - ΔR: 1.0 | Tennis group  - ΔVO_2_max (l/min): 4.4  - ΔVO_2_max (ml/kg/min): 5.7  - ΔV_E_max (l/min): 17.0  - ΔR: 3.7 | Control group  - ΔVO_2_max (l/min): -0.9  - ΔVO_2_max (ml/kg/min): -0.5  - ΔV_E_max (l/min): 3.8  - ΔR: 0.9 |
| p.s.: The article did not report the resting blood pressure (systolic and diastolic) results by each group, however the authors mentioned that there were no statistically significant difference between groups regarding to these outcomes. | | | |

**Blood serum concentration outcomes and between groups comparison**

** statistically significant difference between running group and control group*

^†^ *statistically significant difference between running group and bicycling group*

^§^ *statistically significant difference between running group and tennis group*

^‡^ *statistically significant difference within groups (pre-post)*

| Not applicable | Not applicable | Not applicable | Not applicable |
| --- | --- | --- | --- |

# Electronic Supplementary Material Appendix S3

**- Biomedical indices of health identified -**

Table of Contents

Body composition outcome measures 136

Cardiorespiratory outcome measures 137

Blood serum concentration outcome measures 138

# Body composition outcome measures

| Body mass* |
| --- |
| Lean body mass* |
| Body fat* |
| Body mass index (BMI)* |
| Body density |
| Body fat-free |
| Android body fat |
| Gynoid body fat |
| Body mineral content |
| Leg mineral content |
| Body mineral density |
| Leg mineral density |
| Arm fat |
| Arm lean mass |
| Leg fat |
| Leg lean mass |
| Subcutaneous adiposity tissue area |
| Visceral adiposity tissue area |
| Thigh fat area |
| Abdominal skinfold |
| Suprailiac skinfold |
| Thigh skinfold |
| Sum of skin-folds |
| Abdominal circumference |
| Waist circumference |
| Hip circumference |
| Trochanter circumference |
| Thigh circumference |
| Waist/hip ratio |
| Abdominal/trochanter ratio |
| Upper arm circumference |

* Biomedical indices of health included in the meta-analyses.

# Cardiorespiratory outcome measures

| Resting heart rate* | High frequency power R-R interval over night |
| --- | --- |
| Maximal oxygen uptake (VO_2_max)* | Low frequency power R-R interval over 24 hours |
| Exercise heart rate | Low frequency power R-R interval over night |
| Submaximal heart rate | Very low frequency power R-R interval over 24 hours |
| Maximum 24 hours heart rate | Low/high frequency power ratio R-R interval over 24 hours |
| Maximum heart rate over night | Low/high frequency power ratio R-R interval over night |
| Heart rate not specified | Mean arterial blood pressure |
| Left ventricular mass | Systolic blood pressure |
| Left ventricular mass index | Diastolic blood pressure |
| Left ventricular end-diastolic diameter | Submaximal oxygen uptake (VO_2_submax) |
| Left ventricular end-systolic diameter | Peak oxygen uptake (VO_2_peak) |
| Septum thickness, end diastole | Oxygen uptake at the anaerobic threshold |
| Left ventricular posterior wall thickness, end diastole | Oxygen uptake at blood lactate concentration of 2.0 mmol/l |
| Interventricular septum thickness in diastole | Oxygen uptake at blood lactate concentration of 4.0 mmol/l |
| Left ventricular volume end-diastolic biplane | Oxygen uptake at 6 METs |
| Left ventricular ejection fraction | Oxygen uptake averaged during exercise |
| Early diastole | Oxygen uptake at final 30 s of exercise |
| Early diastole deceleration time | Oxygen deficit |
| Late diastole | Submaximal ventilation |
| Early diastole/atrial contraction ratio | Maximal ventilation |
| Isovolumetric relaxation time | Peak ventilation |
| Right ventricular end-diastolic diameter | Submaximal respiratory exchange ratio |
| Tricuspid annular plane systolic excursion | Respiratory exchange ratio |
| Early diastolic velocity | Peak respiratory exchange |
| Late diastolic velocity | Resting metabolic rate |
| Peak systolic velocity | Metabolic energy |
| Early diastole/early diastolic velocity | Energy cost |
| Systolic longitudinal displacement, left ventricle | Excess post-exercise oxygen consumption |
| Standard deviation of all R-R intervals over 24 hours | Maximum power output |
| High frequency power R-R interval over 24 hours |  |

* Biomedical indices of health included in the meta-analyses.

# Blood serum concentration outcome measures

| Triglycerides* | Apolipoprotein A-I |
| --- | --- |
| Total cholesterol* | Apolipoprotein A-II |
| High density lipoprotein (HDL) cholesterol* | Apolipoprotein B |
| Low density lipoprotein (LDL) cholesterol* | Apolipoprotein E |
| Glucose | Apolipoprotein B/apolipoprotein A-I |
| Insulin | Serum cartilage oligometric matrix protein |
| Insulin sensitivity | Epinephrine |
| Homeostasis model assessment of insulin resistance | Norepinephrine |
| Total insulin-like growth factor I | Total thyroxide |
| Bioactive IGF-I | Free thyroxide |
| Free IGF-I | Lactate |
| Insulin-like growth factor binding protein 1 | High sensitive C-reactive protein |
| Insulin-like growth factor binding protein 2 | Lipocalin-2 |
| Insulin-like growth factor binding protein 3 | Resting free fatty acids |
| Triglycerides/HDL | Free fat acid - end of exercise |
| HDL/TC | Free fat acid - post-exercise |
| VLDL-TC | Phospholipids |
| LDL-TC | Endostatin |
| HDL-TC | Adiponectin |
| Cholesterol/High density lipoprotein | Vascular endothelial growth factor |
| HDL/Cholesterol | Growth hormone |
| Lecithin:cholesterol acyltransferase | Ferritin |
| High density lipoprotein subfraction 2 (HDL2) | Iron |
| High density lipoprotein subfraction 3 (HDL3) | Iron-binding capacity |
| HDL2/HDL3 | Transferrin saturation |
| Small LDL | Hemoglobin |
| Large LDL | Hematocrit |
| LDL peak flotation rate | Haptoglobin |
| LDL peak particle diameter | Vitamin D |
| Very low density lipoprotein (VLDL) | Parathyroid hormone |
| Small VLDL | Serum uric acid |
| Large VLDL | Bone alkaline phosphatase |
| VLDL (Sf 20-400) | Osteocalcin |
| Intermediate density lipoprotein (IDL) | C-terminal telopeptide fragment of type I collagen |
| Lipoprotein (a) | Tartrate-resistant acid phosphatase |
|  | Deoxypyridinoline |

* Biomedical indices of health included in the meta-analyses.

# Electronic Supplementary Material Appendix S4

**- Forest plots and meta-analyses -**

Table of Contents

Body composition outcome measures

Body weight

Overall and length of training subgroups 140

Gender subgroups 141

Lean body mass

Overall and length of training subgroups 142

Gender subgroups 143

Body fat

Overall and length of training subgroups 144

Gender subgroups 145

Body mass index (BMI)

Overall and length of training subgroups 146

Gender subgroups 147

Cardiorespiratory outcome measures

Resting heart rate

Overall and length of training subgroups 148

Gender subgroups 149

Maximal oxygen uptake (VO_2_max)

Overall and length of training subgroups 150

Gender subgroups 151

Blood serum concentration outcome measures

Triglycerides

Overall and length of training subgroups 152

Gender subgroups 153

Cholesterol

Overall and length of training subgroups 154

Gender subgroups 155

High-density lipoprotein (HDL)

Overall and length of training subgroups 156

Gender subgroups 157

Low-density lipoprotein (LDL)

Overall and length of training subgroups 158

Gender subgroups 159

#

# Body mass: overall and length of training subgroups

**Figure S1.** Overall and length of training subgroups meta-analyses for body weight (kg). “I-V Overall” represents the overall fixed-effect model weighted by the inverse-variance. “I-V Subtotal” represents the fixed-effect model weighted by the inverse-variance by length of training. “D+L Overall” represents the overall random-effects model weighted by the inverse of the variance within and between (tau-squared) studies. “D+L Subtotal” represents the random-effects model weighted by the inverse of the variance within and between (tau-squared) studies by length of training. WMD: weighted mean difference. N: number of participants. SD: standard deviation. I-V: inverse-variance. D+L: DerSimonian and Laird method with the estimate of heterogeneity being taken from the inverse-variance fixed-effect model. HI: high intensity. MI: moderate intensity. LI: low intensity. M: males. F: females. Wks: weeks.

# Body mass: gender subgroups

**Figure S2.** Gender subgroups meta-analyses for body weight (kg). “I-V Subtotal” represents the fixed-effect model weighted by the inverse-variance by gender. “D+L Subtotal” represents the random-effects model weighted by the inverse of the variance within and between (tau-squared) studies by gender. WMD: weighted mean difference. N: number of participants. SD: standard deviation. I-V: inverse-variance. D+L: DerSimonian and Laird method with the estimate of heterogeneity being taken from the inverse-variance fixed-effect model. HI: high intensity. LI: low intensity. M: males. F: females. Wks: weeks.

# Lean body mass: overall and length of training subgroups

**Figure S3.** Overall and length of training subgroups meta-analyses for lean body mass (kg). “I-V Overall” represents the overall fixed-effect model weighted by the inverse-variance. “I-V Subtotal” represents the fixed-effect model weighted by the inverse-variance by length of training. “D+L Overall” represents the overall random-effects model weighted by the inverse of the variance within and between (tau-squared) studies. “D+L Subtotal” represents the random-effects model weighted by the inverse of the variance within and between (tau-squared) studies by length of training. WMD: weighted mean difference. N: number of participants. SD: standard deviation. I-V: inverse-variance. D+L: DerSimonian and Laird method with the estimate of heterogeneity being taken from the inverse-variance fixed-effect model. M: males. F: females. Wks: weeks.

# Lean body mass: gender subgroups

**Figure S4.** Gender subgroups meta-analyses for lean body mass (kg). “I-V Subtotal” represents the fixed-effect model weighted by the inverse-variance by gender. “D+L Subtotal” represents the random-effects model weighted by the inverse of the variance within and between (tau-squared) studies. WMD: weighted mean difference. N: number of participants. SD: standard deviation. I-V: inverse-variance. D+L: DerSimonian and Laird method with the estimate of heterogeneity being taken from the inverse-variance fixed-effect model. M: males. F: females. Wks: weeks.

# Body fat: overall and length of training subgroups

**Figure S5.** Overall and length of training subgroups meta-analyses for percentage body fat. “I-V Overall” represents the overall fixed-effect model weighted by the inverse-variance. “I-V Subtotal” represents the fixed-effect model weighted by the inverse-variance by length of training. “D+L Overall” represents the overall random-effects model weighted by the inverse of the variance within and between (tau-squared) studies. “D+L Subtotal” represents the random-effects model weighted by the inverse of the variance within and between (tau-squared) studies by length of training. WMD: weighted mean difference. N: number of participants. SD: standard deviation. I-V: inverse-variance. D+L: DerSimonian and Laird method with the estimate of heterogeneity being taken from the inverse-variance fixed-effect model. M: males. F: females. Wks: weeks.

# Body fat: gender subgroups

**Figure S6.** Gender subgroups meta-analyses for percentage body fat. “I-V Subtotal” represents the fixed-effect model weighted by the inverse-variance by gender. “D+L Subtotal” represents the random-effects model weighted by the inverse of the variance within and between (tau-squared) studies by gender. WMD: weighted mean difference. N: number of participants. SD: standard deviation. I-V: inverse-variance. D+L: DerSimonian and Laird method with the estimate of heterogeneity being taken from the inverse-variance fixed-effect model. M: males. F: females. Wks: weeks.

# Body mass index (BMI): overall and length of training subgroups

**Figure S7.** Overall and length of training subgroups meta-analyses for body mass index (kg/m^2^). “I-V Overall” represents the overall fixed-effect model weighted by the inverse-variance. “I-V Subtotal” represents the fixed-effect model weighted by the inverse-variance by length of training. “D+L Overall” represents the overall random-effects model weighted by the inverse of the variance within and between (tau-squared) studies. “D+L Subtotal” represents the random-effects model weighted by the inverse of the variance within and between (tau-squared) studies by length of training. WMD: weighted mean difference. N: number of participants. SD: standard deviation. I-V: inverse-variance. D+L: DerSimonian and Laird method with the estimate of heterogeneity being taken from the inverse-variance fixed-effect model. Wks: weeks.

# Body mass index (BMI): gender subgroups

**Figure S8.** Gender subgroups meta-analyses for body mass index (kg/m^2^). “I-V Subtotal” represents the fixed-effect model weighted by the inverse-variance by gender. “D+L Subtotal” represents the random-effects model weighted by the inverse of the variance within and between (tau-squared) studies by gender. WMD: weighted mean difference. N: number of participants. SD: standard deviation. I-V: inverse-variance. D+L: DerSimonian and Laird method with the estimate of heterogeneity being taken from the inverse-variance fixed-effect model. Wks: weeks.

# Resting heart rate: overall and length of training subgroups

**Figure S9.** Overall and length of training subgroups meta-analyses for resting heart rate (b/min). “I-V Overall” represents the overall fixed-effect model weighted by the inverse-variance. “I-V Subtotal” represents the fixed-effect model weighted by the inverse-variance by length of training. “D+L Overall” represents the overall random-effects model weighted by the inverse of the variance within and between (tau-squared) studies. “D+L Subtotal” represents the random-effects model weighted by the inverse of the variance within and between (tau-squared) studies by length of training. WMD: weighted mean difference. N: number of participants. SD: standard deviation. I-V: inverse-variance. D+L: DerSimonian and Laird method with the estimate of heterogeneity being taken from the inverse-variance fixed-effect model. HI: high intensity. LI: low intensity. M: males. F: females. Wks: weeks.

# Resting heart rate: gender subgroups

**Figure S10.** Gender subgroups meta-analyses for resting heart rate (b/min). “I-V Subtotal” represents the fixed-effect model weighted by the inverse-variance by gender. “D+L Subtotal” represents the random-effects model weighted by the inverse of the variance within and between (tau-squared) studies by gender. WMD: weighted mean difference. N: number of participants. SD: standard deviation. I-V: inverse-variance. D+L: DerSimonian and Laird method with the estimate of heterogeneity being taken from the inverse-variance fixed-effect model. HI: high intensity. LI: low intensity. M: males. F: females. Wks: weeks.

# Maximal oxygen uptake (VO_2_max): overall and length of training subgroups

**Figure S11.** Overall and length of training subgroups meta-analyses for maximal oxygen uptake (ml/kg/min). “I-V Overall” represents the overall fixed-effect model weighted by the inverse-variance. “I-V Subtotal” represents the fixed-effect model weighted by the inverse-variance by length of training. “D+L Overall” represents the overall random-effects model weighted by the inverse of the variance within and between (tau-squared) studies. “D+L Subtotal” represents the random-effects model weighted by the inverse of the variance within and between (tau-squared) studies by length of training. WMD: weighted mean difference. N: number of participants. SD: standard deviation. I-V: inverse-variance. D+L: DerSimonian and Laird method with the estimate of heterogeneity being taken from the inverse-variance fixed-effect model. HI: high intensity. MI: moderate intensity. LI: low intensity. M: males. F: females. Wks: weeks.

# Maximal oxygen uptake (VO_2_max): gender subgroups

**Figure S12.** Gender subgroups meta-analyses for maximal oxygen uptake (ml/kg/min). “I-V Subtotal” represents the fixed-effect model weighted by the inverse-variance by gender. “D+L Subtotal” represents the random-effects model weighted by the inverse of the variance within and between (tau-squared) studies by gender. WMD: weighted mean difference. N: number of participants. SD: standard deviation. I-V: inverse-variance. D+L: DerSimonian and Laird method with the estimate of heterogeneity being taken from the inverse-variance fixed-effect model. HI: high intensity. LI: low intensity. M: males. F: females. Wks: weeks.

# Triglycerides: overall and length of training subgroups

**Figure S13.** Overall and length of training subgroups meta-analyses for triglycerides (mg/dl). “I-V Overall” represents the overall fixed-effect model weighted by the inverse-variance. “I-V Subtotal” represents the fixed-effect model weighted by the inverse-variance by length of training. “D+L Overall” represents the overall random-effects model weighted by the inverse of the variance within and between (tau-squared) studies. “D+L Subtotal” represents the random-effects model weighted by the inverse of the variance within and between (tau-squared) studies by length of training. WMD: weighted mean difference. N: number of participants. SD: standard deviation. I-V: inverse-variance. D+L: DerSimonian and Laird method with the estimate of heterogeneity being taken from the inverse-variance fixed-effect model. HI: high intensity. LI: low intensity. M: males. F: females. Wks: weeks.

# Triglycerides: gender subgroups

**Figure S14.** Gender meta-analyses for triglycerides (mg/dl). “I-V Subtotal” represents the fixed-effect model weighted by the inverse-variance by gender. “D+L Subtotal” represents the random-effects model weighted by the inverse of the variance within and between (tau-squared) studies by gender. WMD: weighted mean difference. N: number of participants. SD: standard deviation. I-V: inverse-variance. D+L: DerSimonian and Laird method with the estimate of heterogeneity being taken from the inverse-variance fixed-effect model. HI: high intensity. LI: low intensity. M: males. F: females. Wks: weeks.

# Total cholesterol: overall and length of training subgroups

**Figure S15.** Overall and length of training subgroups meta-analyses for cholesterol (mg/dl). “I-V Overall” represents the overall fixed-effect model weighted by the inverse-variance. “I-V Subtotal” represents the fixed-effect model weighted by the inverse-variance by length of training. “D+L Overall” represents the overall random-effects model weighted by the inverse of the variance within and between (tau-squared) studies. “D+L Subtotal” represents the random-effects model weighted by the inverse of the variance within and between (tau-squared) studies by length of training. WMD: weighted mean difference. N: number of participants. SD: standard deviation. I-V: inverse-variance. D+L: DerSimonian and Laird method with the estimate of heterogeneity being taken from the inverse-variance fixed-effect model. M: males. F: females. Wks: weeks.

# Total cholesterol: gender subgroups

**Figure S16.** Gender subgroups meta-analyses for cholesterol (mg/dl). “I-V Subtotal” represents the fixed-effect model weighted by the inverse-variance by gender. “D+L Subtotal” represents the random-effects model weighted by the inverse of the variance within and between (tau-squared) studies by gender. WMD: weighted mean difference. N: number of participants. SD: standard deviation. I-V: inverse-variance. D+L: DerSimonian and Laird method with the estimate of heterogeneity being taken from the inverse-variance fixed-effect model. M: males. F: females. Wks: weeks.

# High-density lipoprotein (HDL) cholesterol: overall and length of training subgroups

**Figure S17.** Overall and length of training subgroups meta-analyses for high-density lipoprotein (mg/dl). “I-V Overall” represents the overall fixed-effect model weighted by the inverse-variance. “I-V Subtotal” represents the fixed-effect model weighted by the inverse-variance by length of training. “D+L Overall” represents the overall random-effects model weighted by the inverse of the variance within and between (tau-squared) studies. “D+L Subtotal” represents the random-effects model weighted by the inverse of the variance within and between (tau-squared) studies by length of training. WMD: weighted mean difference. N: number of participants. SD: standard deviation. I-V: inverse-variance. D+L: DerSimonian and Laird method with the estimate of heterogeneity being taken from the inverse-variance fixed-effect model. M: males. F: females. Wks: weeks.

# High-density lipoprotein (HDL) cholesterol: gender subgroups

**Figure S18.** Gender subgroups meta-analyses for high-density lipoprotein (mg/dl). “I-V Subtotal” represents the fixed-effect model weighted by the inverse-variance by gender. “D+L Subtotal” represents the random-effects model weighted by the inverse of the variance within and between (tau-squared) studies by gender. WMD: weighted mean difference. N: number of participants. SD: standard deviation. I-V: inverse-variance. D+L: DerSimonian and Laird method with the estimate of heterogeneity being taken from the inverse-variance fixed-effect model. M: males. F: females. Wks: weeks.

# Low-density lipoprotein (LDL) cholesterol: overall and length of training subgroups

**Figure S19.** Overall and length of training subgroups meta-analyses for low-density lipoprotein (mg/dl). “I-V Overall” represents the overall fixed-effect model weighted by the inverse-variance. “I-V Subtotal” represents the fixed-effect model weighted by the inverse-variance by length of training. “D+L Overall” represents the overall random-effects model weighted by the inverse of the variance within and between (tau-squared) studies. “D+L Subtotal” represents the random-effects model weighted by the inverse of the variance within and between (tau-squared) studies by length of training. WMD: weighted mean difference. N: number of participants. SD: standard deviation. I-V: inverse-variance. D+L: DerSimonian and Laird method with the estimate of heterogeneity being taken from the inverse-variance fixed-effect model. HI: high intensity. LI: low intensity. M: males. F: females. Wks: weeks.

# Low-density lipoprotein (LDL) cholesterol: gender subgroups

**Figure S20.** Gender subgroups meta-analyses for low-density lipoprotein (mg/dl). “I-V Subtotal” represents the fixed-effect model weighted by the inverse-variance by gender. “D+L Subtotal” represents the random-effects model weighted by the inverse of the variance within and between (tau-squared) studies by gender. WMD: weighted mean difference. N: number of participants. SD: standard deviation. I-V: inverse-variance. D+L: DerSimonian and Laird method with the estimate of heterogeneity being taken from the inverse-variance fixed-effect model. HI: high intensity. LI: low intensity. M: males. F: females. Wks: weeks.
